# Novel and Conserved miRNAs Among Brazilian Pine and Other Gymnosperms

**DOI:** 10.3389/fgene.2019.00222

**Published:** 2019-03-22

**Authors:** José Henrique Galdino, Maria Eguiluz, Frank Guzman, Rogerio Margis

**Affiliations:** ^1^Programa de Pós-graduação e Genética e Biologia Molecular, Departamento de Genética, Universidade Federal do Rio Grande do Sul - UFRGS, Porto Alegre, Brazil; ^2^Programa de Pós-graduação em Biologia Celular e Molecular, Centro de Biotecnologia, Universidade Federal do Rio Grande do Sul - UFRGS, Porto Alegre, Brazil; ^3^Departamento de Biofísica, Instituto de Biociências, Universidade Federal do Rio Grande do Sul - UFRGS, Porto Alegre, Brazil

**Keywords:** *Araucaria angustifolia*, Araucariaceae, microRNAs, non-coding RNAs, transcriptome

## Abstract

The knowledge about plant miRNAs has increased exponentially, with thousands of miRNAs been reported in different plant taxa using high throughput sequencing technologies and bioinformatic tools. Nevertheless, several groups of plants remain unexplored, and the gap of knowledge about conifer miRNAs is considerable. There is no sequence or functional information available on miRNAs in Araucariaceae. This group is represented in Brazil by only one species, *Araucaria angustifolia*, an endangered species known as Brazilian pine. In the present study, Brazilian pine has its transcriptome explored with respect to small RNAs, representing the first description in a member of the Araucariaceae family. The screening for conserved miRNAs in Brazilian pine revealed 115 sequences of 30 miRNA families. A total of 106 precursors sequences were predicted. Forty one comprised conserved miRNAs from 16 families, whereas 65 were annotated as novel miRNAs. The comparison of Brazilian pine precursors with sRNA libraries of other five conifer species indicates that 9 out 65 novel miRNAs are conserved among gymnosperms, while 56 seems to be specific for Brazilian pine or restricted to Araucariaceae family. Analysis comparing novel Brazilian pine miRNAs precursors and *Araucaria cunninghamii* RNA-seq data identified seven orthologs between both species. Mature miRNA identified by bioinformatics predictions were validated using stem-loop RT-qPCR assays. The expression pattern of conserved and novel miRNAs was analyzed in five different tissues of 3-month-old Araucaria seedlings. The present study provides insights about the nature and composition of miRNAs in an Araucariaceae species, with valuable information on miRNAs diversity and conservation in this taxon.

## Introduction

MicroRNAs (miRNAs) represent an important class of gene expression regulators (He and Hannon, 2004). These small nucleic acids correspond to 20–25 nt endogenous noncoding RNA sequences with considerable impact on virtually all biological processes (Budak and Akpinar, 2015). miRNA genes are transcribed as long precursor transcripts, called pri-miRNAs, which have the capacity to form a fold-back hairpin structure (Chorostecki et al., 2017). By dicer-like-1 enzyme (DL1) processing, precursors are cleaved and mature 5p/3p miRNA duplexes are produced (Zhang Y. et al., 2018). Usually, one of these mature miRNAs, is incorporated into RNA-induced silencing complexes (RISCs) (Paroo et al., 2007), and by the action of Argonaute 1 proteins (AGO1), these complexes act over mRNA targets, directing their sequestration or degradation (Pratt and MacRae, 2009).

The first record of a miRNA was performed in 1993 in a study with *C. elegans* (Lee et al., 1993). Since then, a series of studies were carried out and thousands of miRNAs were described in several animal and plant taxa (Cui et al., 2017). Among land plants, the miRNA characterization approach was extensively propagated, mainly, in Angiosperm model species like *Oryza sativa* (Wang, 2004), *Zea mays* (Mica, 2006), *Panicum virgatum* (Xie et al., 2010), *Glycine max* (Severin et al., 2010) among others. In miRBase (Kozomara and Griffiths-Jones, 2014) it is possible note that Angiosperm species miRNAs are remarkably predominant among Viridiplantae data. Even with predictions of novel miRNAs in some conifer species like *Picea abies* (Yakovlev et al., 2010), *Pinus taeda* (Lu et al., 2007), *Pinus densata* (Wan et al., 2012b), among others, the knowledge about the complexity of Gymnosperm miRNAs is very limited. Besides, in the field of small RNAs biology, a series of non-studied conifer species have great commercial and ecological importance, which means that a plethora of valuable genetic resources remains hidden in several taxa.

*Araucaria angustifolia* (Bertol.) Kuntze is the only endemic species of Gymnosperm with economic importance in Brazil (Steiner et al., 2008). This species, commonly named Brazilian pine, was the most important wood species from south Brazil in the past century (Santos et al., 2002). Representing valuable source of seeds, wood, fiber and resin, Brazilian pine was the target of extensive exploitation over decades, suffering massive population decrease (Steiner et al., 2008). Brazilian pine seeds are recalcitrant, maintaining a high metabolism status during the storage (Steiner et al., 2008). Consequently, under normal conditions, the seeds have a short conservation period, with substantial decrease in water potential and viability reduction at 4 months after harvest (Araldi et al., 2015). This recalcitrant feature compromises the conservation of Brazilian pine seeds and, consequently, hampers recovery efforts for degraded populations (Longhi et al., 2009). Currently, this species is classified as critically endangered, according to the International Union of Conservation of Nature Red List of Threatened Species (Thomas, 2013).

Brazilian pine has been targeted by some genetic studies mainly with a focus on somatic embryogenesis (Santos et al., 2002, 2010; Steiner et al., 2008). Recently, an RNA-seq data were used to perform a transcriptome comparative profile analysis of early development stages (Elbl et al., 2015). However, there is no information about miRNAs in this species. In the present study, the Illumina technology was used for sequencing a Brazilian pine small RNA library. Using a bioinformatic approach, a series of conserved and putative novel miRNAs, including their stem-loop structure, sequences, and some potential targets were reported. Also, predicted miRNAs were compared with sRNA sequences from six different conifer species and with RNA-seq data from *Araucaria cunninghamii* to investigate the presence of these miRNAs in other conifer taxa. Finally, stem-loop RT-qPCR was applied to validate bioinformatics outputs and analyze differential expression patterns of 12 conserved and 30 novel predicted miRNAs in five different tissues of 3-month Araucaria plants. The present data provide valuable information about Brazilian pine micro-RNA biology and will be very useful for future studies in this species as well as in Araucariaceae family.

## Materials and Methods

### Plant Material

For small RNA library preparation and sequencing, fresh leaves were collected from an adult Araucaria tree situated at coordinate 29°51′52.3″S 50°53′51.9″ in Rio Grande do Sul in Brazil.

For stem-loop RT-qPCR analysis, Araucaria seeds, obtained from the seasonal production, were used for germination and seedling production. Brazilian pine plants were grown under standard greenhouse conditions (Moreira-souza et al., 1994) until reaching 90 days (3-months). Then, samples of each replication were collected and frozen in an ultra-freezer at −80°C for subsequent molecular analysis.

### Small RNA Isolation and Illumina Sequencing

Total RNA was extracted from Brazilian pine fresh leaves with Trizol reagent (Invitrogen, CA, USA), following the standard protocol. The quantification of isolated RNA was determined using Nanodrop (Nanodrop Technologies, Wilmington, DE, USA). RNA sample was sent to Fasteris SA (Plan-les-Ouates, Switzerland) for sequencing. Using the Illumina HiSeq2000 platform, one sRNA library was constructed and sequenced, comprising 28,376,092 single-end reads with a length of 50 bases (NCBI accession number SRR8599283). The sRNA library building follows a series of standard steps, briefly described as follows: gel purification of the RNA fragments ranging from 20 to 30 nt, ligation of the 3p and 5p adapters and followed by gel purification, cDNA synthesis and cDNA gel purification, and, finally, PCR amplification to generate a cDNA colony template library for deep sequencing.

### Bioinformatic Analysis of sRNA Library

The Illumina small RNA library was processed. First, poor-quality bases, with a Fastq value below 30, were removed and adapter sequences were trimmed using Sickle-Quality-Base-Trimming (https://github.com/najoshi/sickle) and Cutadapt (https://cutadapt.readthedocs.io/en/stable/), respectively. Second, reads with unknown nucleotides (containing one or more “N” bases) were removed with Prin-Seq script (Schmieder and Edwards, 2011). Third, sequences shorter than 18 and longer than 25 nucleotides were also excluded. Finally, Plant small RNAs derived from rRNAs, tRNAs, snRNAs, snoRNAs deposited at the tRNAdb (Jühling et al., 2009), SILVA rRNA (Jühling et al., 2009), and NONCODE v3.0 (Jühling et al., 2009) databanks as well as from Gymnosperm mtRNA and cpRNA deposited at NCBI GenBank database (https://www.ncbi.nlm.nih.gov/) were used as references to align the reads by Bowtie (Langmead et al., 2009).

A set of 24 *A. angustifolia* mRNA-seq libraries were downloaded from NCBI Sequence Read Achieve (SRA) under bioproject PRJNA240554 (Elbl et al., 2015). The libraries were processed in order to cut poor-quality bases off the start and end of the reads, considering a fastq quality threshold of 30, and remove adapter sequences using Trim galore (https://www.bioinformatics.babraham.ac.uk/projects/trim_galore/). Next, the complete transcriptome was assembled with Trinity (Haas et al., 2013) using default parameters, and the predicted contigs were used as reference sequences for pre-miRNA prediction and identification of potential miRNA targets.

### Identification of Conserved Mature miRNAs

To identify conserved mature miRNAs, all mature miRNA sequences of Viridiplantae from miRBase (version 22) (Kozomara and Griffiths-Jones, 2014) were downloaded and mapped against *A. angustifolia* clean small reads with bowtie I (Langmead et al., 2009), allowing no mismatches.

### Prediction of Conserved and Novel miRNA Precursors

miR-PREFeR (Lei and Sun, 2014) was used for prediction of miRNA precursor sequences. This pipeline uses SAM files obtained from the mapping between small RNAs and the complete transcriptome assembled with Bowtie (Langmead et al., 2009). The candidate precursors obtained were manually revised with Tablet software (Milne et al., 2009), and confirmed according to the anchoring patterns: correctly stem-loop secondary structures should harbor the mature miRNA sequence at one arm of the stem and the antisense miRNA sequence (miRNA^*^), when detected, at the opposite arm.

The predicted precursor miRNAs were compared with miRBase stem-loop and mature sequences by BLASTn allowing no mismatches and classified into two categories, conserved precursors or putative novel precursors. The precursor stem-loop structures, as well as their minimal folding free energy (MFE), were analyzed using the annotation algorithm from the UEA sRNA toolkit (Moxon et al., 2008).

### Comparison Between Small RNA-Seq Data of Brazilian Pine and Other Conifer Species

Small RNA-seq libraries of another six-conifer species were downloaded from NCBI: *P. abies* (SRR824149; SRR824150) (Källman et al., 2013), *Ginkgo biloba* (SRR1658896, SRR1658901), *Cunninghamia lanceolata* (SRR066638) (Wan et al., 2012a), *Taxus mairei* (SRR797042) (Hao et al., 2012), and *Taxus wallichiana* (SRR1343578). The parameters for data cleaning and preprocessing were applied as described in the section Bioinformatic Analysis of sRNA Library. Next, all libraries were collapsed into a unique library, redundancy was removed, and reads were tagged with species code and read counts number. Using bowtie (Langmead et al., 2009), all libraries were mapped against Brazilian pine miRNA precursors, separately, allowing no mismatches. Using Tablet software (Milne et al., 2009), the anchoring patterns were visualized.

To investigate the presence of novel miRNAs predicted in *A. angustifolia* in another species of *Araucaria* genus, RNA-seq libraries of *A. cunninghamii* were downloaded from GenBank (accession PRJNA277081) and this data was analyzed as follows. The libraries were processed, low-quality reads and adaptors were trimmed. The complete transcriptome was *de novo* assembled with Trinity. Sequences of novel miRNA precursors from *A. angustifolia* were blasted against *A. cumnigamia* unigenes. Matched sequences comprising a total extension or at least the region flanked by mature and antisense miRNAs were folded using UEA sRNA workbench (Stocks et al., 2012).

### Prediction of miRNA Targets

The prediction of target genes of the mature miRNAs from the conserved and novel pre-miRNAs was performed using psRNAtarget (Dai and Zhao, 2011) using *A. angustifolia* assembled unigenes. Default parameters and the expectation value of 3.0 were considered in this analysis. Blast2Go software (Conesa and Götz, 2008) was used to understand the functions of the putative target genes.

### Biological Confirmation of Predicted miRNAs by Stem-Loop RT-qPCR

To validate and analyze patterns of expression of Brazilian pine predicted miRNAs, Stem-loop RT-qPCR method was performed. Seeds were germinated, and seedlings were grown until reaching an age of 3-months. Then, RNA samples were isolated using the Trizol reagent (Invitrogen, CA, USA). Five different tissues: young leaf, old leaf, stem, main root, and secondary root, were analyzed using four biological replicates. RNA quality was evaluated using 1% agarose gel electrophoresis and Nanodrop. cDNA was obtained for 42 miRNAs based on the stem-loop method (Chen, 2005). Primers sequences for stem-loop cDNA synthesis and mature miRNA expression are in Table S1. RT-qPCR reactions were performed in a CFX 384 RealTime PCR System (Bio-Rad). PCR mixes were carried out in a final volume of 10 μL, containing 5 μL of diluted cDNA (1:100) and 5 μL of reagents mix: 1X SYBR Green, 0.025 mM dNTP, 1X PCR buffer, 3 mM MgCl_2_, 0.25 U Platinum Taq DNA Polymerase (Invitrogen) and 200 nM of each reverse and forward primer. The RT-qPCR conditions were configured in this way: 94°C for 5 min, 40 cycles of 94°C for 15 s, 60°C for 10 and 25 s at 72°C. Melting curves were analyzed at the end of RT-qPCR runs to confirm the quality of amplified products. Samples were evaluated in four technical replicates. Using geNorm (https://genorm.cmgg.be/), normalizations for miRNA were performed with the Aang-miR171, Aang-nmiR009, and Aang-nmiR046, as the best combination of normalizers, following well-stablished criteria for miRNA RT-qPCR analysis (Kulcheski et al., 2010). To calculate the relative expression of miRNAs 2^−ΔΔCt^ method was used (Livak and Schmittgen, 2001). To carry out statistical analysis, ANOVA was applied using SAS software Version 9.4 (SAS Institute, Cary, NC, USA) and Duncan's multiple range test was performed to compare pairwise differences in expression, considering *p* < 0.05.

## Results

### Diversity of Small RNA in *Araucaria angustifolia*

**Figure 1:**
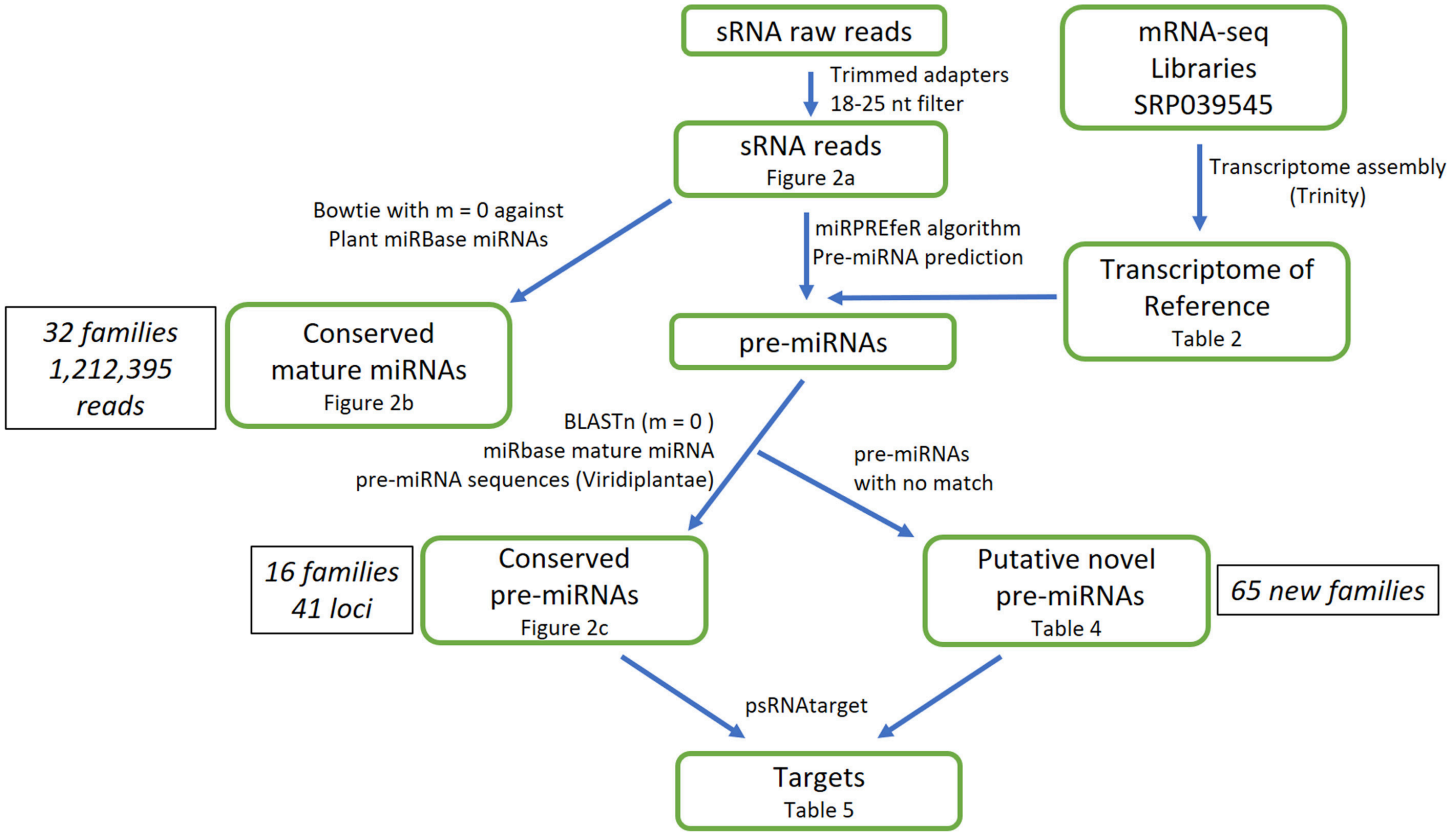
Overall procedure for analyzing Illumina small RNA library of *A. angustifolia*.

**Figure 2:**
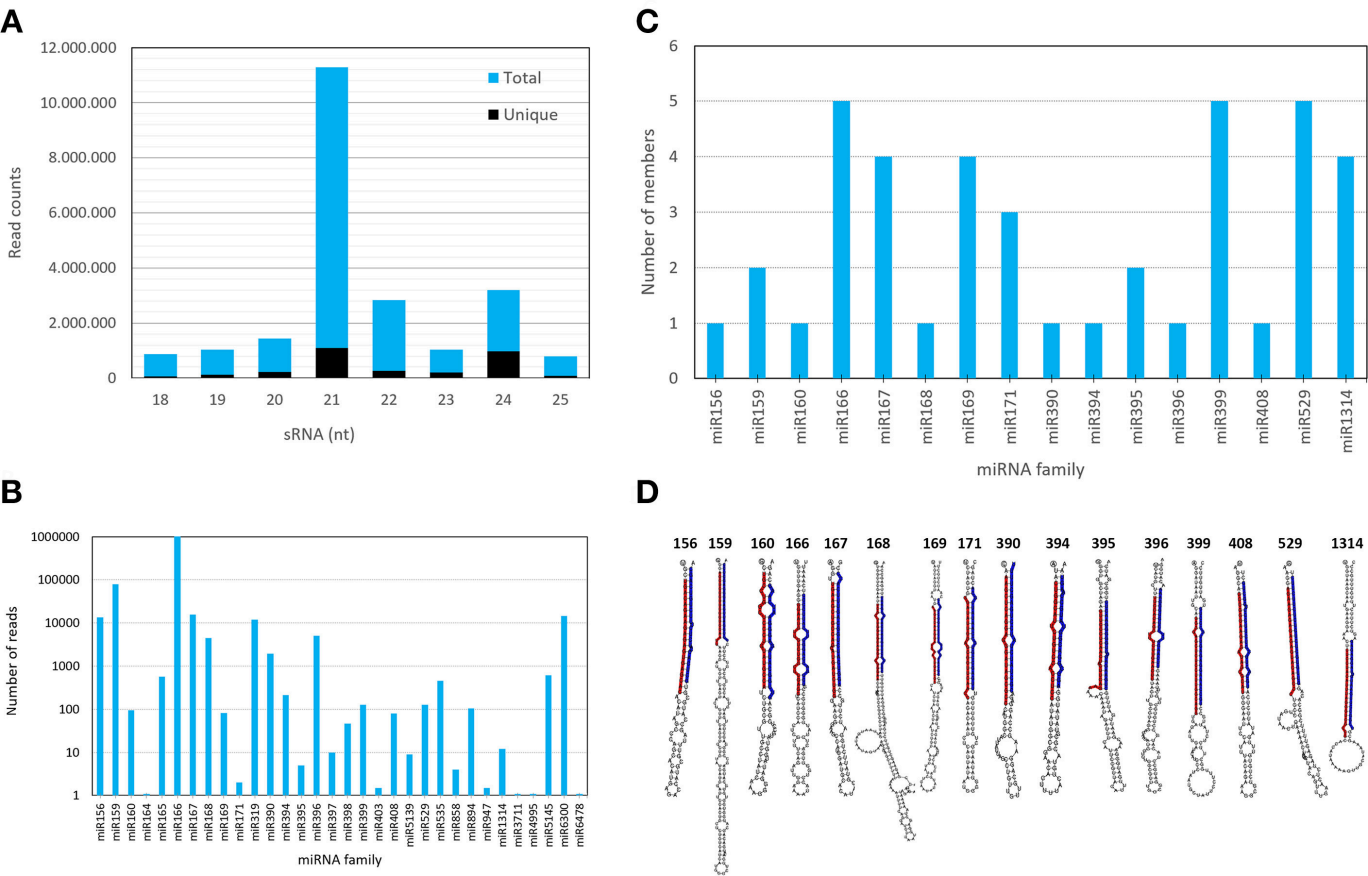
Characteristics of sRNA population, mature and pre-miRNAs identified in *A. angustifolia*. **(A)** Length distribution of unique and redundant *A. angustifolia* small RNAs. **(B)** Member numbers of identified miRNAs in each known miRNA family in *A. angustifolia*. **(C)** pre-miRNAs of conserved plant miRNA families identified in *A. angustifolia*. **(D)** Hairpin structures of pre-miRNAs representants of each conserved miRNA family predicted in *A. angustifolia*.

A total of 26,102,142 reads were obtained from the *A. angustifolia* small RNA library. This library was processed. Adapters, low-quality reads, base redundant reads, as well as reads longer than 25 and shorter than 18 nucleotides were removed. The clean library comprised 19,505,320 (74.73%) reads (Table 1), which were used for further analysis (Figure 1). The small RNA length distribution in Brazilian pine shown an interesting pattern. The length distribution and diversity of sRNAs are shown in the Figure 2A and Table S2. The highest abundance was observed in sequences with 21 and 24 nt, with 21 nt small RNA comprising more than 10 million sequences. The library composition analysis showed that 14.45% of reads matched miRNAs, 19.20% matched rRNA, 1.52% matched tRNAs, 0.06% matched snRNAs, 0.69% matched snoRNAs, 7.35% matched mtRNA, 6.64% matched cpRNA, 13.16% matched transposons (TEs), and 36.93% matched other RNAs (Table 1).

**Table 1:** Summary of data from *A. angustifolia* small RNA library sequencing.

**Type of small RNA**	**Number of reads**	**Percentage**
Total^*^	26,102,142	100.00%
<18 nt	3,847,477	14.74%
>25 nt	2,749,345	10.53%
18–25 nt	19,505,320	74.73%
miRNA	2,819,381	10.80%
rRNA	3,744,202	14.34%
tRNA	297,176	1.14%
snRNA	12,395	0.05%
snoRNA	135,169	0.52%
mtRNA	1,433,049	5.49%
cpRNA	1,295,819	4.96%
retrotransposon	2,567,205	9.84%
Other sRNA	7,200,924	27.59%

* *Reads with length up to 44 nt*.

### Identification of Conserved miRNAs in *Araucaria angustifolia*

All the Viridiplantae mature miRNAs deposited in miRBase were downloaded and mapped against *A. angustifolia* sRNA data with bowtie (Langmead et al., 2009). In this analysis, mismatches were not considered. As shown in Figure 2B and Table S3, 115 sequences matched miRNAs from 30 conserved families (miR156, miR159, miR160, miR164, miR165, miR166, miR167, miR168, miR169, miR171, miR319, miR390, miR394, miR395, miR396, miR397, miR398, miR399, miR403, miR408, miR529, miR535, miR858, miR894, miR947, miR1314, miR3711, miR4995, miR5139, miR5145, miR6300, and miR6478). The number of unique sequences per family as well as their read counts were highly variable, suggesting complex expression patterns of conserved miRNAs in *A. angustifolia* (Table S3).

### Identification of Pre-miRNAs Hairpin Sequences in *Araucaria angustifolia*

Brazilian pine has no nuclear genome sequenced yet. Instead, mRNA-seq data are available in GenBank. Then, 24 libraries were downloaded and the complete transcriptome was *de novo* assembled with Trinity. The assembly features are shown in Table 2. The assembled Araucaria transcriptome comprised 360,259 transcripts with an average length of 673 nt. To identify *A. angustifolia* miRNA precursors, the reference transcriptome, as well as the sRNA library, were loaded into miR-PREFeR (Lei and Sun, 2014). This tool follows the criteria for plant miRNA annotation, using expression patterns of miRNAs to predict plant miRNAs from small RNA-Seq data (Lei and Sun, 2014). In this way, 106 miRNA precursors, were predicted (Datas S1, S2). Using BLAST search, sequences of mature miRNA and antisense miRNA (miRNA^*^) of each precursor was compared with mature and stem-loop Viridiplantae data from the miRBase platform, and mismatches were not considered. Following this stringent condition, 41 precursor sequences (pre-miRNAs) of 16 conserved miRNA families were reported (Table 3, Table S4, and Data S1).

**Table 2:** Statistics on *Araucaria angustifolia* libraries and transcriptome *de novo* assembly.

Total reads	326,525,998
Number of transcripts	360,259
Median transcript length (nt)	256
Mean transcript length (nt)	673
Max transcript length (nt)	23,129
Number of transcripts >1 Kbases	77,861
N50	1,606

**Table 3:** pre-miRNAs and mature miRNAs identified in *A. angustifolia* matching miRNA families in other plant species.

**Loci**		**Pre-miRNA**			**miRNA 5P**			**miRNA 3P**		
**miRNA family**	**miRNA locus**	**Length**	**Read counts**	**MFE**	**Sequence**	**Read count**	**Length**	**Sequence**	**Read count**	**Length**
miR156	156	87	15,116	−48.5	TGACAGAAGAGAGTGAGCAC	13,367	20	GCTCACCATCTCTTTCTGTCAGC	1,583	23
miR159	159a	198	91,542	−71.8	CTTGGATTGAAGGGAGCTCC	77,010	20	AAGCTTCCTTCAGTCCAATCG	3	21
	159b	168	91,690	−91.2	AGCTCCCTTCGGTCCAATT	244	19	CTTGGATTGAAGGGAGCTCC	77,010	20
miR160	160	87	80	−47.1	TGCCTGGCTCCCTGTATGCCA	44	20	GTTGGCATAGAGGGAATCAAG	3	21
miR166	166a	71	1,008,365	−46.2	AAGGGGATTGCGGTCTGGCT	243	20	TCGGACCAGGCTTCATTCCCC	997,155	21
	166b	94	92,632	−42.6	GGACTGTTGTCTGGCTCGAAG	33	21	CCGGACCAGGCTTCATTCCCC	90,796	21
	166c	110	1,008,012	−54.5	GGAATGTTGTCTGGCTCGAGG	22	21	TCGGACCAGGCTTCATTCCCC	997,155	21
	166d	78	1,008,989	−47.7	GGAATGTTGTCTGGCTCGACT	780	21	TCGGACCAGGCTTCATTCCCC	997,155	21
	166e	96	1,063,754	−47.0	Non-detected	–	–	TCGGACCAGGCTTCATTCCCC	997,155	21
miR167	167a	120	13,848	−66.8	TGAAGCTGCCAGCATGATCTGA	11,024	22	AGATCATCTGGTAGCTTCAGC	580	21
	167b	117	4,785	−58.7	TGAAGCTGCCAGCATGATCTGG	2,611	22	TACCAGATCATGGTGGTGGCC	2	21
	167c^*^	91	4,788	−53.2	TGAAGCTGCCAGCATGATCTGG	2,611	22	AGGTCATCTGGCAGTTTCACC	6	21
	167d	109	4,783	−57.3	TGAAGCTGCCAGCATGATCTGG	2,611	22	TACCAGATCATGGTGGTGGCC	2	21
miR168	168	184	6,801	−74.0	TCGCTTGGTGCAGGTCGGGAA	4,436	21	CCCTGCTTGCATCAACTGAAT	332	21
miR169	169a	136	1,137	−99.7	AACAACTTGCCGGCTATTCTA	1	21	GGCAAGTTGTTCTCGGCTATG	918	21
	169b	131	195	−42.8	AAGCCAAGGATGAATTGCCGC	13	21	GGCAAGTTGTTCTTGGCTACG	72	21
	169c	155	1,802	−72.7	AAGCCAAGGATGATTTGCCGG	938	21	GGCAAGTTGTTCTTGGCTACG	72	21
	169d	158	2,886	−71.5	AAGCCAAGGATGATTTGCCGG	938	21	GGCAAGTTGTTCTCGGCTATG	918	21
miR171	171a	94	491	−50.4	GGATATTGGAGCGGTTCAACC	2	21	TTGAGCCGTGCCAATATCGCA	378	21
	171b	114	46	−63.1	GTGATGTTGGCTGGGCTCAAT	4	21	TGAGCCGTGCCAATATCACAA	16	21
	171c	84	36	−47.1	GTGATGTTGGCTGGGCTCAAT	4	21	TGAGCCGTGCCAATATCACAA	16	21
miR390	390a	83	1,998	−45.0	AAGCTCAGGAGGGATAGCGCC	1,952	21	CGCTATCTATCCTGAGCTTTT	13	23
miR394	394	80	611	−43.7	CTGGCATTCTGTCCACCTCC	312	21	AGGCGGACGGTATGCCAAGT	18	20
miR395	395a	112	2,712	−52.1	GTTCCCTCAACTACTTCAGAA	157	21	CTGAAGAGTTTGGGGGAACTC	2,157	21
	395b	95	2,514	−36.5	Non-detected	–	21	CTGAAGAGTTTGGGGGAACTC	2,157	21
miR396	396	120	5,246	−56.8	TTCCACAGCTTTCTTGAACTT	4,606	21	TTCAAGATTGCTGTGGGAAA	1	20
miR399	399a	114	136	−66.3	GGGGAGCTCTCCTTTGGCGGG	6	21	TGCCAAAGGAGAGTTGCCCTG	120	21
	399b	102	129	−64.0	GGGGGGCTCTCCTTTGGTGGG	2	21	TGCCAAAGGAGAGTTGCCCTG	120	21
	399c	111	134	−63.0	GGGGAGCTCTCCTTTGGCAGG	2	21	TGCCAAAGGAGAGTTGCCCTG	120	21
	399d	96	135	−53.2	GGGGAGCTCTCCTTTGGCGGG	6	21	TGCCAAAGGAGAGTTGCCCTG	120	21
	399e	112	134	−67.1	GGGGAGCTCTCCTTTGGCAGG	2	21	TGCCAAAGGAGAGTTGCCCTG	120	21
miR408	408	93	93	−55.6	GCCGGGAAGAGATAGCGCAT	1	20	TGCACTGCCTCTTCCCTGGCTG	79	22
miR529	529a	115	542	−51.9	AGAAGAGAGAGAGCACAGCCT	357	21	AGGCTGTGCTCTCTCTCTTC	1	21
	529b	89	544	−51.4	AGAAGAGAGAGAGCACAGCCT	357	21	GCTGTGCTCTCTCTCTTCTTC	2	21
	529c	119	540	−49.1	AGAAGAGAGAGAGCACAGCCT	357	21	Non-detected	–	–
	529d	104	540	−44.6	AGAAGAGAGAGAGCACAGCCT	357	21	Non-detected	–	–
	529e	103	463	−51.5	AGAAGAGAGAGAGTACAGCCC	35	21	GTTGTGCTCTCTCTCTTCTTC	383	21
miR1314	1314a^*^	71	6,878	−34.1	CTCCTACATTTAGGGTCGCCG	1,188	21	TCGGCCTTGAATGTTAGGAGAG	4,523	22
	1314b^*^	107	6,904	−53.5	CTCCTACATTTAGGGTCGCCG	1,188	21	TCGGCCTTGAATGTTAGGAGAG	4,523	22
	1314c^*^	135	5,853	−60.2	CTTCTAAATTTAAGGTCGCCG	560	21	TCGGCCTTGAATGTTAGGAGAG	4,523	22
	1314d^*^	124	5,921	−51.5	CTTCTAAATTTAAGGTCGCCG	560	21	TCGGCCTTGAATGTTAGGAGAG	4,523	22

* *Precursor miRNAs also identified in Araucaria cunninghamii*.

**Figure 3:**
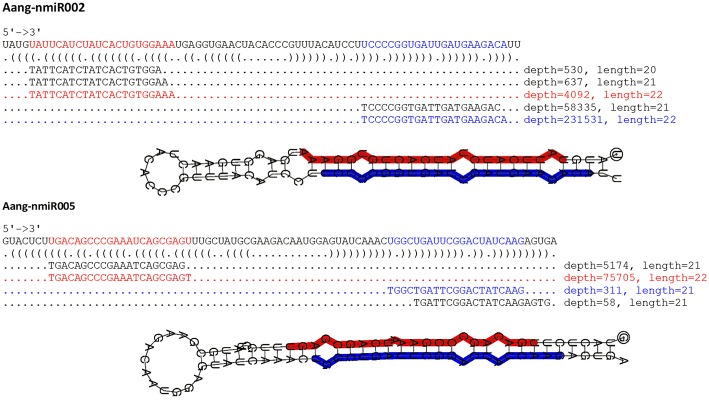
Predicted secondary structures of novel miRNAs identified in *A. angustifolia*. Secondary structures of Aang-miR002 and Aang-miR005 precursors, their locations and the expression of small RNAs mapped onto these precursors. The sequences corresponding to the most abundant 5p miRNA and most abundant 3p miRNA are labeled in blue and red, respectively. Values on the left side of the miRNA sequence represent read counts in the leaf library.

The most represented miRNA families were miR166, miR399, and miR529 with five members, followed by miR167 and miR 1314, with four members (Figures 2C,D). In contrast, with only one member, miR156, miR160, miR168, miR390, miR394, miR396, and miR408 were the less represented miRNA families. The length of conserved pre-miRNAs ranged from 71 to 198 nt and the minimal folding free energy (MFE) ranged from −34.15 to −99.70 kcal/mol (Table 3 and Table S4). The anchoring patterns, as well as stem-loop structures of the 41 known precursors, are shown in the Data S1. The other 65 precursors were considered putative novel pre-miRNAs (Figure 3 and Data S2). The length of these sequences ranged from 61 to 422 nt and the MFE ranged from −335.72 to −14.9 kcal/mol with an average negative folding value of −55, 9 kcal/mol (Table 4 and Table S5). As occurred with conserved pre-miRNAs, all novel pre-miRNAs showed regular hairpin structures. Also, in 58 out of 65 (89.23%) novel pre-miRNAs were possible to detect the antisense miRNA (miRNA^*^), which strongly support their prediction (Table 4, Table S5, and Data S2), indicating that these pre-miRNAs integrate the *A. angustifolia* miRNAome.

**Table 4:** Characteristics of novel pre-miRNAs and mature miRNAs identified in *A. angustifolia*.

**Loci**	**Pre-nmiRNA**			**nmiRNA 5P**			**nmiRNA 3P**		
**nmiRNA**	**Length**	**Read count**	**MFE**	**Sequence**	**Read count**	**Length**	**Sequence**	**Read count**	**Length**
Aang-nmiR001	93	303,638	−41.7	ACTGTGGGATGATGTCAAAAA	945	21	TTTGACATCACACCCGCGGTGA	294,457	22
Aang-nmiR002	78	299,835	−30.1	TATTCATCTATCACTGTGGAAA	4,092	22	TCCCCGGTGATTGATGAAGACA	231,531	22
Aang-nmiR003^*^	90	132,298	−40.4	CGTGGGCGACCGGGGAAAATT	51	21	TTTTCCCTGATCCGCCCATGCC	102,252	22
Aang-nmiR004	126	83,829	−94.3	TAGTAGACCAGACTCGCCATC	82,793	21	CGAGTCGGATCTACTACAACCT	243	22
Aang-nmiR005^*^	85	82,708	−38.0	TGACAGCCCGAAATCAGCGAGT	75,705	22	TGGCTGATTCGGACTATCAAG	311	21
Aang-nmiR006	90	76,565	−36.8	GTGGTCGGCGAGAAGAATCC	37,665	20	Non-detected	–	–
Aang-nmiR007	142	73,017	−63.1	TGGGCTTACATGTCTGTCGATG	141	22	TCAGCAGACATGTAGGCCAACC	71,657	22
Aang-nmiR008	117	62,299	−55.8	TCCCAAACATCGTCCAGAAATA	41,135	22	GTTTGGACGATGTTTGGAATG	548	21
Aang-nmiR009	132	47,232	−65.3	TCTCGAACATCCTGCAGCCATT	44,752	22	TGGCTGCACGACTTCGAGATA	546	21
Aang-nmiR010	243	37,279	−93.6	CTGGTAAACAGATGGGGCACT	181	21	CGCCCCATCTGATTACCGGTC	36,806	21
Aang-nmiR011	94	21,441	−30.8	TCACCGTGGACCGATGTAAAA	354	21	TTACGTCAGGTCCTCTGTGATT	17,626	22
Aang-nmiR012	90	18,386	−35.7	CCATCCGGCACTTGATGTCAAA	39	22	TGACGTCAGGTCCTCGATGGTT	16,623	22
Aang-nmiR013	94	13,462	−34.0	CCATTGAGCGCTTGGTGTCAAA	7,534	22	TAACATCAGGCCCTCGATGATT	310	22
Aang-nmiR014	69	13,373	−14.9	TCTTGGATTTATGGAAGACGAACC	8,615	24	AGGATGTTTTCATTAATCAAGAAC	19	24
Aang-nmiR015^*^	64	13,041	−24.9	GGTCGTCACGGTCGGTCCGCC	3,356	21	Non-detected	–	–
Aang-nmiR016	67	12,997	−38.5	CGAGGAAATAATGTGAAGAAC	1,048	21	TCTTCACATCCTTTCCTCGGA	7,684	21
Aang-nmiR017	81	11,945	−40.7	CGTGGGGGCGTTGGACAAAACC	353	22	TTTTGCCAATACCTCCCATGCC	11,321	22
Aang-nmiR018	114	11,727	−40.7	CCGTATTCATTAACCATAGAG	328	21	CCTGTGGTTAATGAATACATCG	7,218	22
Aang-nmiR019	76	11,414	−35.3	CCATCGAGGCTTGACGTCAAAA	119	22	TTACGTCAGGTCCTCTATGGTT	8,998	22
Aang-nmiR020	100	6,535	−45.4	AGGGCTGTCCGTGATTGGGCA	15	21	TCATACCCAATCACCGACAGC	3,981	21
Aang-nmiR021^*^	149	6,158	−61.0	Non-detected	–	–	TTTTTCCAATTCCGCCCATGCC	5,945	21
Aang-nmiR022	140	5,009	−44.1	CCGTATTCATTAACCATAGAG	328	21	CCTATGATTAATGAATACATCG	4,168	22
Aang-nmiR023	99	4,579	−59.2	TGACTGTCGTGGATGTATATC	4,464	21	TGTACATGCACGACGGTCACG	6	21
Aang-nmiR024	109	3,727	−55.0	CAGCCAAGAATGATTTGCCCGCC	565	23	GGCAGGTCATTCTTGGTGCT	1,089	20
Aang-nmiR025	177	3,525	−64.0	CCGCATCAGGTCTCCAAGGTG	3,447	21	Non-detected	–	–
Aang-nmiR026	88	3,344	−40.2	TGGATAGGAGGAGGATTCATG	1	21	TAAATCCTTCTGCTGTCCATA	3,062	21
Aang-nmiR027	75	2,601	−37.9	CCACCGTGGACCTGGTGTGAA	10	21	TCACGTCAGGACCTCGGTGGTT	1,810	22
Aang-nmiR028	102	2,574	−49.3	TCCGGAGACGTCGGCGGGGGC	1,114	21	Non-detected	–	–
Aang-nmiR029	126	2,184	−54.9	AAAACCATTGACTATCAAAAGA	47	22	TTTTGATAGCCAGTGGCAATC	1,364	21
Aang-nmiR030	127	1,798	−72.4	TGCGCCCTCGCGGCGGGCC	74	19	GCGCTGGCCGGCGGGCTTTC	454	20
Aang-nmiR031	123	1,751	−65.2	ACCTCGCCAACAATCTCAGC	110	22	TGAGATTGTTGGAGAGGTTCG	847	21
Aang-nmiR032	104	1,283	−43.3	AGAAGAGAGAAAGCACATCCC	814	21	GTTGTGCTCTCTCTCTTCTTC	383	21
Aang-nmiR033^*^	176	1,238	−68.2	TTACCACGCCCGCCCATGCCTA	379	22	GGCGTTGCCGGTCTGGTAAAA	571	21
Aang-nmiR034	61	1,232	−18.3	GTCCTATTCCGTTGGCCT	563	18	GAATAACGTGATAGGAGTCTG	12	21
Aang-nmiR035	127	1,203	−68.1	ATGCTTGTTATCTCTGTGCGGC	548	22	CCGCGCAGAAATAAAAGCATG	19	21
Aang-nmiR036^*^	112	1,122	−46.0	CCTTGTTCCTATTTACTGGCA	932	21	TCAATAAATAGGAACACAGGTT	133	22
Aang-nmiR037	122	855	−42.6	AGTCAACTCAAGTCTTTGAAA	14	21	TTAAAGATTTGAGTTGTCCAA	666	21
Aang-nmiR038	65	830	−37.9	AGTGGGAGGAACGGGCAAAAACT	96	23	TTTTCCCGGCTCCTCCCATTCC	662	22
Aang-nmiR039	112	777	−48.5	ATTGGACAACTCAATCTTTGA	260	21	CTCAAGGACTTGAGCTGTCCAA	113	22
Aang-nmiR040	135	713	−55.4	CAGCAAGTGGAAAACTAGAAT	11	21	TATTTCAGTTCTTCACTTGCT	506	21
Aang-nmiR041	114	668	−45.3	TGGGCTTACATGTCTGTCGATG	141	22	TCAGCAGATATGTCAGCCAACC	379	22
Aang-nmiR042	141	664	−69.0	CACATTTTTAGTCTGAAACTG	317	21	TTCAGACTAAAGATGTGTATT	235	21
Aang-nmiR043	81	590	−47.3	TCCGAATTCCGCGACGCTCCA	255	21	GGACCGTCGCTGAATTCGGAG	143	21
Aang-nmiR044^*^	104	559	−22.9	TTGCTGTCCATCAAAGAAGGC	377	21	Non-detected	–	–
Aang-nmiR045	143	465	−71.7	TTTCTGTGAACAAAATTTCAA	112	21	TTTGAAATTTTGGTCATAGAG	38	21
Aang-nmiR046	86	442	−42.8	AGTGGGATGCGAGGATAAGACT	259	22	TCTTTCCTACGCCTCCCATTCC	172	22
Aang-nmiR047	131	392	−45.2	AGAATTGAAAAACTTGCCTAT	126	21	AGGCATGTTTTTCAATTCTGA	40	21
Aang-nmiR048	69	370	−27.0	CCATTGAGCACTTGTTGTCAA	9	21	TTTTTTTGACATCAGGCCCTC	206	21
Aang-nmiR049	89	369	−45.6	TGATAAGGCCCTAATGACACAA	269	21	GTGTTATTTGGGCTTGTCATT	27	21
Aang-nmiR050	82	343	−44.6	TTATTGAATACTGGTGAAAGG	12	21	TTACCAGTCCTCAATGAGATC	279	21
Aang-nmiR051	198	330	−149.8	GTCTGCAGAGTGTATGGCCTG	272	21	CCTGCAGTCCAACATATACG	1	20
Aang-nmiR052	188	275	−67.6	TTCCAAAGCAGATAGATTGCCA	86	22	GCATCTGTCTGCTTCGGAATA	41	21
Aang-nmiR053	94	231	−64.6	GCAATGAATCGGCTGAATCGC	141	21	Non-detected	–	–
Aang-nmiR054	99	223	−61.4	TGACCGTCGTGGATGTATATC	175	21	TGCACGACGGTCACGACTGCC	14	21
Aang-nmiR055	67	218	−20.2	GTGGCCTATCGATCCTTTAG	33	20	CTAGAGGTGTCAGAAAAGTTAC	105	22
Aang-nmiR056	100	180	−34.4	CTTGATGATGATAACCGTTGACG	15	23	CACGGTTTGTCTGAAAGAT	43	19
Aang-nmiR057	135	176	−82.1	TGCTGAAATCGGTCGTACTGA	78	21	GGTACGATCGATTTCGGTATA	70	21
Aang-nmiR058	265	173	−102.0	TGGCATGACTTGCAAATTATG	9	21	CAATTTGTAAGGCCATGCTAAT	145	22
Aang-nmiR059	422	147	−335.7	AGTGTCCAGCATTTCTCGTCT	4	21	TGAGAAATGCTGGACACTTCT	127	21
Aang-nmiR060	98	147	−47.9	TGTTTCTACTGAGTTGGTTTCC	68	22	AACCTACTTAGTGAAAACATG	2	21
Aang-nmiR061	83	123	−36.0	CGTGGGCGTCTTGGACAAAGC	21	22	TTTTTCCAATGCCGCCCATGCC	91	22
Aang-nmiR062	85	101	−49.6	CCCGTATTGAAGATCAACCCA	11	21	GGTTGATCTTCAATATGGCGC	59	21
Aang-nmiR063	90	86	−54.8	TTTTGATTTTCAGTACGAATA	13	21	TTCGTACTGAAAATCAAAATC	8	21
Aang-nmiR064	158	72	−87.6	GTTTTAACTCATGGATATGCA	42	21	CATATCCATGAGTTAAAACCC	18	21
Aang-nmiR065	113	46	−46.9	TTTATTGATTTGATGCTAATGA	3	22	CTTAACACCAGACTAATGAACA	12	22

* *Correspond to pre-miRNAs also identified in Araucaria cunninghamii*.

### Comparison Between *Araucaria angustifolia* Predicted Precursors and Small RNA Data From Different Gymnosperms

As a set of mature miRNA sequences of 65 precursors did not match miRBase data, the main online repository for all miRNA sequences and annotation (Kozomara and Griffiths-Jones, 2014), they could be classified as potential species-specific miRNAs. However, the miRBase platform has a restricted miRNA annotation in gymnosperms. There are only four conifer species of three genera with miRNA sequences annotated in this platform, *C. lanceolata, P. abies, P. densata*, and *P. taeda*. In addition, a series of studies with plant miRNAs, including conifer miRNAs (Chen et al., 2013; Zhang et al., 2013; Li et al., 2017a), was published and several novel plant miRNAs were proposed, but these data were not present in the currently miRBase version. Thus, it is possible that novel miRNAs proposed in different species and classified as species-specific miRNAs could be also present in other taxa.

To avoid overestimation of novel miRNAs, and to provide a comprehensive comparison between conifer miRNAs, sRNA-seq libraries of five conifer species, *P. abies, G. biloba, C. lanceolata, T. mairei*, and *T. wallichiana*, were downloaded from GenBank (the accession codes were shown in Materials and Methods section). A comprehensive Phylogenetic relationship among this species is shown in Lu et al. (2014). The libraries were processed and only reads with length ranging from 18 to 25 nt remained. Then, using Bowtie (Langmead et al., 2009), the conifer sRNA libraries were mapped against the Brazilian pine miRNA precursors in two different ways. First, the small conifer libraries were mapped separately allowing no mismatches. Second, all libraries, including Brazilian pine sRNA, were collapsed, organized into unique reads, and mapped allowing no mismatches, and the mapping was visualized with Tablet (Milne et al., 2009).

All precursor sequences of miR156, miR159, miR160, miR166, miR167, miR168, miR390, and miR396 families matched with reads of all libraries, with high redundancy (Table 5). For the other conserved pre-miRNAs, the mapping pattern was not the same. For example, the four precursor members of conifer conserved family miR1314 (Berruezo et al., 2017) showed correspondent reads in four of seven libraries, with the highest redundancy in *G. biloba* and the minimal coverage in *T. wallichiana* library (Table 5).

**Table 5:** Mapping patterns of sRNAs from different gymnosperms against conserved and novel miRNA precursors predicted in *A. angustifolia*.

	**miRNA precursor**	***Cunninghamia lanceolata***	***Ginkgo biloba***	***Picea abies***	***Taxus mairei***	***Taxus wallichiana***	***Araucaria angustifolia***
Conserved miRNAs	Aang-miR156	283,795	4,702	230	21,967	1,574	15,118
	Aang-miR159ab	1	52	244	28	1	91,693
	Aang-miR160	38	269	270	48	2	80
	Aang-miR166abcde	4,983	719,138	433,596	21,426	4,574	1,063,754
	Aang-miR167abcd	33,472	5,437	955	779	45	13,848
	Aang-miR168	5,639	37,003	1,184	27,150	59,574	6,801
	Aang-miR169abcd	0	28	0	0	0	2,887
	Aang-miR171abc	17	10,214	89	3	4	491
	Aang-miR390a	395	8,922	1,974	164	52,965	1,999
	Aang-miR394	0	11,081	312	4	0	611
	Aang-miR395ab	0	2	0	2	0	2,722
	Aang-miR396	102	4,147	254	794	1,166	5,246
	Aang-miR399abcde	2	20	3	28	0	136
	Aang-miR408	325	47	0	190	22,972	94
	Aang-miR529abcde	0	77	58	174	11	1,086
	Aang-miR1314abcd	0	64,219	20	0	1	6,904
Novel miRNAs	Aang-nmiR006	0	116	0	24	1,151	110,647
	Aang-nmiR014	1,301	295	24	426	1,085	32,638
	Aang-nmiR025	355	3,118	242	2,441	787	5,040
	Aang-nmiR028	696	564	3	68	11,953	3,084
	Aang-nmiR031	0	126,984	42,987	1,402	163	1,751
	Aang-nmiR034	0	4	0	1,470	2,148	1,869
	Aang-nmiR046	0	31	0	41	742	442
	Aang-nmiR055	345	409	128	46	1,402	618
	Aang-nmiR063	43	252	57	60	357	454

**Figure 4:**
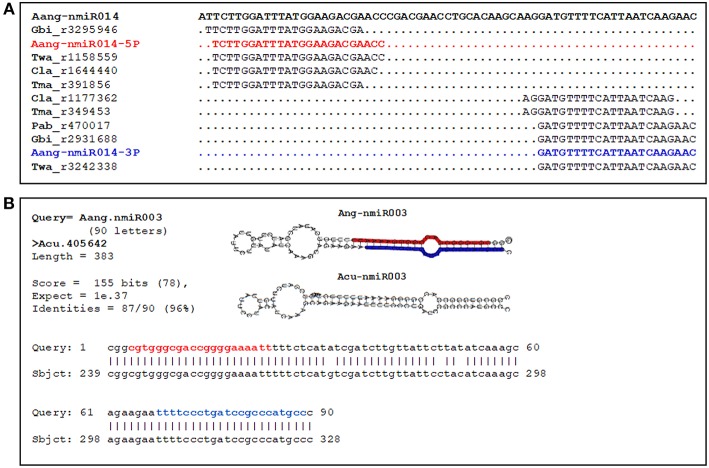
Novel pre-miRNAs identified in *A. angustifolia* and present in other conifer species. **(A)** Locations of small RNAs of conifer-species mapped onto the Aang-nmiR014 precursor. The sequences corresponding to the *A. angustifolia* (Aan) most abundant 5p and 3p miRNAs are colored in blue and red, respectively. Cla, *Cunninghamia lanceolata;* Gbi, *Ginkgo biloba*; Pbi, *Picea abies*; Tma, *Taxus mairei;* Twa, *Taxus wallichiana*. **(B)** BLAST searching output showing the identification of precursor Aang-nmiR003 in *Araucaria cunninghamii*.

Interestingly, 9 out of 65 novel pre-miRNAs (Aang-nmiR006, Aang-nmiR014, Aang-nmiR025, Aang-nmiR028, Aang-nmiR031, Aang-nmiR034, Aang-nmiR046, Aang-nmiR055, and Aang-nmiR063) also matched sRNA sequences of other conifer species (Table 5), and the mapping patterns suggest that these miRNAs could represent potential conserved miRNAs among gymnosperms. By visualizing the mapping patterns, it is possible to note that reads of libraries of different species aligned to precursors in the same way as miRNA and miRNA^*^ sequences from *A. angustifolia*, as illustrated in Figure 4A. An interesting characteristic of miRNA genes is that most loci are conserved across organisms (Mutum et al., 2016). Therefore, it is necessary to be careful to nominate novel miRNAs as species-specific (Taylor et al., 2014). These results suggest that, although the putative novel miRNAs did not match miRBase sequences, they not necessarily represent species-specific miRNAs. Instead, is possible that nine putative novel miRNAs predicted in *A. angustifolia* may have evolved early in Gymnosperm lineages. On the other hand, 56 out of 65 novel pre-miRNAs seem to be novel non-conserved miRNAs in Brazilian pine or specific at some lower taxonomic level, like the Araucariaceae family.

### Identification of Brazilian Pine Pre-miRNA Orthologs in *Araucaria cunninghamii*

To obtain insights about diversity and evolution of novel and conserved miRNA precursors predicted in *A. angustifolia*, these precursors were blast-searched against *A. cunninghamii* complete transcriptome assembled. In this way, five pre-miRNAs of two conserved miRNA families (miR167 and miR1314) and six putative novels pre-miRNAs (Aang-nmiR003, Aang-nmiR005, Aang-nmiR015, Aang-nmiR021, Aang-nmiR033, and Aang-miR036) predicted in *A. angustifolia* were identified in *A. cunninghamii* RNA-seq data (Datas S3, S4). Among the conserved miRNAs, all members of the family miR1314, only present in conifers, were found in *A. cunninghamii* (Data S3). In the pre-miRNA sequence alignments it was possible to note that base identity rates varied to 86% between Aang-miR1314a and putative Acun-miR1314a to 95% between Aang-miR1314d and putative Acun-miR1314d (Data S3). Among novel miRNAs base identity rates were also high, reaching 96% in the Aang-nmiR003/putative Acun-nmiR003 (Figure 4B) and Aang-nmiR033/putative Acun-nmiR033 pairs. In all cases mismatches appeared in loop regions, antisense miRNA (miRNA^*^) region and five to seven bases up or downstream mature miRNAs (Datas S3, S4). These results reinforce the validation of predicted miRNAs in this study and indicate that some novel miRNAs predicted in *A. angustifolia* are conserved in *Araucaria* genus or Araucariaceae family.

### Identification of Targets for Conserved and Novel Brazilian Pine Pre-miRNAs

To add information about the biological function of miRNAs in Brazilian pine, miRNA-targets were computationally predicted using the psRNATarget platform. In this analysis, conserved and putative novel predicted mature sequences were aligned to a set of Brazilian pine assembled unigenes. A cut-off threshold of 3 was applied for expectation value. Following this criterion, 54 potential targets were found (for 32 miRNA families) of which, 11 were targets of conserved miRNAs (10 miRNA families) and 43 were targets of novel miRNAs (22 miRNA families). Detailed annotation outputs are shown in Table 6. Among the conserved miRNAs with predicted targets, Aang-miR156 was the only one that exhibited two targets, SBP (Squamosa promoter binding) and a gene encoding an exocyst complex component (EXO70A1-like). The other conserved miRNAs exhibited only one predicted potential target. For instance, Aang-miR159/transcription factor GAMYB, Aang-miR395/ATP sulfurylase 2 and Aang-miR1314/transcription factor ice 1. Among the novel miRNAs only eight exhibited only one predicted target (Aang-nmiR012, Aang-nmiR019, Aang-nmiR021, Aang-nmiR022, Aang-nmiR029, Aang-nmiR031, Aang-nmiR038, and Aang-nmiR052), the others showed two or more targets, for instance, Aang-nmiR006 exhibited four potential targets. The potential target genes regulated by the conserved miRNAs seem to display physiological functions, such as ATP-sulfurylase, regulation of gene expression (transcription factors GAMYB and ice1), RNA metabolism (U5 small ribonucleoprotein) and signaling cascades (ethylene receptor) (Table 6). Interestingly, a series of novel miRNAs (17 out of 22) are predicted to target genes related to disease resistance, like nucleotide binding site leucine-rich repeats (NBS-LRR). These results suggest that the conserved miRNAs are involved in a broad range of relatively conserved physiological functions, whereas most of the novel miRNAs, possibly Araucariaceae-specific miRNAs, seem to be involved in disease resistance.

**Table 6:** Predicted putative targets of novel and conserved miRNAs in *A. angustifolia*.

**miRNA**	**Target acc**.	**Expectation**	**Inhibition**	**Annotation**
Aang-miR156	1931415	2.5	Cleavage	squamosa promoter-binding 20
	796092	3	Cleavage	exocyst complex component EXO70A1-like
Aang-miR159	977162	2.5	Cleavage	transcription factor GAMYB
Aang-miR166	1704393	2	Cleavage	homeodomain-leucine zipper transcription factor HB-3
Aang-miR167	1066318	2.5	Cleavage	zinc finger ZAT4-like
Aang-miR171	2163525	1.5	Cleavage	scarecrow 6
Aang-miR390	153786	2.5	Cleavage	ethylene receptor 2-like
Aang-miR395	968805	3	Cleavage	ATP sulfurylase 2
Aang-miR399	1917714	2.5	Cleavage	probable apyrase 6
Aang-miR529	1928068	2	Cleavage	U5 small nuclear ribonucleoprotein 40 kDa isoform X1
Aang-miR1314	1949852	2.5	Translation	transcription factor ice1
Aang-nmiR003	799146	2.5	Translation	diterpene synthase
	2042481	2.5	Cleavage	disease resistance RPP13 4
Aang-nmiR005	1950381	2.5	Translation	probable receptor kinase At1g49730
	1063530	2.5	Cleavage	G-type lectin S-receptor-like serine threonine- kinase At2g19130
Aang-nmiR006	1926825	0	Cleavage	disease resistance RGA3
	2436549	0	Cleavage	LRR and NB-ARC domain disease resistance
	1580639	1	Cleavage	NBS-LRR disease resistance
	2026350	1	Cleavage	disease resistance RPM1-like
Aang-nmiR009	225523	3	Cleavage	disease resistance RGA3
	967108	3	Cleavage	disease resistance RGA2-like isoform X1
	1681946	1.5	Cleavage	disease resistance RPM1-like
Aang-nmiR012	1601456	2	Cleavage	TMV resistance N-like
Aang-nmiR017	2007770	2.5	Cleavage	NBS-LRR
	2105514	2.5	Cleavage	disease resistance TAO1-like
	2105518	2.5	Cleavage	TMV resistance N-like
Aang-nmiR018	811798	2.5	Translation	disease resistance (TIR-NBS-LRR class)
	811797	2.5	Translation	TMV resistance N-like
	160015	2.5	Translation	disease resistance (TIR-NBS-LRR class)
Aang-nmiR019	1997048	2.5	Translation	TMV resistance N-like
Aang-nmiR021	2023865	1.5	Cleavage	disease resistance RPM1-like
Aang-nmiR022	2023865	1.5	Cleavage	disease resistance RPM1-like
Aang-nmiR025	869457	1.5	Cleavage	senescence-associated
	1863722	0	Cleavage	cytochrome P450 like TBP
Aang-nmiR027	169430	2.5	Cleavage	cyclin-T1-3-like isoform X1
	1942123	2.5	Cleavage	cyclin-T1-5
Aang-nmiR029	1580639	2.5	Translation	NBS-LRR disease resistance
Aang-nmiR031	2026350	2.5	Translation	disease resistance RPM1-like
Aang-nmiR038	1946613	1.5	Translation	disease resistance RPP13 4
Aang-nmiR039	1799491	2.5	Cleavage	disease resistance RGA1
	2509763	2.5	Cleavage	resistance family
Aang-nmiR051	2060073	0	Cleavage	probable xyloglucan endotransglucosylase hydrolase 10
	706255	1.5	Cleavage	probable xyloglucan endotransglucosylase hydrolase 32
Aang-nmiR052	984263	2.5	Translation	L-ascorbate oxidase homolog
Aang-nmiR054	1236307	3	Cleavage	L-ascorbate oxidase
	2324731	2.5	Cleavage	cell division cycle 123 homolog
Aang-nmiR059	2131961	2.5	Cleavage	target of AVRB operation1
	2174518	0	Cleavage	SUPPRESSOR OF npr1- CONSTITUTIVE 1-like
	2151817	1.5	Cleavage	TMV resistance N-like isoform X2
Aang-nmiR062	818026	0	Cleavage	TMV resistance N-like
	2126093	0	Cleavage	disease resistance TAO1-like
	1820645	0	Cleavage	Disease resistance (TIR-NBS-LRR class) family
Aang-nmiR064	1749835	2.5	Cleavage	disease resistance (TIR-NBS-LRR class)
	2031676	2.5	Cleavage	TMV resistance N-like

### Expression Profiles of Conserved and Novel miRNAs From *Araucaria angustifolia*

**Figure 5:**
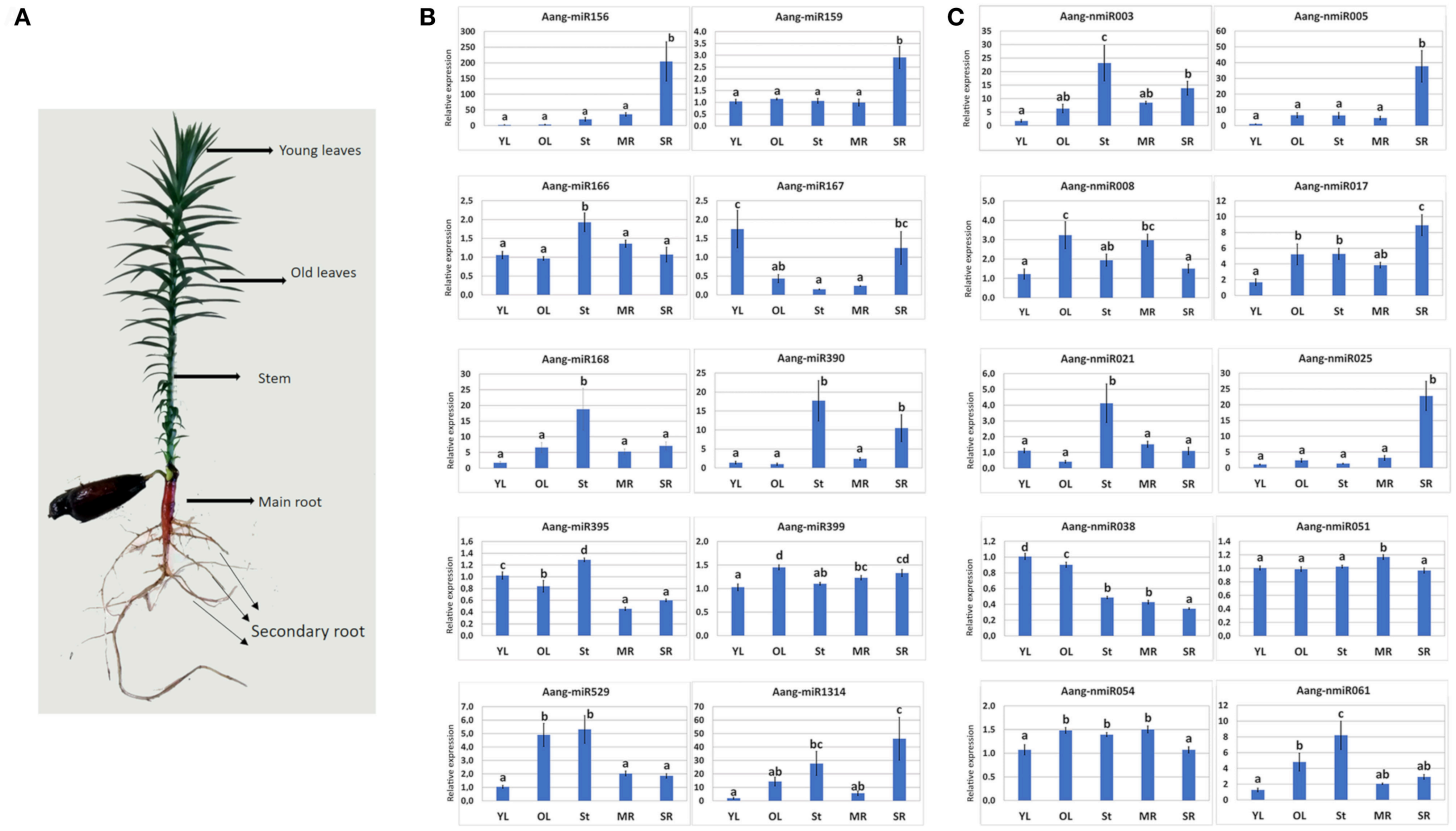
Stem loop RT-qPCR validation of known and new miRNAs in *A. angustifolia*. **(A)** A 3-month-old plant showing the tissues used for Stem-loop RT-qPCR assays. **(B)** Conserved miRNAs. **(C)** Novel miRNAs.

The stem-loop RT-qPCR method was used to validate and measure the expression of 12 conserved miRNAs (Aang-miR156, Aang-miR159, Aang-miR166, Aang-miR167, Aang-miR168, Aang-miR169, Aang-miR171, Aang-miR390, Aang-miR395, Aang-miR399, Aang-miR529, Aang-miR1314) and 30 novel miRNAs (Aang-nmiR001, Aang-nmiR002, Aang-nmiR003, Aang-nmiR004, Aang-nmiR005, Aang-nmiR007, Aang-nmiR008, Aang-nmiR009, Aang-nmiR011, Aang-nmiR012, Aang-nmiR016, Aang-nmiR017, Aang-nmiR018, Aang-nmiR019, Aang-nmiR021, Aang-nmiR023, Aang-nmiR025, Aang-nmiR026, Aang-nmiR027, Aang-nmiR029, Aang-nmiR038, Aang-nmiR040, Aang-nmiR044, Aang-nmiR046, Aang-nmiR049, Aang-nmiR051, Aang-nmiR054, Aang-nmiR057, Aang-nmiR059, Aang-nmiR061) in five tissues (young leaves, old leaves, stem, main root and secondary root) of 3-month-old plants (Figure 5A). The expression patterns of these miRNAs are illustrated in Figures 5B,C, Datas S5–S7.

Four conserved miRNAs showed high expression patterns in just one tissue compared to the others, Aang-miR166, and Aang-miR168 in the stem, and Aang-miR156 and Aang-miR159 in secondary root. The other conserved miRNAs showed different expression patterns (Figure 5B). For example, Aang-miR395 showed high levels in stem, followed by young and old leaves and low levels in main and secondary roots, Aang-miR167 showed highest expression levels in young leaves and secondary roots and Aang-miR399 showed highest levels in old leaves and secondary roots (Figure 5B).

The relative expression data of the novel miRNAs suggested highly complex expression patterns over the plant body (Figure 5C, Datas S6, S7). Among thirty novel miRNAs, twenty-seven exhibited some degree of variation in expression between the tissues (Figure 5C, Datas S6, S7). For example, Aang-nmiR038 was abundantly expressed in young and old leaves, moderately expressed in stem and main root and weakly in secondary roots (Datas S6, S7). The expression level of Aang-nmiR008 was approximately 3-fold higher in old leaves than in young leaves, 2-fold higher than secondary roots and slightly higher than stem and main root (Datas S6, S7). Three novel miRNAs, Aang-nmiR003, Aang-nmiR021, and Aang-nmiR061, had higher expression in the stem than in other tissues (Datas S6, S7). Aang-nmiR021 was barely detected in old leaves, moderately expressed in young leaves, main and secondary roots and strongly expressed in the stem with a predominant expression pattern in this tissue (Datas S6, S7). Aang-nmiR023, Aang-nmiR044, and Aang-nmiR051 showed ubiquitous expression levels in the tissues with a slight increase in main root (Datas S6, S7). Fourteen novel miRNAs had higher expression in secondary roots than in other tissues: Aang-nmiR001, Aang-nmiR002, Aang-nmiR004, Aang-nmiR005, Aang-nmiR007, Aang-nmiR011, Aang-nmiR012, Aang-nmiR016, Aang-nmiR017, Aang-nmiR018, Aang-nmiR019, Aang-nmiR025, Aang-nmiR026, and Aang-nmiR049 (Figure 5, Datas S6, S7). In some cases, the expression levels were more than 10-fold higher in secondary roots than in other tissues. For example, Aang-nmiR016 was more than 50-fold higher in secondary roots than in young leaves and Aang-nmiR025 was 20-fold higher in secondary roots than in stem (Data S6). In contrast, some novel miRNAs were homogenously expressed among the tissues, as illustrated by Ang-nmiR40 and Aang-nmiR044 (Figure 5, Datas S6, S7).

## Discussion

High-throughput sequencing technologies represent a breakthrough in the molecular biology scientific world. Thousands of genome sequences, RNA-seq, and sRNA-seq projects have been released and a plethora of biological process have been comprehensively analyzed. In the plant small RNA biology field, a series of miRNAs have been identified in a series of groups, but there is no genetic data available about miRNAs in any species (members) of Araucariaceae family*. Araucaria angustifolia* is the most important endemic conifer species in Brazil and has been used in a series of genetic studies (Auler et al., 2002; Souza et al., 2009; Elbl et al., 2015), but there is no available information about miRNAs in this species. In the present study, Illumina technology was used for deep sequencing of small RNA library to identify miRNAs in *A. angustifolia*.

The small RNA length distribution in *A. angustifolia* shows high abundance in sequences with 21 and 24 nt, (Figure 2B). Plant sRNAs are commonly reported in two principal size classes, 21 nucleotides and 24 nucleotides (Chávez Montes et al., 2014). This distribution pattern is well-documented in angiosperms (Guzman et al., 2012, 2013; Källman et al., 2013). However, non-angiosperm species comprise alternative ones (Chávez Montes et al., 2014). For example, in conifers, *P. abies* and *Pinus contorta* fail to produce significant numbers of 24-nt long small RNAs (Dolgosheina et al., 2008), whereas Chinese fir (Wan et al., 2012a) libraries were predominantly represented by this length class. The length enrichment toward 21 nt in the Brazilian pine sRNAome was also reported in other conifer-species (Dolgosheina et al., 2008; Chávez Montes et al., 2014; Zhang et al., 2015). The high abundance of 24 nt sRNAs, as well as the presence of 2.5 million sRNA, reads related to transposable elements (TEs) may reflect a myriad of sRNA types and functions, including events of transposition regulation. Liu and El-Kassaby pinpointed that a significant portion of 24 nt sRNAs may be related to TE silencing in *Picea glauca* during early developmental stages, and this expression decreases throughout the progression of phases (Liu and El-Kassaby, 2017). Also, a small RNA class called hc-siRNAs, mostly 24 nts in length, are substantially numerous in sRNA libraries of land plants (Axtell and Meyers, 2018). Usually, these sRNAs are derived from intergenic, repetitive and transposon-related genomic regions (Axtell and Meyers, 2018). Therefore, the high diversity of 24 nt sequences, as well as the high number of sequences related to TEs suggests a role of these elements in Brazilian pine biology.

**Figure 6:**
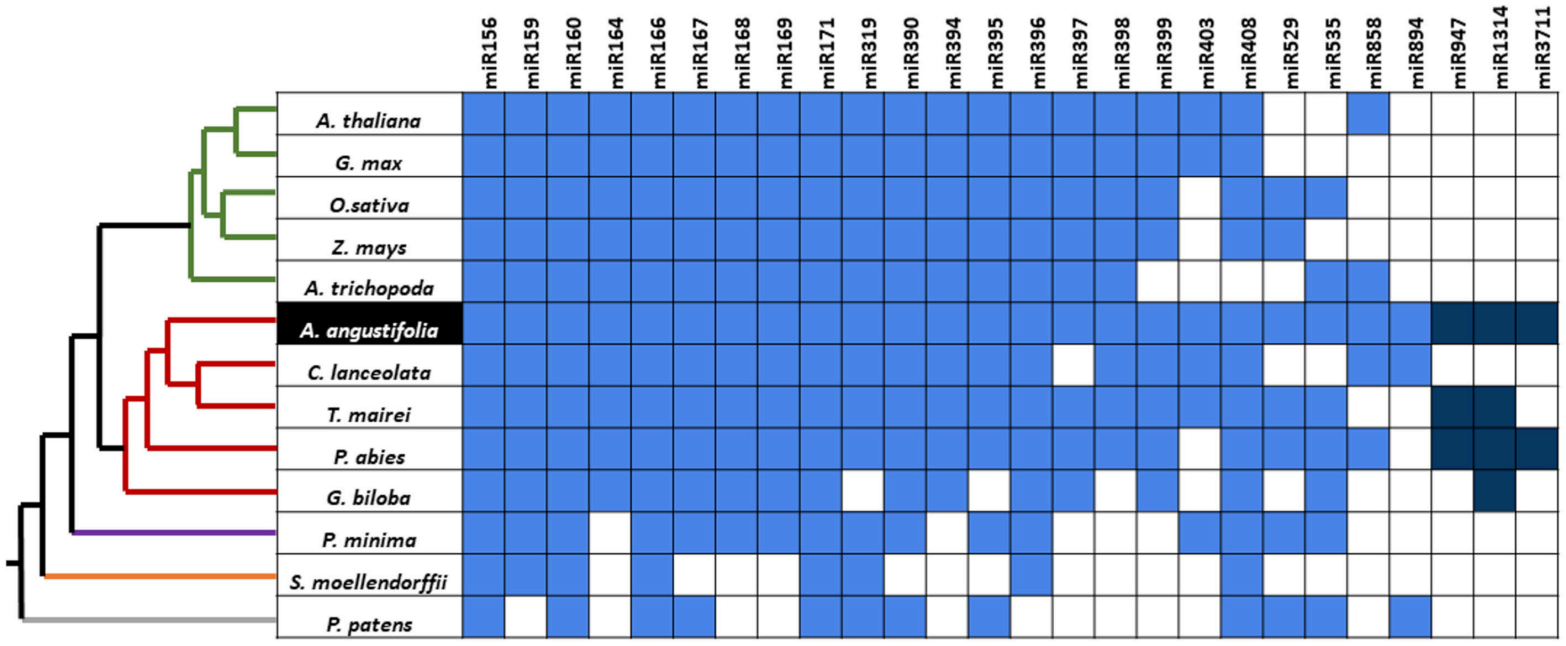
Phylogenetic distribution of conserved Brazilian pine miRNAs. The grid indicates the presence (blue squares) or absence (white squares) of miRNA families in bryophytes (gray branch), lycopods (orange branch), ferns (purple branch), gymnosperms (red branches), and angiosperms (green branches). The cladogram illustrates phylogenetic relationships among these groups and is based on Axtell and Meyers (2018). Dark blue squares indicate gymnosperm-specific miRNAs. Data from *A. thaliana, G. max, O. sativa, Z. mays, A. trichopoda, P. abies, S. moellendorffii*, and *P. patens* were obtained in miRBase release 22.0 (Kozomara and Griffiths-Jones, 2014). Conserved miRNAs from the other species were reported in the literature: *C. lanceolata* (Wan et al., 2012a), *T. maierei* (Hao et al., 2012), *G. biloba* (Zhang et al., 2015), *P. minima* (Berruezo et al., 2017).

By comparing small RNA data from *A. angustifolia* with miRBase, 115 sequences representing 30 evolutionary plant miRNA families were identified (Figure 2C). The number of family members, as well as abundancy, varied among families and sequences, respectively. Among conserved mature miRNA families identified in *A. angustifolia*, miR156, miR159, miR166, and miR167 have been documented as high abundant in several plant groups (Taylor et al., 2014). Other evolutionary conserved miRNA families were also identified in Brazilian pine. Among them, seven are conserved from Embryophytes (Berruezo et al., 2017), miR160, miR171, miR319, mir395, miR396, miR408, miR535, three are conserved from Tracheophytes (Berruezo et al., 2017), miR162, miR168, and miR403, and three conserved from Gymnosperms (Chávez Montes et al., 2014), miR947, miR1314, and miR3711 (Figure 6).

The miR403 family was reported in conifers and other vascular plant groups (Jagtap and Shivaprasad, 2014), with patterns that suggest a complex evolutionary history (Berruezo et al., 2017). For example, miRNA403 was reported in several dicots (Cuperus et al., 2011), but only in a few number of monocot species (Zhang et al., 2011). Beyond miR403, some interesting situations occur with miRNAs of other families identified in Brazilian pine. miR894 has annotations only for the moss *Physcomitrella patens* (Arazi, 2012) in miRBase. Its absence in distant species like Arabidopsis and rice suggested a species-specific character. However, outside the miRBase platform, this family has been identified in some angiosperms, like moth orchid *Phalaenopsis aphrodite* (Chao et al., 2014) and citrus (Tzarfati et al., 2013). miR6300, reported in *G. max* for the first time, has been identified in angiosperms (Biswas et al., 2016) but lack homolog sequences reported in other groups. miR5145 has annotation only for rice (Chen et al., 2011) and miR6478 has annotation only for *Populus trichocarpa* (Chen et al., 2011) in miRBase. Since mismatches were not allowed in comparisons with miRBase data, this suggests that the miRNAs above-mentioned can integrate the miRNAome of Brazilian pine, and, for the first time, were identified in a gymnosperm species.

By using a well-established approach in the prediction of pre-miRNAs (Lei and Sun, 2014), 41 conserved and 65 potential novel pre-miRNAs were identified in *A. angustifolia* (Tables S4 and S5). Taking into account the main criteria for miRNA annotation (Taylor et al., 2014; Axtell and Meyers, 2018), i.e., a stable structure of stem-loop with high base complementarity between the arms (Velayudha Vimala Kumar et al., 2017) and evidence of mature and antisense miRNA with characteristic base-paring between them (Gaspin et al., 2016), the novel and conserved *A. angustifolia* pre-miRNAs were consistently predicted (Datas S1, S2).

To exceed the miRBase blast-search in the novel and conserved miRNA identification and obtain more insights about the presence of pre-miRNAs predicted in *A. angustifolia* in different conifer species, including one of the same genera, some alternative approaches were applied. First, sRNA-seq of conifer species from different taxa, including the basal conifer *G. biloba* were mapped against pre-miRNAs predicted in *A. angustifolia*. Since mismatches were not considered, and the mapping patterns could be visualized, the output data showed that all predicted conserved pre-miRNAs were matched with small RNAs of different conifers, mainly in their sequences of 5P and 3P mature miRNAs. Interestingly, the same pattern was observed in 9 of the 65 novel pre-miRNAs predicted in *A. angustifolia* (Table 5). It is important to mention that pre-miRNAs were obtained from RNAseq data of embryonic tissues, not representing neither the juvenile phase used in the RT-qPCR analysis, nor the mature leaf tissue used to obtain the small RNA seq data corresponding to mature miRNAs. So far, the amount pre-miRNAs detected in this study is certainly underestimated. These findings are particularly important since these miRNAs were considered novel because their mature and antisense miRNAs do not have potential ortholog sequences in the current version of the miRBase platform, even with data from four conifer species. In addition, the probable presence of these nine miRNAs in *G. biloba* suggests that they may evolved in early divergence times of gymnosperms. Extending this idea, another unusual analysis was applied with a similar purpose, but this time toward a related taxon. There is no data from miRNAs in the Araucariaceae family. This family has three genera, *Agathis, Araucaria*, and *Wollemia* with a primarily Southern Hemisphere distribution, with the clear majority of species endemic to Australia, New Zealand, New Guinea, and New Caledonia and just two species, *Araucaria araucana* and *A. angustifolia* endemic to South America (Escapa and Catalano, 2013). For the first time, a sRNA-seq data was analyzed and miRNAs were identified in a species representant from Araucariaceae. To extend this analysis in this family and in the genus *Araucaria*, RNA-seq data from *A. cunninghamii*, a related species endemic from Australia, was downloaded from GenBank. The complete transcriptome was assembled into unigenes, and a BLAST search between *A. angustifolia* pre-miRNAs and *A. cunninghamii* unigenes were applied. Again, some conserved pre-miRNAs predicted in *A. angustifolia* showed sequence identity or high similarity with sequences from a different species, as could be illustrated by the identification of Aang-miR1314abcd family members in *A. cunninghamii* (Data S3). However, these analyses also indicated that seven novel pre-miRNAs identified in *A. angustifolia* showed potential orthologs in *A. cunninghamii* (Data S4). The sequence identity between these orthologs was high, reaching 96% between Aang-nmiR003 and Acun-nmiR003 (Figure 4B), and the mismatches appeared outside the mature miRNA sequences, mainly in the loop region. These patterns were already noted, but mainly in conserved miRNAs (Miskiewicz et al., 2017). For instance, Chorostecki and coworkers showed that plant miRNAs exhibit some evolutionary footprints that extend the mature miRNA sequences, comprising other conserved regions with structural determinants recognized during the biogenesis (Chorostecki et al., 2017). The putative novel orthologs in *A. angustifolia* and *A. cunninghamii* seem to follow the same pattern of evolutive fingerprints.

Several genes were predicted as potential targets for novel and conserved *A. angustifolia* miRNAs (Table 6). Among the conserved miRNA targets, functions related to energetic metabolism, signal transduction and gene expression control were found. The targets related to conserved miRNAs were reported in several taxa, miR171-scarecrow 6 (Zhu et al., 2015), miRNA395-ATP sulfurylase (Zhang et al., 2011; Jagadeeswaran et al., 2012), miR167-Zinc finger ZAT (Omidvar et al., 2015), miR166- HD-ZIPIII (Li et al., 2017b), miRNA159-GAMYB (Li et al., 2017a; Samad et al., 2017; Velayudha Vimala Kumar et al., 2017). This conservation of targets is consistent with the co-evolution of the miRNA-target pairs among plant lineages (Zhu et al., 2015).

Interestingly, a series of novel miRNAs are predicted to target NBS-LRR disease resistance genes. A strong association between the diversity NBS-LRRs and miRNAs was reported in a genome-wide study with 70 land plants (Zhang et al., 2016). There are evidence that NBS-LRR genes keep giving birth to new miRNAs targeting themselves in various plant lineages (Cui et al., 2017). Therefore, the novel miRNAs predicted in Brazilian pine seem to be more lineage-specific, given the high rate of evolution of their targets in response to the high evolution rate of pathogens.

Stem-loop RT-qPCR was applied to analyze expression patterns of conserved and novel miRNAs predicted in *A. angustifolia* (Figure 5, Datas S5–S7). Among the conserved miRNAs analyzed, Aang-nmiR166 showed highest expression levels on stem compared to the other tissues. This miRNA family has HD-ZIPIII transcription factors as potential targets, as reported in a series of studies and in the present work. Experimental analysis showed that the knock-down of miR166 promoted a substantial increase in expression levels of HD-ZIPIII genes OsHB3 and OsHB4 in stem, compared to other tissues in rice plants (Zhang J. et al., 2018). The high expression levels of this miRNA in the stem seem to be in accordance to the regulation of HD-ZIPIII transcription factors in the vascular tissues.

The expression of Aang-miR156 in secondary roots was 200-fold higher than in leaves, 10-fold higher than in stem and more than 5-fold higher than in main root (Figure 5B). These results corroborate other findings of the requirement of high levels of miR156 expression for adventitious root formation in Arabidopsis and *Malus xiaojinensis* (Xu et al., 2016, 2017). Ang-miR159 also had higher expression in secondary roots than in other tissues (Figure 6). The target prediction analysis indicated GAMYB as a potential target for this miRNA (Table 6), a conserved miRNA/target association reported in several plant groups (Xie et al., 2010; Hao et al., 2012; Wan et al., 2012a; Karlova et al., 2013). However, in contrast with the miR156 pro-adventitious root formation in Arabidopsis, miR159 was reported as a posttranscriptional repressor of root growth in the same species (Xue et al., 2017). Once Aang-miR156 and Aang-miR159 were found to be high expressed in secondary roots of 3-month-old plants, these miRNAs seem to act synergistically as positive regulators of root growth in *A. angustifolia*.

Aang-miR171 was expressed homogeneously in all tissues, which suggests that this miRNA family plays important roles in different organs in *A. angustifolia*. A series of studies reported several phenotypes in miR171-overexpressing lineages in different species (Hai et al., 2018). Interestingly, a study with transgenic Arabidopsis plants overexpressing a miR71 mature sequence from the conifer species *P. densata* showed that these mutants exhibited larger leaves, shorter primary roots, higher plant height and early flowering stages (Hai et al., 2018).

Thirty novel miRNAs predicted in *A. angustifolia* were also biologically validated via stem-loop RT-qPCR (Figure 5, Datas S6, S7). These miRNAs exhibited extremely diverse expression patterns among young leaves, old leaves, stem, main root and secondary roots (Figure 5, Datas S6, S7). The association between RT-qPCR data and target prediction rises important clues about the function of these miRNAs in *A. angustifolia*. For instance, among the novel miRNAs targeting disease-resistant genes, Aang-nmiR038 (targeting RPP13) was high expressed in young and old leaves, Aang-nmiR003 (targeting RPP13) and Aang-nmiR021 (targeting RPM1-like) were high expressed in stem, and other three novel miRNAs were higher expressed in secondary roots, Aang-nmiR017 (targeting NBS-LRR, TAO and TMV), Aang-nmiR018 (targeting NBS-LRR class), Aang-nmiR019 (targeting TMV). These patterns and associations suggest that the novel pre-miRNAs predicted in *A. angustifolia* integrate a series pathways in this species.

## Conclusion

In the present study, a small-RNA library was constructed by high-throughput sequencing of *A. angustifolia* leaves with the aim of identifying miRNA precursors, mature miRNAs and miRNA targets in this species. Also, a series of conserved and novel miRNAs predicted in *A. angustifolia* was identified in RNA-seq data from different conifers, including the Australian native congeneric species *A. cunninghamii*. This study provides the first report on the transcriptome-wide identification of miRNAs as well as the first view of the diversity, abundance and expression patterns of these small RNAs in Araucariaceae. Bioinformatics analysis suggests that Brazilian pine conserved and novel miRNAs might contribute to several physiological processes by targeting multiple targets and affecting different pathways. The novel lineage-specific miRNAs seem to be more involved with response to pathogens by targeting NBS-LRR resistance genes. Experimental analysis indicates that these miRNAs are expressed in different patterns through the plant body. This miRNA-target interaction remains to be further explored in order to achieve novel biological and evolutionary aspects in Brazilian pine and related species. It is possible that a series of miRNAs annotated in the present study can integrate the genetic pool of several non-studied conifers, including species from Araucariaceae and genus *Araucaria*. Therefore, these data represent valuable information for future genetic studies of miRNAs in Gymnosperms, by providing insights about biology, diversity, expression and evolution of these small RNAs. The upload of these data in miRBase will also be important for comparative analysis with other plant groups.

## Author Contributions

RM, JG, and FG conceived and designed the study. FG conducted *in silico* analysis. JG and ME conducted the RT-qPCR experiments. JG and RM analyzed the data. JG and RM drafted the manuscript. All authors have read and approved the manuscript.

### Conflict of Interest Statement

The authors declare that the research was conducted in the absence of any commercial or financial relationships that could be construed as a potential conflict of interest.
