# Supplementary material for: Novel and Conserved miRNAs Among Brazilian Pine and Other Gymnosperms
Source: Front Genet. 2019 Mar 22;10:222. doi: 10.3389/fgene.2019.00222 (PMC6448024; doi:10.3389/fgene.2019.00222)

**Data S1. Anchoring patterns and stem-loop structures of conserved miRNAs of *A. angustifolia*.** The sequences corresponding to the most abundant mature miRNAs in the 5p and 3p arms are labeled in red and blue, respectively. Values on the right side represent mature miRNAs and iso-miRNAs read counts.

**Aang-miR156**

```
5'->3'
TGCTGACAGAAGAGAGTGAGCACACATAGCCCAGCACATGAGACCATGCGTTTAGCCATGCGTGCTCACCATCTCTTTCTGTCAGCA
.((((((((((((((((((((((.(((.((..(((.((((....)))).)))..)).))).))))))))..)))).)))))))))).
...TGACAGAAGAGAGTGAGCAC................................................................   depth=13367, length=20
...............................................................GCTCACCATCTCTTTCTGTCAGC.   depth=1583, length=23
```

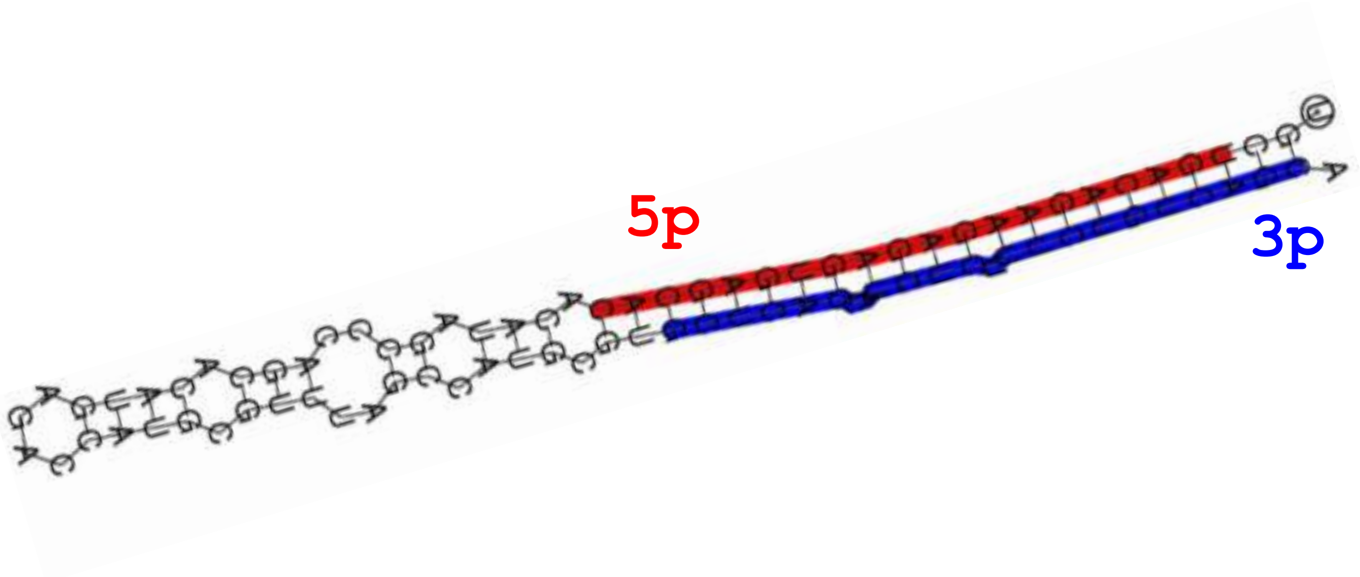

**Aang-miR159a**

```
5'->3'
GCCACAATGCATCTCTTACTTGGATTGAAGGGAGCTCCCCGAAATCCTCTTTGCATGCCTAACTGCTGAGATGGAAAATTAGTATTTCTCTCTGGAATGATATTTTTGCTATTTAC
TTTACATTGAATTTTTTAAATCTGTGTTAGGAAGTGGGAAGTGACGGGAAGCTTCCTTCAGTCCAATCGAGGGTTGTGTGGG
.((((...(((.(((((..(((((((((((((((((.((((...((..((((.(((.((((((.((.((..(((((((((..((((((.....)))))).................
.........)))))))))..)).))))))))..))).)))).)))))).)))))))))))))))))..))))).))))))).
...................TTGGATTGAAGGGAGCTCC..............................................................................
.................................................................................. depth=13078, length=19
..................CTTGGATTGAAGGGAGCTCC..............................................................................
.................................................................................. depth=77010, length=20
....................................................................................................................
................................................AAGCTTCCTTCAGTCCAATCG............. depth=3, length=21
```

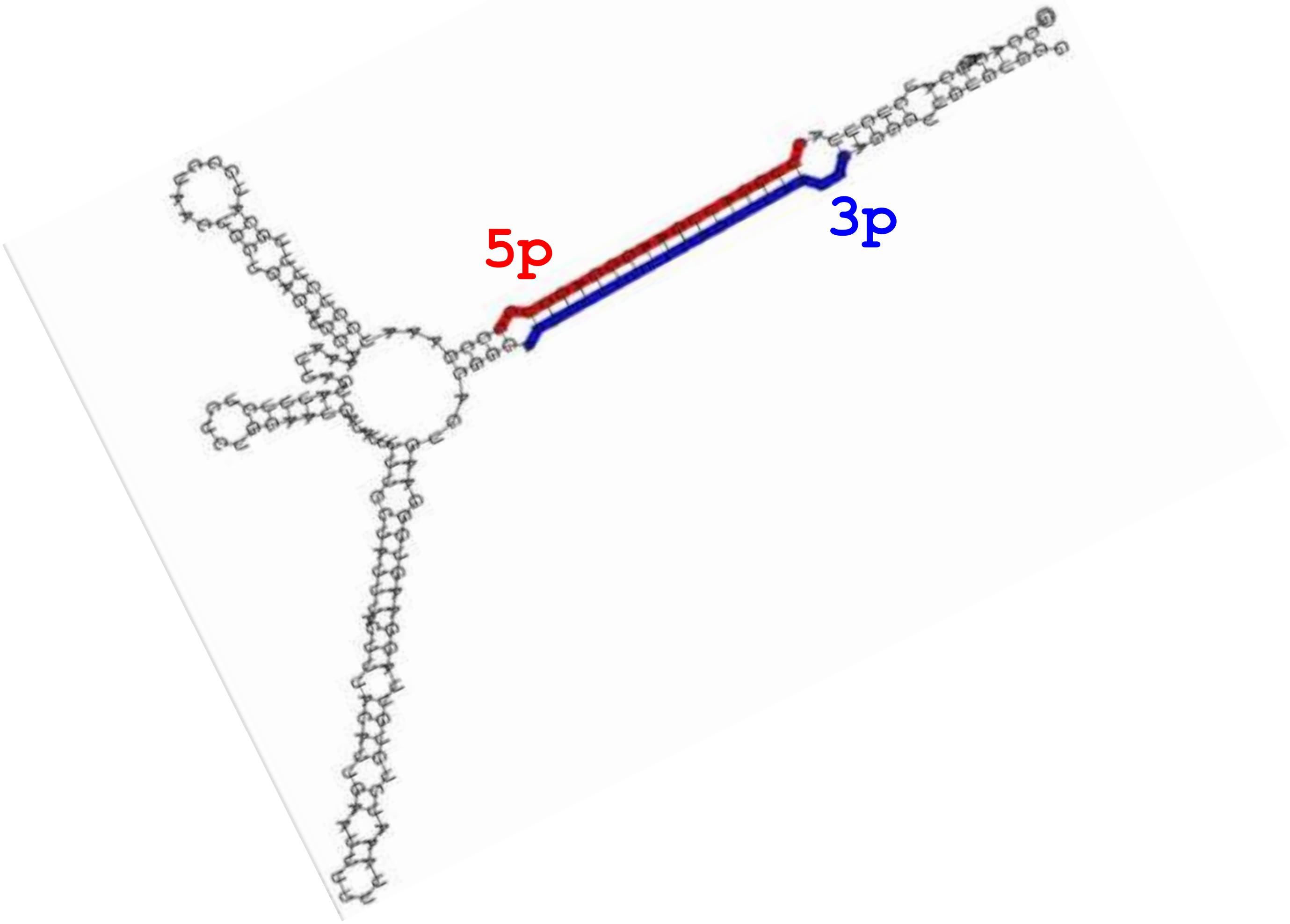

**Aang-miR159b**

```
5'->3'
GGGAGCTCCCTTCGGTCCAATTAAGGATGGTTCAGTACTGTTTCAGCTGCTGTATCATGGATTCCACTGCTCTGTCATCATGGGCTTGACATGACAGCATGGTGGTGTGCATGAGC
CGGGAGCTGTGATGTGACTGCGCTGGCCTTCCTTGGATTGAAGGGAGCTCCA
.(((((((((((((((((((..((((..(((.((((...(((.(((((.(((..(((((.((.((((((..(((((((((......)))..))))))..)))))).)).))))).. 
))).))))).)))...)))).)))..))))..))))))))))))))))))).
...AGCTCCCTTCGGTCCAATT..............................................................................................
....................................................    depth=244, length=19
...AGCTCCCTTCGGTCCAATTA.............................................................................................
....................................................    depth=35, length=20
....................................................................................................................
...............................CTTGGATTGAAGGGAGCTCC.    depth=77010, length=20
....................................................................................................................
................................TTGGATTGAAGGGAGCTCC.    depth=13078, length=19
```

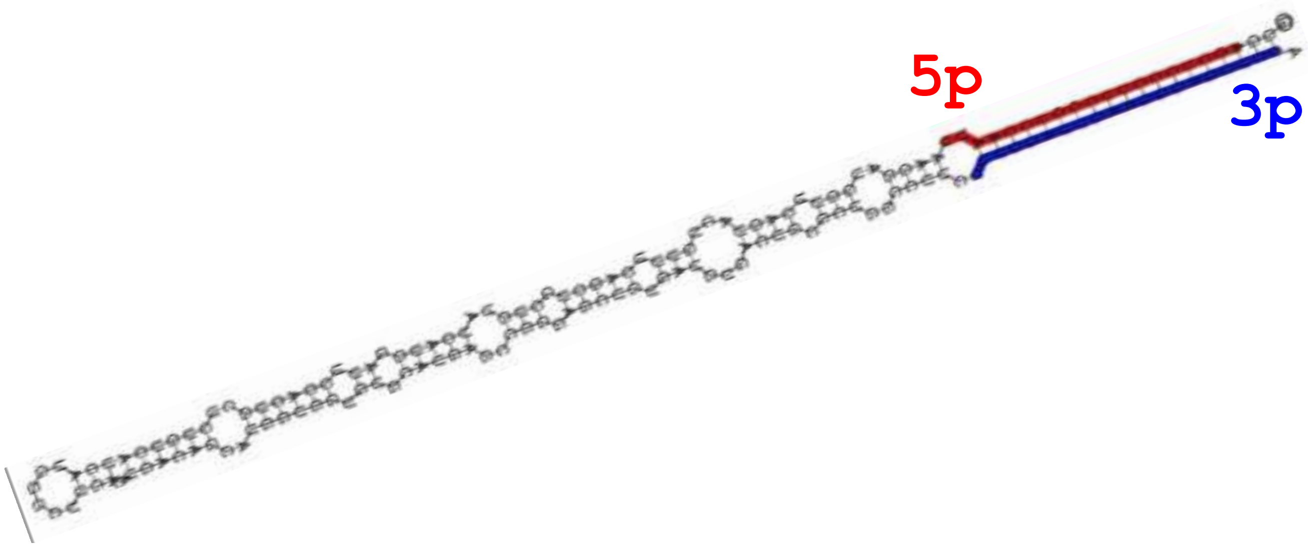

**Aang-miR160**

```
5'->3'
GCTGCCTGGCTCCCTGTATGCCATCTGCGTTGCCTATCAAAGGGGATATGGTATGCCTCGCAGTTGGCATAGAGGGAATCAAGCAGA
.((((.(((.(((((.(((((((.(((((..((.(((((.........))))).))..))))).))))))).))))).))).)))).
.CTGCCTGGCTCCCTGTATGCC.................................................................   depth=17, length=21
..TGCCTGGCTCCCTGTATGCC.................................................................   depth=8, length=20
..TGCCTGGCTCCCTGTATGCCA................................................................   depth=44, length=21
........................................................CTCGCAGTTGGCATAGAGGGA..........   depth=2, length=21
..............................................................GTTGGCATAGAGGGAATCAAG....   depth=3, length=21
..................................................................GCATAGAGGGAATCAAGCAGA   depth=1, length=21
```

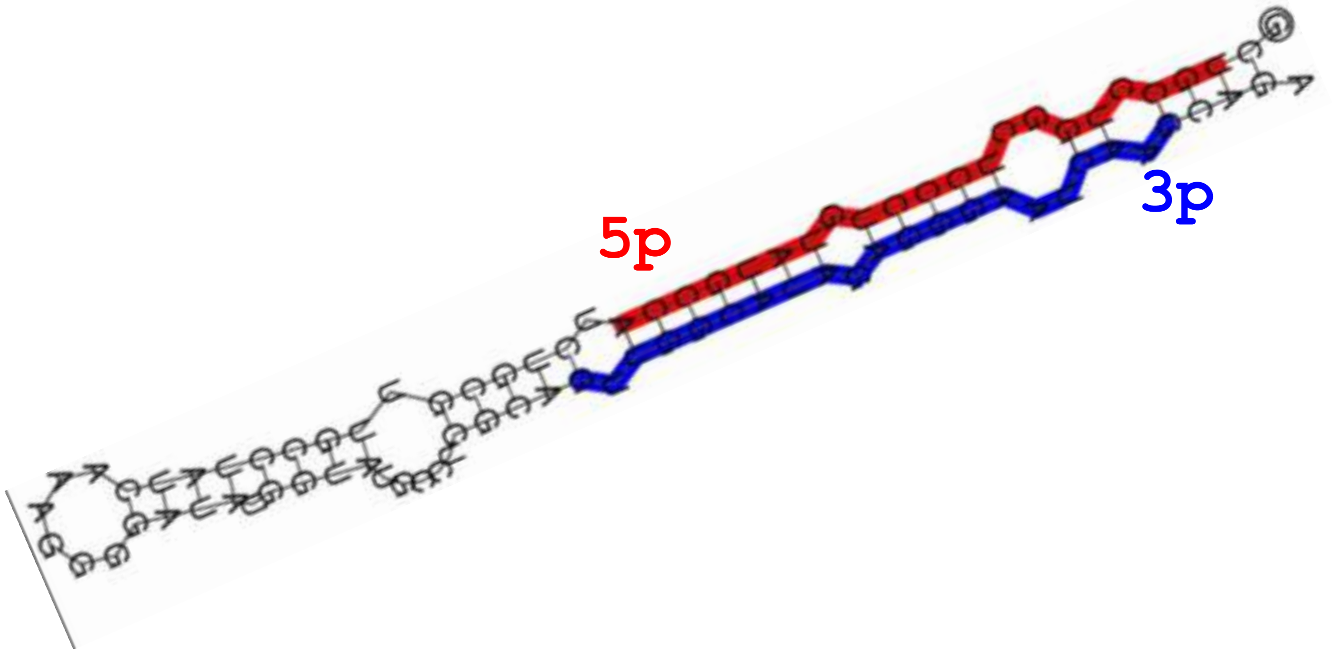

**Aang-miR166a**

```
5'->3'
AAGGGGATTGCGGTCTGGCTCGAGGTCGTCGCGGCGCCCGCGATGCCGTCGGACCAGGCTTCATTCCCCTC
.((((((.((.(((((((.((((((.(((((((((...))))))))))).)))).))))))).)).)))))).
AAGGGGATTGCGGTCTGGCT....................................................    depth=243, length=20
....GGATTGCGGTCTGGCTCGAGG...............................................    depth=172, length=21
................................................TCGGACCAGGCTTCATTCC....    depth=5305, length=19
................................................TCGGACCAGGCTTCATTCCC...    depth=2100, length=20
................................................TCGGACCAGGCTTCATTCCCC..    depth=997155, length=21
```

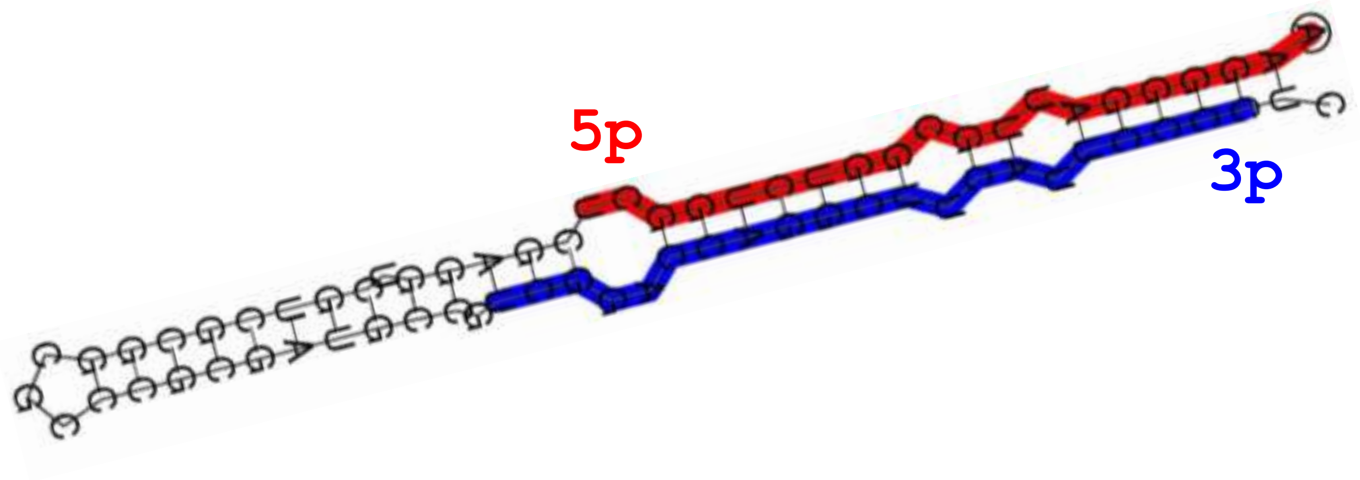

**Aang-miR166b**

```
5'->3'
AAGTTGTGGGGACTGTTGTCTGGCTCGAAGTCATTGTTGTTGGTGCAGTGATGTGTCCGAGTGACGCCGGACCAGGCTTCATTCCCCCCAACTC
.(((((.(((((.((..((((((.(((..(((((.....((((((((....)))).)))))))))..))).))))))..)).))))).))))).
.........GGACTGTTGTCTGGCTCGAAG................................................................      depth=33, length=21
..................................................................CCGGACCAGGCTTCATTCCCC.......      depth=90796, length=21
```

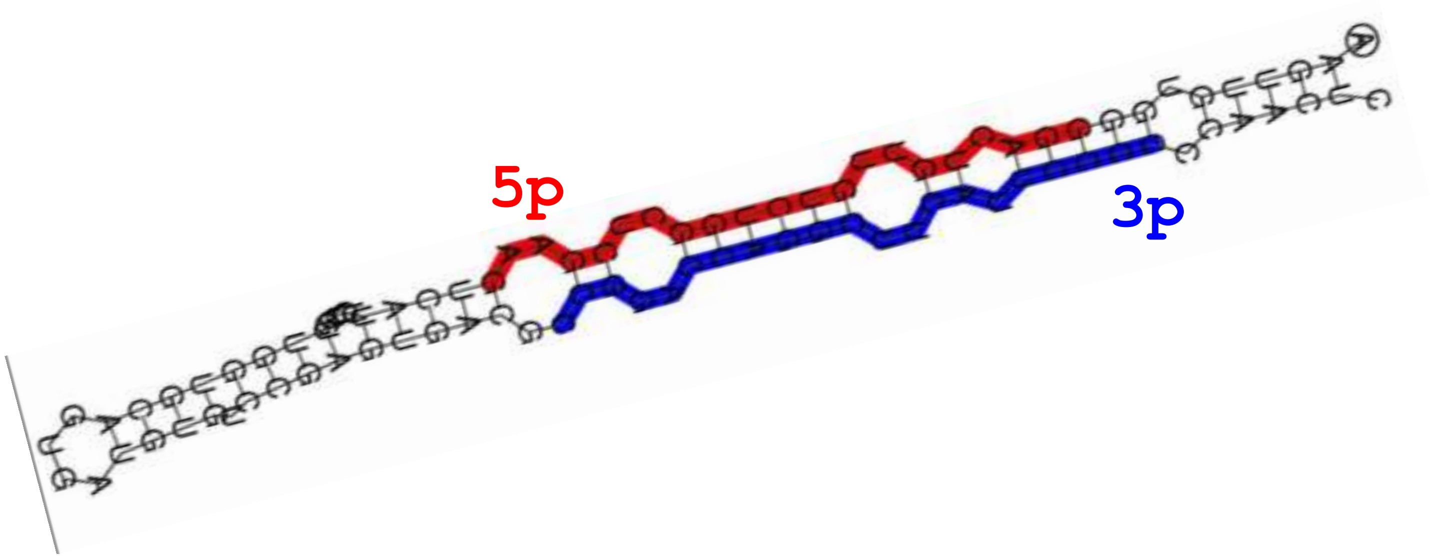

**Aang-miR166c**

```
5'->3'
TGTTTGAGGGGAATGTTGTCTGGCTCGAGGTCATCCACTCGTTTCAGATTGGAAAATACCTGGTTGACTCTATTGGGTGCCGTCGGACCAGGCTTCATTCCCCTCAAATT
.((((((((((((((..((((((.((((((.((((((...(..((((...((......))...))))..)...)))))))).)))).))))))..)))))))))))))).
.....GAGGGGAATGTTGTCTGGCTC....................................................................................depth=13, length=21
.........GGAATGTTGTCTGGCTCG...................................................................................depth=12, length=18
.........GGAATGTTGTCTGGCTCGAGG................................................................................depth=22, length=21
..................................................................................TCGGACCAGGCTTCATTCC.........depth=5305, length=19
..................................................................................TCGGACCAGGCTTCATTCCC........depth=2100, length=20
.................................................................................GTCGGACCAGGCTTCATTCCC........depth=1364, length=21
..................................................................................TCGGACCAGGCTTCATTCCCC.......depth=997155, length=21
```

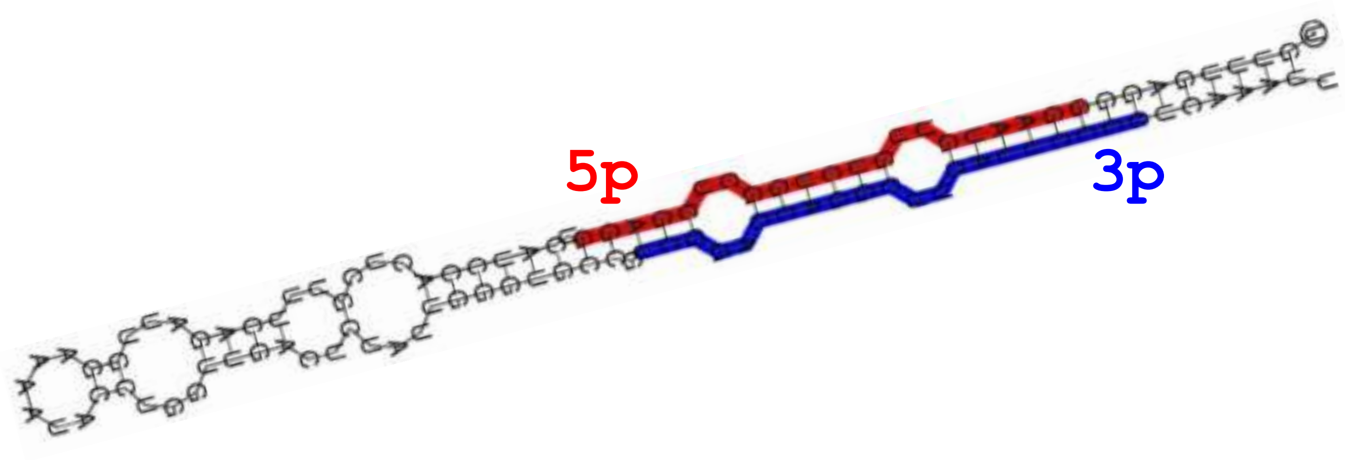

**Aang-miR166d**

```
5'->3'
TTTGAGGGGAATGTTGTCTGGCTCGACTTCGTCCAGATTTTGGCGGACGCCGTCGGACCAGGCTTCATTCCCCTCAAT
.((((((((((((..((((((.(((((..(((((..........)))))..))))).))))))..)))))))))))).
.......GGAATGTTGTCTGGCTCGAC...................................................    depth=116, length=20
.......GGAATGTTGTCTGGCTCGACT..................................................    depth=780, length=21
....................................................TCGGACCAGGCTTCATTCC.......    depth=5305, length=19
....................................................TCGGACCAGGCTTCATTCCC......    depth=2100, length=20
...................................................GTCGGACCAGGCTTCATTCCC......    depth=1364, length=21
....................................................TCGGACCAGGCTTCATTCCCC.....    depth=997155, length=21
```

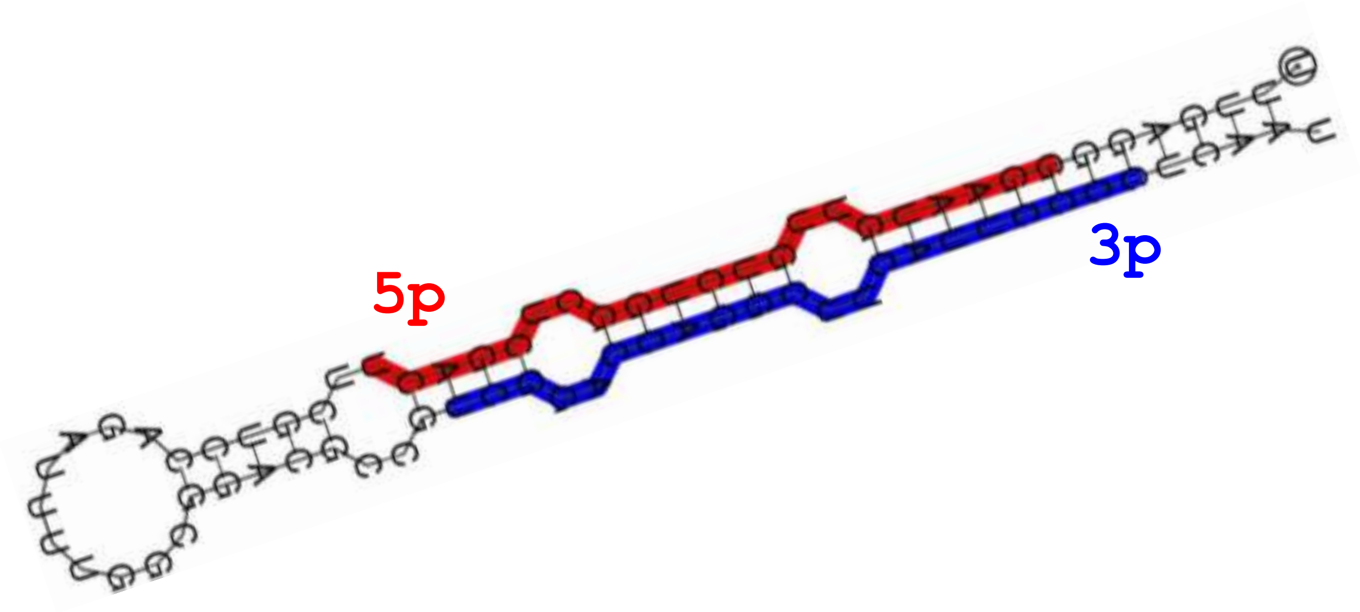

**Aang-miR166e**

```
5'->3'
GGGTGTGGGGATTGTAGTCTGGCTCGAGACCGCGCGGGATGTATTATAGTTATTTCGATGTTATGTTGGTCTCGGACCAGGCTTCATTCCCCTCAC
.(((.(((((.((.(((((((.((((((((((((((((((.........))))))).)))......)))))))).))))))).)).))))).))).
.....................................................................TCTCGGACCAGGCTTCATTCC......   depth=56326, length=21
.......................................................................TCGGACCAGGCTTCATTCC......   depth=5305, length=19
.......................................................................TCGGACCAGGCTTCATTCCC.....   depth=2100, length=20
.......................................................................TCGGACCAGGCTTCATTCCCC....   depth=997155, length=21
```

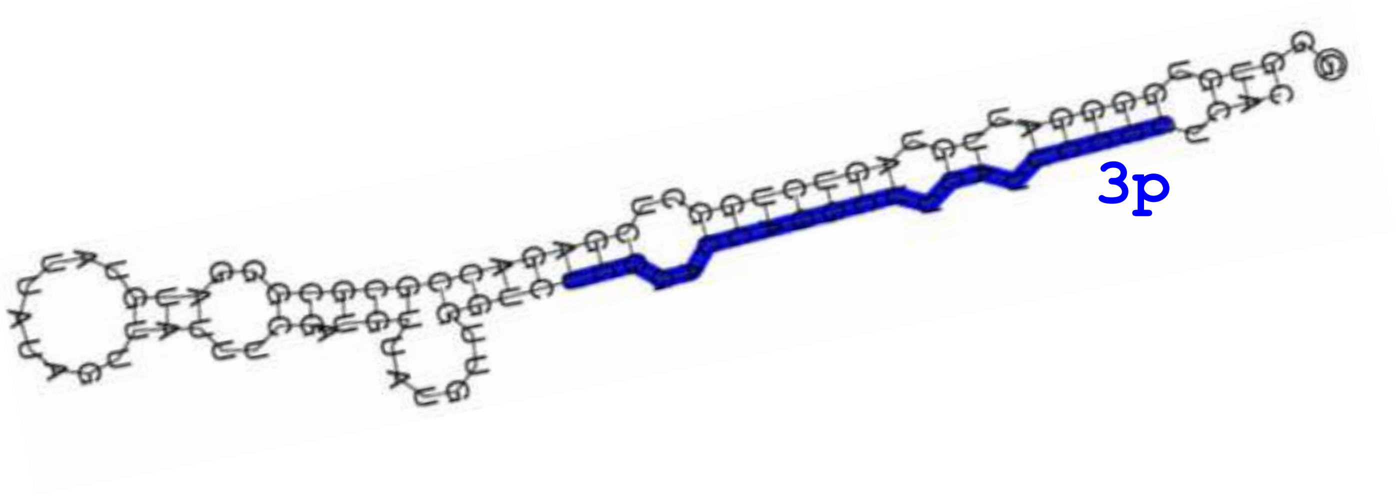

# Aang-miR167a

```
5'->3'
TGGTAAGAGAGGCTGAAGCTGCCAGCATGATCTGATGTTTTGTGTATTTTTGGATCTCATGCATAAAGCATGTGAATTCACCCATATCAGATCATCTGGTAGCTTCAGCTTCTTTTGCTG
.((((((((((((((((((((((((.((((((((((((...(((.((.......((.(((((.....))))).)))).)))..)))))))))))))))))))))))))))))))))))).
.............TGAAGCTGCCAGCATGATC........................................................................................   depth=198, length=19
.............TGAAGCTGCCAGCATGATCT.......................................................................................   depth=139, length=20
.............TGAAGCTGCCAGCATGATCTG......................................................................................   depth=1819, length=21
.............TGAAGCTGCCAGCATGATCTGA.....................................................................................   depth=11024, length=22
........................................................................................AGATCATCTGGTAGCTTCAGC...........   depth=580, length=21
```

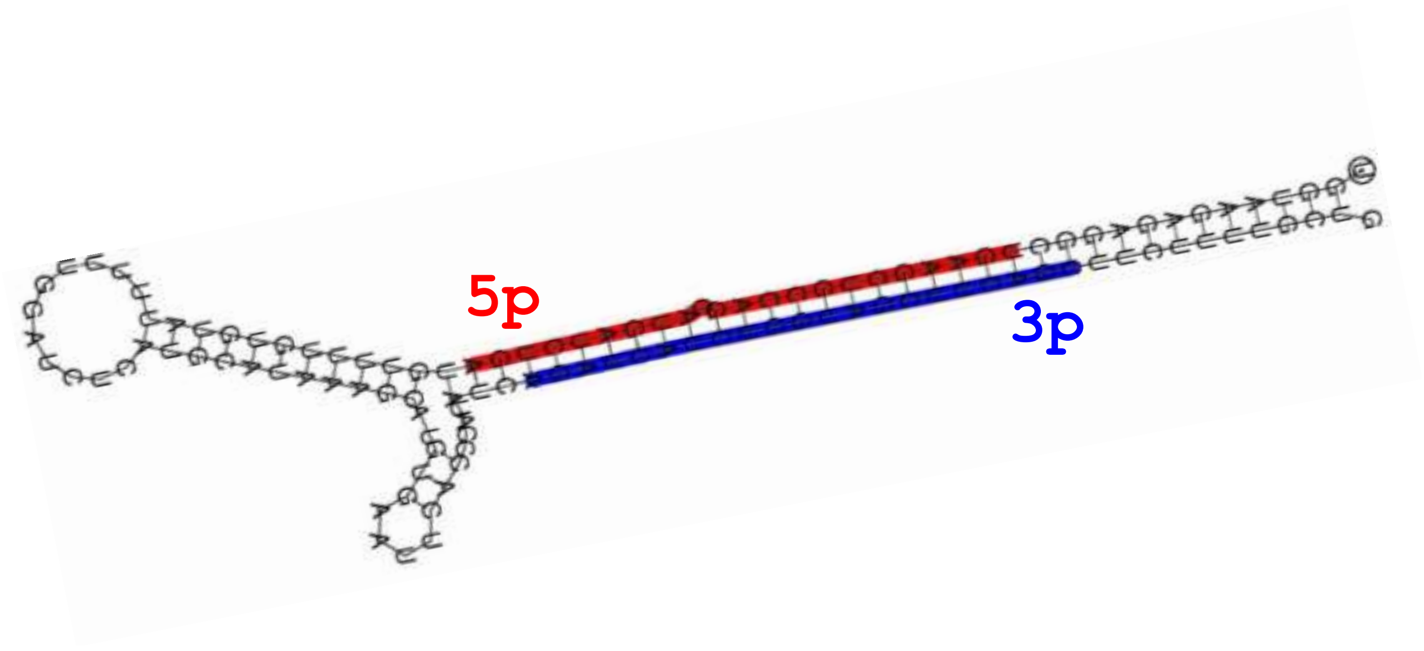

# Aang-miR167b

5'->3'

```
TGAAGGGAACAGCTGAAGCTGCCAGCATGATCTGGTGAATGTTTTATATGAGTGTTTTGTATCAGTGCATTTTATTTATTTACCAGATCATGGTGGTGGCCTCAACTTTTCCCTTCT
.((((((((.((.(((.(((((((.((((((((((((((((........((((((.(((...))).))))))....))))))))))))))))).))))))).))).)).)))))))).
.............TGAAGCTGCCAGCATGATC.................................................................................  depth=198, length=19
.............TGAAGCTGCCAGCATGATCT................................................................................  depth=139, length=20
.............TGAAGCTGCCAGCATGATCTG...............................................................................  depth=1819, length=21
.............TGAAGCTGCCAGCATGATCTGG..............................................................................  depth=2611, length=22
.................................................................................TACCAGATCATGGTGGTGGCC................  depth=2, length=21
```

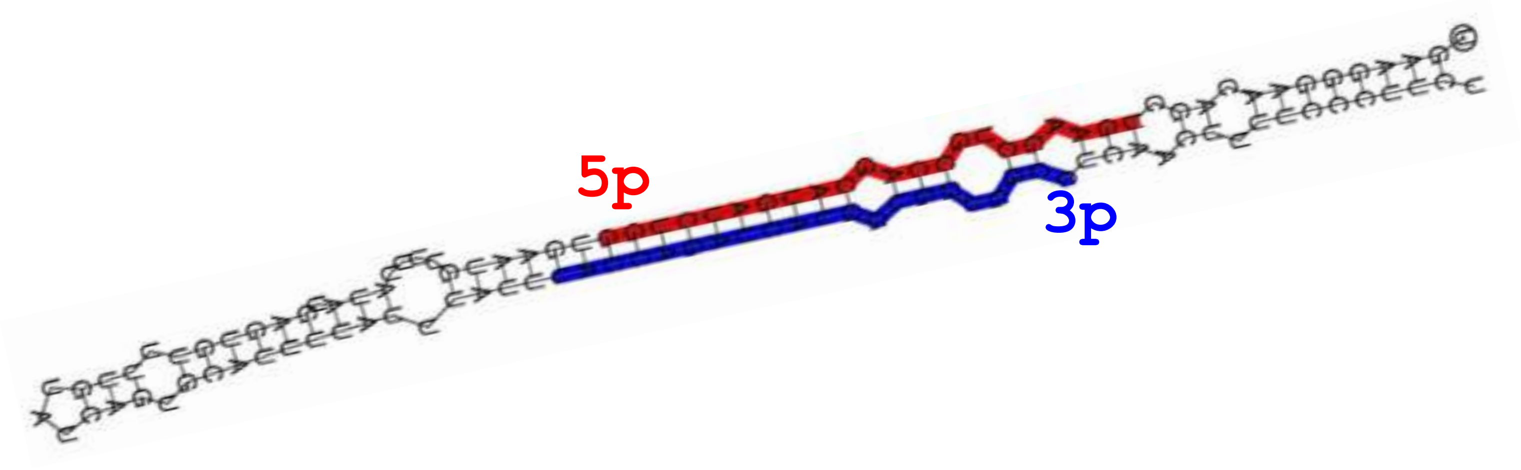

**Aang-miR167c**

```
5'->3'
AGGTTGAAGCTGCCAGCATGATCTGGGAAGGGAAGCCATGGATGCACATACGTATTCTGGATCTTGCCAGGTCATCTGGCAGTTTCACCCG
.((.((((((((((((.(((((((((.((((....(((.((((((......))))))))).)))).))))))))))))))))))))).)).
....TGAAGCTGCCAGCATGATC.................................................................... depth=198, length=19
....TGAAGCTGCCAGCATGATCT................................................................... depth=139, length=20
....TGAAGCTGCCAGCATGATCTG.................................................................. depth=1819, length=21
....TGAAGCTGCCAGCATGATCTGG................................................................. depth=2611, length=22
....................................................................AGGTCATCTGGCAGTTTCACC.. depth=6, length=21
```

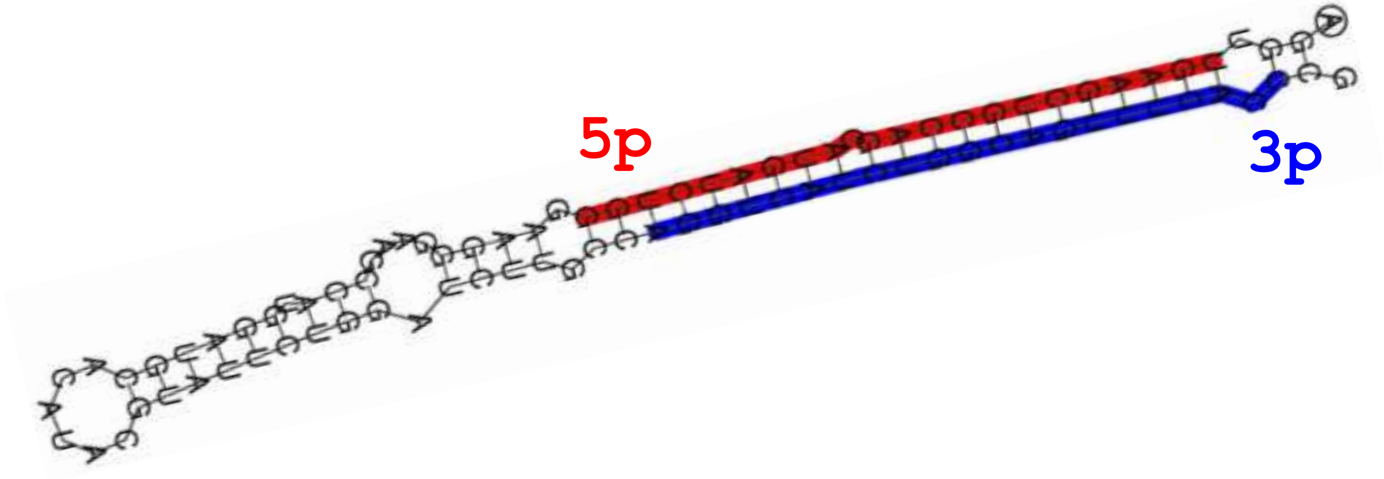

**Aang-miR167d**

```
5'->3'
AGGAGAGGTTGAAGCTGCCAGCATGATCTGGTGAATGTTTTATATGAGTGTTTTGTATCAGTGCATTTTATTTATTTACCAGATCATGGTGGTGGCCTCAACTTTTCCC
.(((((((((((.(((((((.((((((((((((((((........((((((.(((...))).))))))....)))))))))))))))).))))))).))))))))))).
.........TGAAGCTGCCAGCATGATC................................................................................. depth=198, length=19
.........TGAAGCTGCCAGCATGATCT................................................................................ depth=139, length=20
.........TGAAGCTGCCAGCATGATCTG............................................................................... depth=1819, length=21
.........TGAAGCTGCCAGCATGATCTGG.............................................................................. depth=2611, length=22
............................................................................TACCAGATCATGGTGGTGGCC............ depth=2, length=21
```

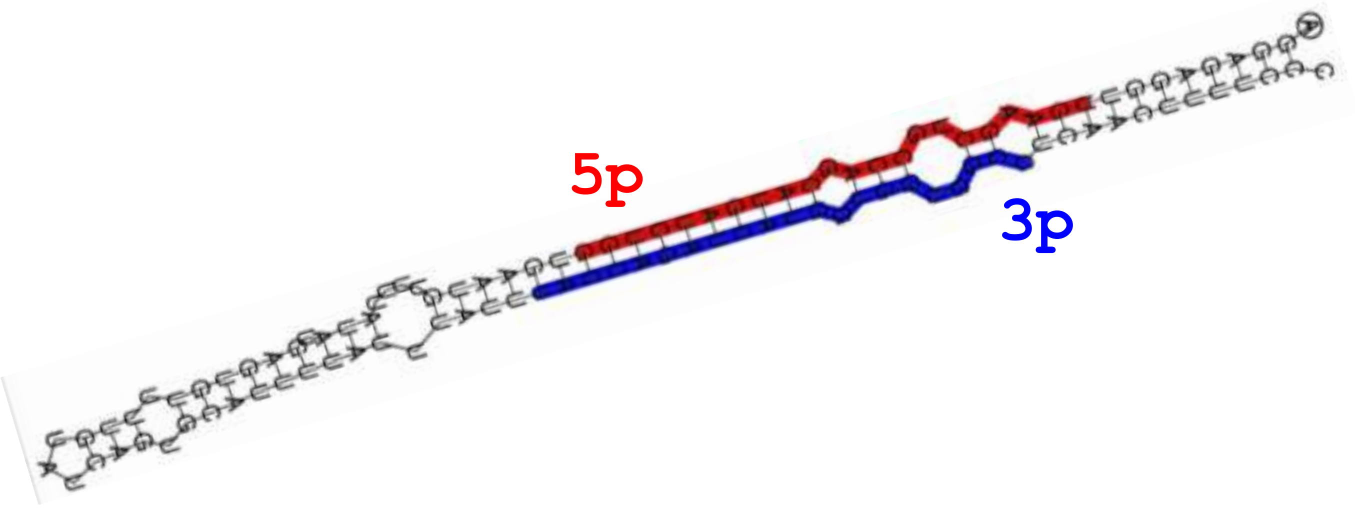

**Aang-miR168**

5'->3'
GTGGCTCGAATTCGCTTGGTGCAGGTCGGGAACGATTGTGTCCGCCCTTAGTTAATGATAATAGTAAAGCGAGCTTTTTAAAAAATTTTCAGGATCTTAATGGAGCTAAAAATAGAAGGAAACCGAGA
AGCTGTTCTGATGTGGCGGATTTGTTCCCTGCTTGCATCAACTGAATTTGAGCCAA
.(((((((((((((.(((((((((((.((((((((....((((((((((((........((((((....((..(((((((.....((((.((..(((....))).)).)))))))))))....))...
.)))))))))).).))))))))))))))).))))))))))).))))))))))))).
...........TCGCTTGGTGCAGGTCGGGAA................................................................................................
........................................................                                depth=4436, length=21
............CGCTTGGTGCAGGTCGGGAAC...............................................................................................
........................................................                                depth=1025, length=21
................................................................................................................................
.........................TCCCTGCTTGCATCAACTGA...........                                depth=117, length=20
................................................................................................................................
..........................CCCTGCTTGCATCAACTGAAT.........                                depth=332, length=21
................................................................................................................................
...............................CTTGCATCAACTGAATTTGAG....                                depth=133, length=21

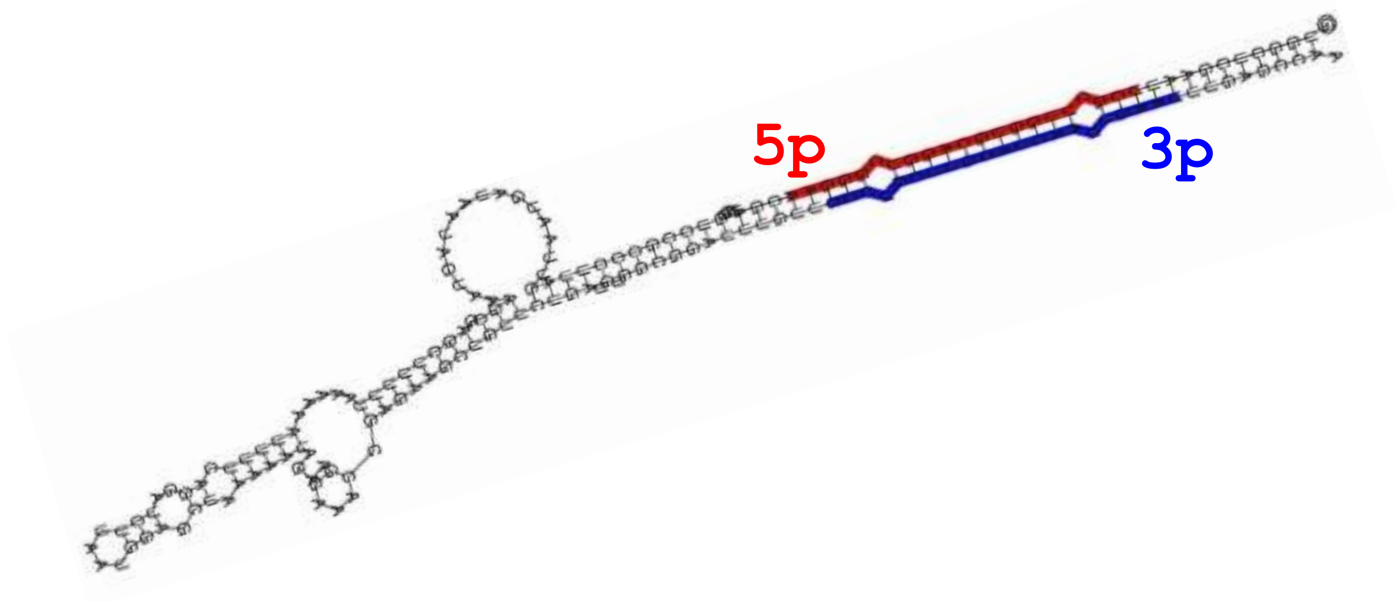

**Aang-miR169a**

5'->3'

GAGCCGAAGCAGATTGCATAGCCGAGAACAACTTGCCGGCTATTCTATTGATATATGTGGTCATATATTCAGATCACATACAGATTAGATTAGATGCCGGCAAGTTGTTCTCGGCTATGCAATCTGCTTCGGCTCT

((((((((((((((((((((((((((((((((((((((((...(((((((((.(((((((((.........)))))))))...)))))..)))).)))))))))))))))))))))))))))))))))))))))).

..........................AACAACTTGCCGGCTATTCTA.........................................................................................

depth=1, length=21

..................................................................................................GGCAAGTTGTTCTCGGCT....................

depth=71, length=18

..................................................................................................GGCAAGTTGTTCTCGGCTA...................

depth=44, length=19

...................................................................................................GCAAGTTGTTCTCGGCTATG.................

depth=58, length=20

..................................................................................................GGCAAGTTGTTCTCGGCTATG.................

depth=918, length=21

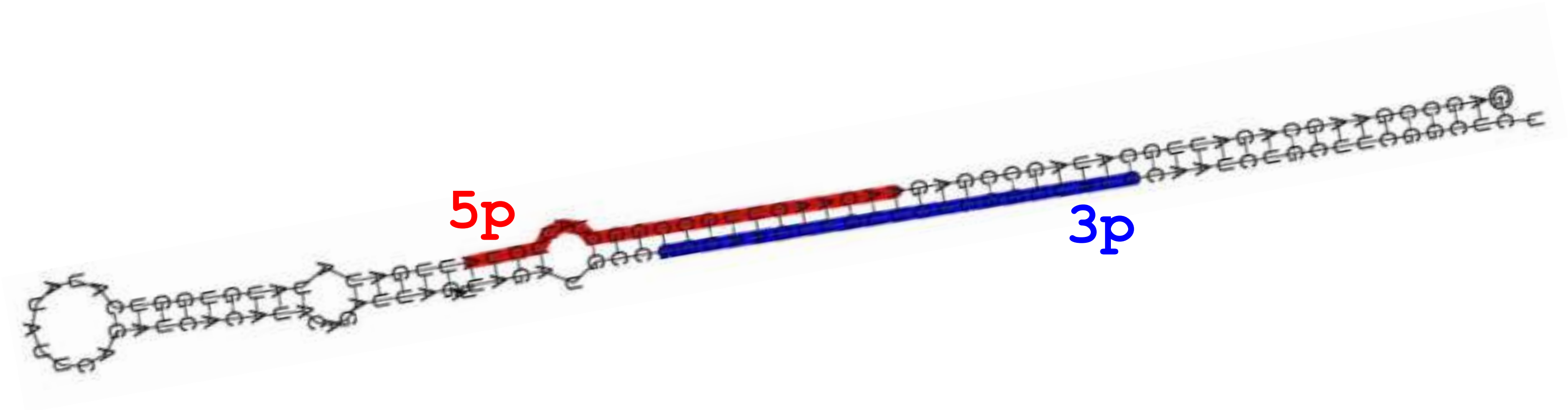

**Aang-miR169b**

5'->3'

```
TAGCAGGTAATGAAGCCAAGGATGAATTGCCGCCATCTAATCTAATCTGTAAGTTATCTGAATATATGACCACTATGAATAGACTATGAATAGAATAGCCGGCAAGTTGTTCTTGGCTACGCAATCTGCTT
.(((((((..((.((((((((((((.((((((.(.((((.((((.(((((.(((..((.........))..)))....))))).)).)).))))...).)))))).)))))))))))).))..))))))).
............AAGCCAAGGATGAATTGCCGC..................................................................................................  depth=13, length=21
..............................................................................................ATAGCCGGCAAGTTGTTCTTG................  depth=63, length=21
....................................................................................................GGCAAGTTGTTCTTGGCTACG..........  depth=72, length=21
```

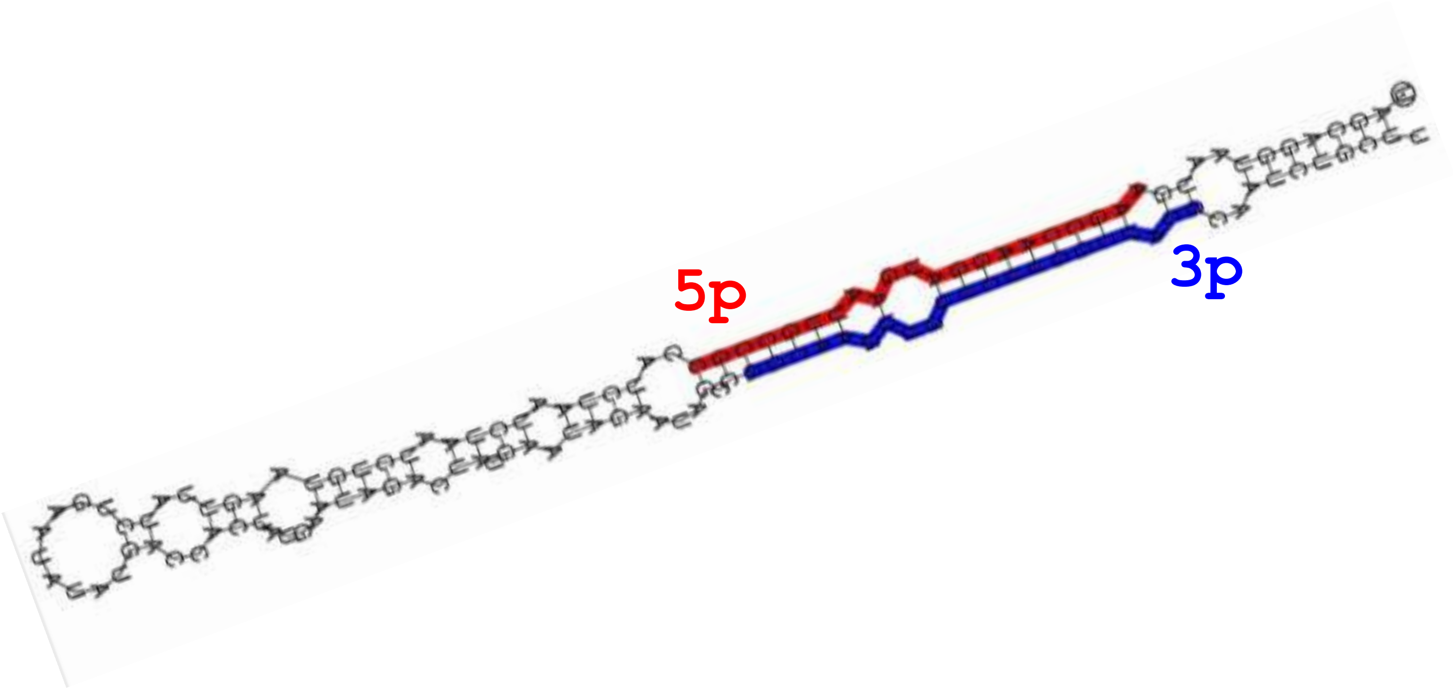

**Aang-miR169c**

```
5'->3'
TTCTGCCATGAAGGGCCATAGCAGGTAATGAAGCCAAGGATGATTTGCCGGCATCTAATCTAATCTGTAAGTGATCTGAATGGATGACCACTATGAATAGAATAGCCGGCAAGTTGTTCTTGGCTACGCAATCTGCTTGGGC
GCTCATGCCAGAC
.((((.(((((.(.(((..(((((((..((.(((((((((((((((((((((.((((.((......((..((.((((....)))).)).))...)).))))...))))))))))))))))))))).))..)))))))..)))
.)))))).)))).
..............................AAGCCAAGGATGATTTGC..............................................................................................
.............    depth=155, length=18
..............................AAGCCAAGGATGATTTGCCGG...........................................................................................
.............    depth=938, length=21
..............................AAGCCAAGGATGATTTGCCGGC..........................................................................................
.............    depth=136, length=22
.....................................................................................................ATAGCCGGCAAGTTGTTCTTG....................
.............    depth=63, length=21
...........................................................................................................GGCAAGTTGTTCTTGGCTACG..............
.............    depth=72, length=21
```

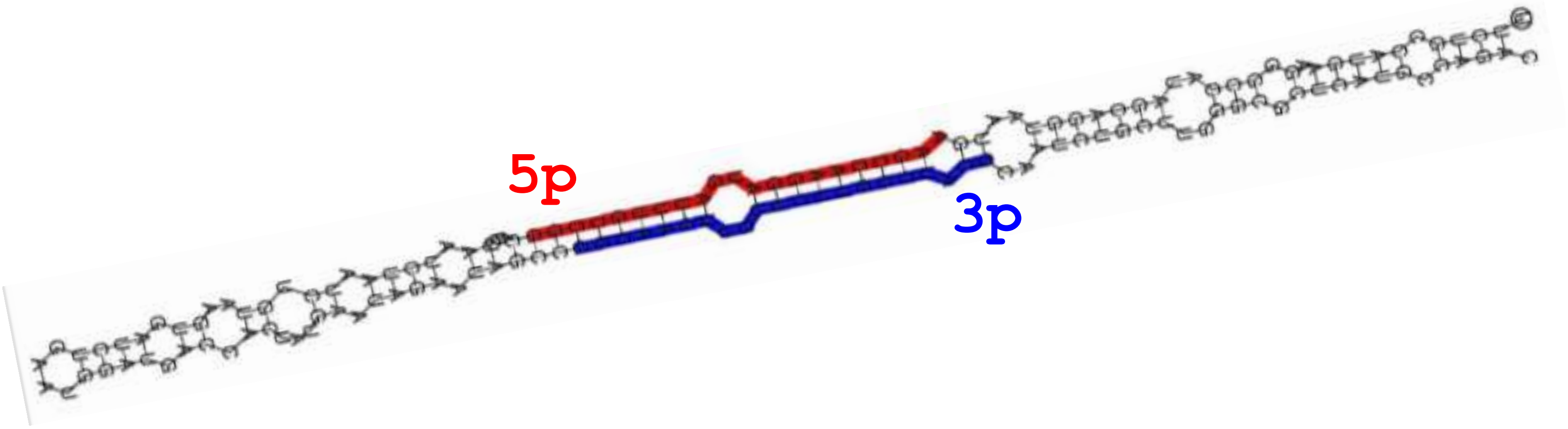

# Aang-miR169d

```
5'->3'
TTCTGCCATGAAGGGCCATAGCAGGTAATGAAGCCAAGGATGATTTGCCGGCATCTAATCTAATCTGTATGTGATCTGAATATATGACCACATATATCAATAGAATAGCCGGCAAGTTGTTCTCGGCTATGCAATCTGCTTCGGCTCTCATGCCAGAC

.((((.(((((.(((((..(((((((.....((((.((((((((((((((((...((.((((...((((((((.((.........)).))))))))....)))).)))))))))))))))))).)))).....)))))))..)))))))))).)))).
........TGAAGGGCCATAGCAGGTAATG................................................................................................................................
          depth=189, length=22
..............................AAGCCAAGGATGATTTGC..............................................................................................................
          depth=155, length=18
..............................AAGCCAAGGATGATTTGCCGG...........................................................................................................
          depth=938, length=21
..............................AAGCCAAGGATGATTTGCCGGC..........................................................................................................
          depth=136, length=22
..............................................................................................................GGCAAGTTGTTCTCGGCT..............................
          depth=71, length=18
..............................................................................................................GGCAAGTTGTTCTCGGCTATG...........................
          depth=918, length=21
```

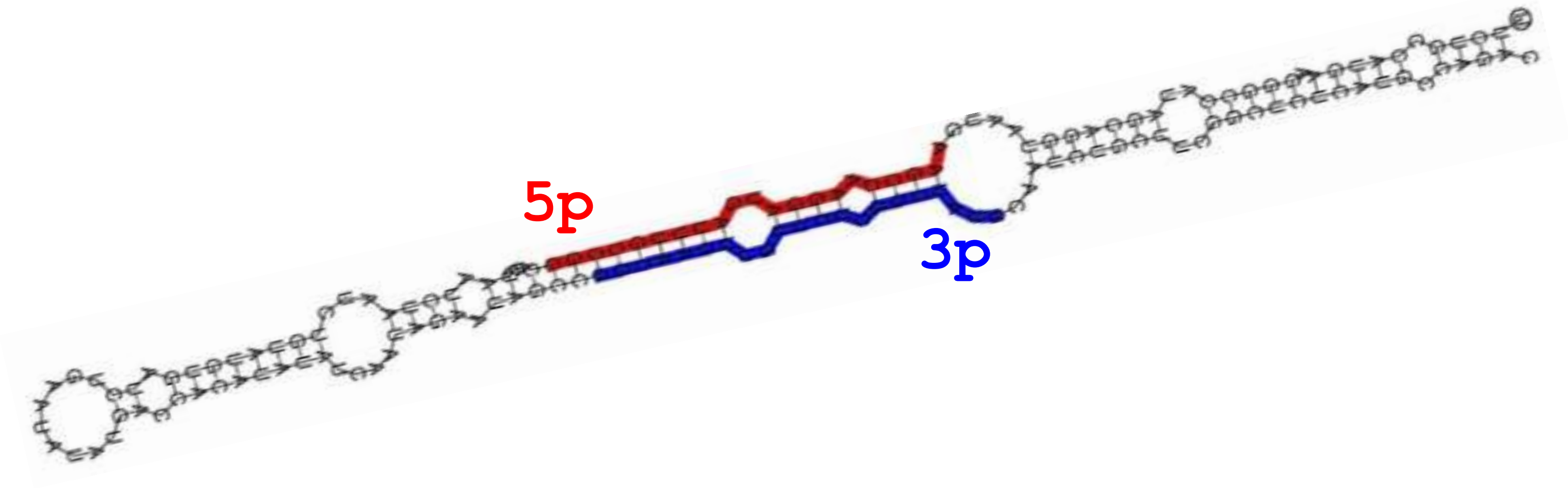

**Aang-miR171a**

```
5'->3'
TCTAGTGGGATATTGGAGCGGTTCAACCAGACAATCTCATTATAATGGGGTATAATGCTGTTGTTTGTTTGAGCCGTGCCAATATCGCACTAGC
.((((((.((((((((.((((((((.((((((((..((((((((......))))))))..)))))))))).))))))))).))))))))).)))))).
.......GGATATTGGAGCGGTTCAACC.....................................................................  depth=2, length=21
...................................................................TTGAGCCGTGCCAATATCGC......  depth=73, length=20
...................................................................TTGAGCCGTGCCAATATCGCA.....  depth=378, length=21
```

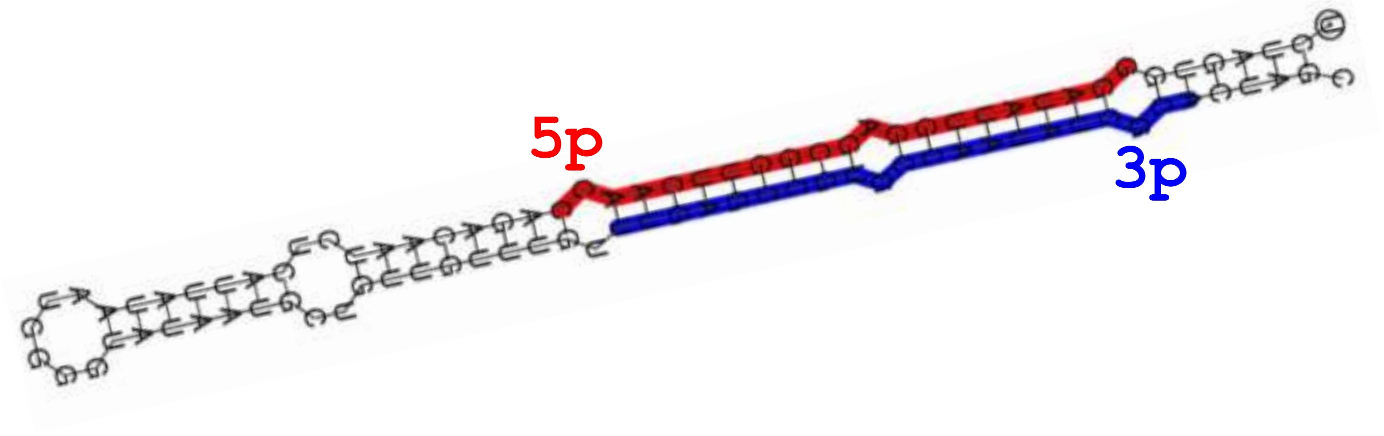

**Aang-miR171b**

```
5'->3'
TGATGGAGCTGACCGTATGTGATGTTGGCTGGGCTCAATCGGATTACGATACCAAAGAAGGGTGGTGCTCATATCTGATTGAGCCGTGCCAATATCACAATCTCAGCTTCGTCT
.((((((((((.....((((((((((((..(((((((((((((...(((((((........))))).))..)))))))))))))..))))))))))))...)))))))))).
.................TGTGATGTTGGCTGGGCTCAA............................................................................   depth=1, length=21
..................GTGATGTTGGCTGGGCTCAAT...........................................................................   depth=4, length=21
...................TGATGTTGGCTGGGCTCAATC..........................................................................   depth=2, length=21
........................................................................ATCTGATTGAGCCGTGCCAAT.....................   depth=3, length=21
..............................................................................TTGAGCCGTGCCAATATC..................   depth=9, length=18
...............................................................................TGAGCCGTGCCAATATCACAA..............   depth=16, length=21
```

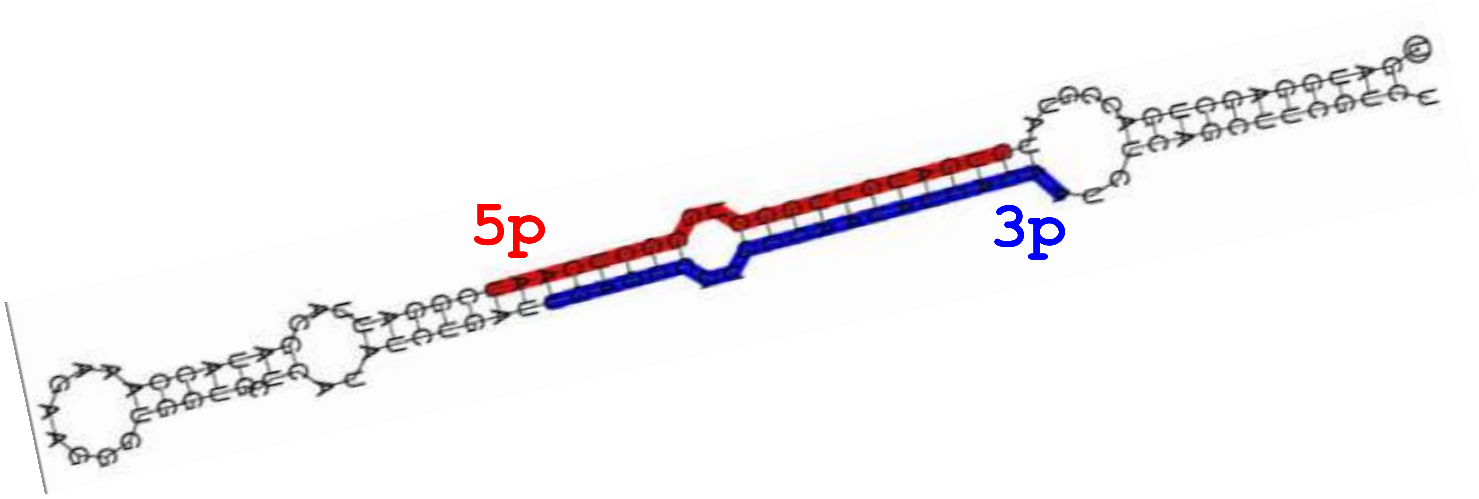

**Aang-miR171c**

```
5'->3'
ATGTGATGTTGGCTGGGCTCAATCGGATTACGATACCAAAGAAGGGTGGTGCTCATATCTGATTGAGCCGTGCCAATATCACAA
.((((((((((((..(((((((((((((...(((((((........))))).))..)))))))))))))..)))))))))))).
..GTGATGTTGGCTGGGCTCAAT.............................................................   depth=4, length=21
...TGATGTTGGCTGGGCTCAATC............................................................   depth=2, length=21
........................................................ATCTGATTGAGCCGTGCCAAT.......   depth=3, length=21
..............................................................TTGAGCCGTGCCAATATC....   depth=9, length=18
...............................................................TGAGCCGTGCCAATATCACAA   depth=16, length=21
```

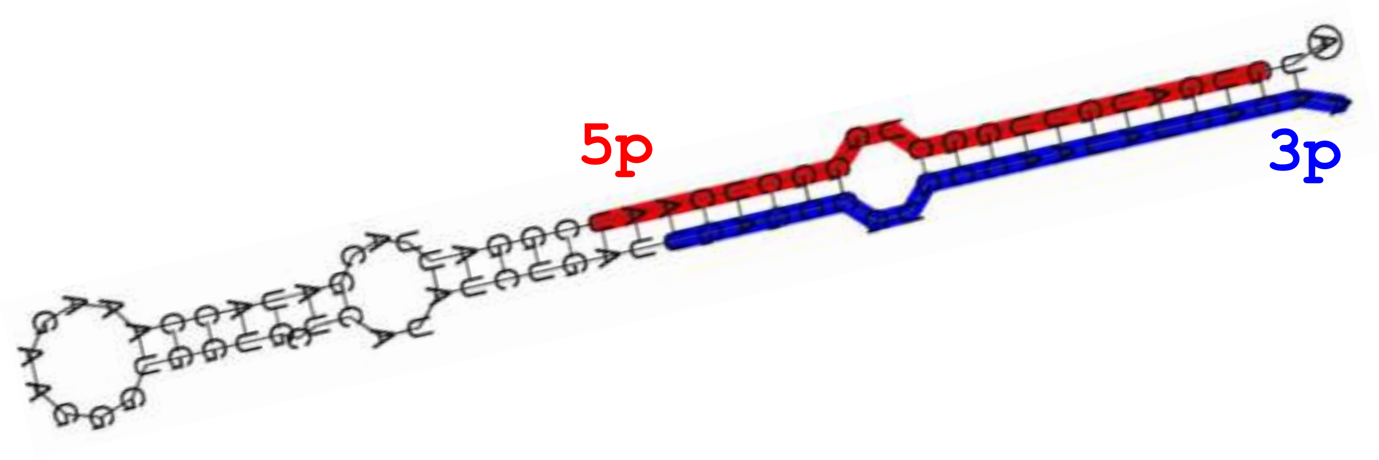

**Aang-miR390a**

```
5'->3'
CAAAGCTCAGGAGGGATAGCGCCCATGGCATTATTAGCCTGTTGTTGCAGGAAGCCAGGAGACGCTATCTATCCTGAGCTTTT
.(((((((((((.(((((((((((.((((........(((((....)))))..)))))).).)))))))).))))))))))).
..AAGCTCAGGAGGGATAGCGCC............................................................ depth=1952, length=21
..............................................................CGCTATCTATCCTGAGCTTTT depth=13, length=21
```

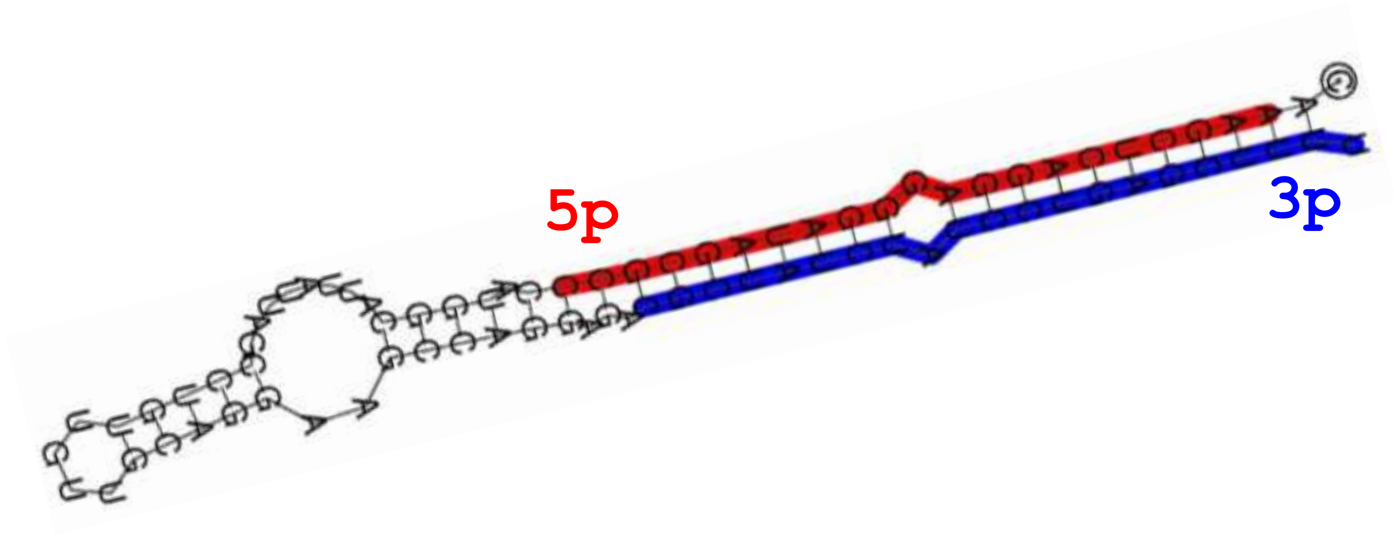

**Aang-miR394**

```
5'->3'
ATACTGGCATTCTGTCCACCTCCATATGGCGGAACACTTTTACTGTACGCGTATATGGAGGCGGACGGTATGCCAAGTAA
.(((((((((.((((((.(((((((((((((..(((.......))).))).)))))))))).)))))).))))).)))).
...CTGGCATTCTGTCCACCTCC.........................................................   depth=312, length=20
...CTGGCATTCTGTCCACCTCCA........................................................   depth=234, length=21
..........................................................AGGCGGACGGTATGCCAAGT..   depth=18, length=20
.........................................................GAGGCGGACGGTATGCCAAGT..   depth=7, length=21
```

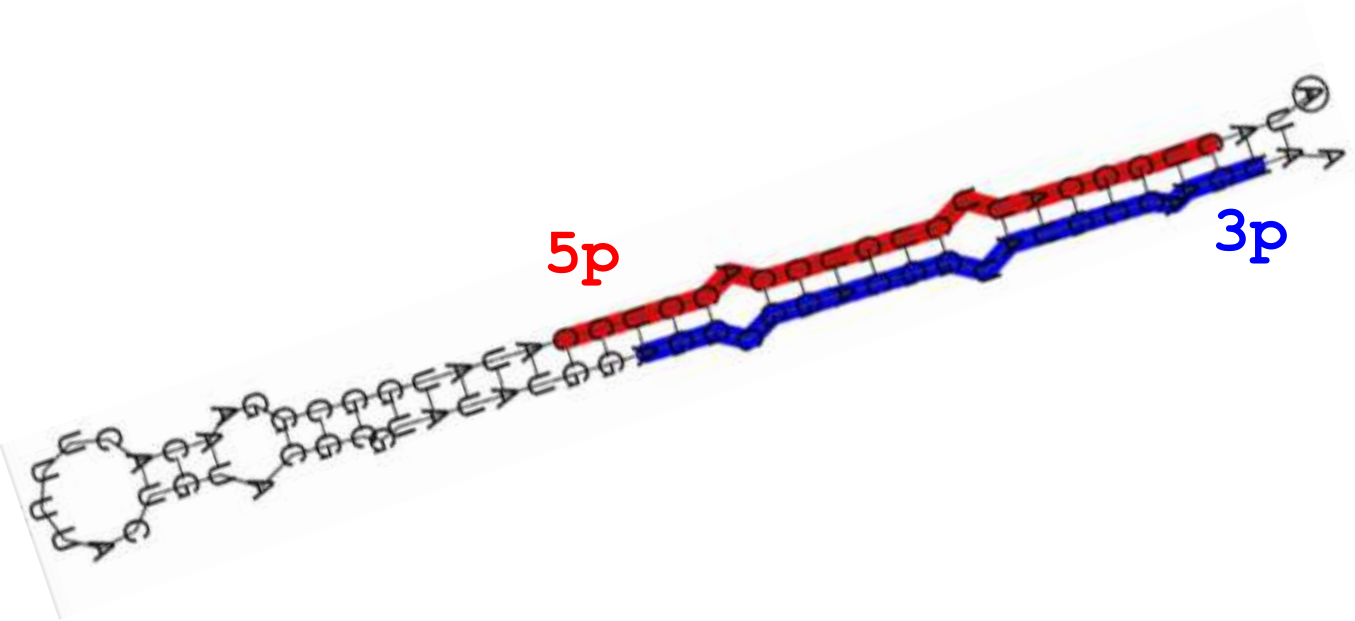

**Aang-miR395a**

```
5'->3'
GGGTGGCTAGAGTTCCCTCAACTACTTCAGAAAAACAAATAGATTTCATTCCGTTACATTTGATGGGAAACGAATTGATTTGCTGAAGAGTTTGGGGGAACTCTGGCGATCA
.(((.((((((((((((((((((.((((((.....(((((.(((((..((((((((....))))))))...))))).))))))))))))).)))))))))))))))).))).
...........GTTCCCTCAACTACTTCAGA.................................................................................  depth=32, length=20
...........GTTCCCTCAACTACTTCAGAA................................................................................  depth=157, length=21
..................................................................................CTGAAGAGTTTGGGGGAACT..........  depth=289, length=20
..................................................................................CTGAAGAGTTTGGGGGAACTC.........  depth=2157, length=21
```

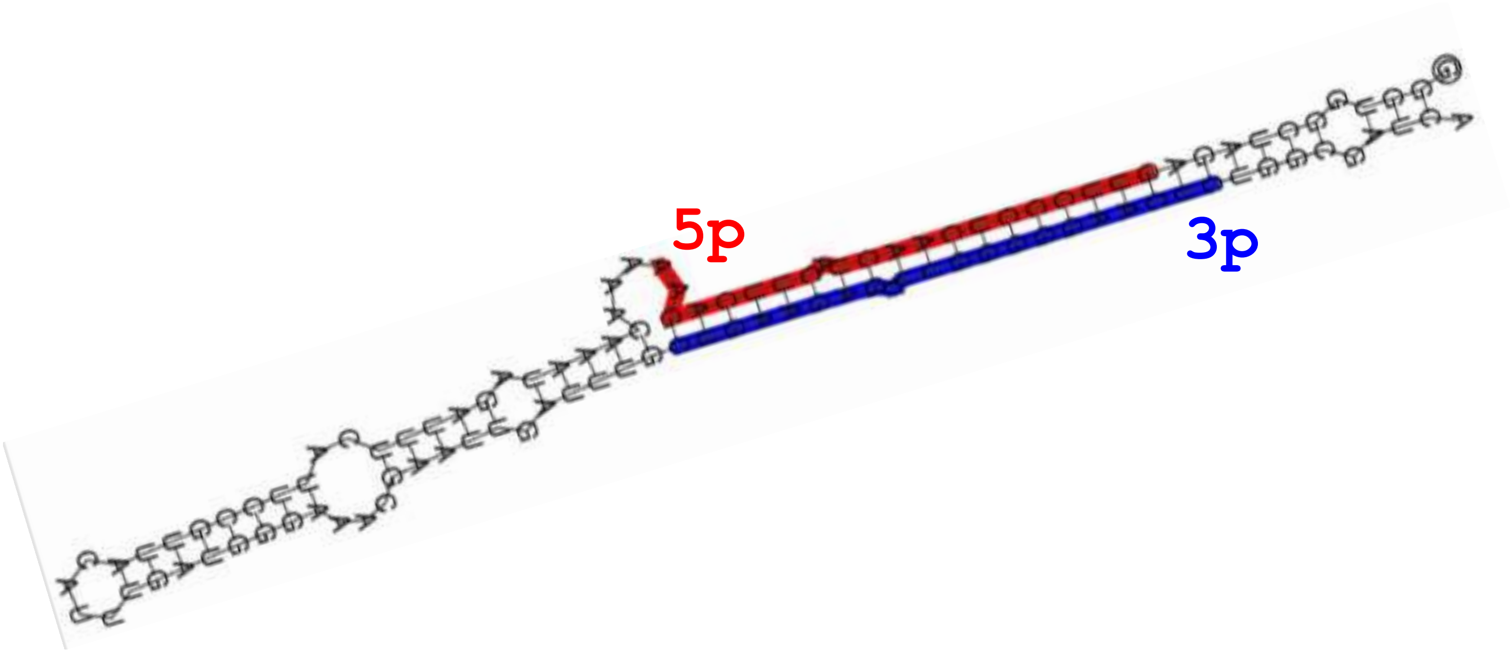

**Aang-miR395b**

```
5'->3'
TGGGTGGCTACAGTTCTCCCAACTACTTCAGGAAAACGAATACATTTTATTCTGTTTCATTTCATGCTGAAGAGTTTGGGGGAACTCTGGCGATC
.(((.((((.(((((((((((((.((((((.((((((((((.....))))).)))).......).)))))))).))))))))))).)))).))).
..................................................................CTGAAGAGTTTGGGGGAACT.........    depth=289, length=20
..................................................................CTGAAGAGTTTGGGGGAACTC........    depth=2157, length=21
```

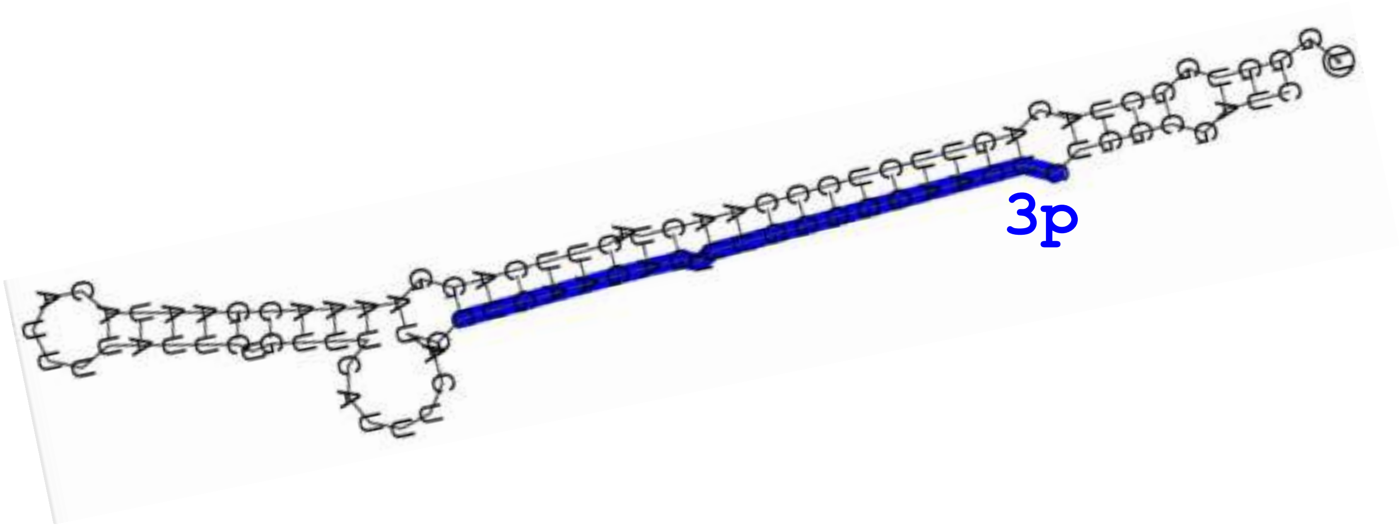

**Aang-miR396**

```
5'->3'
TCATGCTTTTCCACAGCTTTCTTGAACTTTAGTTGCGTAGGAGAAGATGGTTTGGCGTTTGGCGGATGCCTACATGCCTGTGCTTGCTTAAAGTTCAAGATTGCTGTGGGAAAACATGGA
(((((.(((((((((((..((((((((((((((.(((((((.......(((((.((.....)).)).))).......))))))))..)).))))))))))))..))))))))))).))))).
........TTCCACAGCTTTCTTGAACT............................................................................................    depth=364, length=20
........TTCCACAGCTTTCTTGAACTT...........................................................................................    depth=4606, length=21
.............................................................................................TTCAAGATTGCTGTGGGAAA.......    depth=1, length=20
..............................................................................................TCAAGATTGCTGTGGGAAAACA....    depth=1, length=22
```

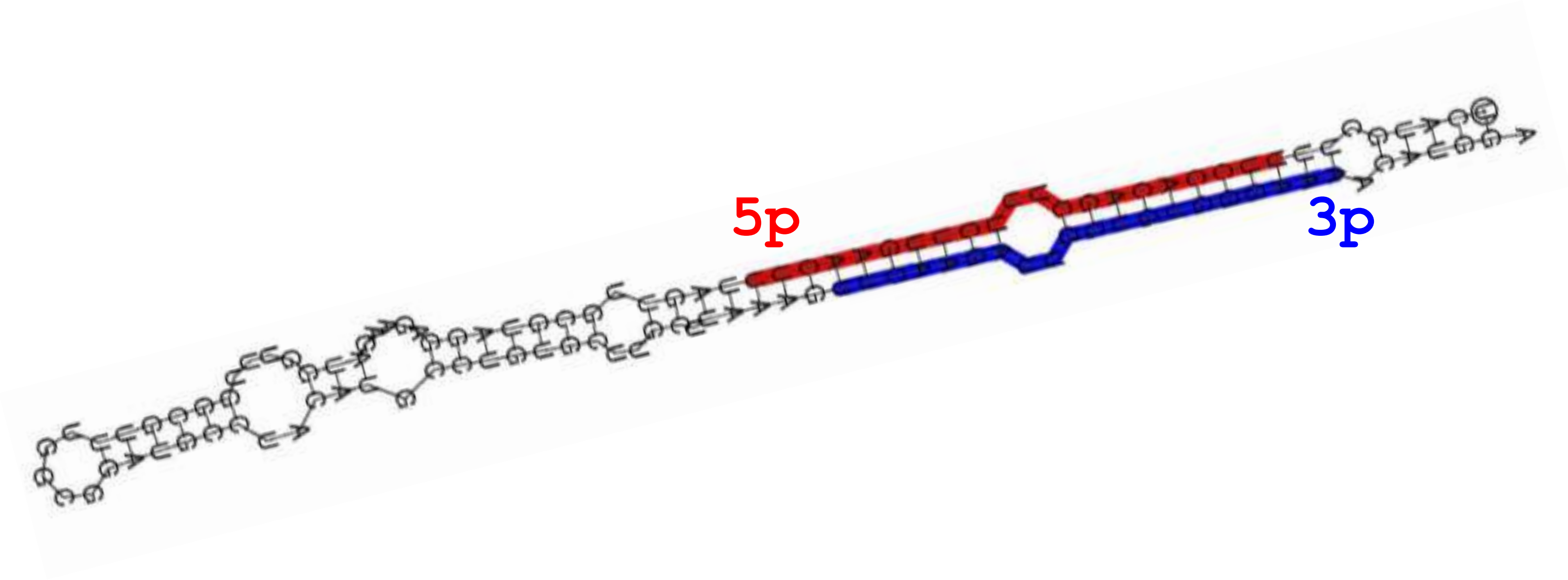

**Aang-miR399a**

```
5'->3'
CAGGATAATTGCAGGGGAGCTCTCCTTTGGCGGGTGAGACTGATACATGCTTAATGCCATTACTTTGGCTTTCTCTACTGCCTGCCAAAGGAGAGTTGCCCTGTGATTGTCCTT
.(((((((((((((((.((((((((((((((((((((((................((((......))))..))))....)))))))))))))))))).))))))))))))))).
.............GGGGAGCTCTCCTTTGGC...................................................................................   depth=3, length=18
.............GGGGAGCTCTCCTTTGGCGG.................................................................................   depth=6, length=20
..................................................................................TGCCAAAGGAGAGTTGCCCTG...........   depth=120, length=21
```

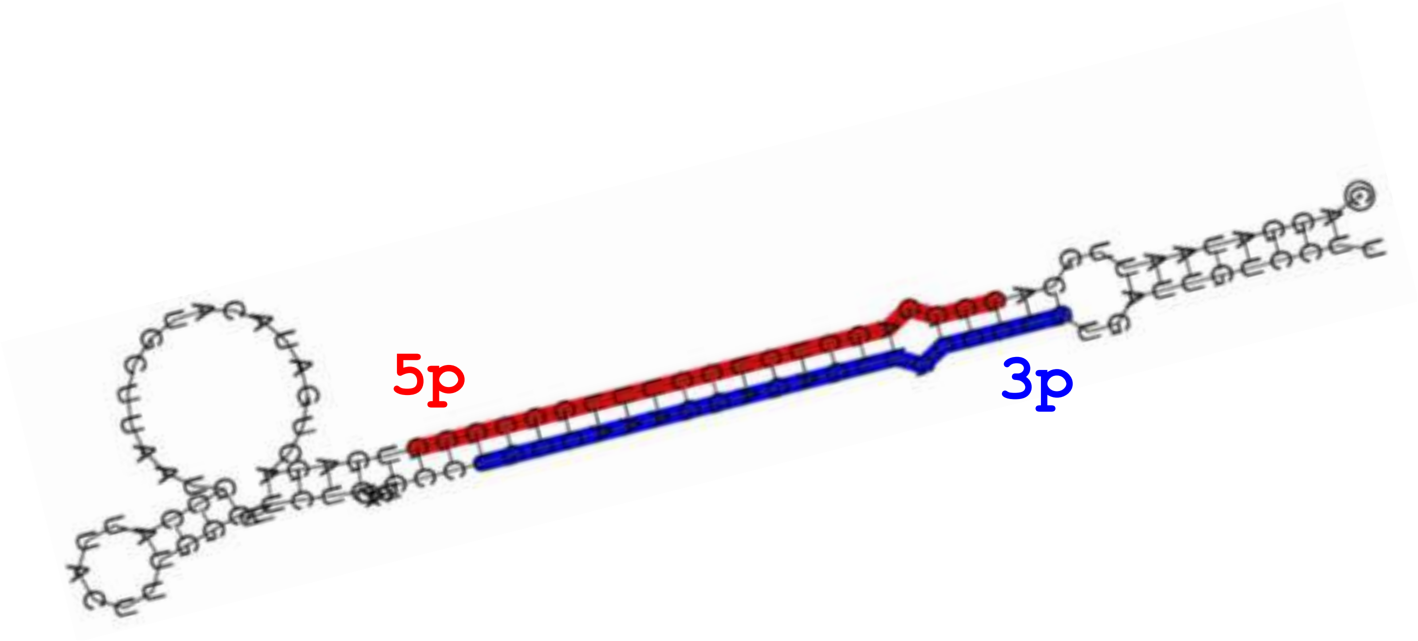

**Aang-miR399b**

5'->3'
CAGGATAATTGCAGGGGGGCTCTCCTTTGGTGGGTGAGATTGCTACATGCATAATGCTTTCTCAACTGCCTGCCAAAGGAGAGTTGCCCTGTGATTGTCCTT
.(((((((((((((((.((((((((((((((((((((((..((............))..))))....)))))))))))))))))).))))))))))))))).
.............GGGGGGCTCTCCTTTGGTGGG.................................................................... depth=2, length=21
......................................................................TGCCAAAGGAGAGTTGCCCTG........... depth=120, length=21

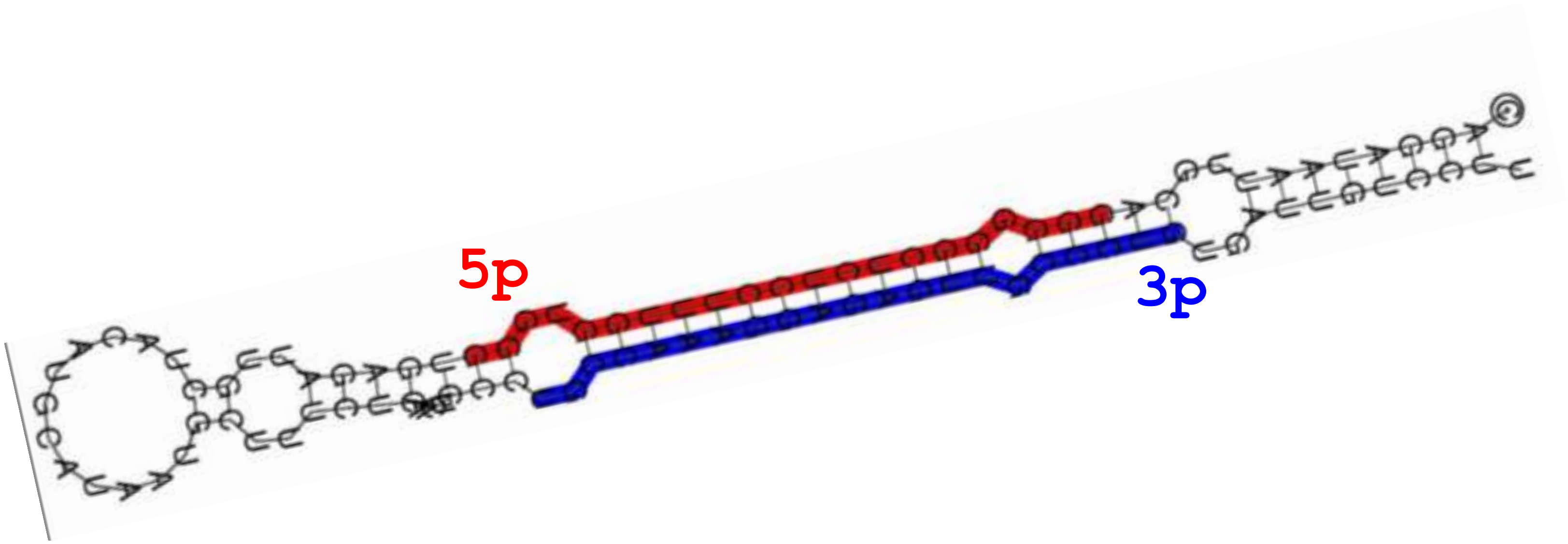

**Aang-miR399c**

5'->3'
AGGATAATTGCAGGGGAGCTCTCCTTTGGCAGGTGAGATTGATACATGCCTAATGCTATTCCTTTGGCTTCTCTATTGCCTGCCAAAGGAGAGTTGCCCTGTGATTGTTCC
.((((((((((((((.((((((((((((((((((((....((.....(((...............)))...))..)))))))))))))))))))).)))))))))))))).
............GGGGAGCTCTCCTTTGGCAGG.............................................................................. depth=2, length=21
................................................................................TGCCAAAGGAGAGTTGCCCTG.......... depth=120, length=21

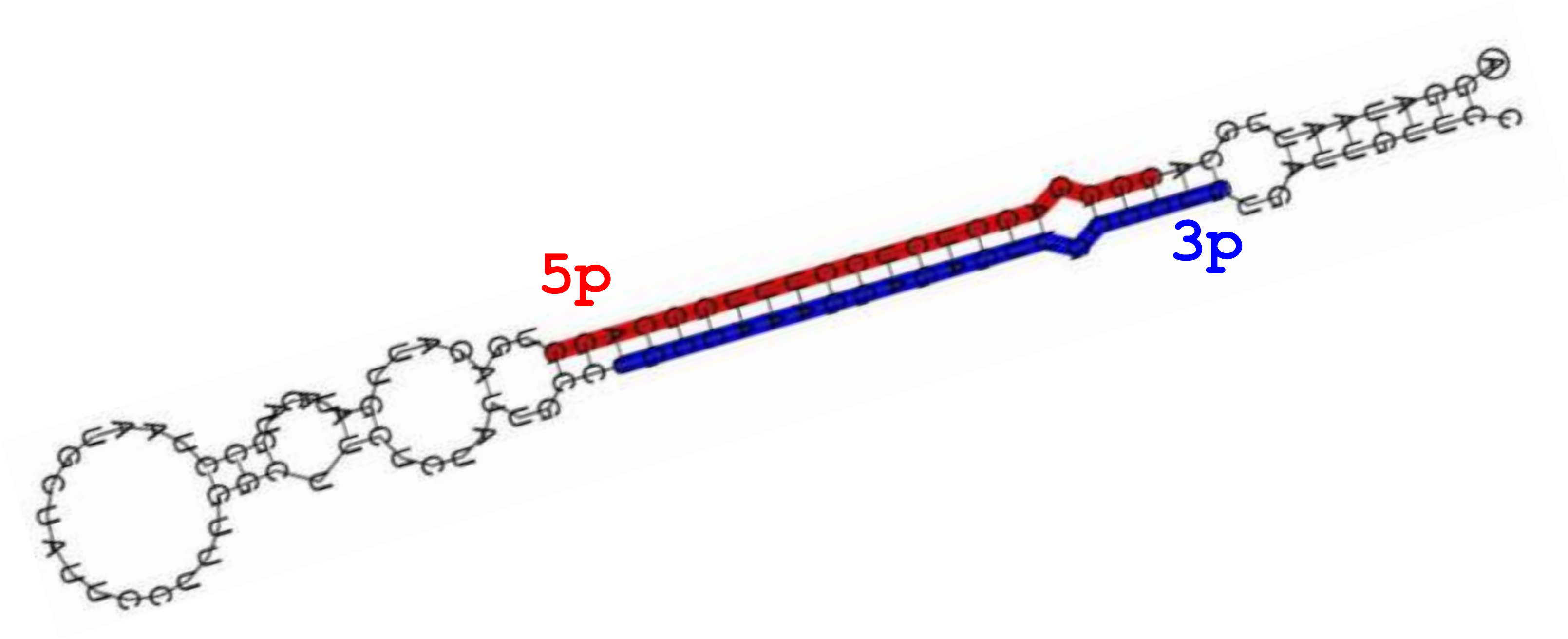

**Aang-miR399d**

```
5'->3'
TGCAGGGGAGCTCTCCTTTGGCGGGTGAGACTGATACATGCTTAATGCCATTACTTTGGCTTTCTCTACTGCCTGCCAAAGGAGAGTTGCCCTGTG
.((((((.((((((((((((((((((((((...............((((......))))..)))))....)))))))))))))))))))).))))))).
....GGGGAGCTCTCCTTTGGC................................................................    depth=3, length=18
....GGGGAGCTCTCCTTTGGCGG..............................................................    depth=6, length=20
.......................................................................TGCCAAAGGAGAGTTGCCCTG..    depth=120, length=21
```

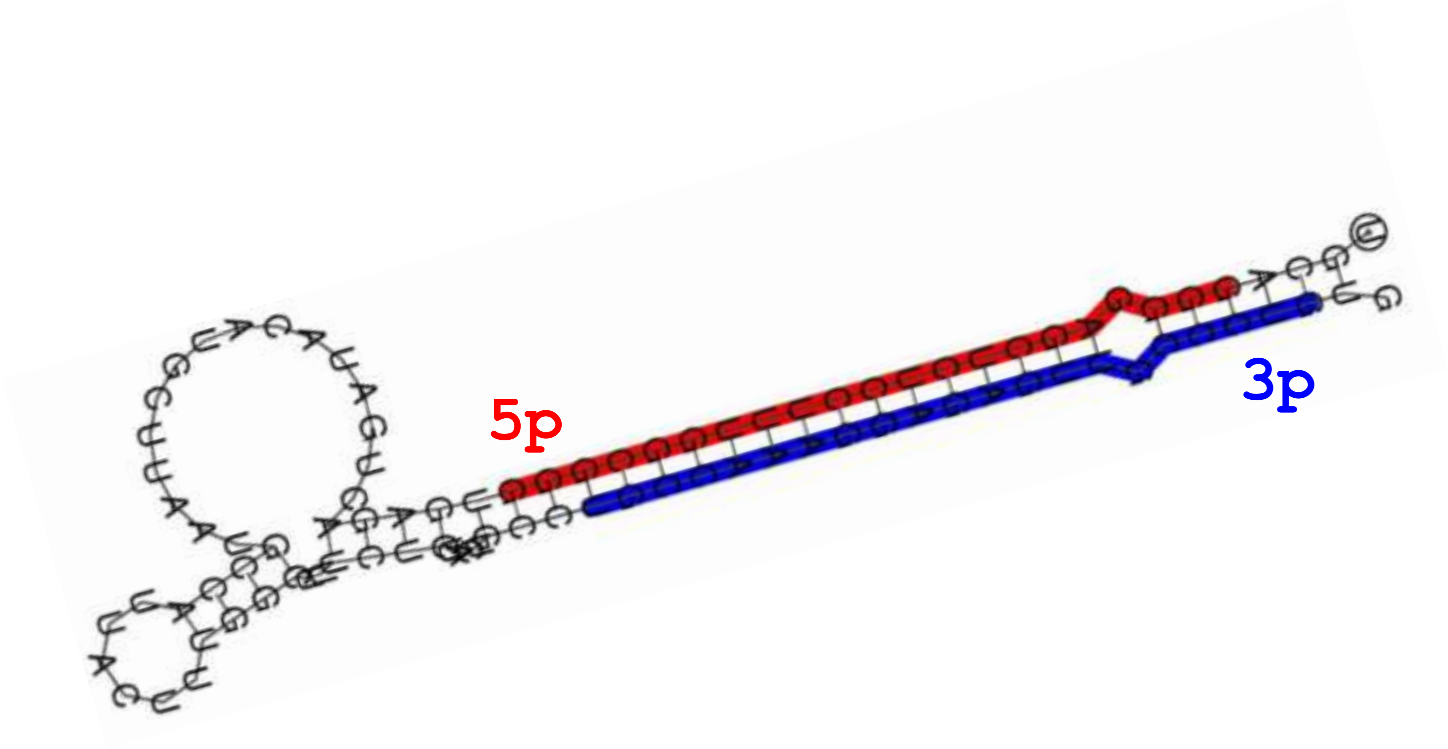

**Aang-miR399e**

5'->3'
AGGATAATTGCAGGGGAGCTCTCCTTTGGCAGGTGAGATTGATACATGCCTAATGCTATTCCTTTGGCTTTCTCTATTGCCTGCCAAAGGAGAGTTGCCCTGTGATTGTCCA
.((((((((((((((.((((((((((((((((((((....((.....(((...............)))..))....)))))))))))))))))))).)))))))))))))).
............GGGGAGCTCTCCTTTGGCAGG...............................................................................  depth=2, length=21
.................................................................................TGCCAAAGGAGAGTTGCCCTG..........  depth=120, length=21

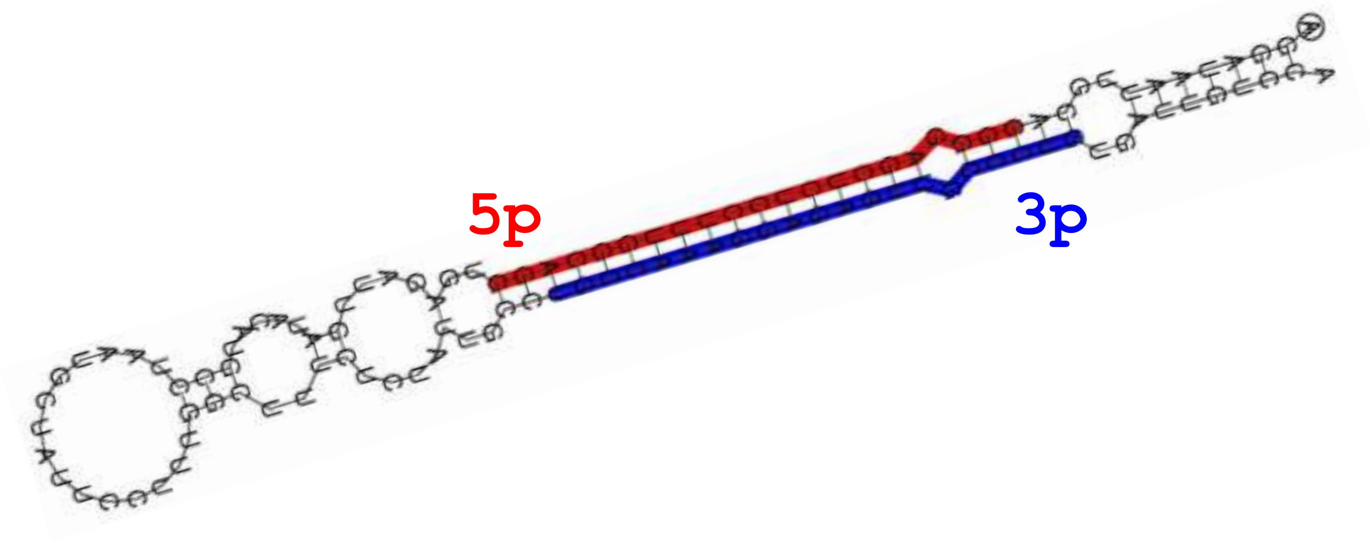

**Aang-miR408**

```
5'->3'
TAGGCAGCCGGGAAGAGATAGCGCATGGGAATTCCATTTACAGAAAGGCGTCTGTGTTTGATATTCTCATGCACTGCCTCTTCCCTGGCTGCT
.(((((((((((((((.(((.((((((((((..((..((((((......))))))..))..)))))))))).))).))))))))).))))))).
......GCCGGGAAGAGATAGCGCAT...................................................................       depth=1, length=20
.....................................................................TGCACTGCCTCTTCCCTGGCTG..       depth=79, length=22
```

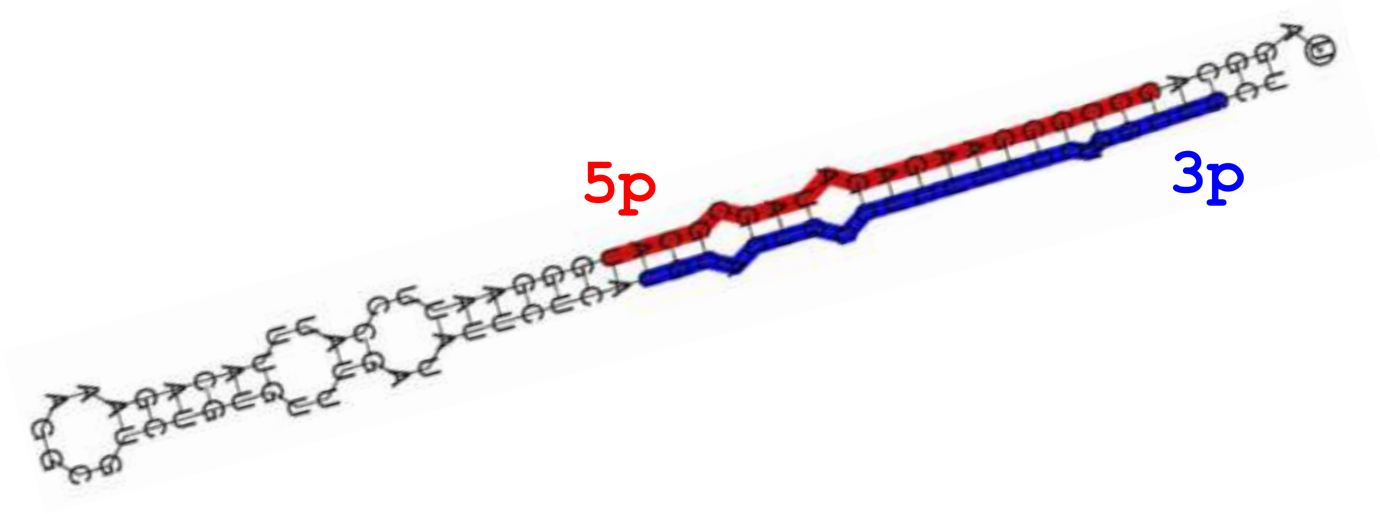

**Aang-miR529a**

```
5'->3'
AGCTAAAACTCGTAGAAGAAGAGAGAGAGCACAGCCTGCGCCGAAAAATCTAAAGTTCAAACTGTAGCCATAGTTTTCAGGCTGTGCTCTCTCTCTTCTCCAACATATTTTGGTG
.(((((((...((.(.((((((((((((((((((((((...........(((.(((....))).)))..........))))))))))))))))))))))).).))...))))))).
................AGAAGAGAGAGAGCACAGCCT..............................................................................   depth=357, length=21
.................GAAGAGAGAGAGCACAGCCT..............................................................................   depth=53, length=20
.................GAAGAGAGAGAGCACAGCCTG.............................................................................   depth=128, length=21
..............................................................................AGGCTGTGCTCTCTCTCTTC.................   depth=1, length=20
```

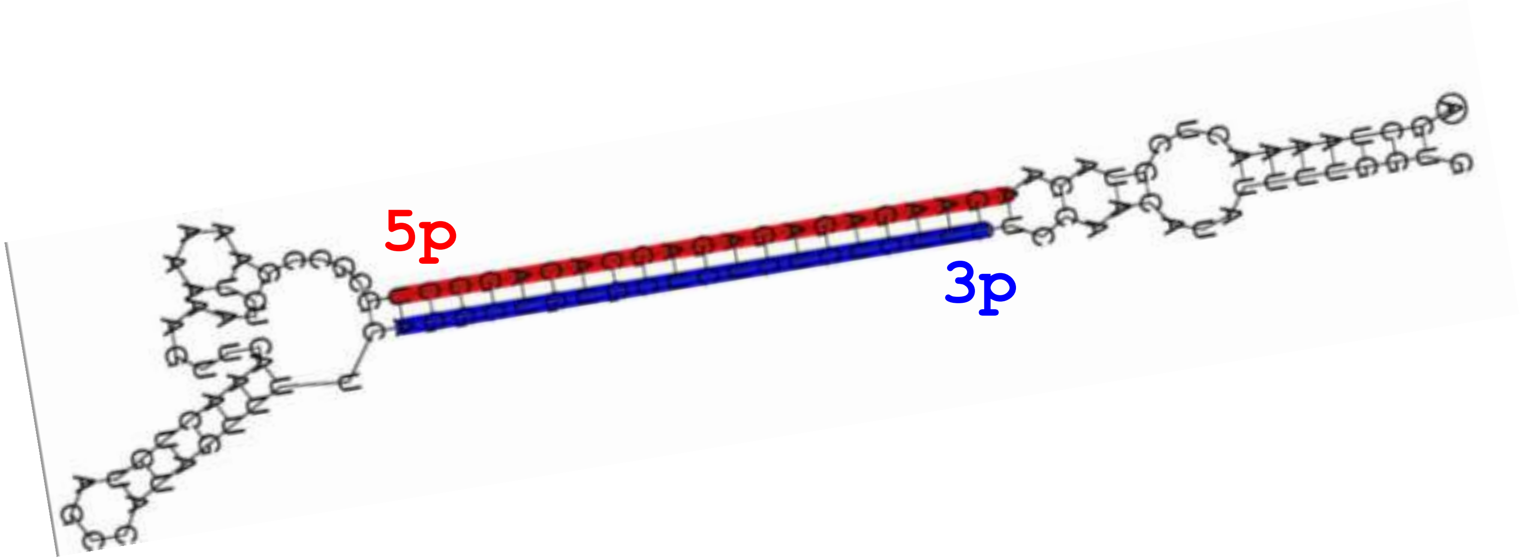

**Aang-miR529b**

```
5'->3'
TGGAGAAGAGAGAGAGCACAGCCTGAAAACTATGGCTACAGTTTGAACTTTAGATTTTTCGGCGCAGGCTGTGCTCTCTCTCTTCTTCT
.((((((((((((((((((((((((..........(((.(((....))).)))...........))))))))))))))))))))))))).
....GAAGAGAGAGAGCACAGCCT.................................................................   depth=53, length=20
...AGAAGAGAGAGAGCACAGCCT.................................................................   depth=357, length=21
....GAAGAGAGAGAGCACAGCCTG................................................................   depth=128, length=21
...................................................................GCTGTGCTCTCTCTCTTCTTC.   depth=2, length=21
```

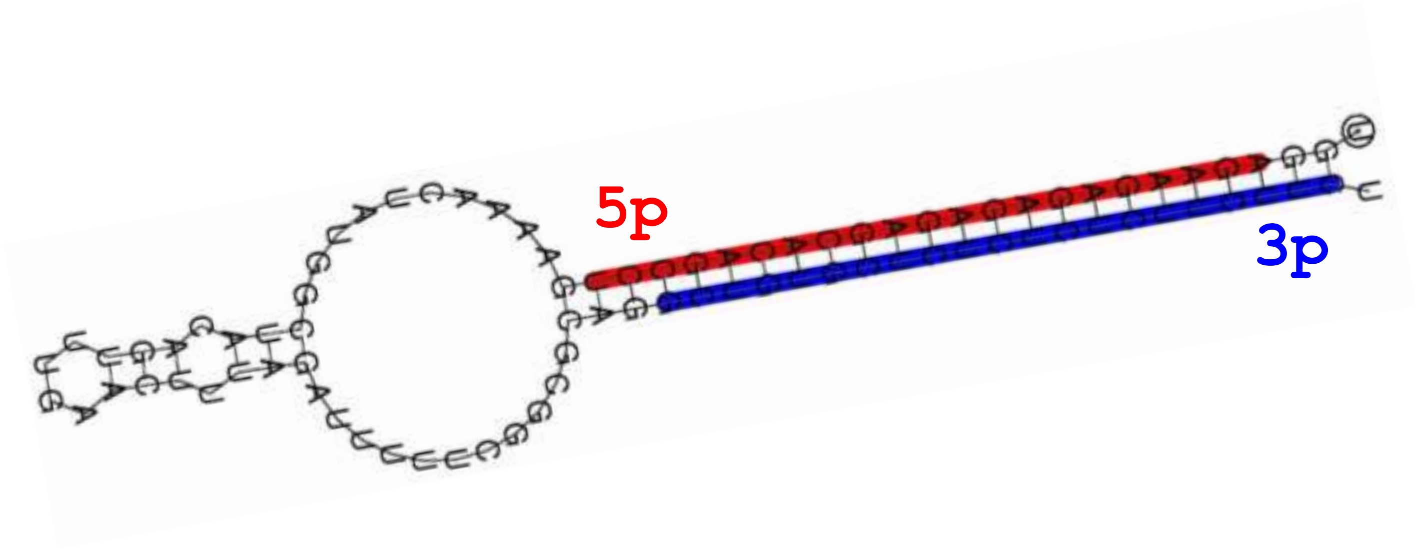

**Aang-miR529c**

```
5'->3'
AAACTCATAGAAGAAGAGAGAGAGCACAGCCTGTGCCGAAAAATCTAAAGTTCAAACTGTAGCCATAGTTTTCAGTTATCAGGCAGTGGCTATGCTCTCTCTCTTCTCCTTCAGGGTTC
.(((((..((.(((((((((((((((.((((..((((.................(((((.(((....)))..)))))....))))..)))).))))))))))))))).))...))))).
...........AGAAGAGAGAGAGCACAGCCT.......................................................................................
        depth=357, length=21
............GAAGAGAGAGAGCACAGCCT.......................................................................................
        depth=53, length=20
............GAAGAGAGAGAGCACAGCCTG......................................................................................
        depth=128, length=21
```

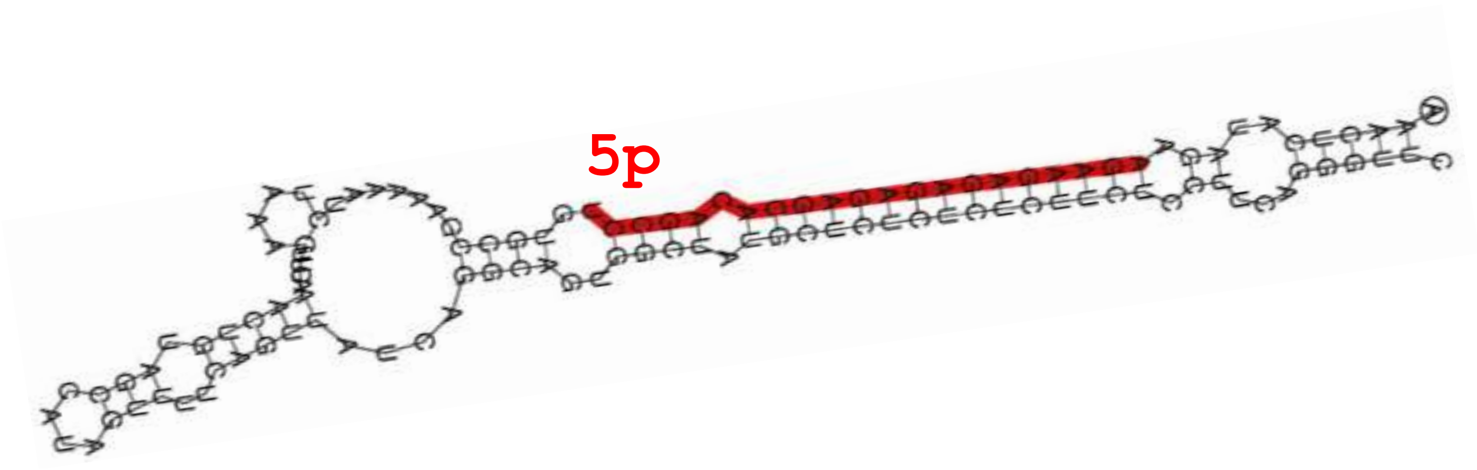

**Aang-miR529d**

```
5'->3'
TAGAAGAAGAGAGAGAGCACAGCCTGTGCCGAAAAATCTAAAGTTCAAACTGTAGCCATAGTTTTCAGTTATCAGGCAGTGGCTATGCTCTCTCTCTTCTCCTT
.((.((((((((((((((.((((..((((.................(((((.(((....)))..)))))....))))..)))).))))))))))))))).)).
.....GAAGAGAGAGAGCACAGCCT...............................................................................      depth=53, length=20
....AGAAGAGAGAGAGCACAGCCT...............................................................................      depth=357, length=21
.....GAAGAGAGAGAGCACAGCCTG..............................................................................      depth=128, length=21
```

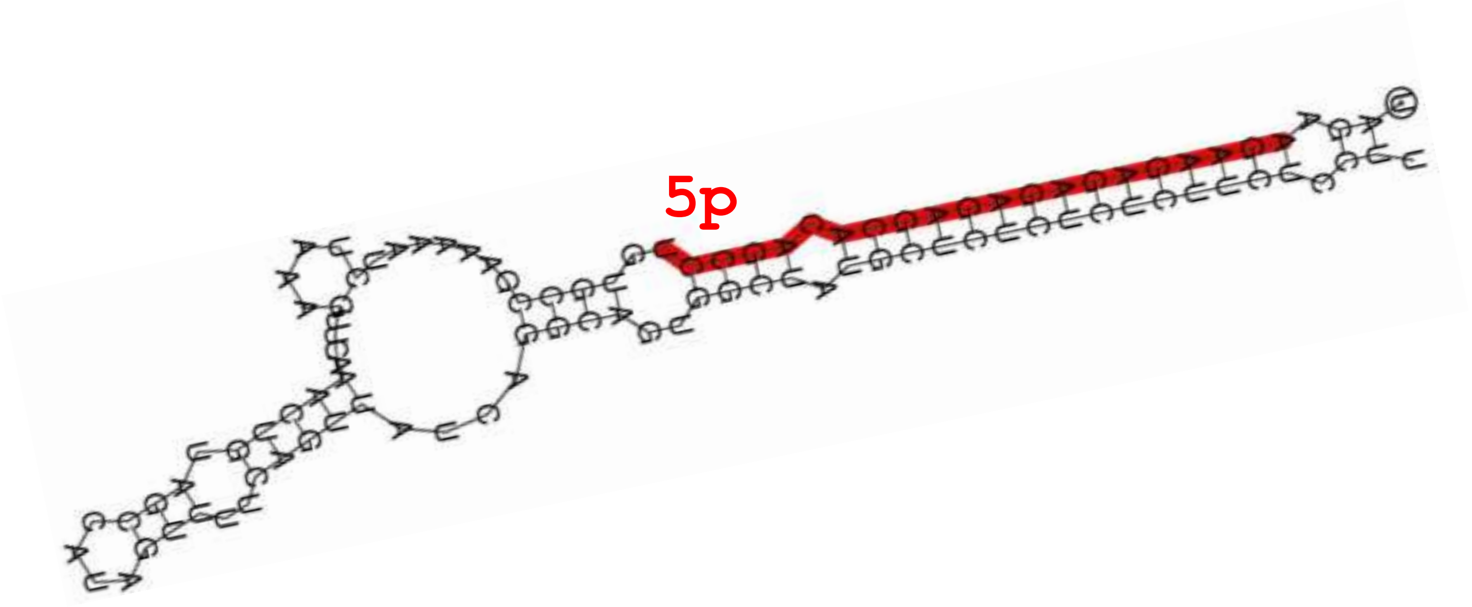

**Aang-miR529e**

```
5'->3'
GAAGGAGAAGAGAGAGAGTACAGCCCTGCCTGATAACTGAAAACTATGGCTACAGTTTGAACTCTAGAGTTTTCGGCACAGGTTGTGCTCTCTCTCTTCTTCT
.(((((((((((((((((((((((.((((........(((((((....(((.(((....))).))))))))))))))..))))))))))))))))))))))).
.....AGAAGAGAGAGAGTACAGCCC.............................................................................  depth=35, length=21
.................................................................................GTTGTGCTCTCTCTCTTCTTC.  depth=383, length=21
..................................................................................TTGTGCTCTCTCTCTTCTTC.  depth=21, length=20
```

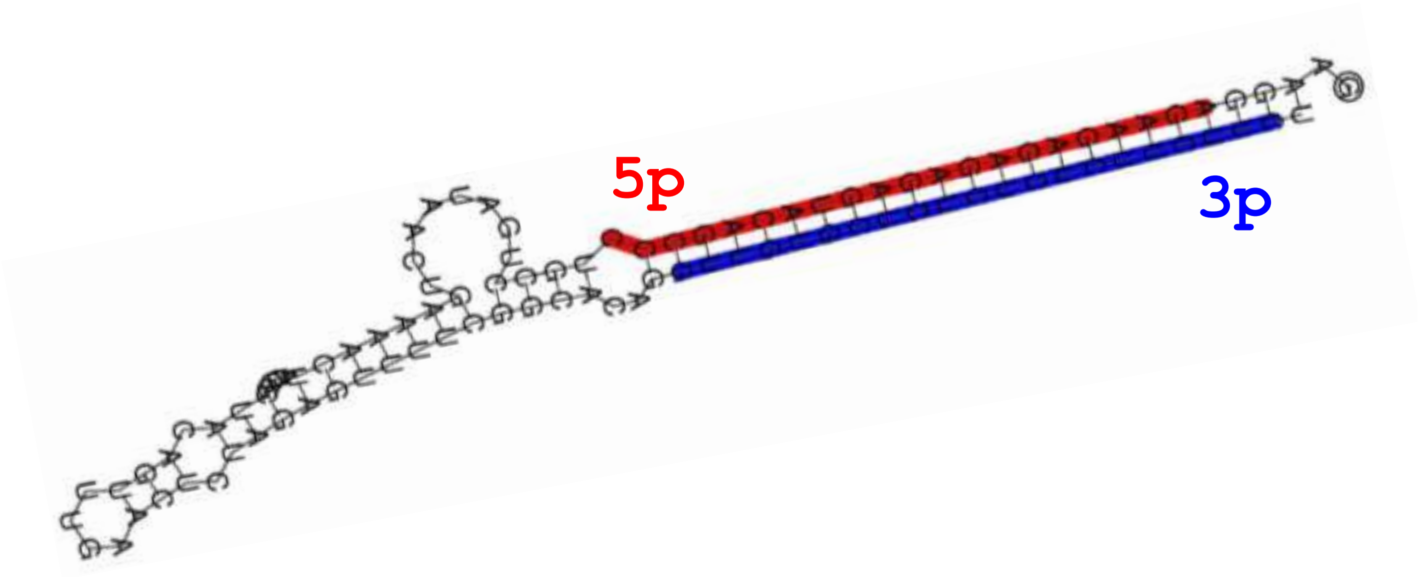

**Aang-miR1314a**

```
5'->3'
ACTCTCCTACATTTAGGGTCGCCGCACCATAAAAAGTTGCATAAAGCGTCGGCCTTGAATGTTAGGAGAGA
.(((((((((((((((((((((.(((..................))).))))))))))))).))))))))).
...CTCCTACATTTAGGGTCGCC................................................     depth=626, length=20
...CTCCTACATTTAGGGTCGCCG...............................................     depth=1188, length=21
...............................................GTCGGCCTTGAATGTTAGGAGA..     depth=227, length=22
................................................TCGGCCTTGAATGTTAGGAGAG.     depth=4523, length=22
```

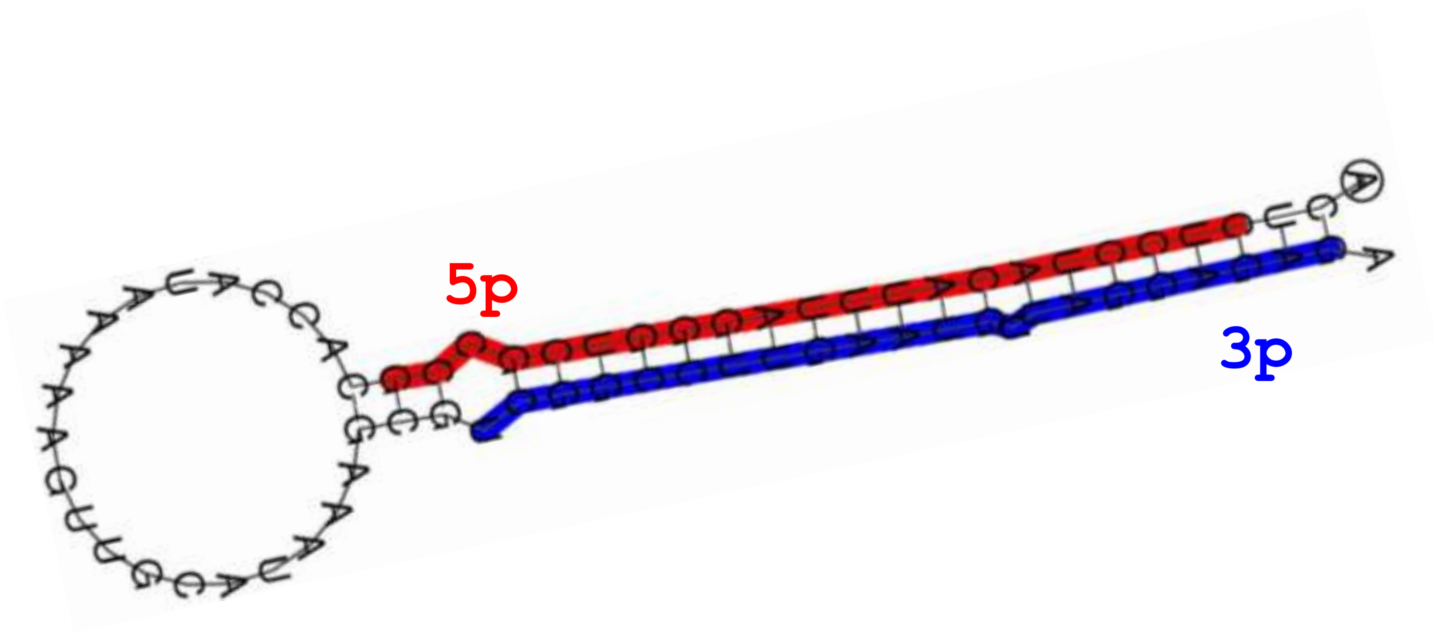

# Aang-miR1314b

```
5'->3'
TGGTAAGCAATGAAGGAAACTCTCCTACATTTAGGGTCGCCGCACCATAAAAAGTTGCATAAAGCGTCGGCCTTGAATGTTAGGAGAGAGTCTTTCTTTGTTTGCCC
.(((((((((.((((((..((((((((((((((((((((.(((....................))).)))))))))))).))))))))..)))))).))))))))).
.....................CTCCTACATTTAGGGTCGCC..................................................................   depth=626, length=20
.....................CTCCTACATTTAGGGTCGCCG.................................................................   depth=1188, length=21
.................................................................GTCGGCCTTGAATGTTAGGAGA....................   depth=227, length=22
..................................................................TCGGCCTTGAATGTTAGGAGAG...................   depth=4523, length=22
```

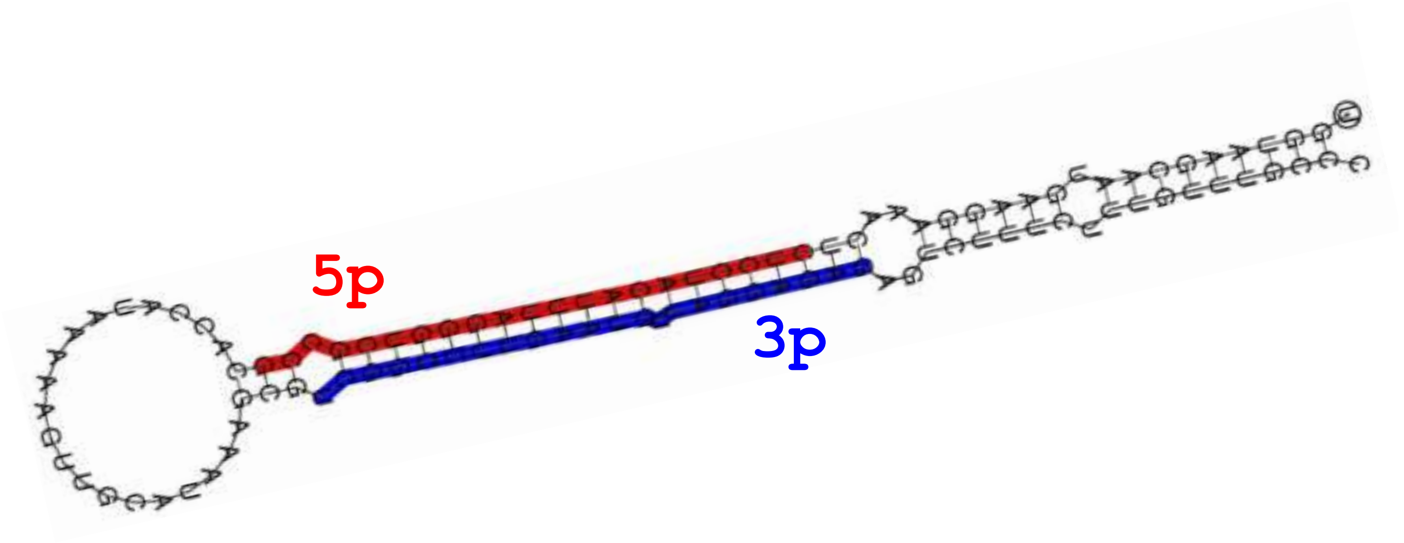

# Aang-miR1314c

5'->3'

```
TGGCATAGATGGCCTGGTAGGCAACGAACGAAACCCTTCTAAATTTAAGGTCGCCGCTCCATCAAAAGTTGTGTAAAGCGTCGGCCTTGAATGTTAGGAGAGAGTCTTTCTTTGTTTGCCCATGCCATCTTGCCT
.((((.((((((((..((((((((.(((.((..(.((((((((((((((((((.((((.(((........)))...)))).))))))))))).))))))).)..)).))).)))))))))...))))))))))).
...................................CTTCTAAATTTAAGGTCGCC................................................................................   depth=135, length=20
...................................CTTCTAAATTTAAGGTCGCCG...............................................................................   depth=560, length=21
...............................................................................GTCGGCCTTGAATGTTAGGAGA..................................   depth=227, length=22
................................................................................TCGGCCTTGAATGTTAGGAGAG.................................   depth=4523, length=22
```

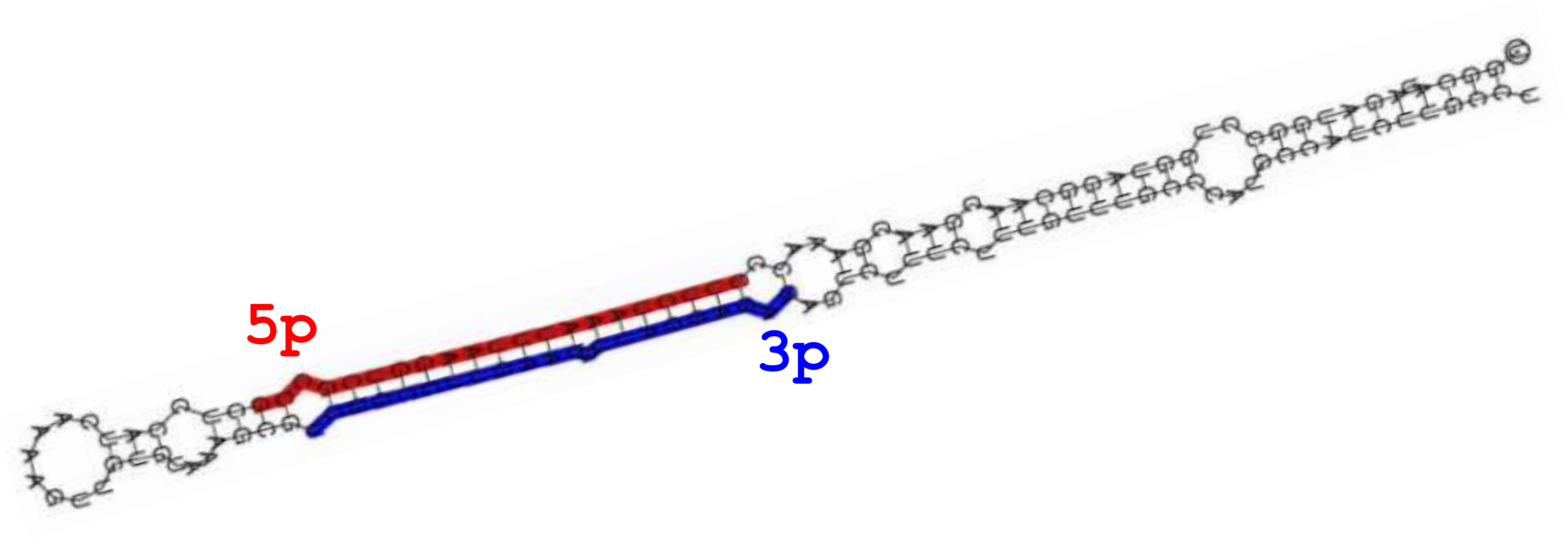

**Aang-miR1314d**

```
5'->3'
AGATGGCCTGGTAGGCAACGAACGAAACCCTTCTAAATTTAAGGTCGCCGCTCCATCAAAAGTTGTGTAAAGCGTCGGCCTTGAATGTTAGGAGAGAGTCTTTCTTTGTTTGTCCATGCCATCC
.((((((.((((((((((.(((.((..(.((((((((((((((((((.((((.(((........)))...)))).))))))))))).))))))).)..)).))).))))))).))).)))))).
.............................CTTCTAAATTTAAGGTCGCC...........................................................................depth=135, length=20
.............................CTTCTAAATTTAAGGTCGCCG..........................................................................depth=560, length=21
.........................................................................GTCGGCCTTGAATGTTAGGAGA.............................depth=227, length=22
..........................................................................TCGGCCTTGAATGTTAGGAGAG............................depth=4523, length=22
```

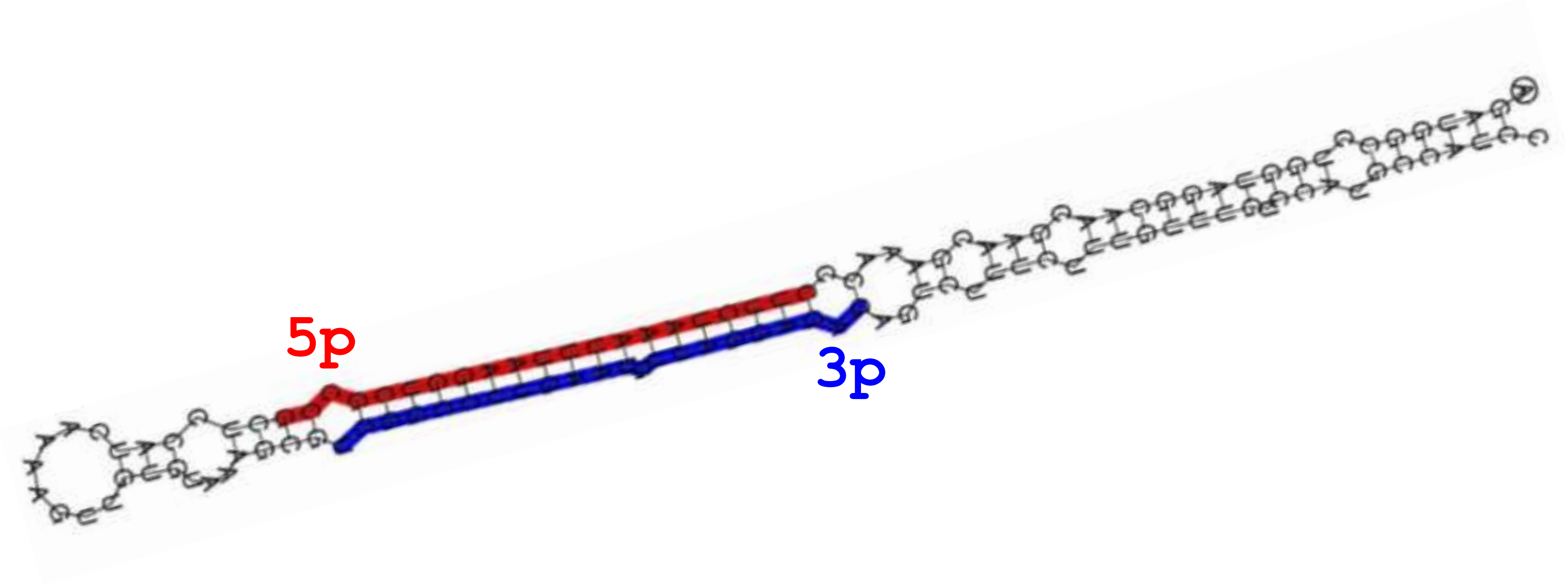

Supplement: Supplementary file 1 [file Data_Sheet_1.PDF]
