# Supplementary material for: Novel and Conserved miRNAs Among Brazilian Pine and Other Gymnosperms
Source: Front Genet. 2019 Mar 22;10:222. doi: 10.3389/fgene.2019.00222 (PMC6448024; doi:10.3389/fgene.2019.00222)

Data S2. Anchoring patterns and stem-loop structures of novel miRNAs of *A. angustifolia*. The sequences corresponding to the most abundant mature miRNAs in the 5p and 3p arms are labeled in red and blue, respectively. Values on the right side represent mature miRNAs and iso-miRNAs read counts.

Aang-nmiR001

```
5'->3'
CCACTGTGGGATGATGTCAAAAATGGAGTCGTAGGCGGATGATGTGAACACATTCTTCACTTTTTTCTTTTTTTGACATCACACCCGCGGTGA
.((((((((.(((((((((((.((((....(((.(((.(((((....))))).))).)))..)))).)))))))))))..)))))))).
.CACTGTGGGATGATGTCAAAA........................................................................  depth=115, length=21
..ACTGTGGGATGATGTCAAAAA......................................................................  depth=945, length=21
.......................................................................TTTGACATCACACCCGCGGT..  depth=3538, length=20
.......................................................................TTTGACATCACACCCGCGGTGA  depth=294457,length=22
```

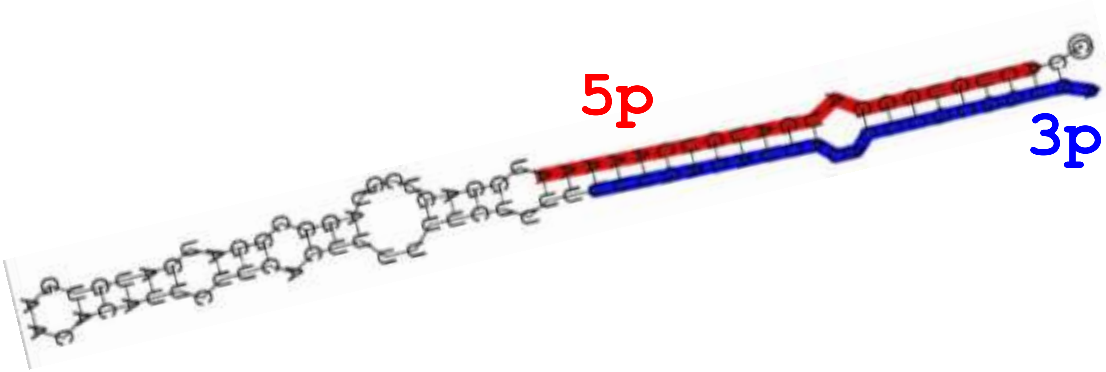

Aang-nmiR002

```
5'->3'
TATGTATTCATCTATCACTGTGGAAATGAGGTGAACTACACCCGTTTACATCCTTCCCCGGTGATTGATGAAGACATT
.((((.((((((.(((((((.((((..((.((((((......)))))).)).)))).))))))).)))))).)))).
....TATTCATCTATCACTGTGGA..........................................................      depth=530, length=20
....TATTCATCTATCACTGTGGAA.........................................................      depth=637, length=21
....TATTCATCTATCACTGTGGAAA........................................................      depth=4092, length=22
.......................................................TCCCCGGTGATTGATGAAGAC...      depth=58335, length=21
.......................................................TCCCCGGTGATTGATGAAGACA..      depth=231531, length=22
```

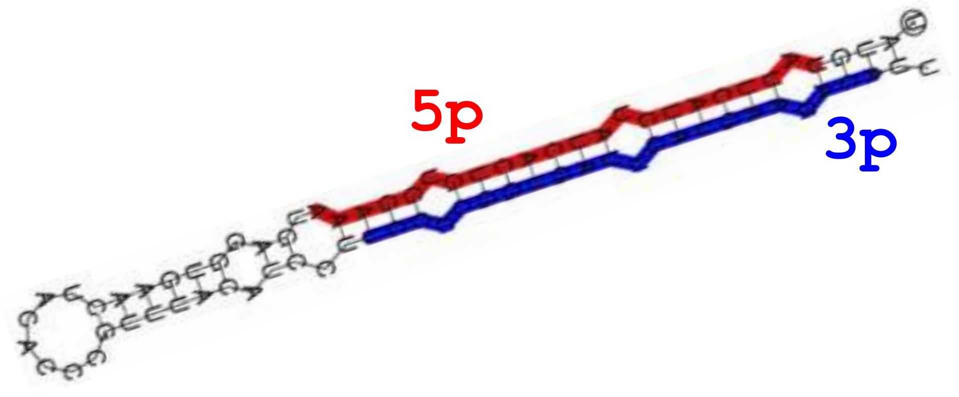

Aang-nmiR003

```
5'->3'
CGGCGTGGGCGACCGGGGAAAATTTTTCTCATATCGATCTTGTTATTCTTATATCAAAGCAGAAGAATTTTCCCTGATCCGCCCATGCCC
.(((((((((((.((((((((((((((((......(((..............))).....)))))))))))))))).).)))))))))).
...CGTGGGCGACCGGGGAAAAT...................................................................   depth=12, length=20
..GCGTGGGCGACCGGGGAAAAT...................................................................   depth=31, length=21
...CGTGGGCGACCGGGGAAAATT..................................................................   depth=51, length=21
....................................................................TTTCCCTGATCCGCCCATGCC.   depth=2173, length=21
...................................................................TTTTCCCTGATCCGCCCATGCC.   depth=102252, length=22
....................................................................TTTCCCTGATCCGCCCATGCCC   depth=26158, length=22
```

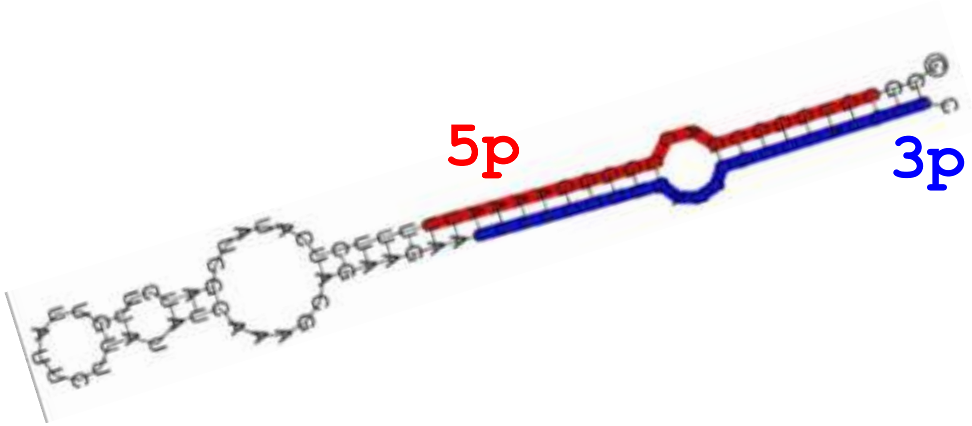

Aang-nmiR004

```
5'->3'
TGAAGACTTTGACATTCCTGTTGCGGAGGTGGTAGTAGACCAGACTCGCCATCTAACAAGACTCGCCATCTGGATGGCGAGTCGGATCTACTACAACCTCCGCAACAGGAATGTCAAAGTCTTCAT
((((((((((((((((((((((((((((((.((((((((((.(((((((((((((...............))))))))))))))).)))))))).))))))))))))))))))))))))))))))).
................................TAGTAGACCAGACTCGCCATC.........................................................................          depth=82793, length=21
...........................................................................GCGAGTCGGATCTACTACAAC..............................          depth=189, length=21
..............................................................................GAGTCGGATCTACTACAACCT...........................          depth=108, length=21
.............................................................................CGAGTCGGATCTACTACAACCT...........................          depth=243, length=22
```

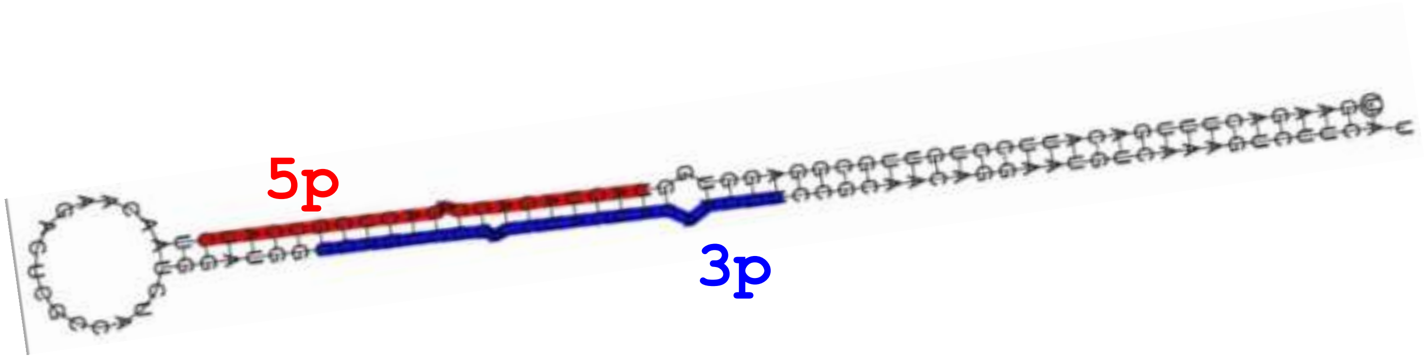

Aang-nmiR005

```
5'->3'
GTACTCTTGACAGCCCGAAATCAGCGAGTTTGCTATGCGAAGACAATGGAGTATCAAACTGGCTGATTCGGACTATCAAGAGTGA
.(((((((((.((.(((((.(((((.((((((..((((............)))))))))).)))))))))).)).))))))))).
.......TGACAGCCCGAAATCAGCGAG.........................................................      depth=5174, length=21
.......TGACAGCCCGAAATCAGCGAGT........................................................      depth=75705, length=22
...........................................................TGGCTGATTCGGACTATCAAG.....      depth=311, length=21
...............................................................TGATTCGGACTATCAAGAGTG.      depth=58, length=21
```

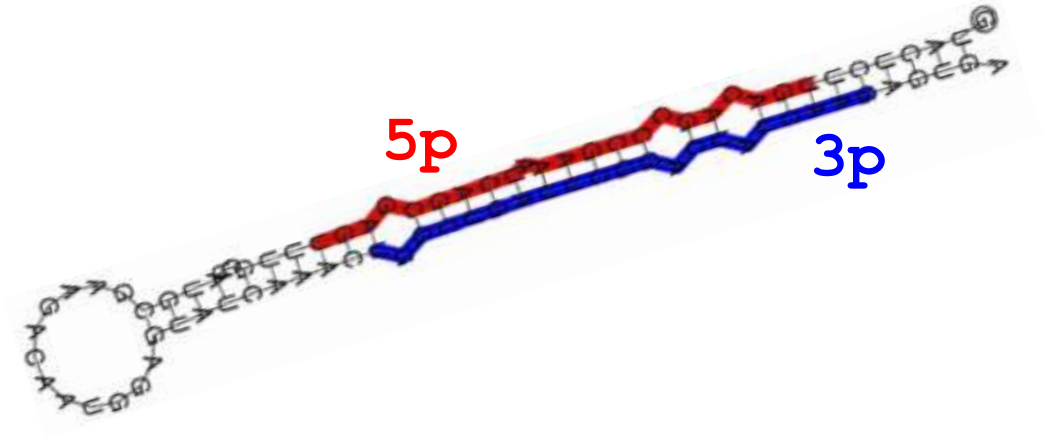

Aang-nmiR006

5'->3'
CAGGTGGTCGGCGAGAAGAATCCTTTCCCGATGTGGTCCGGAACATCAATTCCAGGTGGTCGGCGAGAAGATTCTTTTGTAGACGACCTA
.((((.(((.((.(((((((((.(((((((((.....((((((......)))).))..))))).)))).))))))))))).))).)))).
..GGTGGTCGGCGAGAAGAAT... depth=1058, length=19
..GGTGGTCGGCGAGAAGAATC.. depth=1663, length=20
...GTGGTCGGCGAGAAGAATC.. depth=9778, length=19
...GTGGTCGGCGAGAAGAATCC... depth=37665, length=20
...GTGGTCGGCGAGAAGAATCCT.. depth=14686, length=21

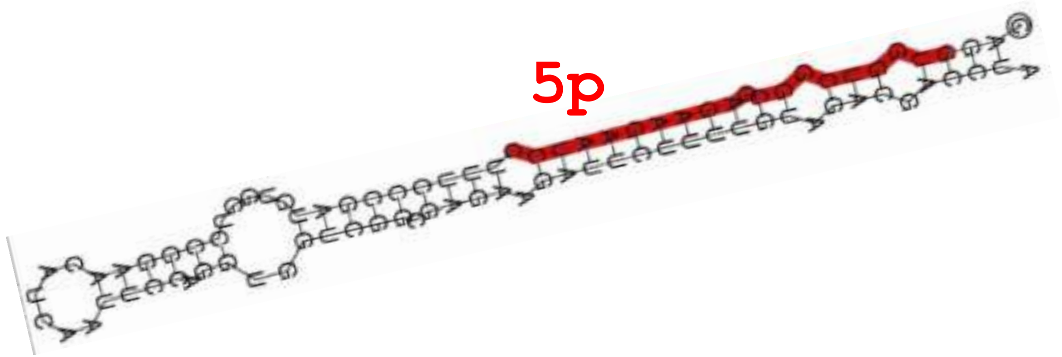

Aang-nmiR007

```
5'->3'
TTGGTCTTTGTGATATTTAACTTCTTTTGTTGTGGGCTTACATGTCTGTCGATGTTGTAGCTAGCTCTGGCTCATTCCATTTATAGCTACGATATCAGCAGACATGTAGGCCAACCACCACTAGTTATATCATAGCGATTAT
.(((((.((((((((..(((((......((.((.(((((((((((((((.(((((((((((((....(((......)))....))))))))))))).))))))))))))))).)).))....))))))))))))).))))).
.................................GGGCTTACATGTCTGTCGATG........................................................................................          depth=14, length=21
................................TGGGCTTACATGTCTGTCGATG........................................................................................          depth=141, length=22
............................................TCTGTCGATGTTGTAGCTAGC.............................................................................          depth=78, length=21
..............................................................................................TCAGCAGACATGTAGGCCAAC...........................          depth=613, length=21
..............................................................................................TCAGCAGACATGTAGGCCAACC..........................          depth=71657, length=22
..............................................................................................TCAGCAGACATGTAGGCCAACCA.........................          depth=254, length=23
```

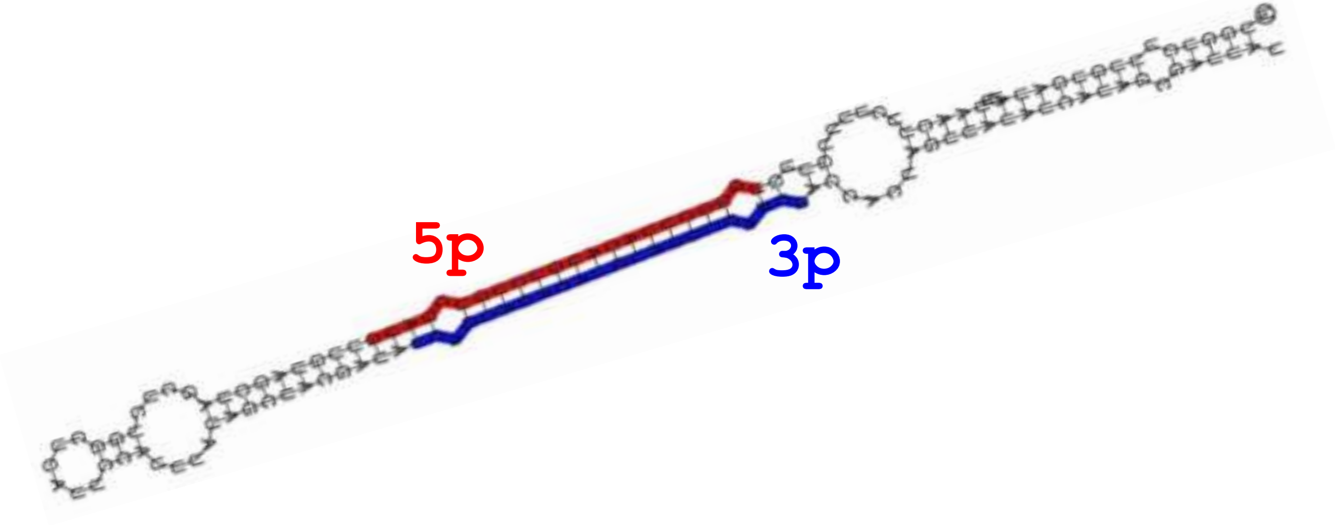

Aang-nmiR008

```
5'->3'
TGGAGAGTTTTGCATCCCAAACATCGTCCAGAAATACAGGGTATTTATTTTAGTGTCTTTATCTTGGTCTTAGATACCCTCTAGTTTGGACGATGTTTGGAATGCAAAACATCTTCT
.((((((((((((((.((((((((((((((((..((.((((((((((.......................)))))))))).)).)))))))))))))))).))))))))).))))).
........TTTGCATCCCAAACATCGTCC........................................................................................   depth=7939, length=21
..............TCCCAAACATCGTCCAGAAA...................................................................................   depth=774, length=20
..............TCCCAAACATCGTCCAGAAAT..................................................................................   depth=10308, length=21
..............TCCCAAACATCGTCCAGAAATA.................................................................................   depth=41135, length=22
...................................................................................GTTTGGACGATGTTTGGAATG.............   depth=548, length=21
.........................................................................................ACGATGTTTGGAATGCAAAAC.......   depth=376, length=21
```

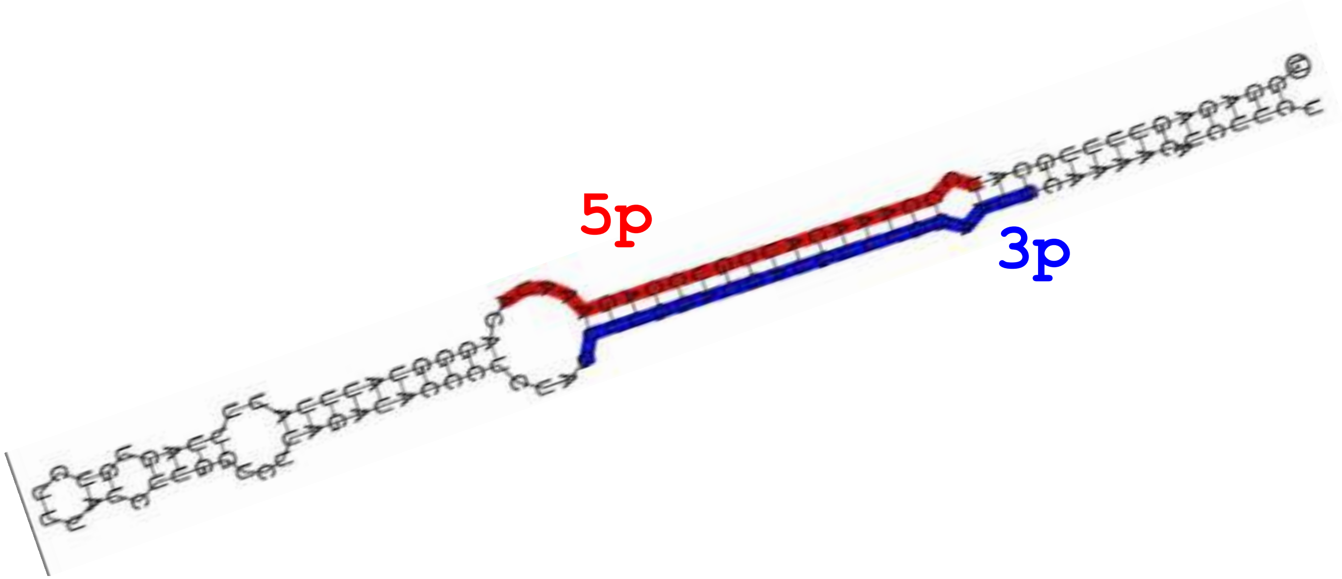

Aang-nmiR009

```
5'->3'
ATGGCGTCAGAGGTGATATCTCGAACATCCTGCAGCCATTCTCTATTCCCTTTGCTCTGCTTGGCCTAGAGATTGTGCCATCAGTCAAAGTGCAGTGGCTGCACGACTTCGAGATATTTCCTTTGACGCCAC
.((((((((((((.(((((((((((..((.((((((((((........(((((..(((..(((((........).)))).))).)))))...)))))))))).)).))))))))))).)))))))))))).
..................TCTCGAACATCCTGCAGCC...............................................................................................  depth=381, length=19
..................TCTCGAACATCCTGCAGCCA..............................................................................................  depth=371, length=20
..................TCTCGAACATCCTGCAGCCAT.............................................................................................  depth=922, length=21
..................TCTCGAACATCCTGCAGCCATT............................................................................................  depth=44752, length=22
...............................................................................................TGGCTGCACGACTTCGAGAT.................  depth=13, length=20
................................................................................................GGCTGCACGACTTCGAGATA................  depth=13, length=20
...............................................................................................TGGCTGCACGACTTCGAGATA................  depth=546, length=21
```

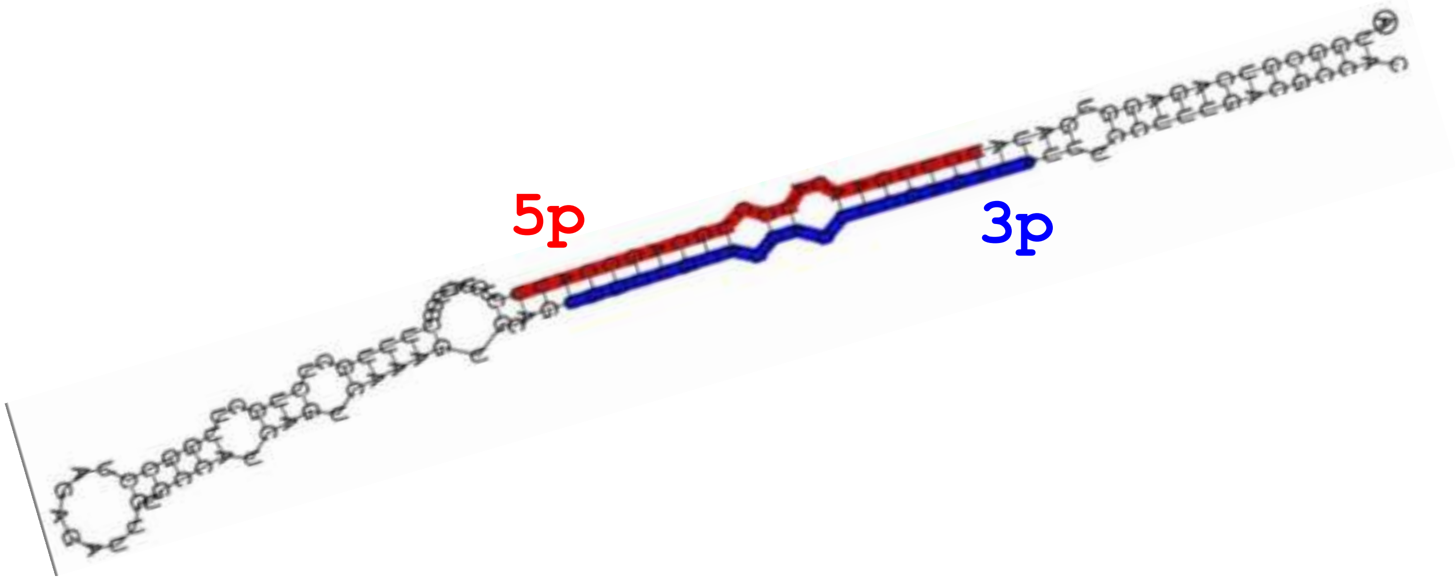

Aang-nmiR010

5'->3'
AGGAGGCGGAGGAGGCGAGACATGACTGGTAAACAGATGGGGCACTAAAAATGAGAAGTGCTGTTAAGAAAGATGGTGTTATATTTAGTGTTCTATTCATCTGCTTGTGCTTTTCTAAATGGAAATGT
AGTCCGATCTGGACAGAGTTATTGACGATAATAGATCTATTGCCTTCTTGAAGCAGTATTTGTTTTGTTTAGCGCCCCATCTGATTACCGGTCTTTCTCTCCTTGCCTAAGTTTC
.(((((..((((((.((((...(((((((((.((((((((((.(((((...((.((((((((((....((((.((((.((.(((((....((...(((...((((.((((((((.....))))).)))
((((.....)))).))))...))).))...))))).)).)))).))))..)))))))))).))...))))).)))))))))).)))))))))..)))).)))..)))..))))).
.........................CTGGTAAACAGATGGGGCACT..
....
depth=181, length=21
....
....CGCCCCATCTGATTACCGGTC......................
depth=36806, length=21

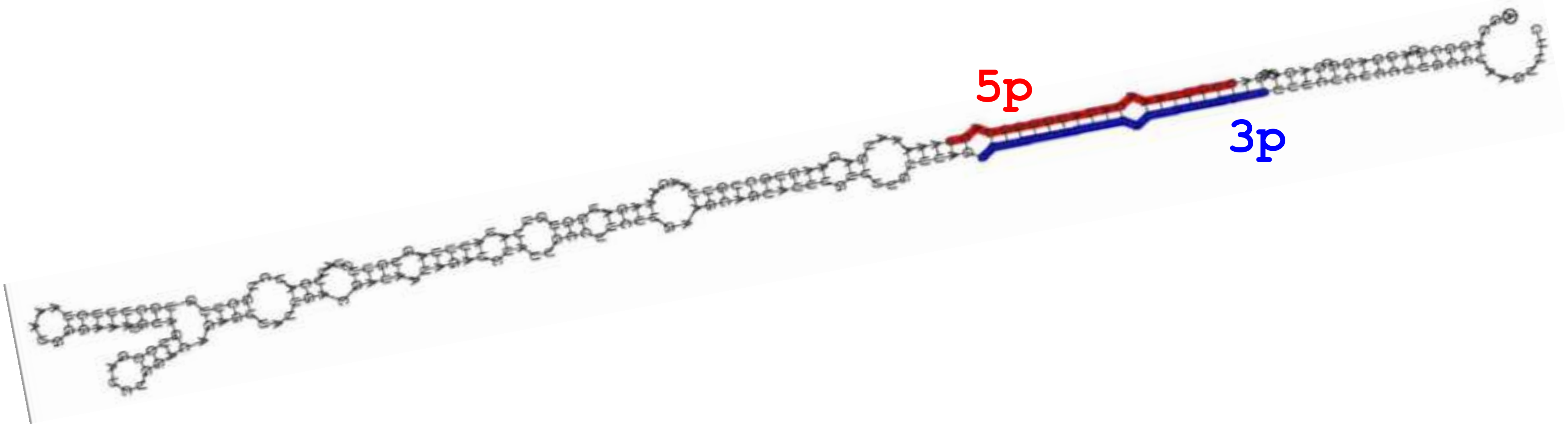

Aang-nmiR011

```
5'->3'
TCGTCTATCACCGTGGACCGATGTAAAAAAATTACATTGCCCTTTCTGTGATTTTTATTTTTATTTTTTACGTCAGGTCCTCTGTGATTGATGC
.((((.(((((.(.((((((((((((((((((((((..........)))))).........)))))))))))).))))).).))))).)))).
.......TCACCGTGGACCGATGTAAAA..................................................................      depth=354, length=21
........CACCGTGGACCGATGTAAAAA.................................................................      depth=77, length=21
..................................................................TTTACGTCAGGTCCTCTGTGAT......      depth=1724, length=22
...................................................................TTACGTCAGGTCCTCTGTGATT.....      depth=17626, length=22
```

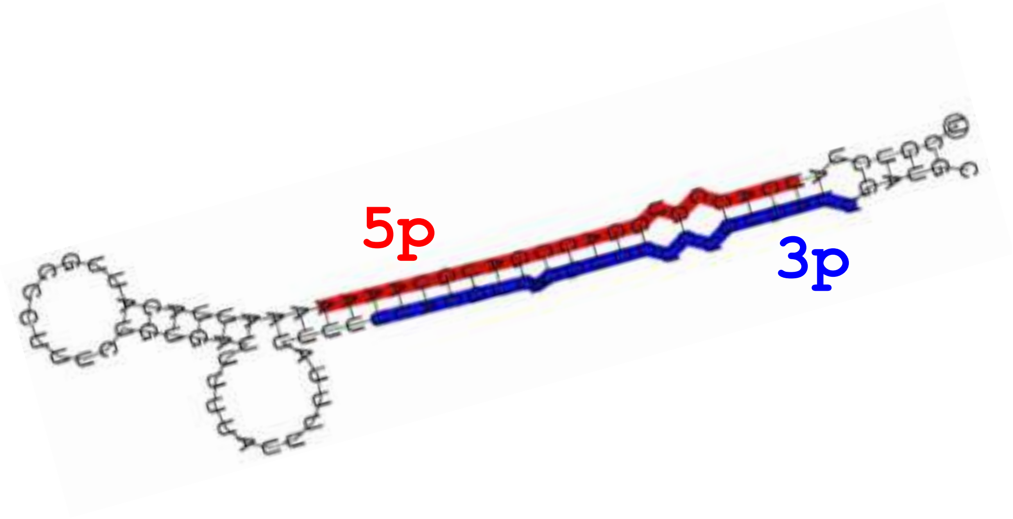

Aang-nmiR012

```
5'->3'
AATGCAACCACCATCCGGCACTTGATGTCAAAAATTACAATGCTTGCTCTTTGATCTTTTTGACGTCAGGTCCTCGATGGTTGATGCATA
.(((((.(.((((((.((.(((((((((((((((...(((.........)))...))))))))))))))))))..)))))).).))))).
..........CCATCCGGCACTTGATGTCAA..........................................................   depth=5, length=21
..........CCATCCGGCACTTGATGTCAAA.........................................................   depth=39, length=22
.............................................................TGACGTCAGGTCCTCGATGG..........   depth=814, length=20
.............................................................TGACGTCAGGTCCTCGATGGTT........   depth=16623, length=22
```

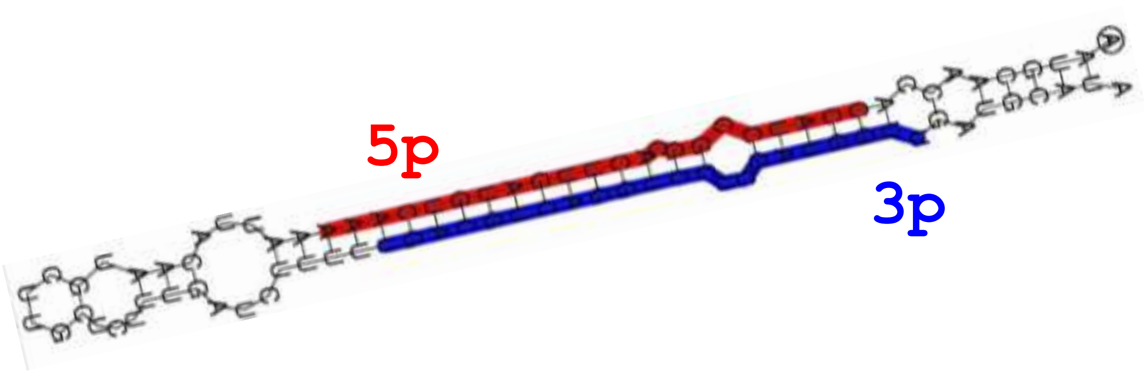

Aang-nmiR013

```
5'->3'
CGTATTCAACCACCATTGAGCGCTTGGTGTCAAAAAATTCAATGTCAGCTCTTTGGTTTTTTTAACATCAGGCCCTCGATGATTGATGAATACC
.(((((((.(...((((((.(((((((((.((((((.((((.(......).)))).)))))).))))))))).)))))))...).))))))).
............CCATTGAGCGCTTGGTGTCAA.............................................................          depth=4847, length=21
............CCATTGAGCGCTTGGTGTCAAA............................................................          depth=7534, length=22
..............................................................TAACATCAGGCCCTCGATGATT..........          depth=310, length=22
```

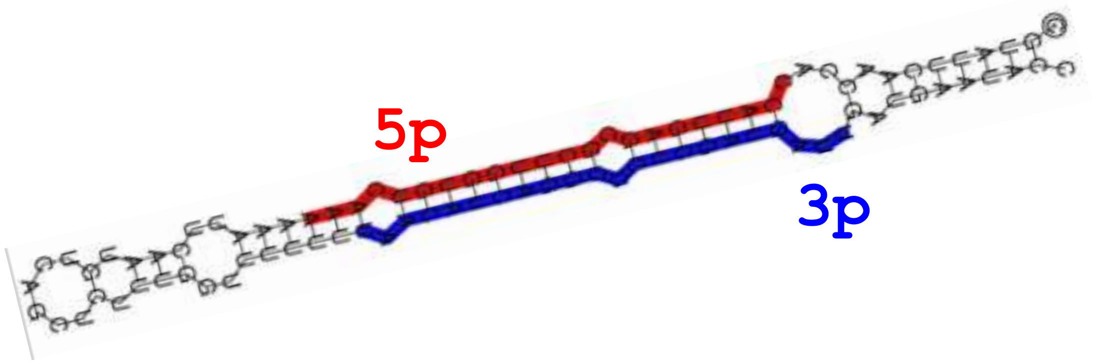

Aang-nmiR014

```
5'->3'
ATTCTTGGATTTATGGAAGACGAACCCGACGAACCTGCACAAGCAAGGATGTTTTCATTAATCAAGAAC
.(((((((..(((..((((((((...........(((((....)).))).))))))))..))))))))))).
..TCTTGGATTTATGGAAGACGAAC..............................................        depth=785, length=23
..TCTTGGATTTATGGAAGACGAACC.............................................        depth=8615, length=24
...CTTGGATTTATGGAAGACGAACC.............................................        depth=2928, length=23
..............................................AGGATGTTTTCATTAATCAAGAAC        depth=19, length=24
...............................................GGATGTTTTCATTAATCAAGAAC        depth=9, length=23
................................................GATGTTTTCATTAATCAAGAAC        depth=9, length=22
```

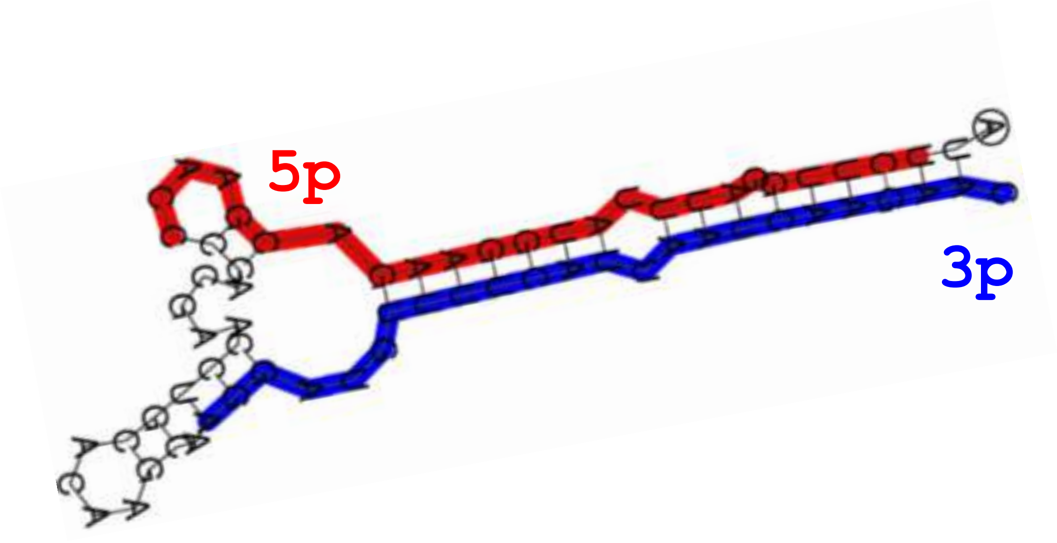

Aang-nmiR015

```
5'->3'
TGGATCTGGGTCGTCACGGTCGGTCCGCCTTCTTGGGTGTGCACCTGCCCTCACGTCCCTTCTG
.(((...(((.(((...(((.((((((((((....)))))).).))).))).)))...))).))).))).
......TGGGTCGTCACGGTCGGTC.....................................    depth=544, length=19
.......GGGTCGTCACGGTCGGTCC....................................    depth=2157, length=19
......TGGGTCGTCACGGTCGGTCC....................................    depth=837, length=20
.........GTCGTCACGGTCGGTCCG...................................    depth=1101, length=18
.......GGGTCGTCACGGTCGGTCCG...................................    depth=369, length=20
...........CGTCACGGTCGGTCCGCC.................................    depth=482, length=18
..........TCGTCACGGTCGGTCCGCC.................................    depth=452, length=19
........GGTCGTCACGGTCGGTCCGCC.................................    depth=3356, length=21
.......GGGTCGTCACGGTCGGTCCGCC.................................    depth=1355, length=22
......TGGGTCGTCACGGTCGGTCCGCC.................................    depth=414, length=23
```

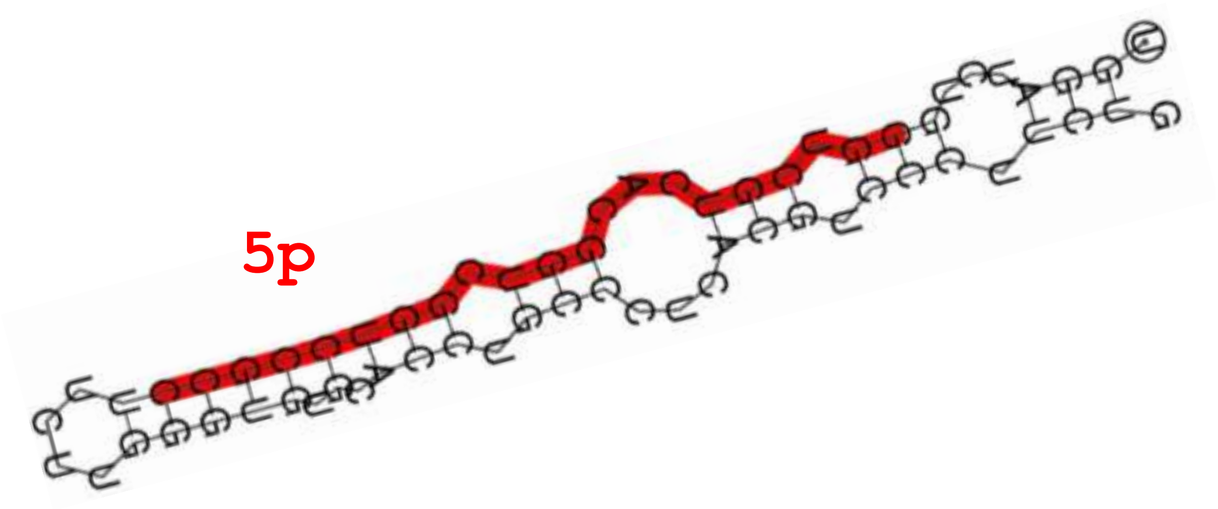

Aang-nmiR016

```
5'->3'
CCTCCGAGGAAATAATGTGAAGAACACATTGACTACAGTTATGTTCTTCACATCCTTTCCTCGGAGA
.(((((((((((..((((((((((((.((((....))))..))))))))))))..))))))))))).
....CGAGGAAATAATGTGAAGAAC.......................................... depth=1048, length=21
.....GAGGAAATAATGTGAAGAACA......................................... depth=701, length=21
......AGGAAATAATGTGAAGAACAC........................................ depth=499, length=21
...........................................TTCTTCACATCCTTTCCTCGG... depth=1338, length=21
...........................................TTCTTCACATCCTTTCCTCGGA.. depth=434, length=22
............................................TCTTCACATCCTTTCCTCGGA.. depth=7684, length=21
...............................................TCACATCCTTTCCTCGGAG. depth=470, length=19
```

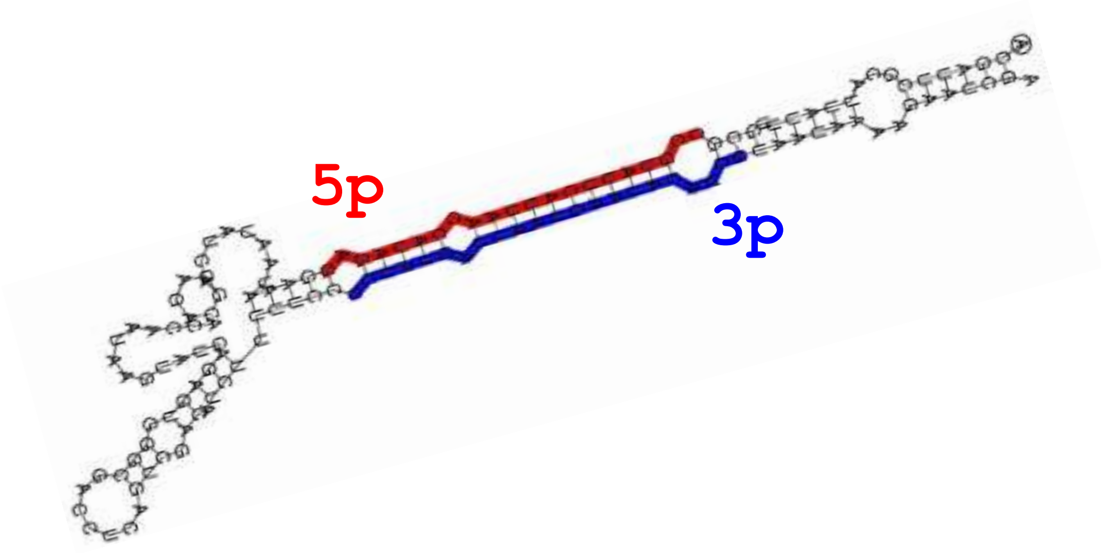

Aang-nmiR017

```
5'->3'
GGGCGTGGGGGCGTTGGACAAAACCGGTGTAAGATTTTAGGGATTTATCTTACAAGAGTTTTGCCAATACCTCCCATGCCT
.((((((((((.(((((.((((((...((((((((...........))))))))...))))))))))).)).)))))))).
...CGTGGGGGCGTTGGACAAAACC........................................................   depth=353, length=22
..........................................................TTTTGCCAATACCTCCCATGCC.   depth=11321, length=22
```

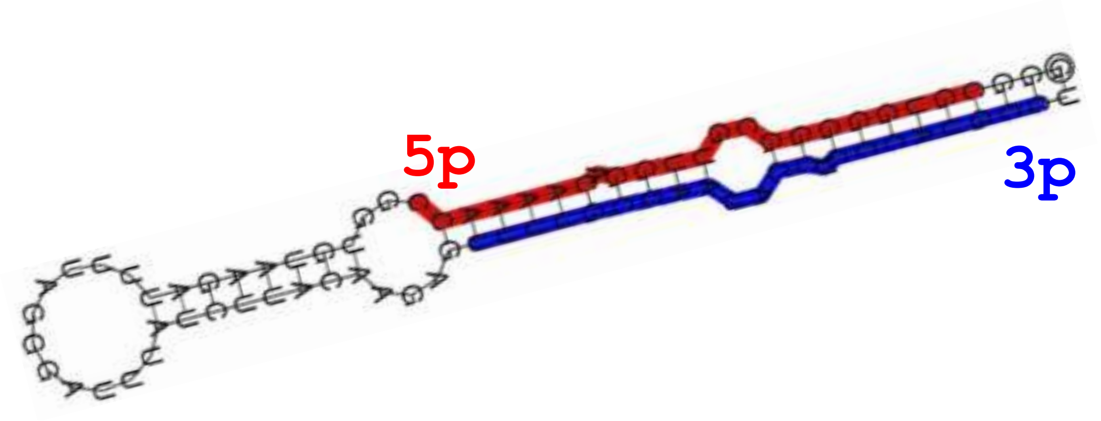

Aang-nmiR018

```
5'->3'
TGCGCCGTATTCATTAACCATAGAGGAAATAATTTCAAGAGACACCAAGCAGTATCAGACTCGGCTGCCTTACTCGACATCTTTTTCCCCTGTGGTTAATGAATACATCGTAA
((((..(((((((((((((((((.(((((.........(((.......(((((.........)))))....))).........))))).)))))))))))))))))..)))).
....CCGTATTCATTAACCATAGAG........................................................................................   depth=328, length=21
....CCGTATTCATTAACCATAGAGG.......................................................................................   depth=65, length=22
........................................................................................CCTGTGGTTAATGAATACATC....   depth=3137, length=21
........................................................................................CCTGTGGTTAATGAATACATCG...   depth=7218, length=22
```

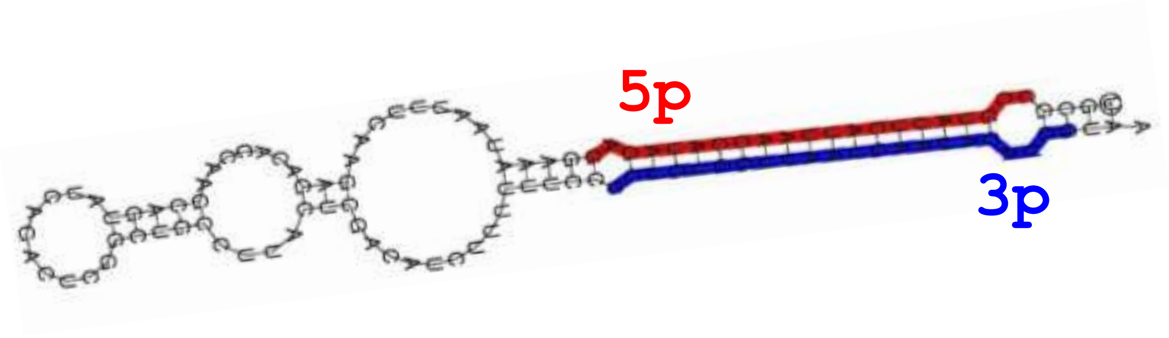

Aang-nmiR019

```
5'->3'
CACCATCGAGGCTTGACGTCAAAAAAAATACAAAGTCCGCTCTTTGATTTTTTTTTACGTCAGGTCCTCTATGGTT
.((((.(((((((((((.((((((((.(((((......))))))))))))))).)))))))).))))).))))).
..CCATCGAGGCTTGACGTCAAAA.....................................................depth=119, length=22
......................................................TTACGTCAGGTCCTCTATGG..depth=1529, length=20
......................................................TTACGTCAGGTCCTCTATGGTT depth=8998, length=22
```

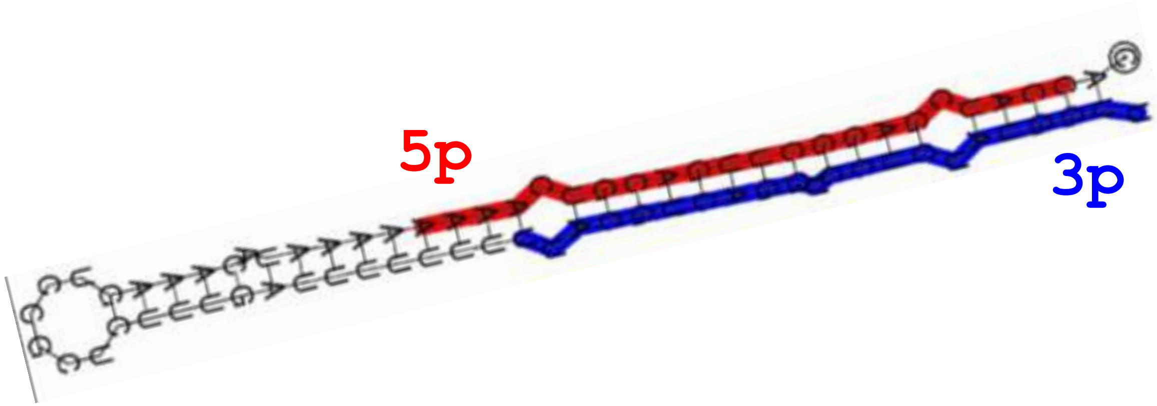

Aang-nmiR020

```
5'->3'
AGGGCTGTCCGTGATTGGGCATAAGTTATCTAGGGTTTATTGATGTTTTTTTTTTTGGTTTAAAAACCTAGATAATTCATACCCAATCACCGACAGCCTT
.((((((((.(((((((((.((.(((((((((((.((((.....................))))..))))))))))).)).))))))))).)))))))).
AGGGCTGTCCGTGATTGGGC................................................................................  depth=8, length=20
AGGGCTGTCCGTGATTGGGCA...............................................................................  depth=15, length=21
............................................................................TCATACCCAATCACCGACAG....  depth=533, length=20
............................................................................TCATACCCAATCACCGACAGC...  depth=3981, length=21
...............................................................................TACCCAATCACCGACAGCCT.  depth=576, length=20
...............................................................................TACCCAATCACCGACAGCCTT  depth=855, length=21
```

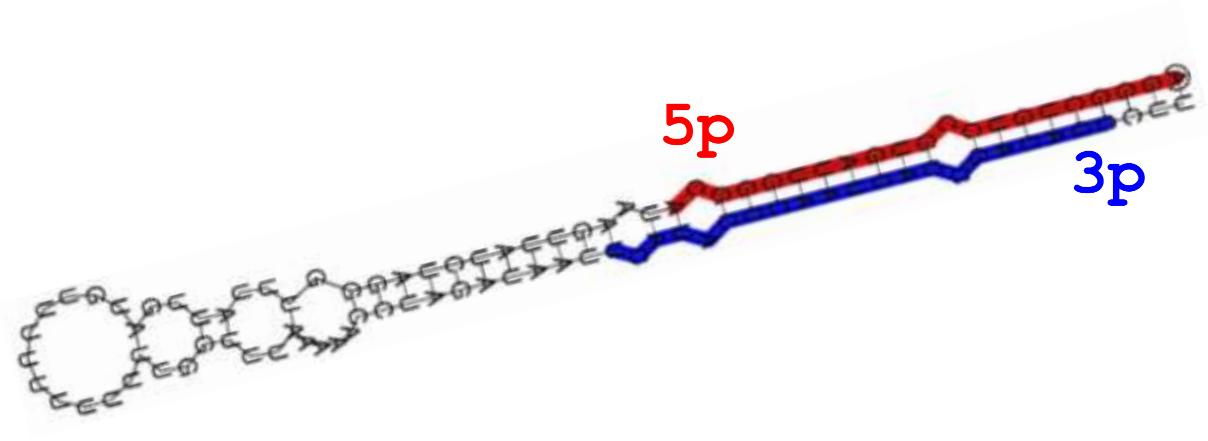

Aang-nmiR021

```
5'->3'
GTGTGAGAGAAGAGTATGGGCGTGGGAGCGTTGGAGAAAGCTGGTGTAAGAGCGGGCATGGGAGTGTTGGAGAAAGCTGGTGTAAGAGGTCGGTGGTTTTCTCTTATAAGAGTTTTTCCAATTCCGCCCATGCCTTTGGTCTTCTTCAC
.((((.((((((.((.(((((((((.(.(((((((((((((.((......)).))).......(((.(((((((((((.((........)).))))))))))).)))....))))))))))..).))))))))).)).)))))))))).
................................................................................................................TTTTTCCAATTCCGCCCA...................
          depth=45, length=18
................................................................................................................TTTTTCCAATTCCGCCCATGC................
          depth=51, length=21
................................................................................................................TTTTTCCAATTCCGCCCATGCC...............
          depth=5945, length=22
.................................................................................................................TTTTCCAATTCCGCCCATGCC...............
          depth=67, length=21
```

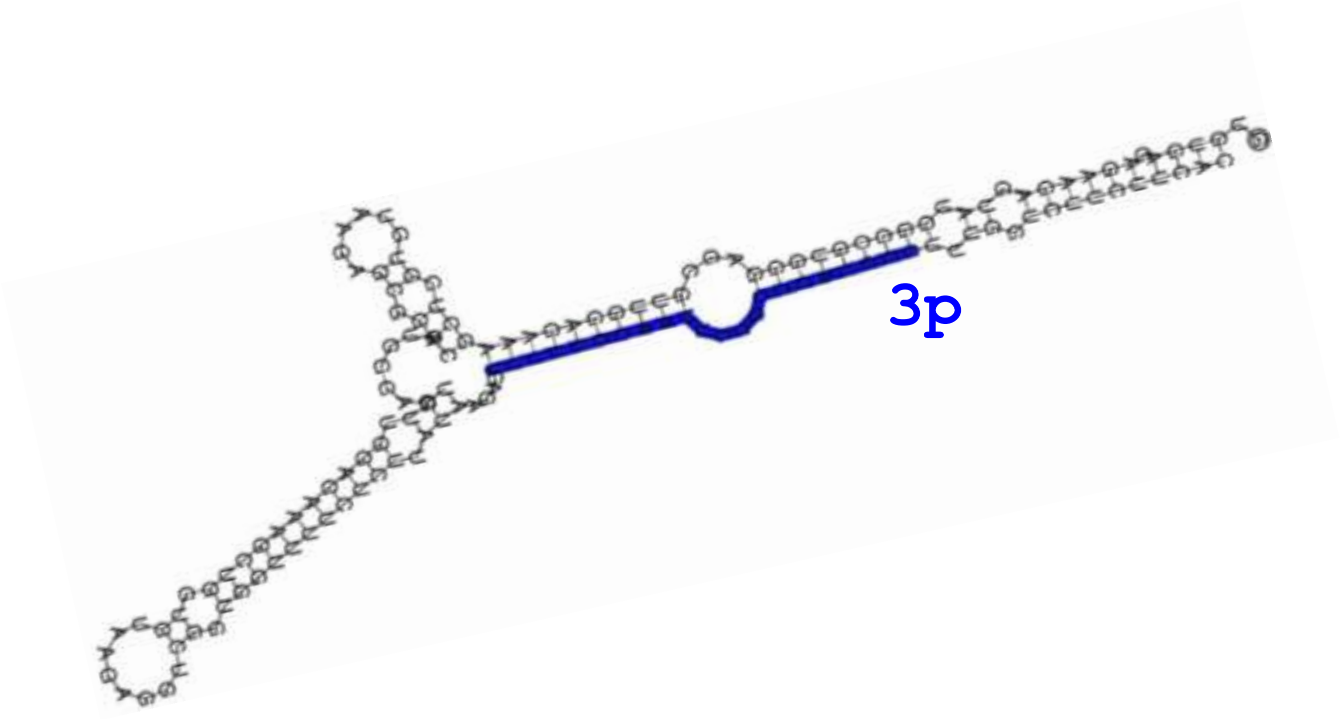

Aang-nmiR022

```
5'->3'
ACGATTCGCATTATTTGCGCCGTATTCATTAACCATAGAGGAAATAATATCAGGAGACACCAATAAGTATCAGAGTGGGCGACCTCAGTCGACATCTTTTTCCCCTATGATTAATGAATACATCGTAATAAAAGAATCGA
.((((((...(((((.(((..(((((((((((.(((((.(((((........((.....))..........(((((.(((.......))).)).))).))))).))))).)))))))))))..))))))))..)))))).
...................CCGTATTCATTAACCATAGAG....................................................................................................
        depth=328, length=21
...................CCGTATTCATTAACCATAGAGG...................................................................................................
        depth=65, length=22
.......................................................................................................CCTATGATTAATGAATACATC................
        depth=239, length=21
.......................................................................................................CCTATGATTAATGAATACATCG...............
        depth=4168, length=22
```

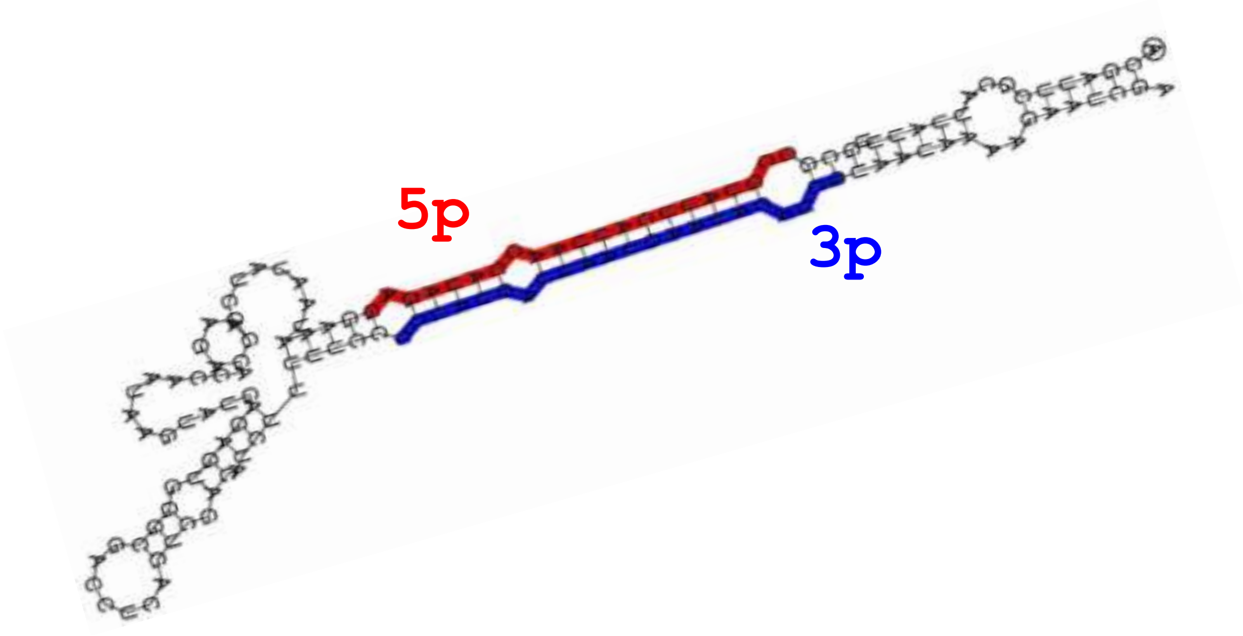

Aang-nmiR023

5'->3'

CTAGGGCACTCGTGACTGTCGTGGATGTATATCAGCGAACTCGGCCAATTAAGAGTTCGTCCGGCTCATGTACATGCACGACGGTCACGACTGCCCTAT

.(((((((.((((((((((((((.((((((((.(((((((((..........)))))).....))).)))))))).)))))))))))))).))))))).

............TGACTGTCGTGGATGTATATC.. depth=4464, length=21

....TGTACATGCACGACGGTCACG.......... depth=6, length=21

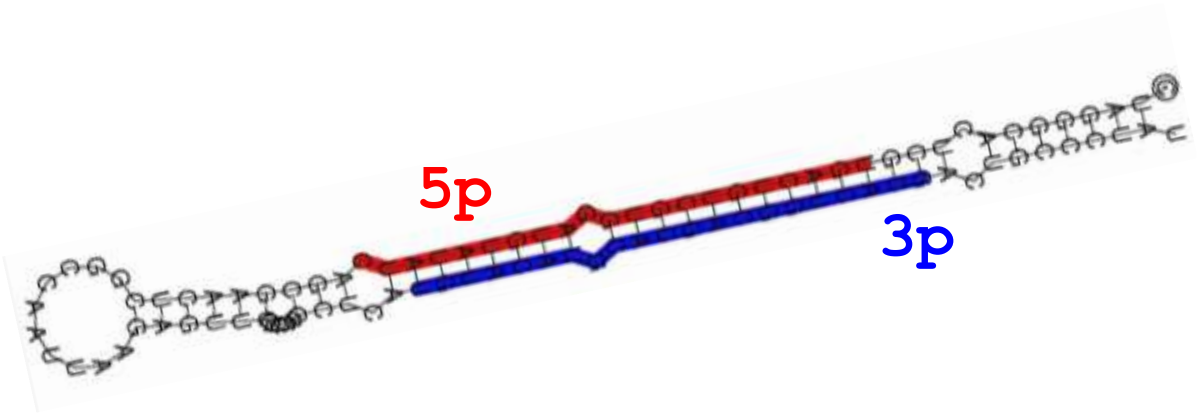

Aang-nmiR024

```
5'->3'
AGAGCACGGTGCCCAGCCAAGAATGATTTGCCCGCCAATTAGATATATCTATATATCTATATCTATAAATTATAGTGGGCAGGTCATTCTTGGTGCTGCATCTTGCTCG
.(((((.(((((...((((((((((((((((((((.(((((((((((..........))))))))...)))...))))))))))))))))))))...))))).))))).
.............CAGCCAAGAATGATTTGCCCG........................................................................... depth=245, length=21
.............CAGCCAAGAATGATTTGCCCGC.......................................................................... depth=384, length=22
.............CAGCCAAGAATGATTTGCCCGCC......................................................................... depth=565, length=23
.............................................................................GGCAGGTCATTCTTGGTGCT............ depth=1089, length=20
...........................................................................TGGGCAGGTCATTCTTGGTGCT............ depth=262, length=22
..............................................................................GCAGGTCATTCTTGGTGCTG........... depth=478, length=20
```

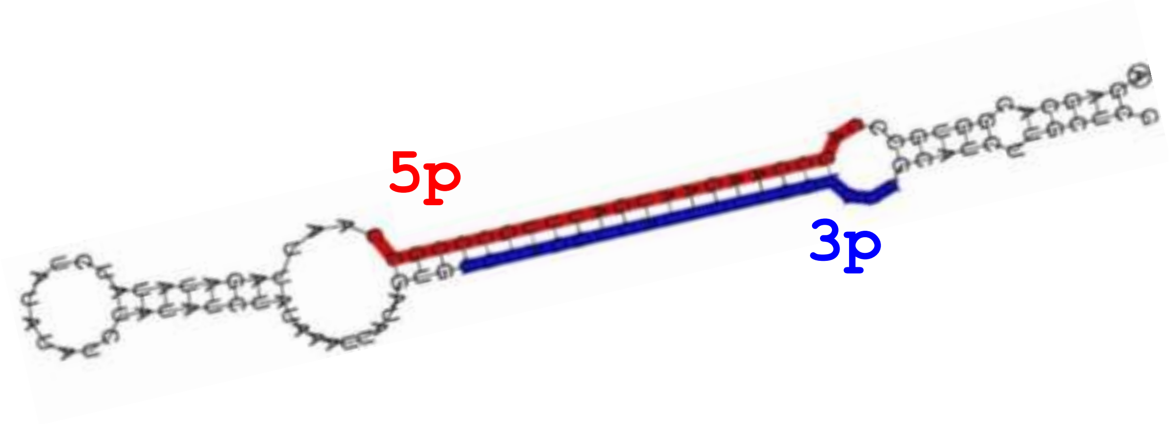

Aang-nmiR025

5'->3'

CAGCCGCATCAGGTCTCCAAGGTGAACAGCCTCTTGGCCATAGAAAAATTGTAGGTTATGGGGAAGTCGGAAAGCAGATCCGTAAACTTCGGAGAAGAAGGGATTTGGCAGATCCGTAACTTCGGGAAAAGGTTGGCTCTTAAGGGTTGGGCGCGTTGGG
CCTTTGGTGGACGGCTC
.(((((((((((..((((.(((..(((((.(((((((........(((.(((((((((....(((......((((((.(...((((...)))).).)))))))...))))))))))))))))........)))))...))))))))..))).)))))
..))))))))..))))).
...CCGCATCAGGTCTCCAAGGTG...
....   depth=3447, length=21

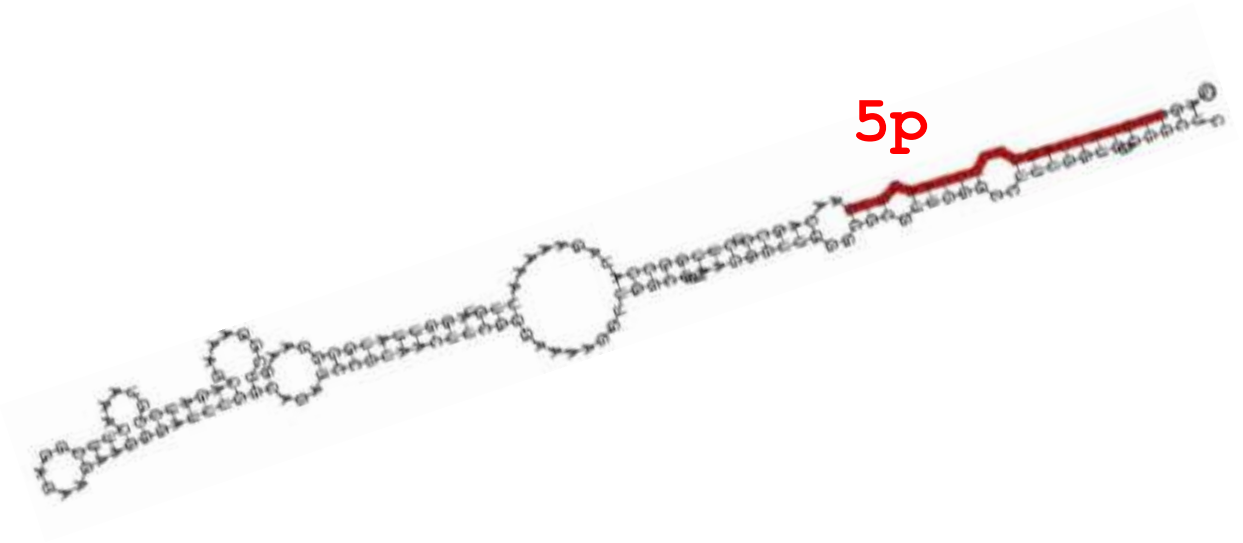

Aang-nmiR026

```
5'->3'
TTGTGGATAGGAGGAGGATTCATGTGAATATTTGTTCCGAAAATTTCTTGAACGAGATTGTTCATATAAATCCTTCTGCTGTCCATAT
.(((((((((.(((((((((.(((((((((((((((.(((...)))..)))))))..))))))))).)))))))))).))))))))).
...TGGATAGGAGGAGGATTCATG.................................................................      depth=1, length=21
...................................................................TAAATCCTTCTGCTGTCCA...      depth=210, length=19
...................................................................TAAATCCTTCTGCTGTCCATA.      depth=3062, length=21
```

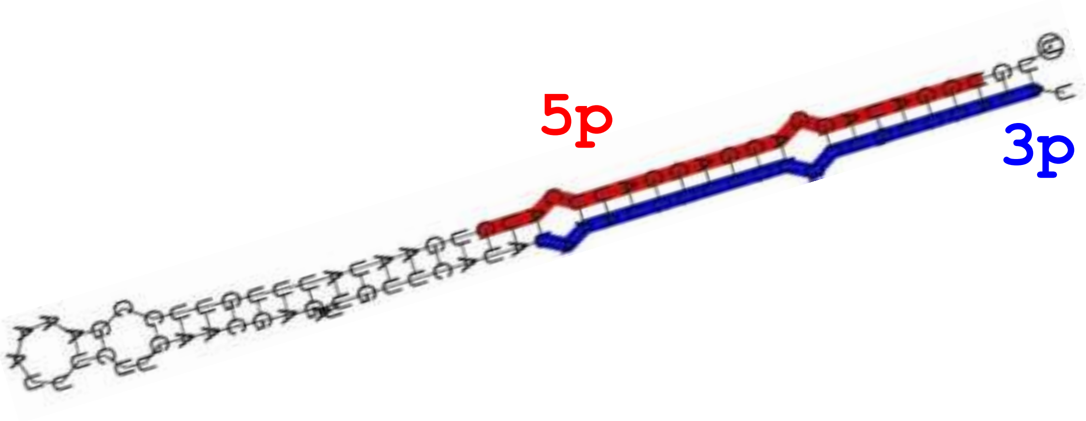

Aang-nmiR027

```
5'->3'
TACCACCGTGGACCTGGTGTGAAAAAAATACATTGTCTGCTCTTTGATTTTTTTCACGTCAGGACCTCGGTGGTT
.(((((((.((.(((((((((((((((((.((..(......)..))))))))))))))))))).)).))))))).
..CCACCGTGGACCTGGTGTGAA....................................................  depth=10, length=21
.....................................................TCACGTCAGGACCTCGGTGGTT  depth=1810, length=22
......................................................CACGTCAGGACCTCGGTGGTT  depth=640, length=21
```

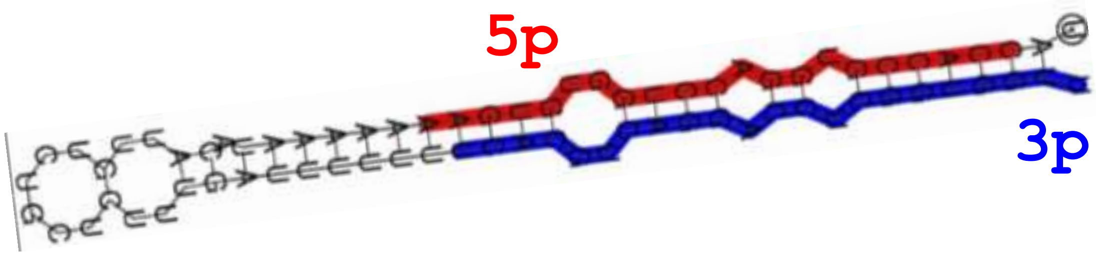

Aang-nmiR028

5'->3'
AGGGAGTCCGGAGACGTCGGCGGGGGCCTCGGGAAGAGTTATCTTTTCTGTTTAACAGCCTGCCCACCCTGGAAACGGCTCAGCCGACGTCGGCGGAGCCCG
.(((..((((..(((((((((..(((((..(((((((....))))))).((((..(((..........))).))))))))).)))))))))..)))).))).
......TCCGGAGACGTCGGCGGGGG.. depth=190, length=20
.......CCGGAGACGTCGGCGGGGGC... depth=565, length=20
......TCCGGAGACGTCGGCGGGGGC... depth=1114, length=21
.......CCGGAGACGTCGGCGGGGGCC.. depth=199, length=21

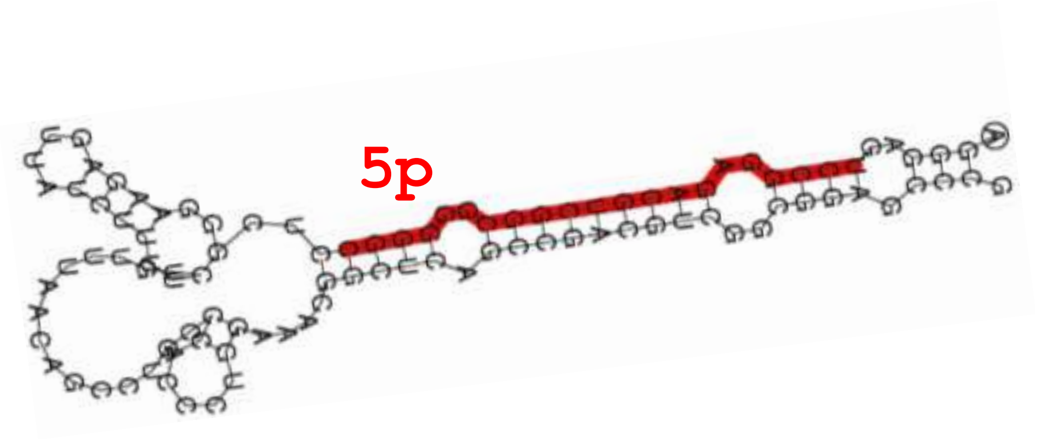

Aang-nmiR029

```
5'->3'
GGAAGGAAATGTGTTAGAAAACCATTGACTATCAAAAGAGAGGCAGCCAGCAGCAGCGGACAACAGCTCTGAGATGTTTTTTGCTTCTGTTTTGATAGCCAGTGGCAATCTTGCACATTTTGTTCA
.(((.(((((((((.(((...((((((.(((((((((.((((((((..((((....(((((....).))))...))))..)))))))))).))))))))).))))))...))).))))))))).))).
.................AAAACCATTGACTATCAAAAG....................................................................................... depth=11, length=21
.................AAAACCATTGACTATCAAAAGA...................................................................................... depth=47, length=22
.........................................................................................TTTTGATAGCCAGTGGCAATC................ depth=1364, length=21
.........................................................................................TTTTGATAGCCAGTGGCAATCT............... depth=296, length=22
```

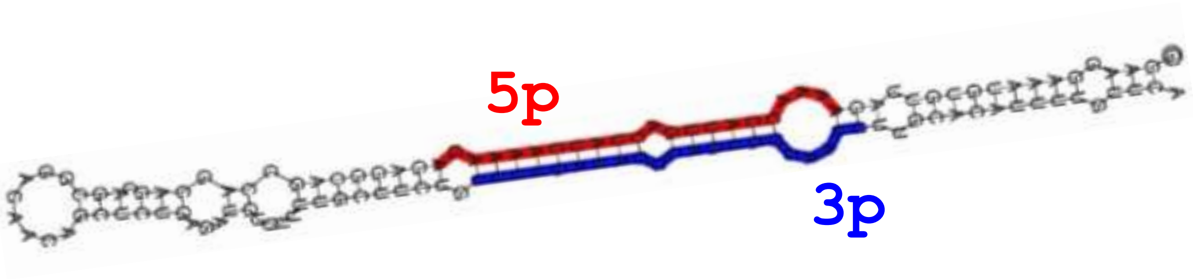

Aang-nmiR030

```
5'->3'
CCCGGCGGCCGTGGTTCACAAACGCCTGCGCCCTCGCGGCGGGCCGCGCCCCTCGCGGCGGGGCCCTTCGGGGCACCCTTGCGGCTCCGTGGCGCTGGCCGGCGGGCTTTCCATCGACGGTCTTGGA
.((((.((((((.(.....(((.((((((((((..(((.((((((((((((......)))(((((((....)))).)))..))))).)))).)))..)))..)))))))))....).)))))))))).
..........................TGCGCCCTCGCGGCGGGC...................................................................................    depth=74, length=18
...........................GCGCCCTCGCGGCGGGCC..................................................................................    depth=32, length=18
.......................................................................................CGTGGCGCTGGCCGGCGGGC....................    depth=128, length=20
..........................................................................................GGCGCTGGCCGGCGGGCTT..................    depth=305, length=19
..........................................................................................GGCGCTGGCCGGCGGGCTTTC................    depth=116, length=21
...........................................................................................GCGCTGGCCGGCGGGCTTT.................    depth=177, length=19
...........................................................................................GCGCTGGCCGGCGGGCTTTC................    depth=454, length=20
```

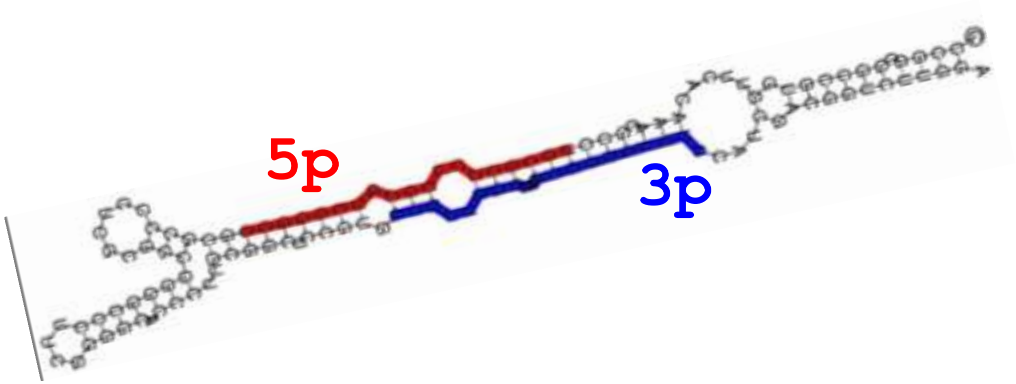

Aang-nmiR031

```
5'->3'
AGGCATCTTGACCTCGCCAACAATCTCAGCGACGATGATGGAGGCTTAAGTCTTGTATGATATATATACACATTGTATATGTAACGTGATGAGCTGAGATTGTTGGAGAGGTTCGAGATGCCC
.((((((((((((((.((((((((((((((....((.((((((((....)))))......((((((((((...)))))))))).))).))..)))))))))))))).)))).)))))))))).
..........ACCTCGCCAACAATCTCAGC.............................................................................................  depth=110, length=20
..............................................................................................TGAGATTGTTGGAGAGGTTC.........  depth=744, length=20
..............................................................................................TGAGATTGTTGGAGAGGTTCG........  depth=847, length=21
```

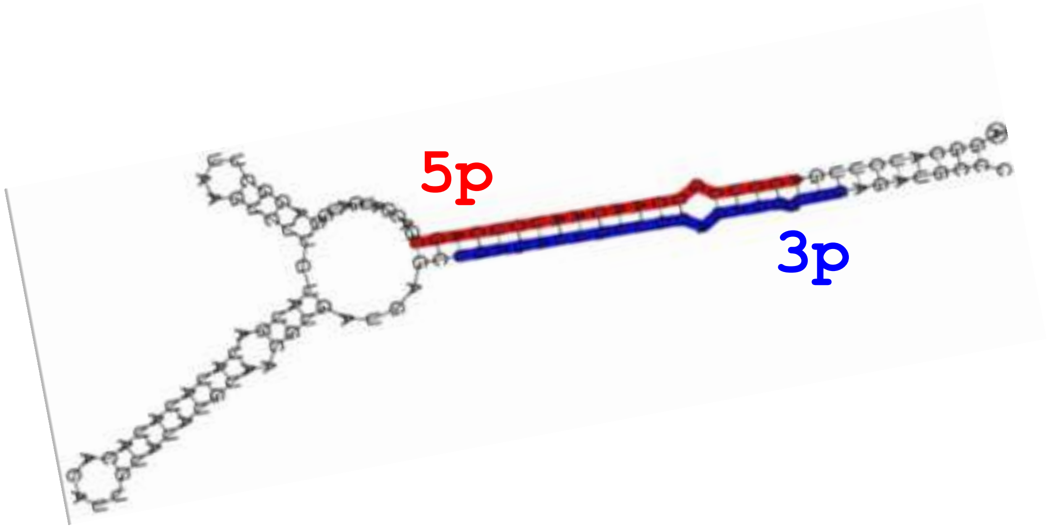

Aang-nmiR032

5'->3'
CAGGAGAAGAGAGAAAGCACATCCCCTGCCTGATAACTGAAAACTATGGCTATAGTTTGAACTCTAGATTTTTCGGCACAGGTTGTGCTCTCTCTCTTCTTCTA
.((((((((((((.((((((.((..((((.((.((((((((((((((....))))))))....)).)).)).)).))))..)).)))))).)))))))))))).
....AGAAGAGAGAAAGCACATCCC.. depth=814, length=21
....GTTGTGCTCTCTCTCTTCTTC.. depth=383, length=21

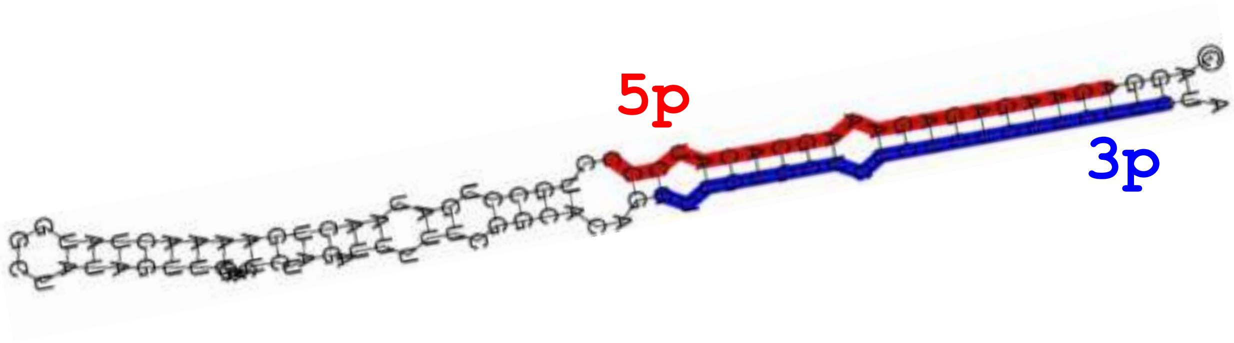

Aang-nmiR033

```
5'->3'
GGAGGTTTTACCACGCCCGCCCATGCCTATTATATTCTTAAGAAAATTTCCATACTTTTCGCTGCCATGGCCGTTAGAACCGGCGACTCGAGCAAGAACACTTGGACGCTTCTTATTGTTCTTGTTTGTGTTCTTCTTAACA
ATATGGTAGGCGTTGCCGGTCTGGTAAAACTTTA
.((((((((((((.(((.((..((((((((((((((.(((((((.........................((((.......))))(((.((((((((((((...(((....)))...)))))))))))).))).))))))).)
))))))))))))).)).))).)))))))))))).
.......TTACCACGCCCGCCCATGCC.......................................................................................................................
..................................          depth=48, length=20
.......TTACCACGCCCGCCCATGCCTA.................................................................................................................
.................. ................          depth=379, length=22
..............................................................................................................................................
........GGCGTTGCCGGTCTGGTAAA......          depth=183, length=20
..............................................................................................................................................
........GGCGTTGCCGGTCTGGTAAAA.....          depth=571, length=21
```

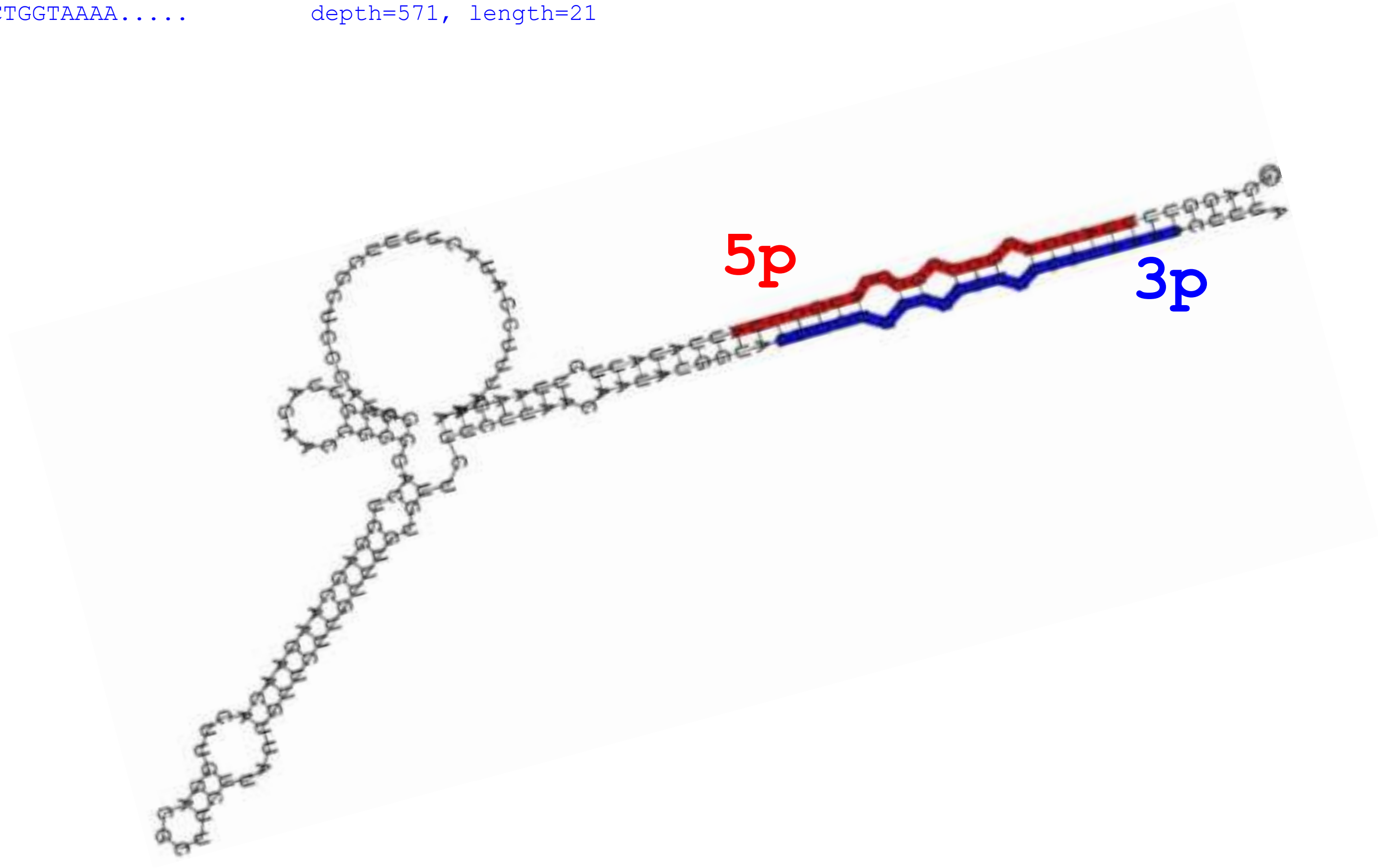

Aang-nmiR034

```
5'->3'
AGTCTGGTCCTATTCCGTTGGCCTGGGAATTAGCATGGAATAACGTGATAGGAGTCTGGCC
.(((.(((((((((.(((((.((((........)).))..))))).))))))).)).))).
..TCTGGTCCTATTCCGTTGGCCT..................................... depth=126, length=22
.....GGTCCTATTCCGTTGGCC...................................... depth=234, length=18
......GTCCTATTCCGTTGGCCT..................................... depth=563, length=18
.................................CATGGAATAACGTGATAGGAGTC..... depth=8, length=23
..................................ATGGAATAACGTGATAGGAGTCTG... depth=6, length=24
.....................................GAATAACGTGATAGGAGTCTG... depth=12, length=21
......................................AATAACGTGATAGGAGTCTG... depth=7, length=20
```

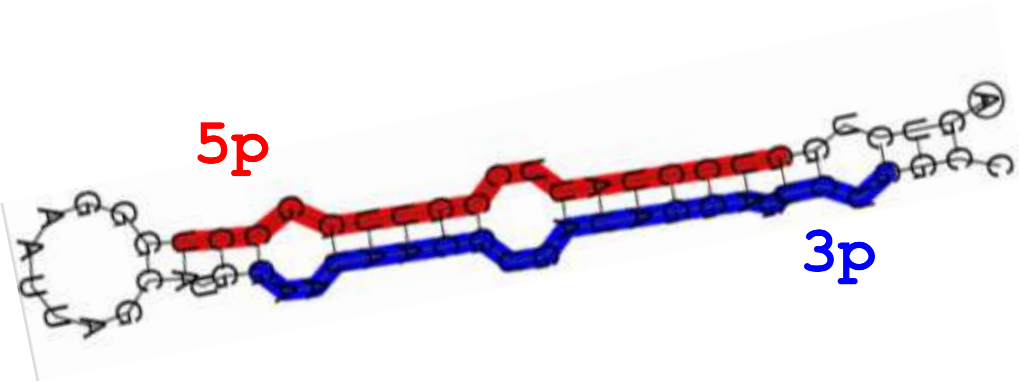

Aang-nmiR035

```
5'->3'
ATTTCGTTCCCATGCTTGTTATCTCTGTGCGGCGTCTCGATGAGCTCTTTTTTGAACAAAATGGTGGGCCGCAAATCCCCACTGAAGGGAGGGATGCCGCGCAGAAATAAAAGCATGGGAACGAAAA
.((((((((((((((((.((((.((((((((((((((.......((((((((..........((((((.........)))))))))))))))))))))))))))).)))))))))))))))))))).
........CCCATGCTTGTTATCTCTGTGC.................................................................................................  depth=154, length=22
.........CCATGCTTGTTATCTCTGTGCG................................................................................................  depth=124, length=22
........CCCATGCTTGTTATCTCTGTGCG................................................................................................  depth=115, length=23
...........ATGCTTGTTATCTCTGTGCGGC..............................................................................................  depth=548, length=22
................................................................................................CCGCGCAGAAATAAAAGCATG..........  depth=19, length=21
...................................................................................................CGCAGAAATAAAAGCATGGGA.......  depth=6, length=21
```

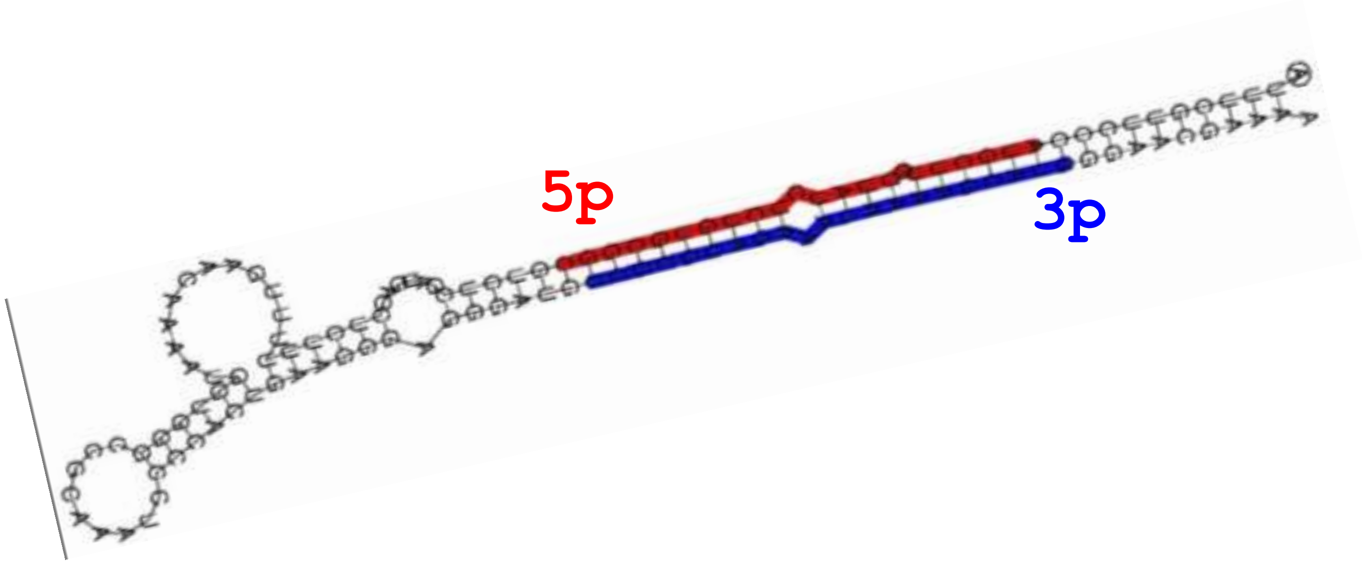

Aang-nmiR036

```
5'->3'
AATTCGTTTTTGATGGAGCCTTGTTCCTATTTACTGGCAACCCTGAAGGGAGTAAGATTTCTCGGAACGGATTTTCAATAAATAGGAACACAGGTTTTATCACAGACGAATA
.((((((((.(((((((((((((((((((((((.(((.((((.....(((((......))))).....)).)).))).))))))))))))).))))))))))).)))))))).
..................CCTTGTTCCTATTTACTGGCA.........................................................................     depth=932, length=21
..........................................................................TCAATAAATAGGAACACAGGTT................     depth=133, length=22
```

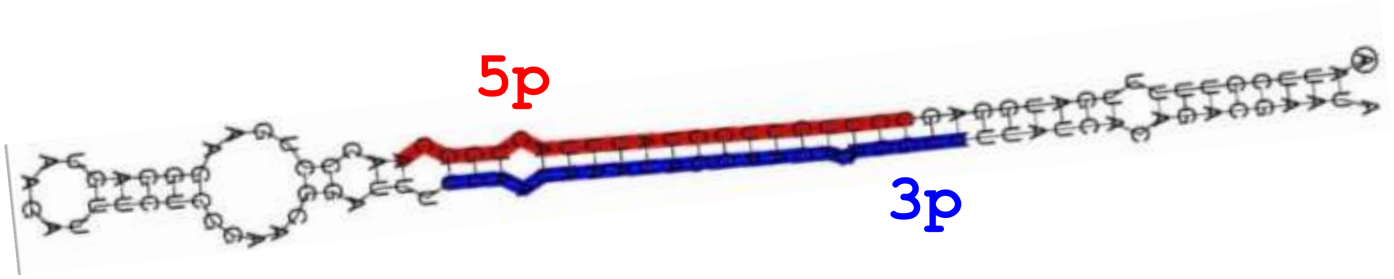

Aang-nmiR037

```
5'->3'
TGATAAATTAGTCAACTCAAGTCTTTGAAATATATTGGAAGGGTATGGGCTAGGATGGGCCATAGAATCTCCTATGTATCCATCTGTATTTTTTTAAAGATTTGAGTTGTCCAATCTATCAA
(((((.(((.(.((((((((((((((((((.....((((.((((((.((((......))))((((......)))))))))).)))).....))))))))))))))))))).).))).))))))).
.......TTAGTCAACTCAAGTCTTTGA..............................................................................................  depth=9, length=21
.........AGTCAACTCAAGTCTTTGAAA............................................................................................  depth=14, length=21
.............................................................................................TTAAAGATTTGAGTTGTCCAA........  depth=666, length=21
```

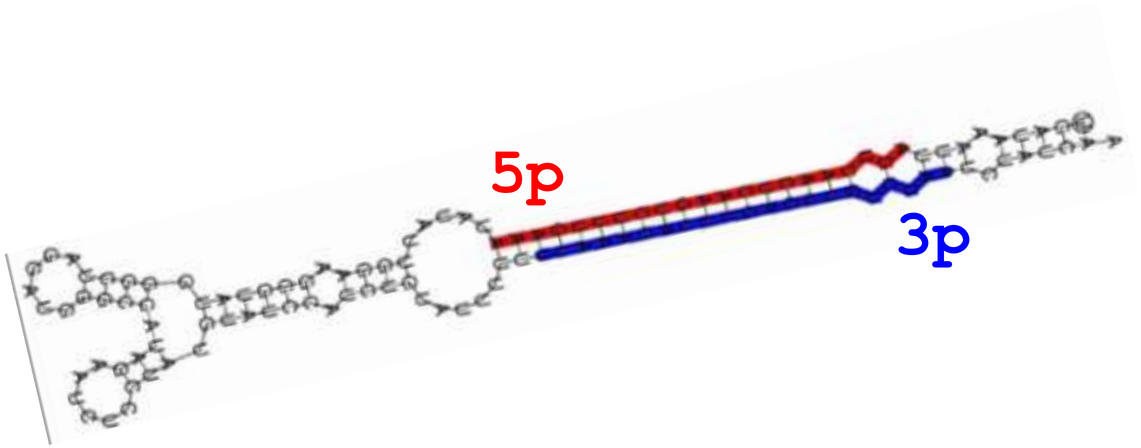

Aang-nmiR038

```
5'->3'
TGGGAGTGGGAGGAACGGGCAAAAACTTGTCTGAGACATAGTTTTCCCGGCTCCTCCCATTCCTC
.(((((((((((((.((((..((((((((((...)))).))))))))))..))))))))))))).
....AGTGGGAGGAACGGGCAAAAAC.......................................    depth=51, length=22
....AGTGGGAGGAACGGGCAAAAACT......................................    depth=96, length=23
.....................................TTTTCCCGGCTCCTCCCATTCC..    depth=662, length=22
```

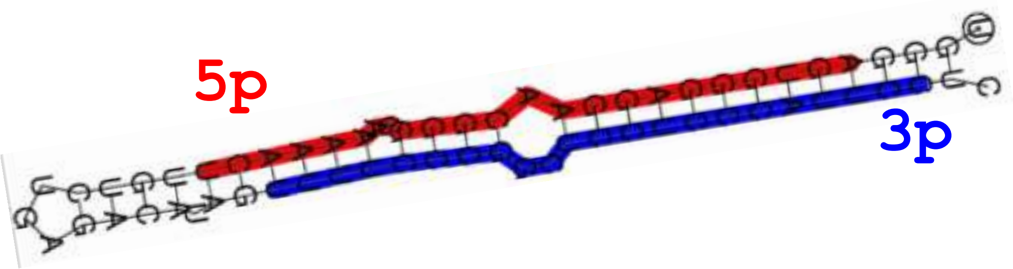

Aang-nmiR039

```
5'->3'
CGGATAAATTGGACAACTCAATCTTTGAAATATCGCAAAGAGGCATGGAGTCTAGGGGGAATGATTCTCTCTTTTAAGATATCTCAAGGACTTGAGCTGTCCAATTTATCTA
.((((((((((((((.((((((((((((.(((((..((((((....((((((..........))))))))))))...))))).))))))).))))).)))))))))))))).
.......ATTGGACAACTCAATCTTTGA....................................................................................    depth=260, length=21
......AATTGGACAACTCAATCTTTGA....................................................................................    depth=189, length=22
..................................................................................CTCAAGGACTTGAGCTGTCCAA........    depth=113, length=22
...................................................................................TCAAGGACTTGAGCTGTCCAAT.......    depth=22, length=22
```

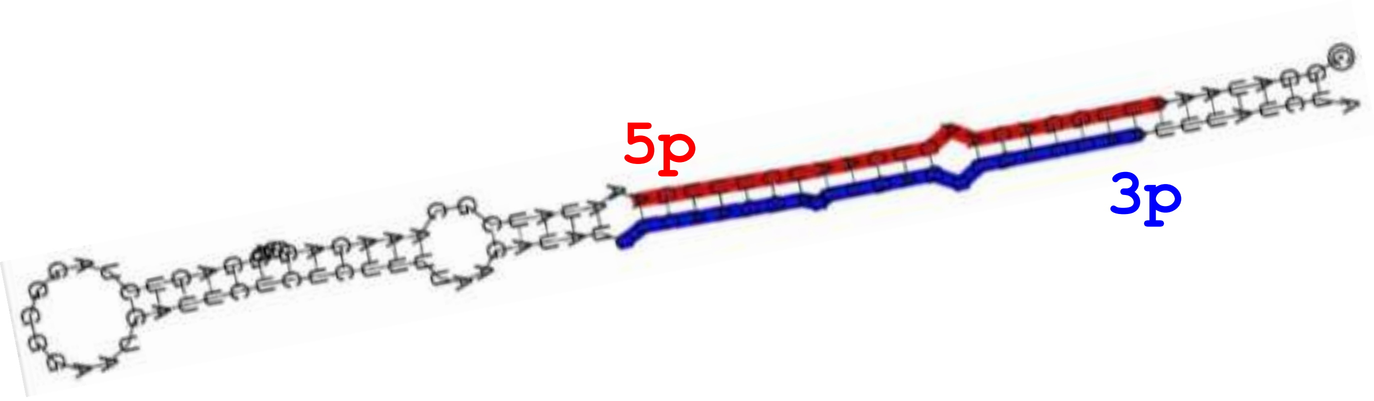

Aang-nmiR040

5'->3'

```
AAGGGCACCGCATACAGCAAGTGGAAAACTAGAATAAGCGTGATAAACACCTCGTGTCATTTTTTGTCTTTCACGAACGGAAAGGTGTTTATCAGGGGTATTTCAGTTCTTCACTTGCTAAATGCCGTGACTCTC
.(((((((.((((..((((((((((.((((.(((((..(.((((((((((((((((...............))))).......))))))))))).)..))))).)))).))))))))))..)))).))).)))).
..............CAGCAAGTGGAAAACTAGAAT....................................................................................................   depth=11, length=21
.................CAAGTGGAAAACTAGAATAAGC................................................................................................   depth=8, length=22
..................................................................................................TATTTCAGTTCTTCACTTGC.................   depth=72, length=20
..................................................................................................TATTTCAGTTCTTCACTTGCT................   depth=506, length=21
```

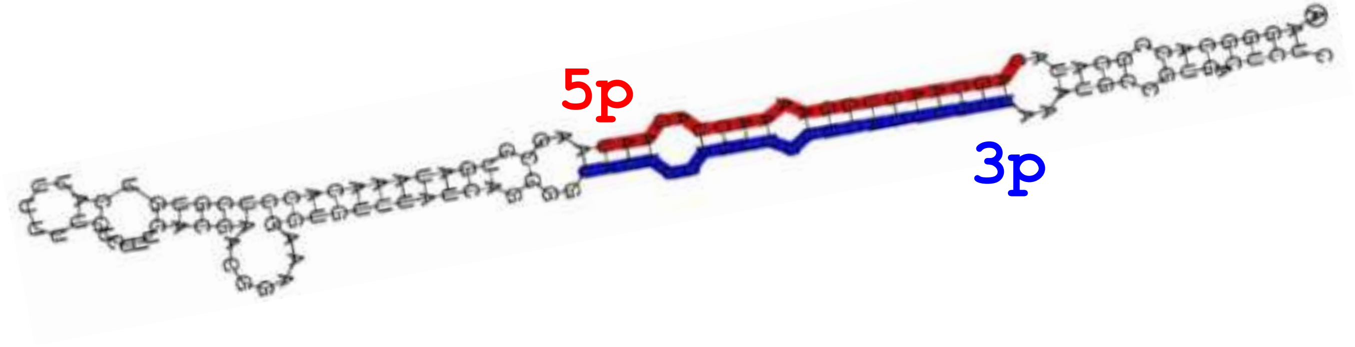

Aang-nmiR041

```
5'->3'
TTTGTTGTGGGCTTACATGTCTGTCGATGTTGTAGCTAGCTCTGGCTCATTCCATTTATAGCTACACTATCAGCAGATATGTCAGCCAACCACCACCACTCATATCACAGCAAT
.((((((((((((.((((((((((.((((.((((((((....(((......)))....)))))))).)))).)))))))))).))))..................)))))))).
........GGGCTTACATGTCTGTCGATG.....................................................................................     depth=14, length=21
.......TGGGCTTACATGTCTGTCGATG.....................................................................................     depth=141, length=22
.....................................................................TCAGCAGATATGTCAGCCAAC........................     depth=31, length=21
.....................................................................TCAGCAGATATGTCAGCCAACC.......................     depth=379, length=22
```

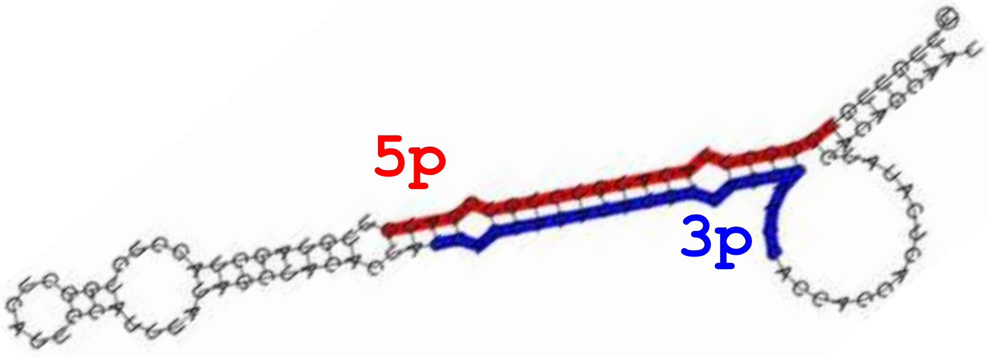

Aang-nmiR042

5'->3'

```
AATCTGCATCCACAATGCACATTTTTAGTCTGAAACTGCATATATATAGTAAAAATGTGCATTGCATATCTGAATTTGCATCTACAATTCACATCTTTAGTATGCAGTTTCAGACTAAAGATGTGTATTGTGATGCAGATT
.(((((((((.((((((((((((((((((((((((((((((((......((((.(((((.((((....................)))).))))).))))))))))))))))))))))))))))))))))))))))))))).
.................CACATTTTTAGTCTGAAACTG....................................................................................................... depth=317, length=21
............................................................................................................TTCAGACTAAAGATGTGTATT............ depth=235, length=21
```

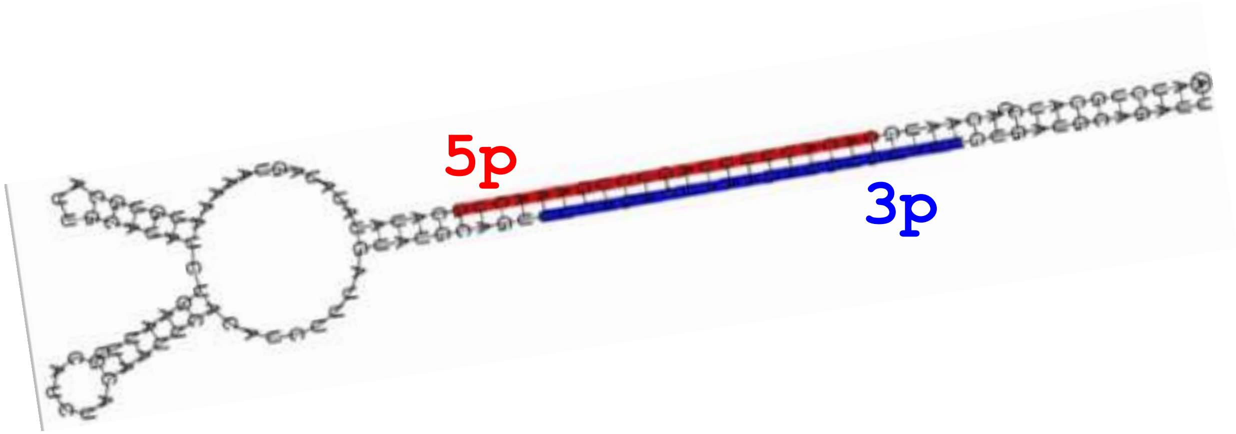

Aang-nmiR043

```
5'->3'
ACCTCCGAATTCCGCGACGCTCCACCATAACATGGTGGTCATCAAAGCCATTTATCGTGGACCGTCGCTGAATTCGGAGGG
.(((((((((((.((((((.(((((.((((.(((((..........))))))))).))))).)))))).))))))))))).
...TCCGAATTCCGCGACGCTCCA.........................................................   depth=255, length=21
........................................................GTGGACCGTCGCTGAATTCGG....   depth=60, length=21
.........................................................GGACCGTCGCTGAATTCGGAG..   depth=143, length=21
```

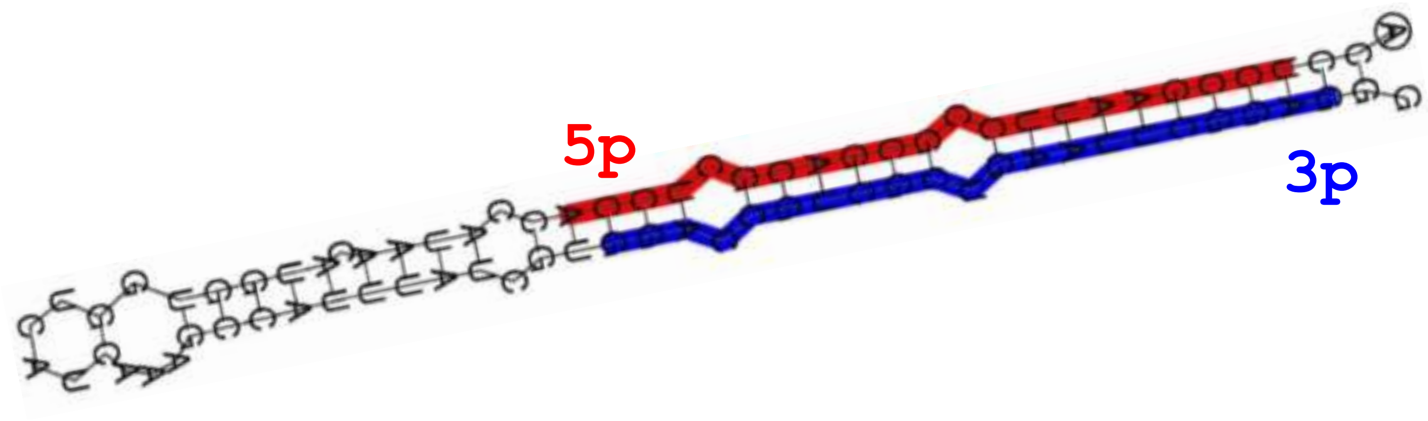

Aang-nmiR044

```
5'->3'
TTTTGCTGTCCATCAAAGAAGGCATATGCACAAAACAATTCCCAACTATCTACTTCCAACAAATGCTTCATCTGGTATATGCGGGCATTCATGGACCAGCAAAT
.((((((((((((.((.(...((((((((.((.....................................)))))))))))...).)).))))).))))))).
TTTTGCTGTCCATCAAAGAAG....................................................................................   depth=36, length=21
TTTTGCTGTCCATCAAAGAAGG...................................................................................   depth=23, length=22
.TTTGCTGTCCATCAAAGAAGG...................................................................................   depth=73, length=21
..TTGCTGTCCATCAAAGAAGGC..................................................................................   depth=377, length=21
```

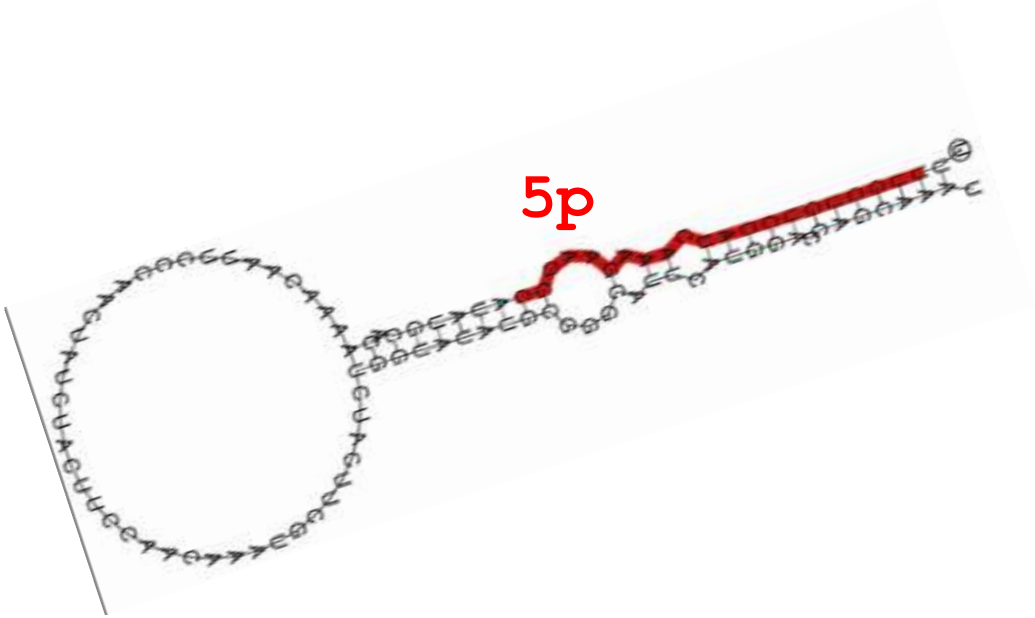

Aang-nmiR045

5'->3'

```
TGACAGTGGAAGGTTGTGTCACATTTTTCTGTGAACAAAATTTCAAACTCGTGAGATAAGAAATTTTTCAACTTTGTAAATGTCATCTCACGAGTTTGAAATTTTGGTCATAGAGAAATATTACACAGTCTTTTCCACTGTCA
.((((((((((((((((((...((((((((((((.((((((((((((((((((((((..((.((((..((....)).)))).)))))))))))))))))))))))))).))))))))))))...)))))).).))))))))))).
........................TTTTCTGTGAACAAAATTTCA..................................................................................................depth=91,length=21
.........................TTTCTGTGAACAAAATTTC...................................................................................................depth=18,length=19
.........................TTTCTGTGAACAAAATTTCA..................................................................................................depth=72,length=20
.........................TTTCTGTGAACAAAATTTCAA.................................................................................................depth=112, length=21
..............................................................................................TTTGAAATTTTGGTCATAGAG............................depth=38, length=21
...............................................................................................TTGAAATTTTGGTCATAGAGA...........................depth=20, length=21
```

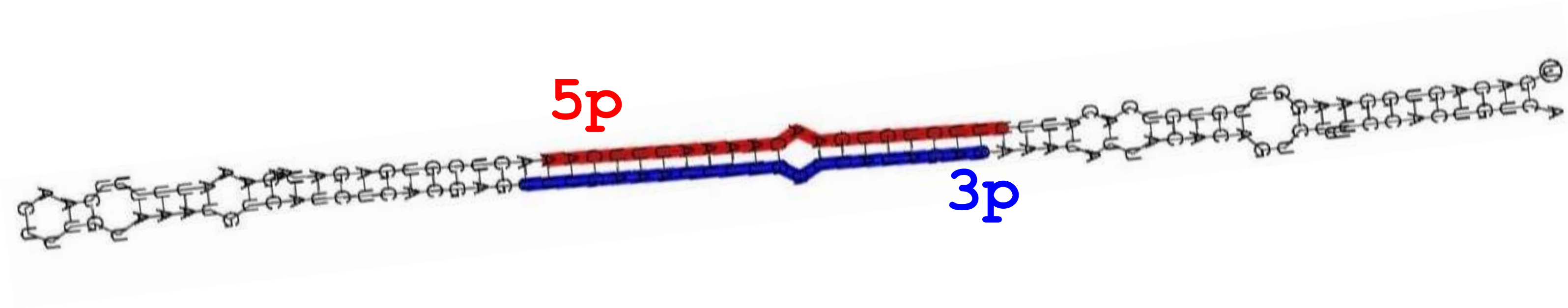

Aang-nmiR046

```
5'->3'
CAGGAGTGGGATGCGAGGATAAGACTAAGATAGCTTTCCGATAGCTGGATAGAGATTGATAGTCTTTCCTACGCCTCCCATTCCTC
.((((((((((.(((((((.(((((((.(((..((.((((.....)))).))..)))..))))))))))).))).)))))))))).
....AGTGGGATGCGAGGATAAGACT...........................................................   depth=259, length=22
...............................................................TCTTTCCTACGCCTCCCATTCC..   depth=172, length=22
```

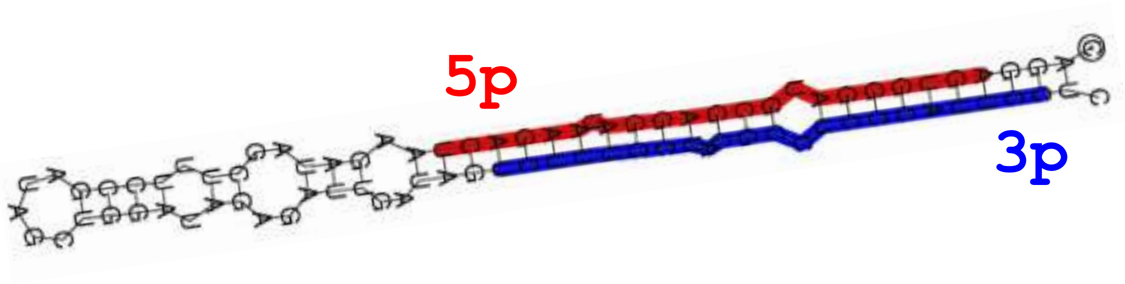

Aang-nmiR047

```
5'->3'
TTAGATTGATCAGAATTGAAAAACTTGCCTATATTATTAGAATCCCCTCTGAAAAATAAAAGCTTCAATTCAACAAACTAATGAATCCCCTCTAACAATATAGGCATGTTTTTCAATTCTGATAAATCTAT
.((((((.((((((((((((((((.((((((((((.(((((........((((..........))))(((((.........)))))....))))).)))))))))).)))))))))))))))).)))))).
.........TCAGAATTGAAAAACTTGCCT.....................................................................................................   depth=26, length=21
...........AGAATTGAAAAACTTGCCTAT...................................................................................................   depth=126, length=21
....................................................................................................TAGGCATGTTTTTCAATTCTG..........   depth=24, length=21
.....................................................................................................AGGCATGTTTTTCAATTCTGA.........   depth=40, length=21
```

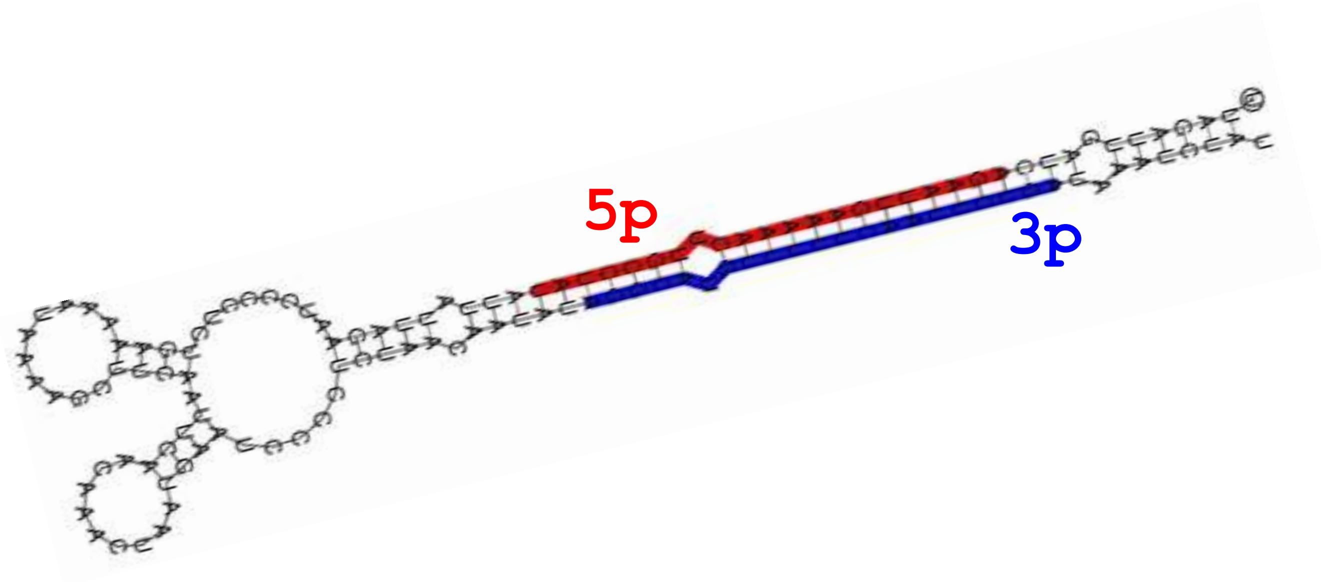

Aang-nmiR048

```
5'->3'
CCATTGAGCACTTGTTGTCAAAAAAATTCAATGTCCGCTTTGATTTTTTTGACATCAGGCCCTCGATGA
.(((((((..((((.((((((((((((.(((........))))))))))))))).))))..))))))).
CCATTGAGCACTTGTTGTCAA................................................        depth=9, length=21
..........................................ATTTTTTTGACATCAGGCCCT......        depth=42, length=21
...........................................TTTTTTTGACATCAGGCCCTC.....        depth=206, length=21
............................................TTTTTTGACATCAGGCCCTCG....        depth=52, length=21
```

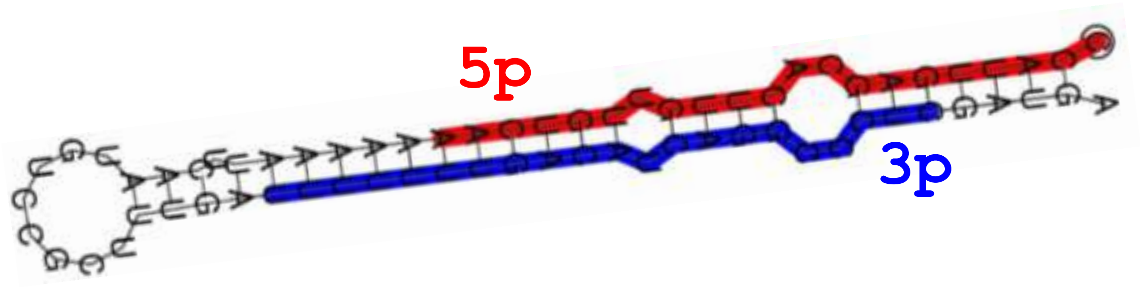

Aang-nmiR049

```
5'->3'
TTCTTGTGATGATAAGGCCCTAATGACACAATCTGTTAATCATTATATGGATTATCAGATGGTGTTATTTGGGCTTGTCATTGCAAGAC
.((((((((((((((.(((.((((((((.(((((.(((((........))))).))))).)))))))).))))))))))))))))))).
.........TGATAAGGCCCTAATGACACAA...................................................................   depth=269, length=22
..............................................................GTGTTATTTGGGCTTGTCATT.......   depth=27, length=21
```

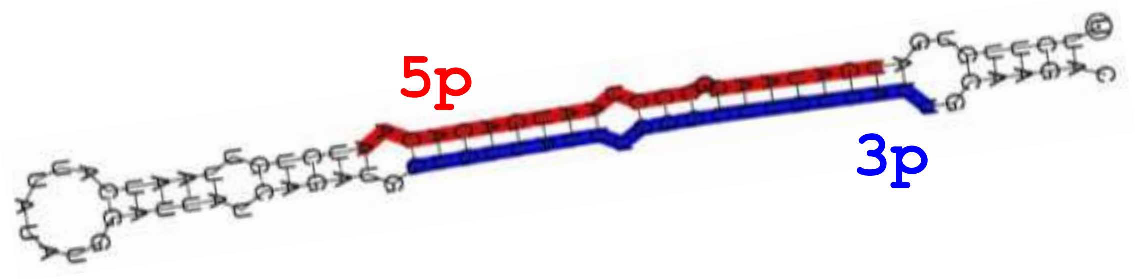

Aang-nmiR050

5'->3'
TTACAAATGATCTTATTGAATACTGGTGAAAGGGGTTTTACACAAGTCCCCATTTACCAGTCCTCAATGAGATCATTTGTAA
.((((((((((((((((((..(((((((((.((((............)))).)))))))))..)))))))))))))))))).
............TTATTGAATACTGGTGAAAGG... depth=12, length=21
............TTATTGAATACTGGTGAAAGGG.. depth=2, length=22
....ATTTACCAGTCCTCAATGAGA.......... depth=15, length=21
....TTACCAGTCCTCAATGAGATC........ depth=279, length=21

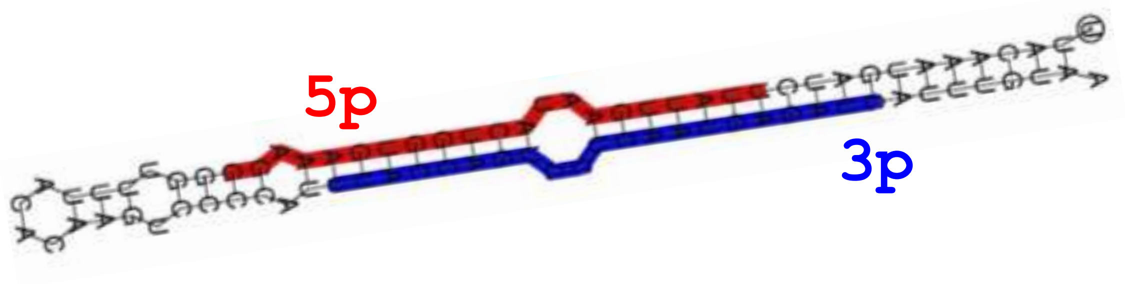

Aang-nmiR051

5'->3'
TGCTGGCAGTGTTCATGTATATGTTGGTCTGCAGAGTGTATGGCCTGCTGGGAATTGTGCCCAGAAATTCCATGTCTATCTCATCAAACAGCCAGGTGCACCCGTACTCCCCCAAC
GAGATAGACATGGAATTTATGGGTACAATTCCCAGCAGGCCATACACCCTGCAGTCCAACATATACGTGAATGCTGCCAGCG
.((((((((((((((((((((((((((.((((((.((((((((((((((((((((((((((((.((((((((((((((((((............(((((....)))))........
)))))))))))))))))).)))))))))))))))))))))))))))).)))))).)))))))))))))))))))))))))).
..........................GTCTGCAGAGTGTATGGCCTG...
.... depth=272, length=21
....
....CCTGCAGTCCAACATATACG............... depth=1, length=20

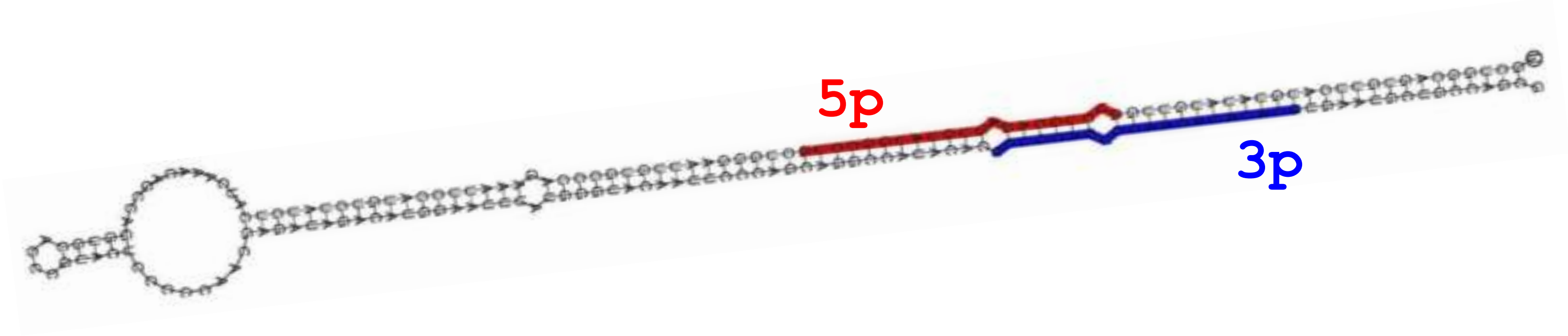

Aang-nmiR052

```
5'->3'
AGGAGGGTAAGTTCATCTATTCCAAAGCAGATAGATTGCCATATATTTTTTTTTGAGGATGTCAACGCTGGCATCAGAAAGACCTTGCAAAATATTTCTTTGGTTTGAGGGTTAGTCTTTTAGCTTTG
GACAACTTATAAATTTTATTTGGCATCTGTCTGCTTCGGAATACGTGAGATCATCCTTTA
.(((((((...(((((.((((((.((((((((((((.((((.(((....(((.((((..((((...(((((....(((..((((((.((((.((......)).))))))))))..))).)))))....
)))).))))).)))...))).))))))))))))))).)))))).)))))...))))))).
...................TTCCAAAGCAGATAGATTG..........................................................................................
............................................................ depth=48, length=19
...................TTCCAAAGCAGATAGATTGC.........................................................................................
............................................................ depth=61, length=20
...................TTCCAAAGCAGATAGATTGCCA.......................................................................................
............................................................ depth=86, length=22
................................................................................................................................
......................GCATCTGTCTGCTTCGGAATA................. depth=41, length=21
```

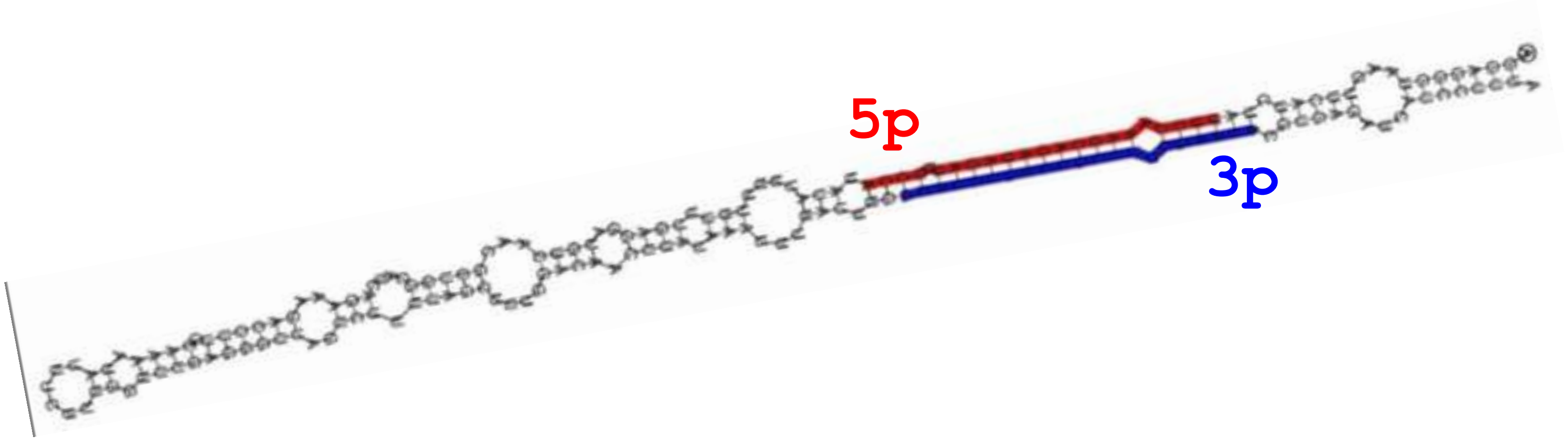

Aang-nmiR053

5'->3'

CGGCAATGAATCGGCTGAATCGCGATTCGATGCCGATTGACTCCTTTCGGCAATGAATCGGCAAAAAATCGCGATTCAGCCGATTCATTGCCGA
(((((((((((((((((((((((((((...((((((((.(.(((....)).).).))))))))...))))))))))))))))))))))))))).
..GCAATGAATCGGCTGAATCGC... depth=141, length=21
....AATGAATCGGCTGAATCGCGA... depth=72, length=21

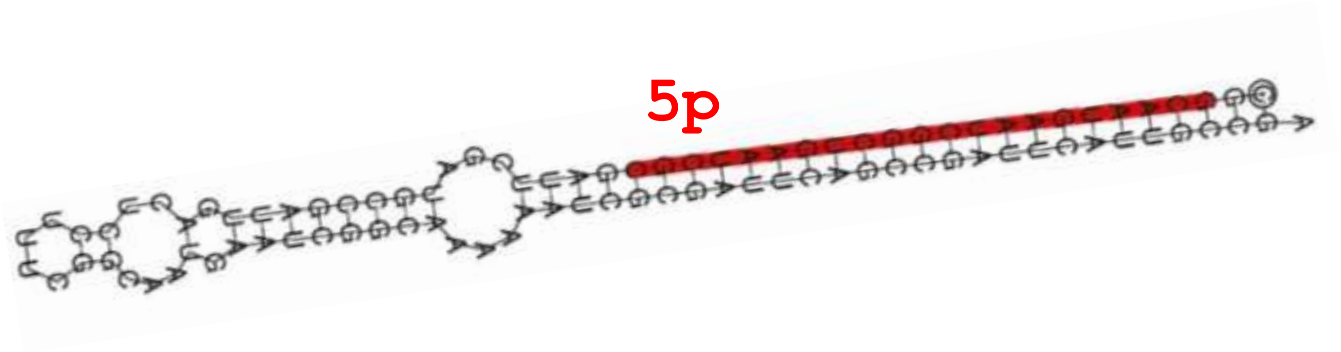

Aang-nmiR054

```
5'->3'
CTAGGGCACTCGTGACCGTCGTGGATGTATATCAGCGAACTCGGCCAATTAAGAGTTCGTCCGGCTCATGTACATGCACGACGGTCACGACTGCCCTAT
.(((((((.((((((((((((((.((((((((.(((((((((..........)))))).....))).)))))))).)))))))))))))).))))))).
............TGACCGTCGTGGATGTATATC..................................................................   depth=175, length=21
..................................................................CATGTACATGCACGACGGTCA............   depth=7, length=21
....................................................................TGTACATGCACGACGGTCACG..........   depth=6, length=21
.........................................................................ATGCACGACGGTCACGACTGC.....   depth=7, length=21
..........................................................................TGCACGACGGTCACGACTGCC....   depth=14, length=21
```

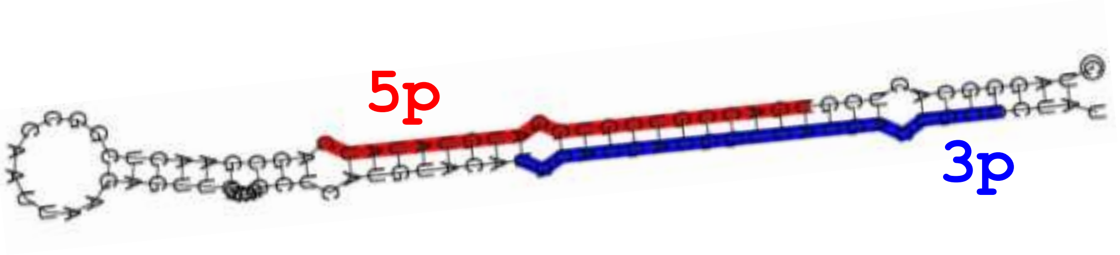

Aang-nmiR055

```
5'->3'
AGTGTGGCCTATCGATCCTTTAGGTTCCTCGAAATTTGAGGCTAGAGGTGTCAGAAAAGTTACCACG
.(((((((...((((((((((((...((((((...)))))))))))))).))).))...)))).))).
...GTGGCCTATCGATCCTTTAG..............................................  depth=33, length=20
........................................GCTAGAGGTGTCAGAAAAGTTAC....  depth=13, length=23
........................................GCTAGAGGTGTCAGAAAAGTTACC...  depth=18, length=24
.........................................CTAGAGGTGTCAGAAAAGT.......  depth=18, length=19
.........................................CTAGAGGTGTCAGAAAAGTT......  depth=17, length=20
.........................................CTAGAGGTGTCAGAAAAGTTAC....  depth=105, length=22
```

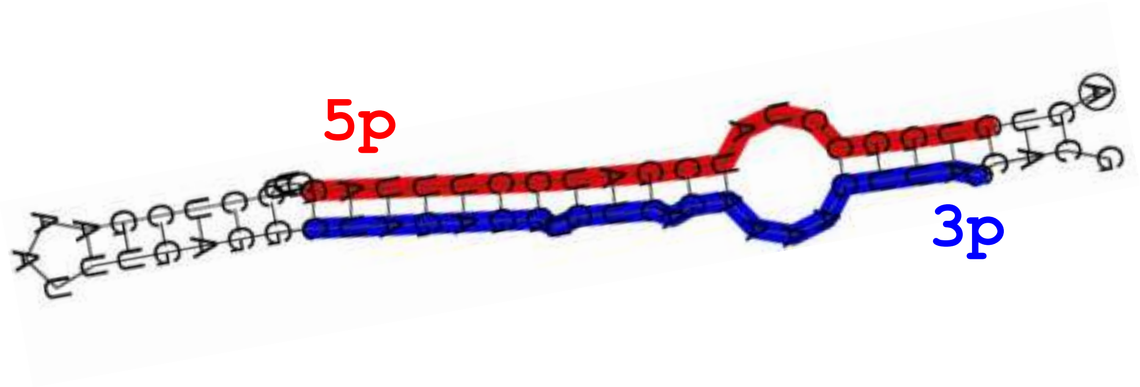

Aang-nmiR056

```
5'->3
GGGTTTCTTGATGATGATAACCGTTGACGCCCAGTCGGAGGGGCTGACTGCTTACGCGGCTCCGCTGATGCGAAATTCACGGTTTGTCTGAAAGATGCTA
.(((.((((...(((((..(((((.(((((...(((((.(((((((.(.......))))))).))))))))....))))))))))))...)))).))).
......CTTGATGATGATAACCGTTGAC........................................................................  depth=4, length=22
......CTTGATGATGATAACCGTTGACG.......................................................................  depth=15, length=23
......CTTGATGATGATAACCGTTGACGCC.....................................................................  depth=8, length=25
..............................................................................ACGGTTTGTCTGAAAGAT....  depth=34, length=18
.............................................................................CACGGTTTGTCTGAAAGAT....  depth=43, length=19
............................................................................TCACGGTTTGTCTGAAAGAT....  depth=22, length=20
...........................................................................TTCACGGTTTGTCTGAAAGAT....  depth=13, length=21
```

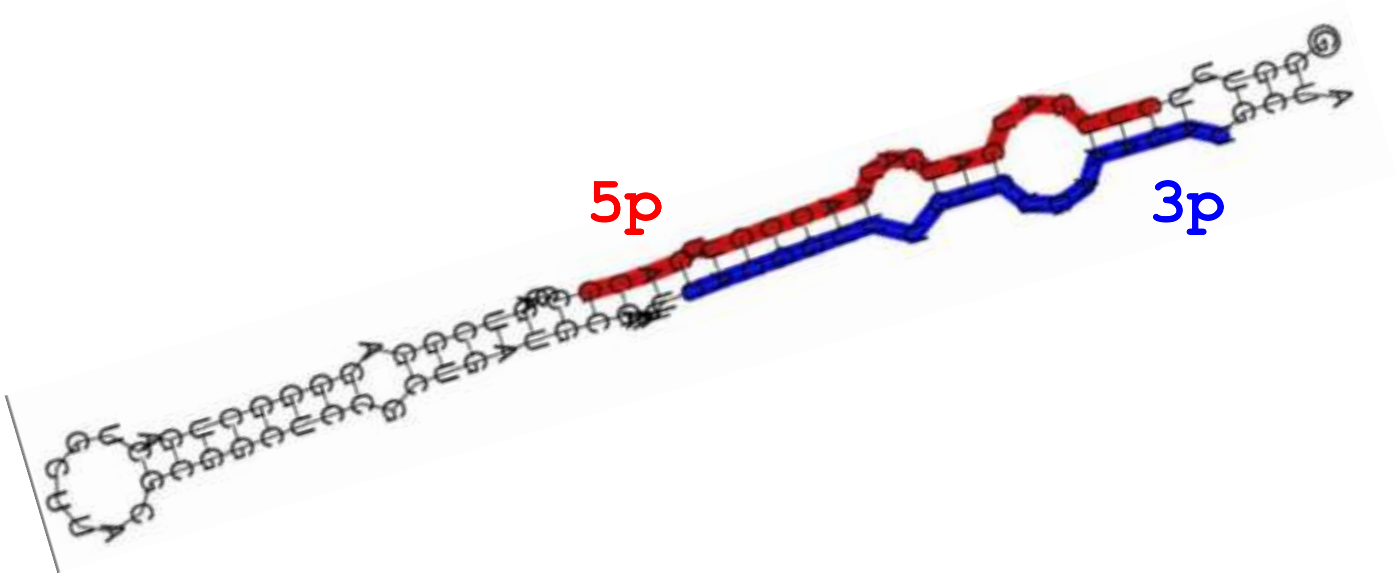

Aang-nmiR057

5'->3'
TCTTTTCCCTAGGAGGTGTCGGCTATGCTGAAATCGGTCGTACTGAAATACACCTCAATGCAAAAGATGAGCCAAAGTAGAGAGGTGTAGTTCGGTACGATCGATTTCGGTATAGTTCGACTCCTAGGGAAAAGT
.((((((((((((...((((((((((((((((((((((((((((((.((((((((..(((.................)))..)))))))).))))))))))))))))))))))).))))))))))))))))))).
.........................TGCTGAAATCGGTCGTACTGA...
depth=78, length=21
....GGTACGATCGATTTCGGTATA.....................
depth=70, length=21

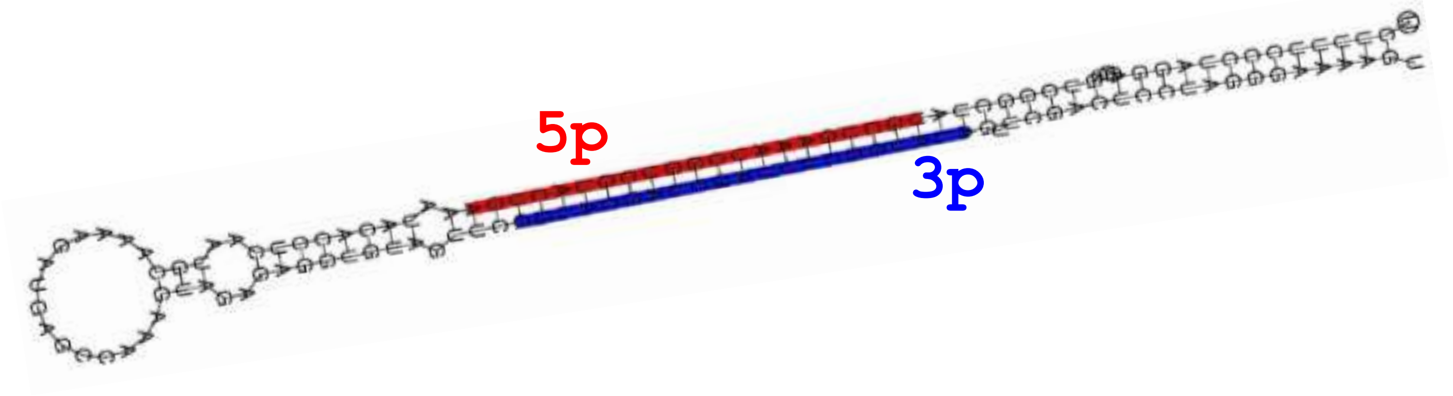

Aang-nmiR058

5'->3'

AAAACGGTCCAGTCCTGAATGGCATGACTTGCAAATTATGTTGGCTTGTTGGGGTGTGTGATATGTAGCTTTTTGTGTGGTGGGCTTACATGCCTTTTGCTGTTGTAGCTAACTCTGTCTCATTCTCATTCCATTTATAGCTACACTATTAGCAGATATG
TAGGCCAGCCACAACCACTCATATCACAGCAATTATTTTTAATATCAGCCATTATTCAAGAGCCTACACAATTTGTAAGGCCATGCTAATCGGGACTTCATCGTT
.((((((..((((((((.(((((((.((((((((((.(((.((((((((((..(((((((((((.((.......((((((.((((((((((...(((((((.((((((((........................))))))))....))))))))))))
))))).)))))))....)))))))))))).)).............))))).........))))).))).)))))))))))..))))))).))))))))..)))))).
...................TGGCATGACTTGCAAATTATG..
.... depth=9, length=21
....
....CAATTTGTAAGGCCATGCTAAT............... depth=145, length=22

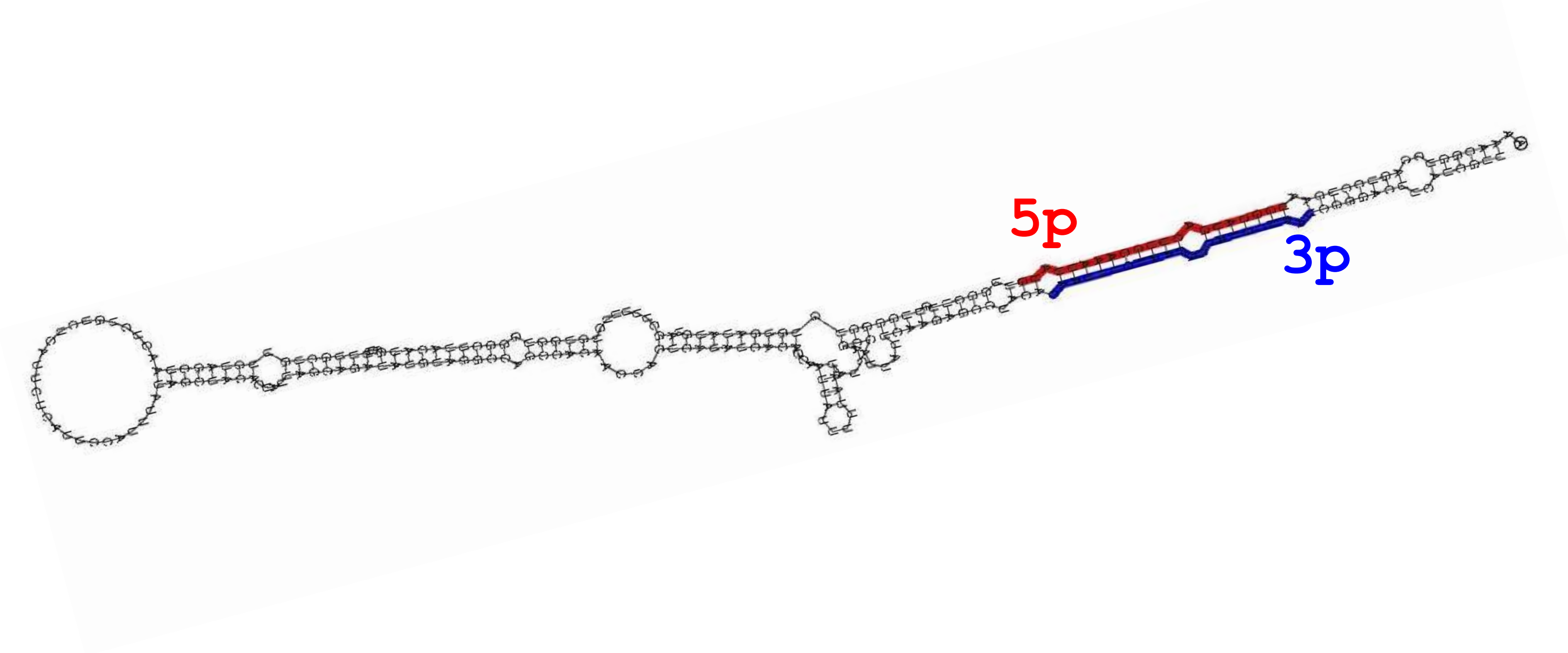

Aang-nmiR059

```
5'->3'
AGATAGCCTGAGTTGTTAGGGTCTGTAAGAAGTGTCCAGCATTTCTCGTCTGAAAAGACCAAAAAACATAAACAAACTTTAGTAGCATACTATATATTTAAGGTCTTAAGCTGTTGAAGTCGATATGGTTTTAAGTGTCTCGTGCTTGTATGCATGATGC
ATAGGAATGTATTGAAACTTCTATGTATATTTATATCTGTTTTGGTGATTAATAAAGTATGTGTACATGATGTAAATATACATAGAAGTTTCAATACATTCCTATGCATCATGCATACAAGCACGAGACACTTAAAACCATATCGACTTCAACAGCTTAA
GACCTTAAATATATAGTATGCTACTAAAGTTTGTTTATGTTTTTTGGTTTTTTCAAATGAGAAATGCTGGACACTTCTTACAGACCCTAACAACTCAGGCTA
.(((((((((((((((((((((((((((((((((((((((((((((((.(((((((((((((((((((((((((((((((((((((((((((((((((((((((((((((((((((((((((((((((((((((((((((((((((((((((((((((((
(((((((((((((((((((((((((((((((((((((((....(((.((....)).)))....))).)))))))))))))))))))))))))))))))))))))))))))))))))))))))))))))))))))))))))))))))))))))))))))))
)))))))))))))))))))))))))))))))))))))))))))).)))))))))))))))))))))))))))))))))))))))))))))))))))
..............................AGTGTCCAGCATTTCTCGTCT.............................................................................................................
................................................................................................................................................................
......................................................................................................   depth=4, length=21
................................................................................................................................................................
................................................................................................................................................................
.........................................................TGAGAAATGCTGGACACTTCT........................   depth=127, length=21
```

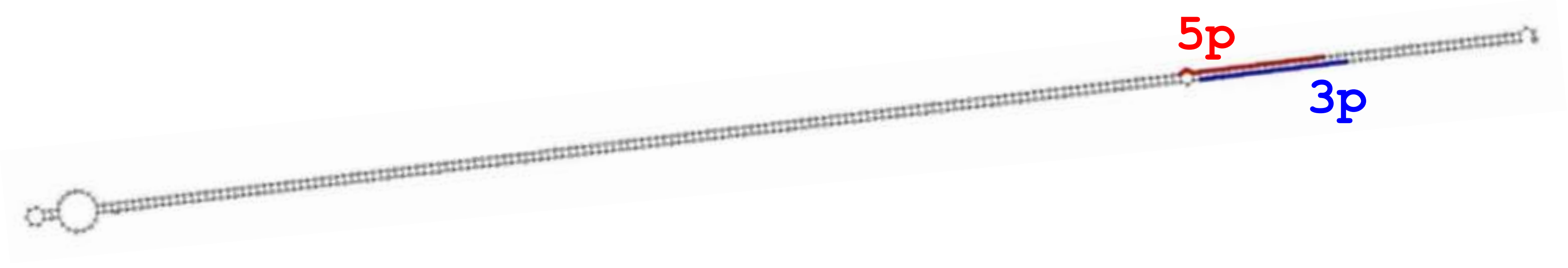

Aang-nmiR060

```
5'->3'
AGAGAGTAGATGGTATGTTTCTACTGAGTTGGTTTCCAAAACTACAGAGCTATGTATATTTTGGAACCTACTTAGTGAAAACATGCCATCTTCTCTTC
.(((((.(((((((((((((.((((((((.((((.((((((.((((......))))..)))))))))).)))))))).))))))))))))).))))).
...............TGTTTCTACTGAGTTGGTTTC..............................................................  depth=30, length=21
...............TGTTTCTACTGAGTTGGTTTCC.............................................................  depth=68, length=22
................................................................AACCTACTTAGTGAAAACATG.............  depth=2, length=21
```

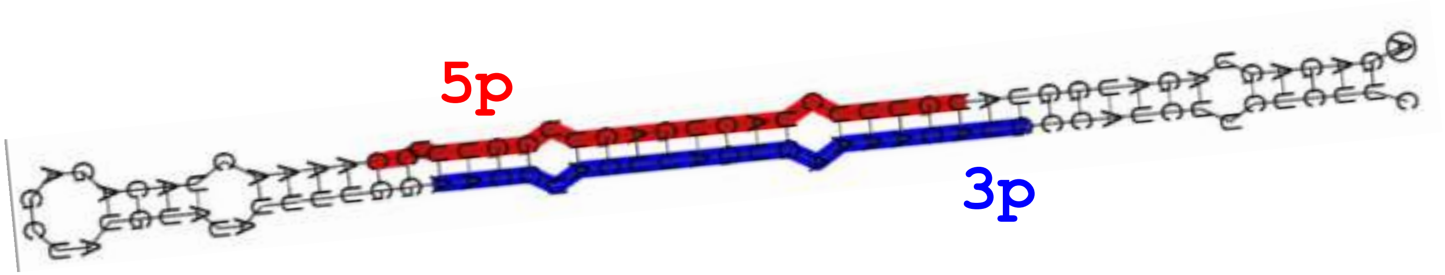

Aang-nmiR061

```
5'->3'
CAGGCGTGGGCGTCTTGGACAAAGCTGGTCCAGGAGTTTAAAACTTTCTCTTATCAAAGTTTTTCCAATGCCGCCCATGCCTT
.(((((((((((.((((((.((((((.....(((((...........))))).....))))))))))).).))))))))))).
....CGTGGGCGTCTTGGACAAAGC.......................................................... depth=21, length=21
...........................................................TTTTTCCAATGCCGCCCATGCC.. depth=91, length=22
```

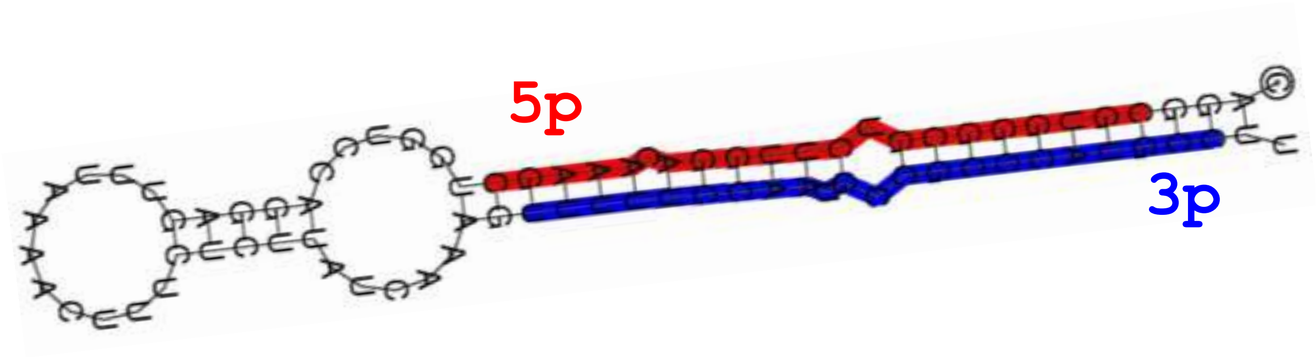

Aang-nmiR062

```
5'->3'
AGAATATCAGTCCCGTATTGAAGATCAACCCATAATACACGAAGTTTCAGTATGGGGTTGATCTTCAATATGGCGCTGATATTCA
.((((((((((.(((((((((((((((((((...((((..((....)).)))).))))))))))))))))))).)))))))))).
...........CCCGTATTGAAGATCAACCCA.....................................................      depth=11, length=21
............CCGTATTGAAGATCAACCCA.....................................................      depth=4, length=20
.......................................................GGTTGATCTTCAATATGGCGC.........      depth=59, length=21
.........................................................TTGATCTTCAATATGGCGCTG.......      depth=8, length=21
```

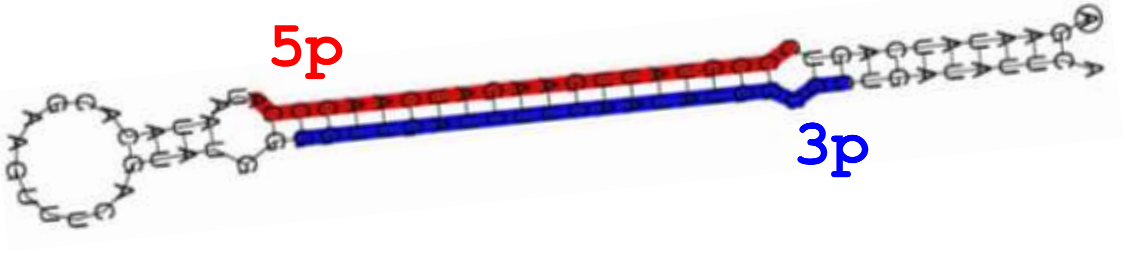

Aang-nmiR063

```
5'->3'
CAAAAGCTCCTTTGATTTTGATTTTCAGTACGAATACAAACCGTGAAAGGATTTGTATTCGTACTGAAAATCAAAATCAAATGAGCTTTT
.((((((((.((((((((((((((((((((((((((((((((......)).)))))))))))))))))))))))))))))).))))))))
.............GATTTTGATTTTCAGTACGAATA......................................................  depth=5, length=23
..............ATTTTGATTTTCAGTACGAATA......................................................  depth=5, length=22
...............TTTTGATTTTCAGTACGAATA......................................................  depth=13, length=21
................TTTGATTTTCAGTACGAATA......................................................  depth=6, length=20
.........................................................TTCGTACTGAAAATCAAAATC............  depth=8, length=21
```

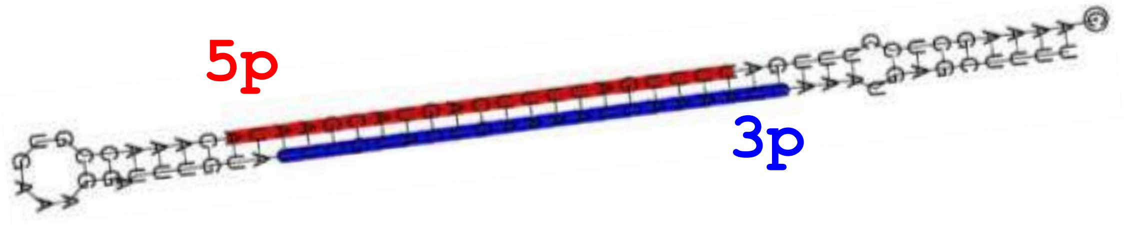

Aang-nmiR064

```
5'->3'
GAGTAAGGGTTTTAACTCATGGATATGCATAACCAGCATCTTAAACAAGGCGAAGCCGAAGCTTGCTACCCCCAATCTAATTTGATGAATATTTAGATTGGCGGTAGCTTCGGCGTCTCCATGTTTCA
TGCATATCCATGAGTTAAAACCCTTACTCA
((((((((((((((((((((((((((((((............(((((.((.((.(((((((((....(((.(((((((((............))))))))).)))))))))))).)).)).))))).)
))))))))))))))))))))))))))))).
.........TTTTAACTCATGGATATGCA...................................................................................................
.............................. depth=7, length=20
........GTTTTAACTCATGGATATGCA...................................................................................................
.............................. depth=42, length=21
................................................................................................................................
..CATATCCATGAGTTAAAACCC....... depth=18, length=21
```

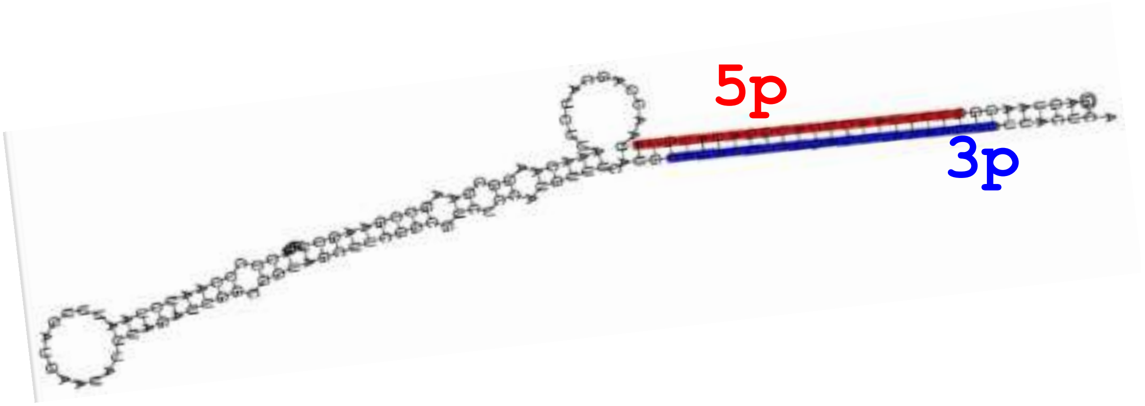

Aang-nmiR065

```
5'->3'
CATTTCTATTGTTTATTGATTTGATGCTAATGAATGAAATGAGGGTTTTTGGTGGTTTTTCATCGTTACCCTCATTTTATTTCTTAACACCAGACTAATGAACAACAGAAGTG
.((((((.((((((((((.((((.((.(((.(((((((((((((((...((((((....))))))..))))))))))))))).))).)).)))).)))))))))).)))))).
...........TTTATTGATTTGATGCTAATG.................................................................................   depth=3, length=21
...........TTTATTGATTTGATGCTAATGA................................................................................   depth=3, length=22
..................................................................................CTTAACACCAGACTAATGAAC..........   depth=7, length=21
..................................................................................CTTAACACCAGACTAATGAACA.........   depth=12, length=22
...................................................................................TTAACACCAGACTAATGAACA.........   depth=12, length=21
```

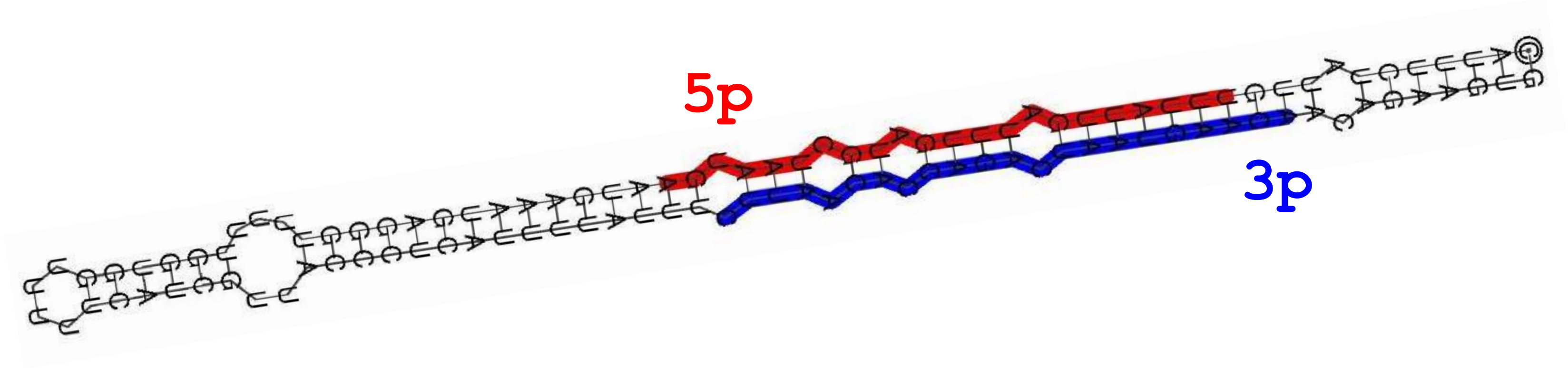

Supplement: Supplementary file 2 [file Data_Sheet_2.PDF]
