# Supplementary material for: Novel and Conserved miRNAs Among Brazilian Pine and Other Gymnosperms
Source: Front Genet. 2019 Mar 22;10:222. doi: 10.3389/fgene.2019.00222 (PMC6448024; doi:10.3389/fgene.2019.00222)

**Data S3. *A. angustifolia* conserved pre-miRNAs identified in *A. cunninghamii*.** Conserved pre-miRNAs from *A. angustifolia* were Blasted against *A. cunninghamii* unigenes. BLAST-search statistics, as well as sequence alig[illegible]

| *Araucaria angustifolia* | *Araucaria cunninghamii* |
|---|---|
| **Ang-miR167c** | **Acun-miR167** |

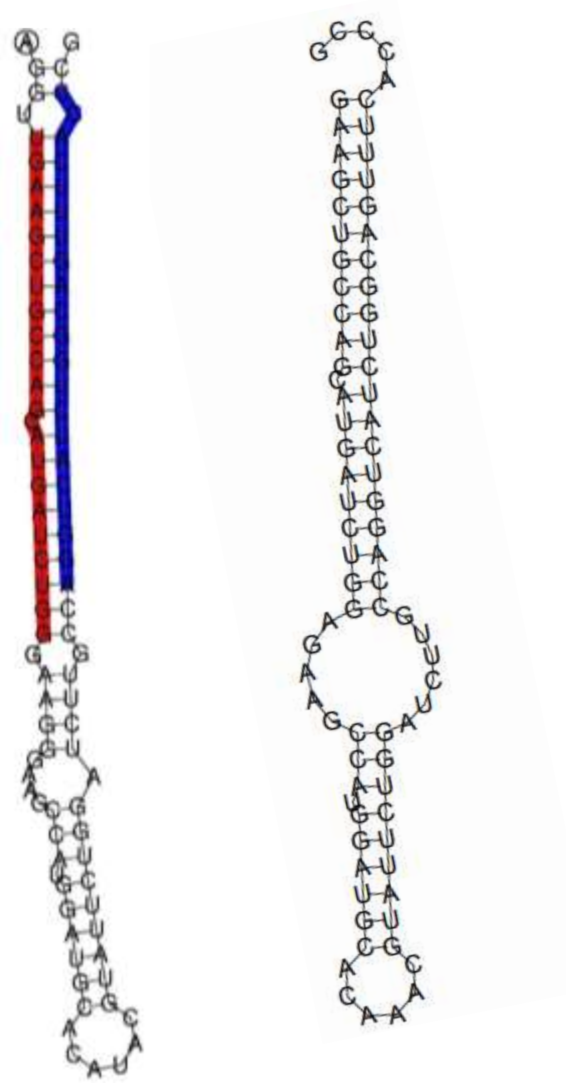

```
Query= Aang-miR167c
         (91 letters)



Sequences producing significant alignments:
Acu-207112

>Acu-207112
Length = 94

 Score =  121 bits (61), Expect = 1e-27
 Identities = 80/86 (93%), Gaps = 4/86 (4%)
 Strand = Plus / Plus


Query: 6   gaagctgccagcatgatctgggaagggaagccatggatgcacatacgtattctggatctt 65
           |||||||||||||||||||||     |||||||||||||||| ||||||||||||||||
Sbjct: 1   gaagctgccagcatgatctgga----gaagccatggatgcacaaacgtattctggatctt 56


Query: 66 gccaggtcatctggcagtttcacccg 91
           ||||||||||||||||||||||||||
Sbjct: 57 gccaggtcatctggcagtttcacccg 82
```

| *Araucaria angustifolia* | *Araucaria cunninghamii* |
| --- | --- |
| **Ang-miR1314a** | **Acun-miR1314a** |

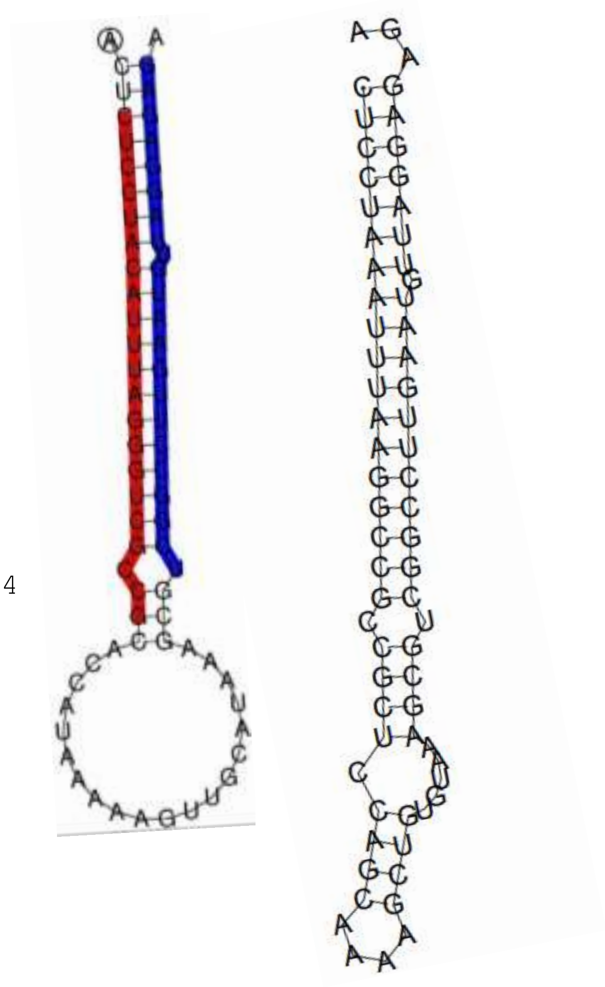

**Query= Aang-miR1314a**
(71 letters)

Sequences producing significant alignments:
Acu-157334

>Acu-157334
Length = 361

Score = 63.9 bits (32), Expect = 2e-10
Identities = 59/68 (86%)
Strand = Plus / Plus

```
Query: 4   ctcctacatttagggtcgccgcaccataaaaagttgcataaagcgtcggccttgaatgtt 63
           |||||| ||||| || |||||| |||  ||||| ||  ||||||||||||||||||||||
Sbjct: 55  ctcctaaatttaaggccgccgctccagcaaaagctgtgtaaagcgtcggccttgaatgtt 114


Query: 64  aggagaga 71
           ||||||||
Sbjct: 115 aggagaga 122
```

**Query= Aang-miR1314b**
(107 letters)

Sequences producing significant alignments:
Acu-157334

>Acu-157334
Length = 361

```
 Score =  111 bits (56), Expect = 2e-24
 Identities = 92/104 (88%)
 Strand = Plus / Plus


Query: 1   tggtaagcaatgaaggaaactctcctacatttagggtcgccgcaccataaaaagttgcat 60
           ||||| |||| ||||||||| |||||| ||||| || |||||| |||  ||||| ||  |
Sbjct: 34  tggtaggcaacgaaggaaaccctcctaaatttaaggccgccgctccagcaaaagctgtgt 93


Query: 61  aaagcgtcggccttgaatgttaggagagagtctttctttgtttg 104
           ||||||||||||||||||||||||||||||||||||||||||||
Sbjct: 94  aaagcgtcggccttgaatgttaggagagagtctttctttgtttg 137
```

| *Araucaria angustifolia* | *Araucaria cunninghamii* |
| --- | --- |
| **Ang-miR1314b** | **Acun-miR1314b** |

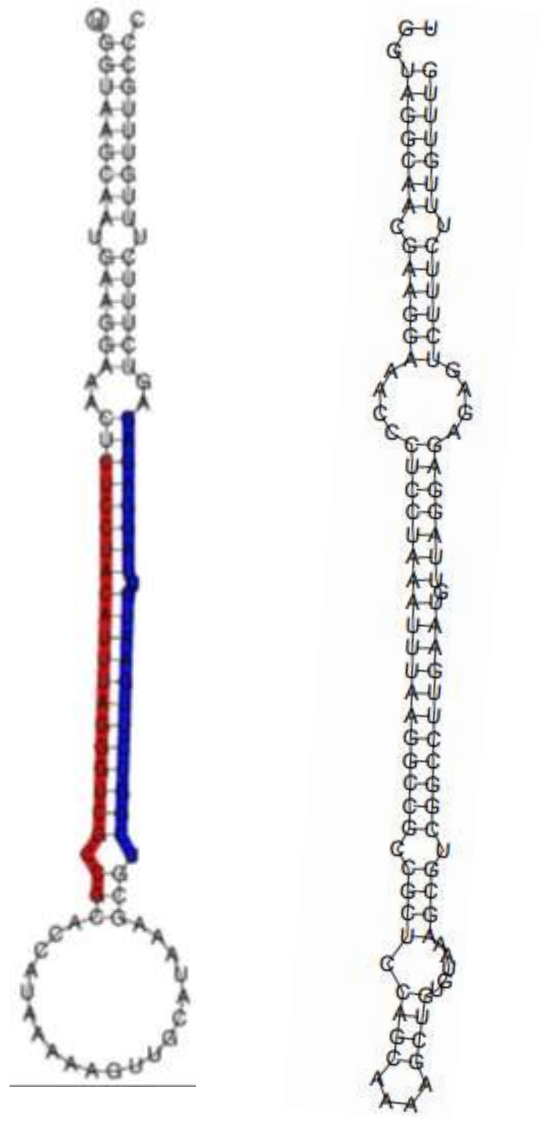

**Query= Aang-miR1314c**
(135 letters)

Sequences producing significant alignments:
Acu-157334

>Acu-157334
Length = 361

Score = 202 bits (102), Expect = 7e-52
Identities = 123/130 (94%)
Strand = Plus / Plus

```
Query: 1   tggcatagatggcctggtaggcaacgaacgaaacccttctaaatttaaggtcgccgctcc 60
           ||||| |||||||||||||||||||||| |||||||| |||||||||||| |||||||||
Sbjct: 20  tggcacagatggcctggtaggcaacgaaggaaaccctcctaaatttaaggccgccgctcc 79

Query: 61  atcaaaagttgtgtaaagcgtcggccttgaatgttaggagagagtctttctttgtttgcc 120
           | |||||| ||||||||||||||||||||||||||||||||||||||||||||||||| |
Sbjct: 80  agcaaaagctgtgtaaagcgtcggccttgaatgttaggagagagtctttctttgtttgtc 139

Query: 121 catgccatct 130
           ||||||||||
Sbjct: 140 catgccatct 149
```

| *Araucaria angustifolia* | *Araucaria cunninghamii* |
|---|---|
| **Ang-miR1314c** | **Acun-miR1314c** |

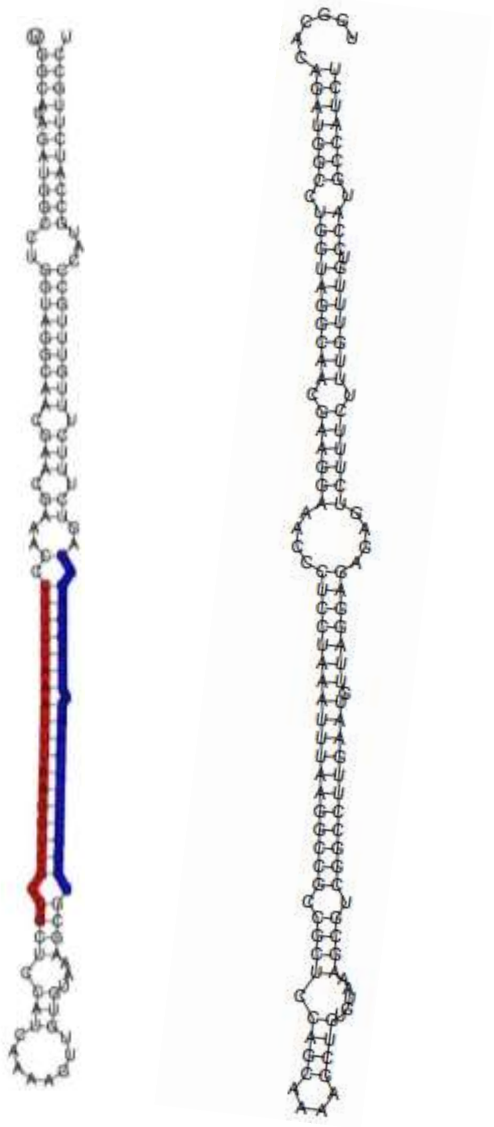

**Query= Aang-miR1314d**
(124 letters)

Sequences producing significant alignments:
Acu-157334

>Acu-157334
Length = 361

```
 Score =  204 bits (103), Expect = 2e-52
 Identities = 118/123 (95%)
 Strand = Plus / Plus


Query: 1   agatggcctggtaggcaacgaacgaaacccttctaaatttaaggtcgccgctccatcaaa 60
           |||||||||||||||||||||| |||||||| |||||||||||| |||||||||| ||||
Sbjct: 26  agatggcctggtaggcaacgaaggaaaccctcctaaatttaaggccgccgctccagcaaa 85


Query: 61  agttgtgtaaagcgtcggccttgaatgttaggagagagtctttctttgtttgtccatgcc 120
           || |||||||||||||||||||||||||||||||||||||||||||||||||||||||||
Sbjct: 86  agctgtgtaaagcgtcggccttgaatgttaggagagagtctttctttgtttgtccatgcc 145


Query: 121 atc 123
           |||
Sbjct: 146 atc 148
```

| *Araucaria angustifolia* | *Araucaria cunninghamii* |
|---|---|
| **Ang-miR1314d** | **Acun-miR1314d** |

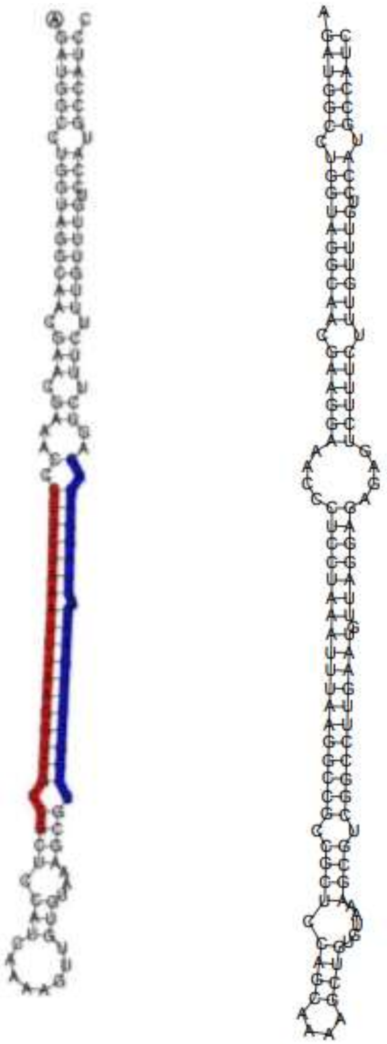

Supplement: Supplementary file 3 [file Data_Sheet_3.PDF]
