# Supplementary material for: Novel and Conserved miRNAs Among Brazilian Pine and Other Gymnosperms
Source: Front Genet. 2019 Mar 22;10:222. doi: 10.3389/fgene.2019.00222 (PMC6448024; doi:10.3389/fgene.2019.00222)

**Data S4. *A. angustifolia* novel pre-miRNAs identified in *A. cunninghamii*.** Novel pre-miRNAs from *A. angustifolia* were Blasted against *A. cunninghamii* unigenes. BLAST-search statistics, as well as sequence alignment and hairpin structures were shown.

| *Araucaria angustifolia* | *Araucaria cunninghamii* |
|---|---|
| **Ang-nmiR003** | **Acun-nmiR003** |

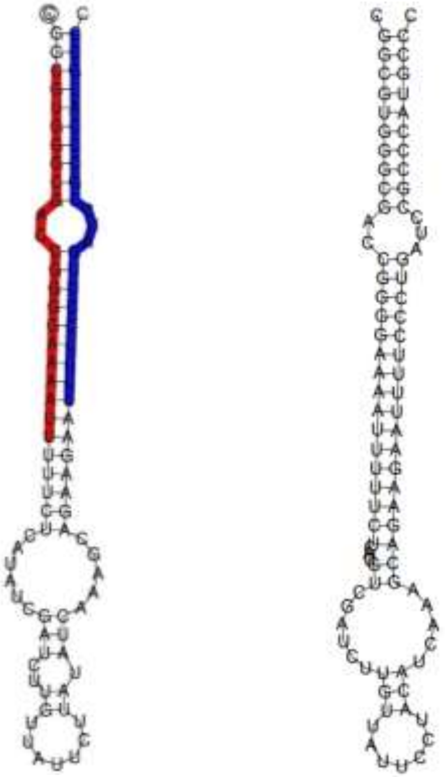

```
Query= Aang-nmiR003
        (90 letters)


                                                                    Score    E
Sequences producing significant alignments:                         (bits)  Value

Acu-405642                                                            155   1e-37

>Acu-405642
Length = 383

Score =  155 bits (78), Expect = 1e-37
Identities = 87/90 (96%)
Strand = Plus / Plus


Query: 1   cggcgtgggcgaccggggaaaatttttctcatatcgatcttgttattcttatatcaaagc 60
           |||||||||||||||||||||||||||||||| ||||||||||||||| || ||||||||
Sbjct: 239 cggcgtgggcgaccggggaaaatttttctcatgtcgatcttgttattcctacatcaaagc 298


Query: 61  agaagaattttccctgatccgcccatgccc 90
           ||||||||||||||||||||||||||||||
Sbjct: 299 agaagaattttccctgatccgcccatgccc 328
```

```
Query= Aang-nmiR005
        (85 letters)

                                                                     Score    E
Sequences producing significant alignments:                          (bits)  Value

Acu-56083                                                              121    1e-27

>Acu-56083
Length = 424

Score =  121 bits (61), Expect = 1e-27
Identities = 80/85 (94%), Gaps = 1/85 (1%)
Strand = Plus / Minus


Query: 1   gtactcttgacagcccgaaatcagcgagtttgctatgcgaagacaatggagtatcaaact 60
           ||||||||||||||||||||||||||||||||||||  |||||||||||   |||| ||||
Sbjct: 349 gtactcttgacagcccgaaatcagcgagtttgctatttgaagacaatggcctatc-aact 291


Query: 61  ggctgattcggactatcaagagtga 85
           |||||||||||||||||||||||||
Sbjct: 290 ggctgattcggactatcaagagtga 266
```

| *Araucaria angustifolia* | *Araucaria cunninghamii* |
| --- | --- |
| **Ang-nmiR005** | **Acun-nmiR005** |

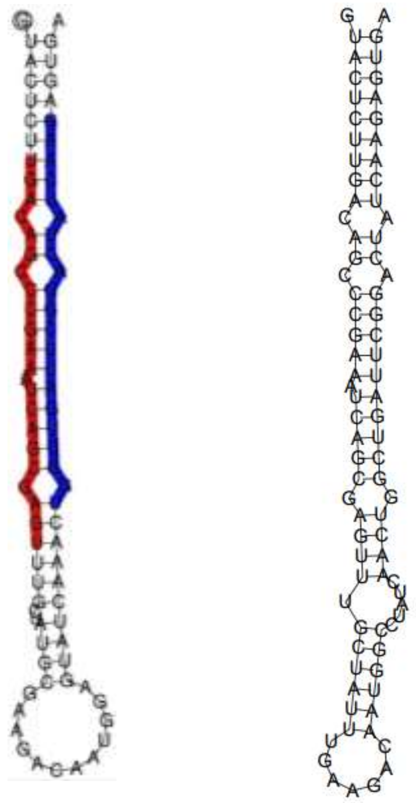

| *Araucaria angustifolia* | *Araucaria cunninghamii* |
| --- | --- |
| **Ang-nmiR015** | **Acun-nmiR015** |

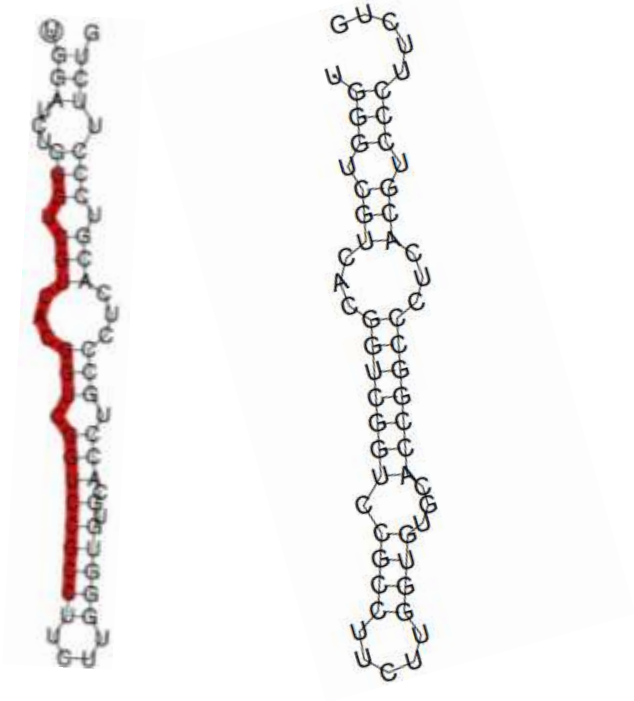

**Query= Aang-nmiR015**
(64 letters)

```
                                                                  Score    E
Sequences producing significant alignments:                       (bits) Value

Acu-88071                                                           92   8e-19


>Acu-88071
Length = 481

Score = 91.7 bits (46), Expect = 8e-19
Identities = 56/58 (96%), Gaps = 1/58 (1%)
Strand = Plus / Plus


Query: 7   tgggtcgtcacggtcggtccgccttcttgggtgtgcacctgccctcacgtcccttctg 64
           |||||||||||||||||||||||||||| |||||||||| ||||||||||||||||||
Sbjct: 344 tgggtcgtcacggtcggtccgccttctt-ggtgtgcaccggccctcacgtcccttctg 400
```

| *Araucaria angustifolia* | *Araucaria cunninghamii* |
|---|---|
| **Ang-nmiR021** | **Acun-nmiR021** |

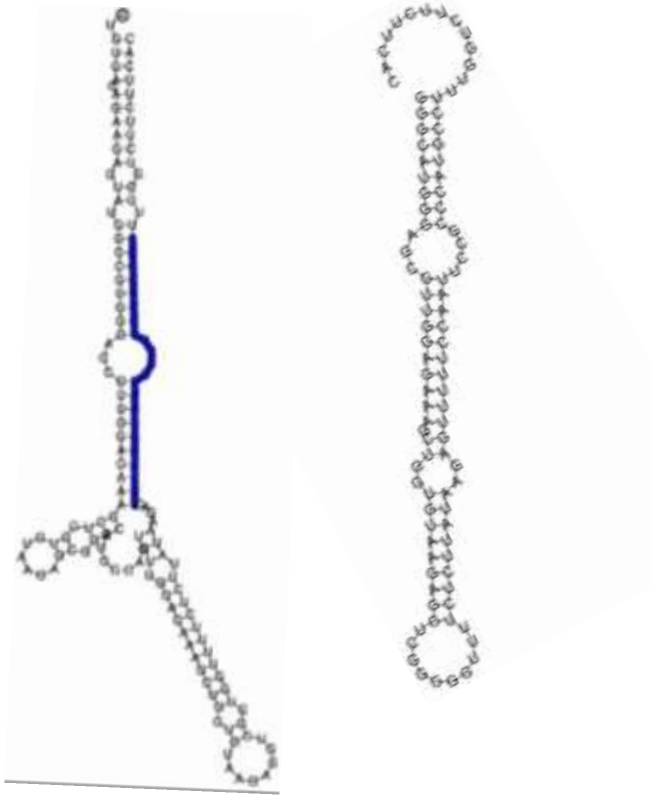

```
Query= Aang-nmiR021
        (149 letters)

                                                                     Score    E
Sequences producing significant alignments:                          (bits) Value

Acu-440915                                                             174   2e-43


>Acu-440915
Length = 238

Score =  174 bits (88), Expect = 2e-43
Identities = 94/96 (97%)
Strand = Plus / Minus


Query: 54  gggcatgggagtgttggagaaagctggtgtaagaggtcggtggttttctcttataagagt 113
           ||||||||||| |||||||||||||||||||||||||||| |||||||||||||||||||
Sbjct: 180 gggcatgggagcgttggagaaagctggtgtaagaggtcgggggttttctcttataagagt 121


Query: 114 ttttccaattccgcccatgcctttggtcttcttcac 149
           ||||||||||||||||||||||||||||||||||||
Sbjct: 120 ttttccaattccgcccatgcctttggtcttcttcac 85
```

| *Araucaria angustifolia* | *Araucaria cunninghamii* |
|---|---|

**Ang-nmiR033** **Acun-nmiR033**

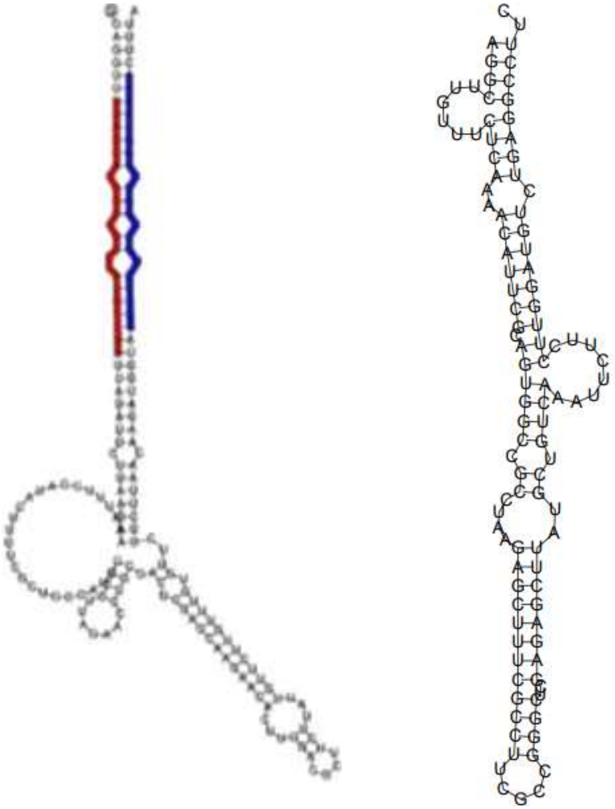

```
Query= Aang-miR033
        (109 letters)

                                                                   Score    E
Sequences producing significant alignments:                         (bits) Value

Acu-426749                                                           176   3e-44


>Acu-426749
Length = 180

Score =  176 bits (89), Expect = 3e-44
Identities = 101/105 (96%)
Strand = Plus / Plus


Query: 1   aggcttgtttctcataacattcgcagtggccgcctaagagctttcgcctttgccgggctc 60
           |||||||||||||| |||||||||||||||||||||||||||||||||| |||||||||
Sbjct: 17  aggcttgtttctcaaaacattcgcagtggccgcctaagagctttcgccttcgccgggctc 76


Query: 61  gagagcttatgctgttcaattcttccttggatgtctgaggccttc 105
           |||||||||||||||  ||||||||||||||||||||||||||||
Sbjct: 77  gagagcttatgctgtcaaattcttccttggatgtctgaggccttc 121
```

```
Query= Aang-nmiR036
        (112 letters)
                                                                 Score    E
Sequences producing significant alignments:                      (bits) Value

Acu-28639                                                          82   1e-15
Acu-23755                                                          82   1e-15

>Acu-28639
Length = 362

Score = 81.8 bits (41), Expect = 1e-15
Identities = 62/69 (89%)
Strand = Plus / Minus


Query: 44  tgaagggagtaagatttctcggaacggattttcaataaataggaacacaggttttatcac 103
            ||||| |||| || ||||| ||||||| |||||||||| || ||||||||||||||||||
Sbjct: 362 tgaagcgagtgaggtttctgggaacgggttttcaataattatgaacacaggttttatcac 303


Query: 104 agacgaata 112
            |||||||||
Sbjct: 302 agacgaata 294


>Acu-23755
Length = 86

Score = 81.8 bits (41), Expect = 1e-15
Identities = 44/45 (97%)
Strand = Plus / Plus


Query: 4  tcgttttgatggagccttgttcctatttactggcaaccctgaag 48
           |||||||||| ||||||||||||||||||||||||||||||||||
Sbjct: 40 tcgttttgatagagccttgttcctatttactggcaaccctgaag 84
```

| *Araucaria angustifolia* | *Araucaria cunninghamii* |
|---|---|
| **Ang-nmiR036** | **Acun-nmiR036** |

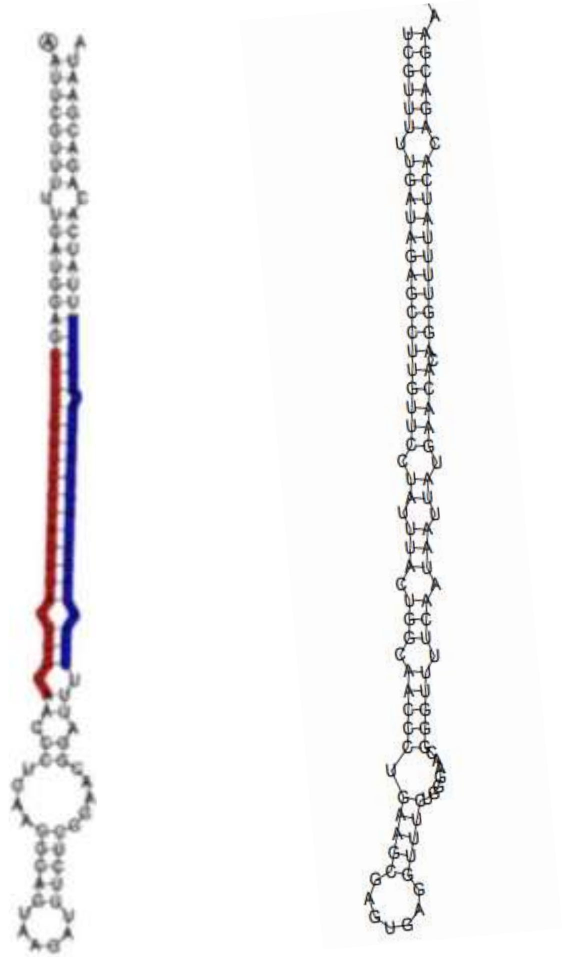

Supplement: Supplementary file 4 [file Data_Sheet_4.PDF]
