# Supplementary material for: Novel and Conserved miRNAs Among Brazilian Pine and Other Gymnosperms
Source: Front Genet. 2019 Mar 22;10:222. doi: 10.3389/fgene.2019.00222 (PMC6448024; doi:10.3389/fgene.2019.00222)
Supplement: Supplementary file 5 [file Data_Sheet_5.PDF]

**Data S5. Expression patterns of conserved mature miRNAs of *A. angustifolia* in different tissues.** The RT-qPCR validation of miRNAs were done in five different tissues (X-axis) : YL (Young Leaf), OL (Old Leaf), St (stem), MR (Main Root), SR (Secondary root). The relative expression values (Y-axis) presented here were the means of four biological replicates ± SD. Letters *a*, *b*, *c or d i*ndicate statistical differences: $P < 0.05$ (one-way ANOVA followed by Duncan's test).

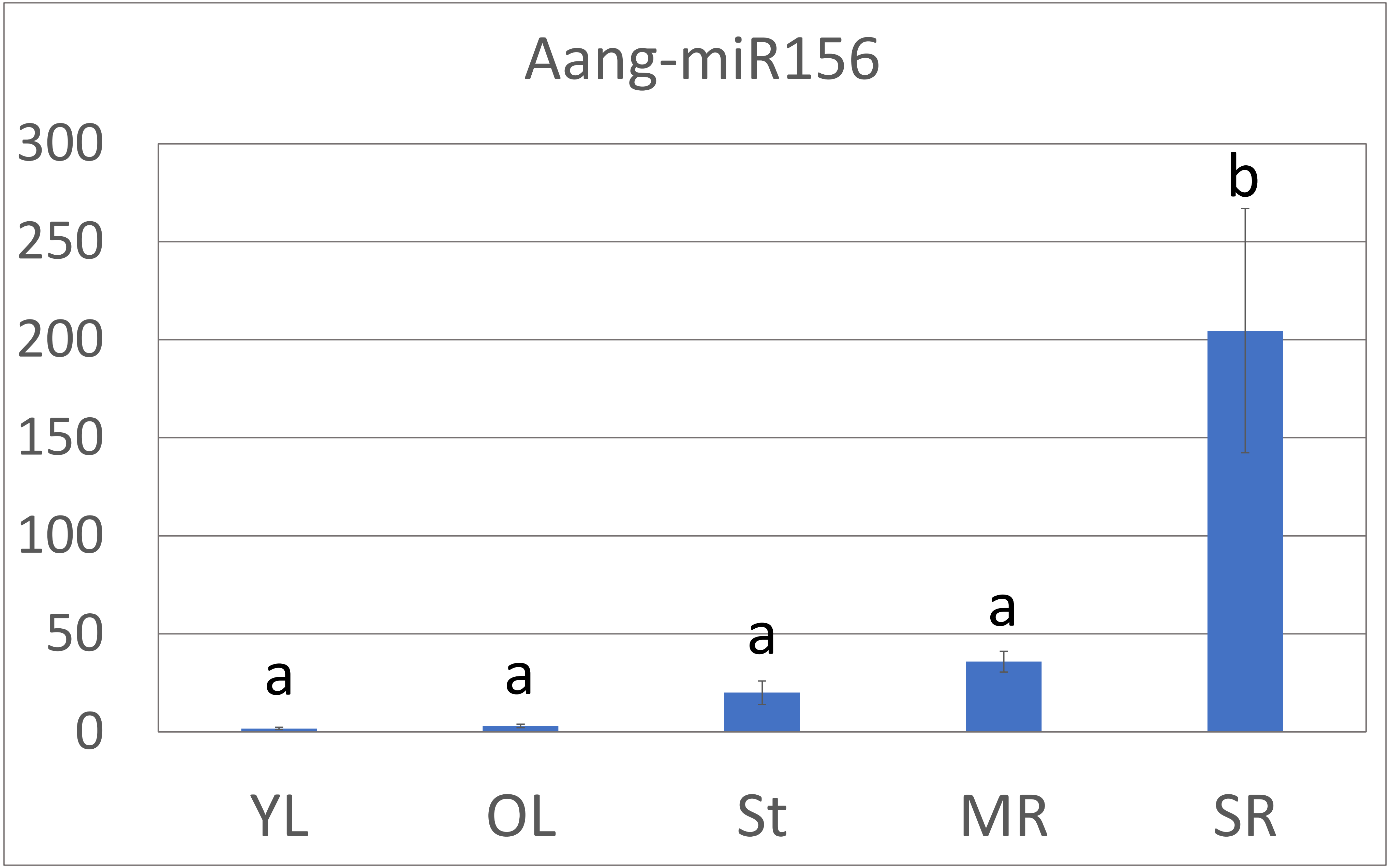
Aang-miR156
300
250
200
150
100
50
0
a
a
a
a
b
YL
OL
St
MR
SR

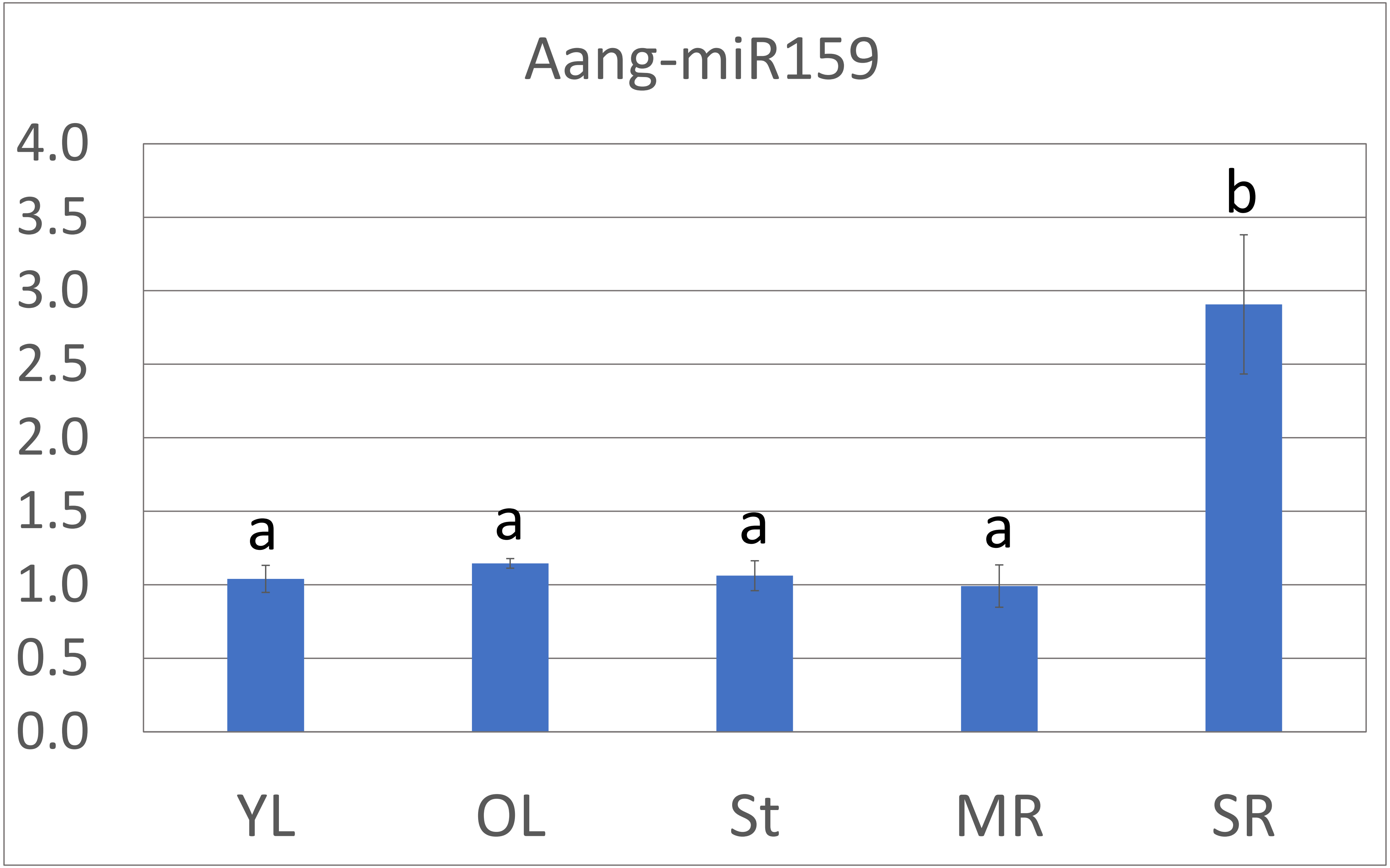
Aang-miR159
4.0
3.5
3.0
2.5
2.0
1.5
1.0
0.5
0.0
a
a
a
a
b
YL
OL
St
MR
SR

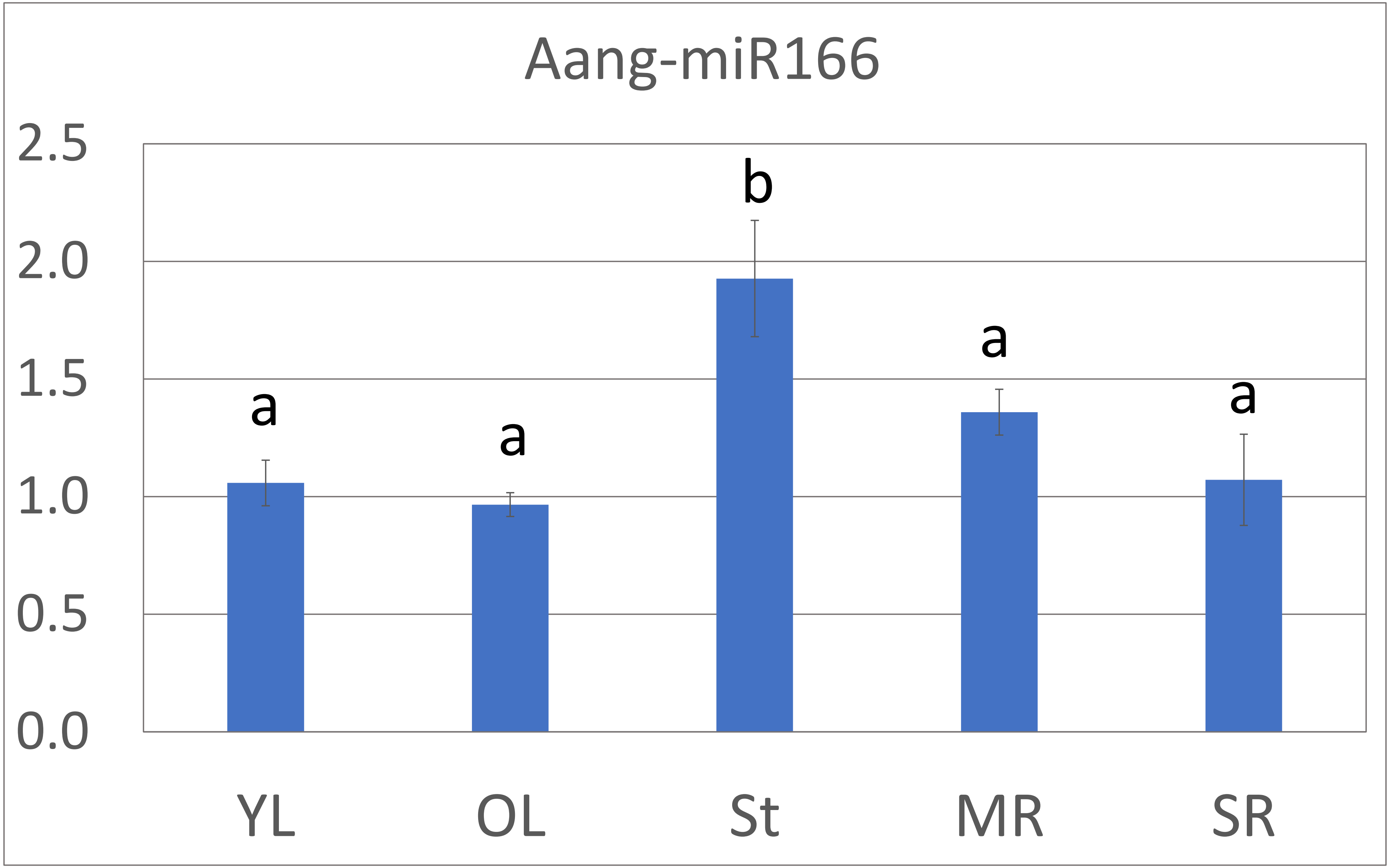
Aang-miR166
2.5
2.0
1.5
1.0
0.5
0.0
a
a
b
a
a
YL
OL
St
MR
SR

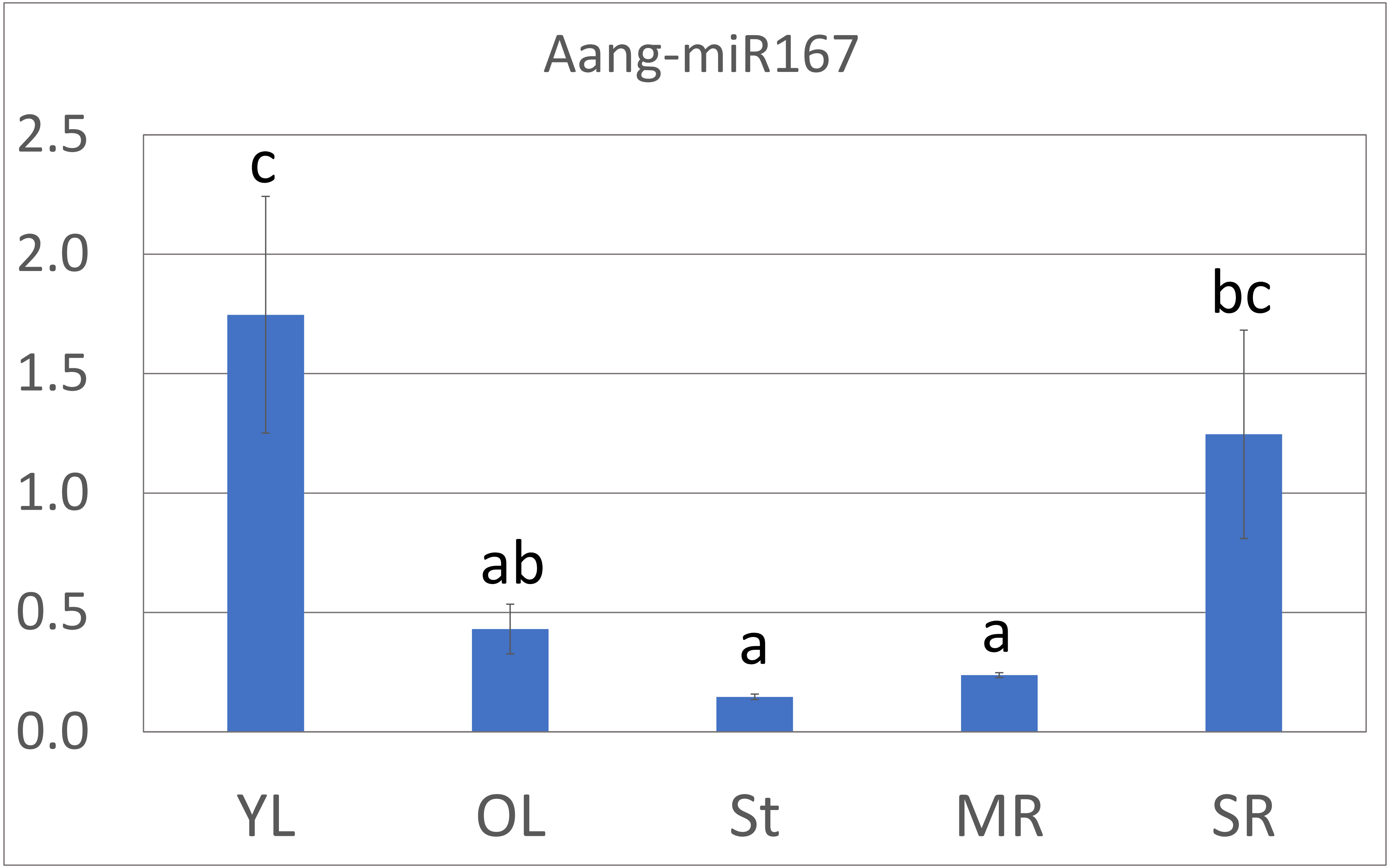
Aang-miR167
2.5
2.0
1.5
1.0
0.5
0.0
c
ab
a
a
bc
YL
OL
St
MR
SR

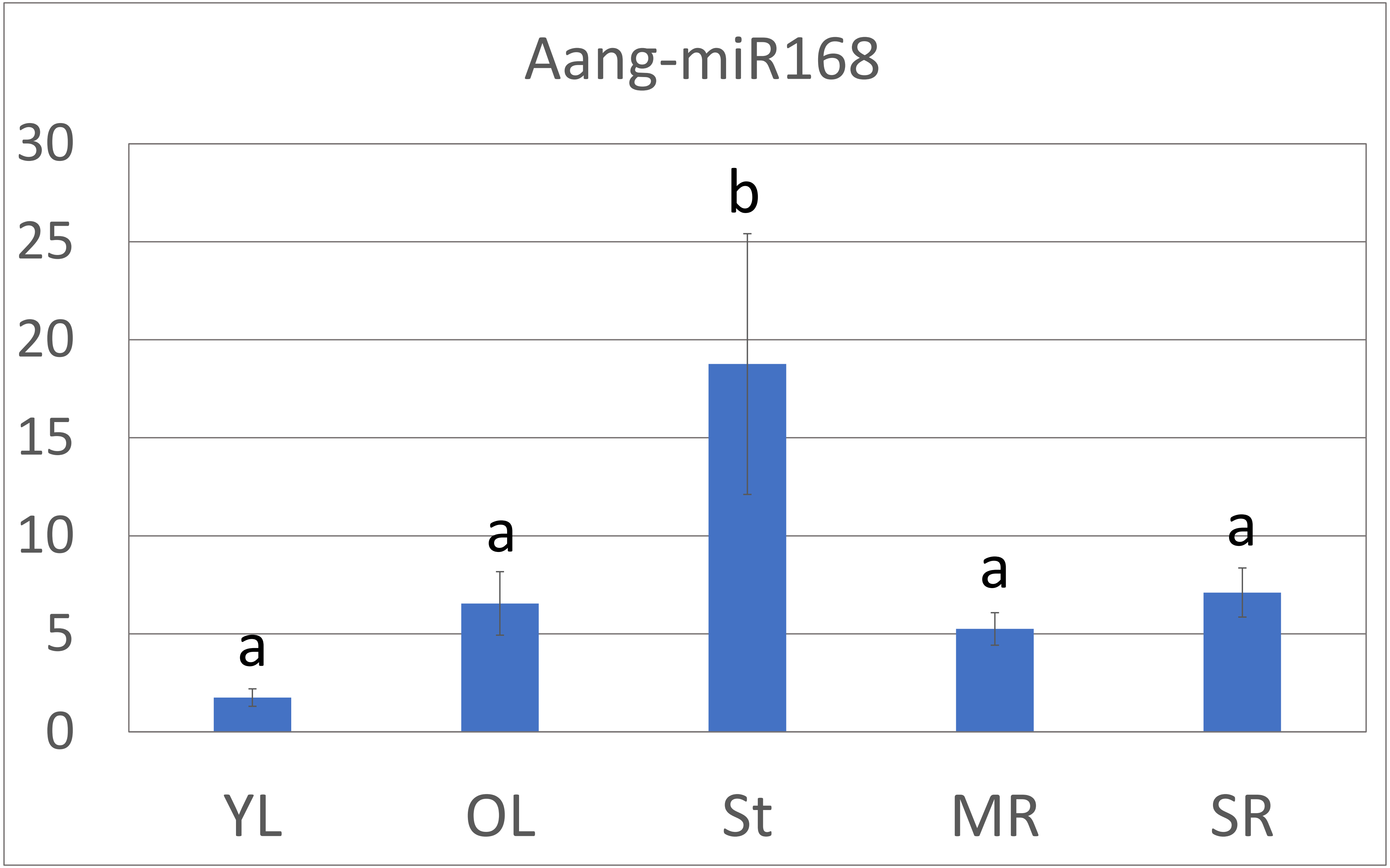
Aang-miR168
30
25
20
15
10
5
0
b
a
a
a
a
YL
OL
St
MR
SR

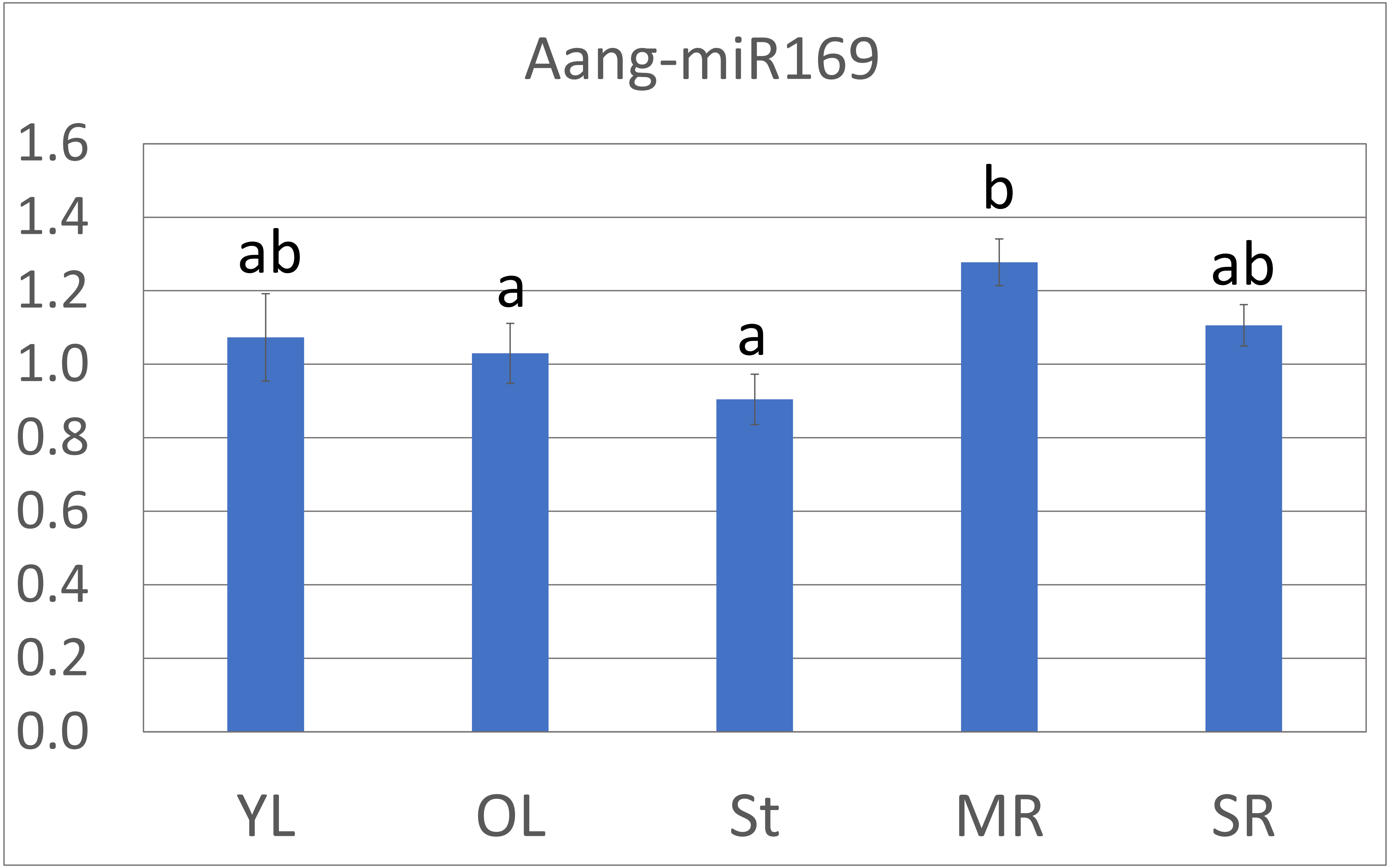
Aang-miR169
1.6
1.4
1.2
1.0
0.8
0.6
0.4
0.2
0.0
ab
a
a
b
ab
YL
OL
St
MR
SR

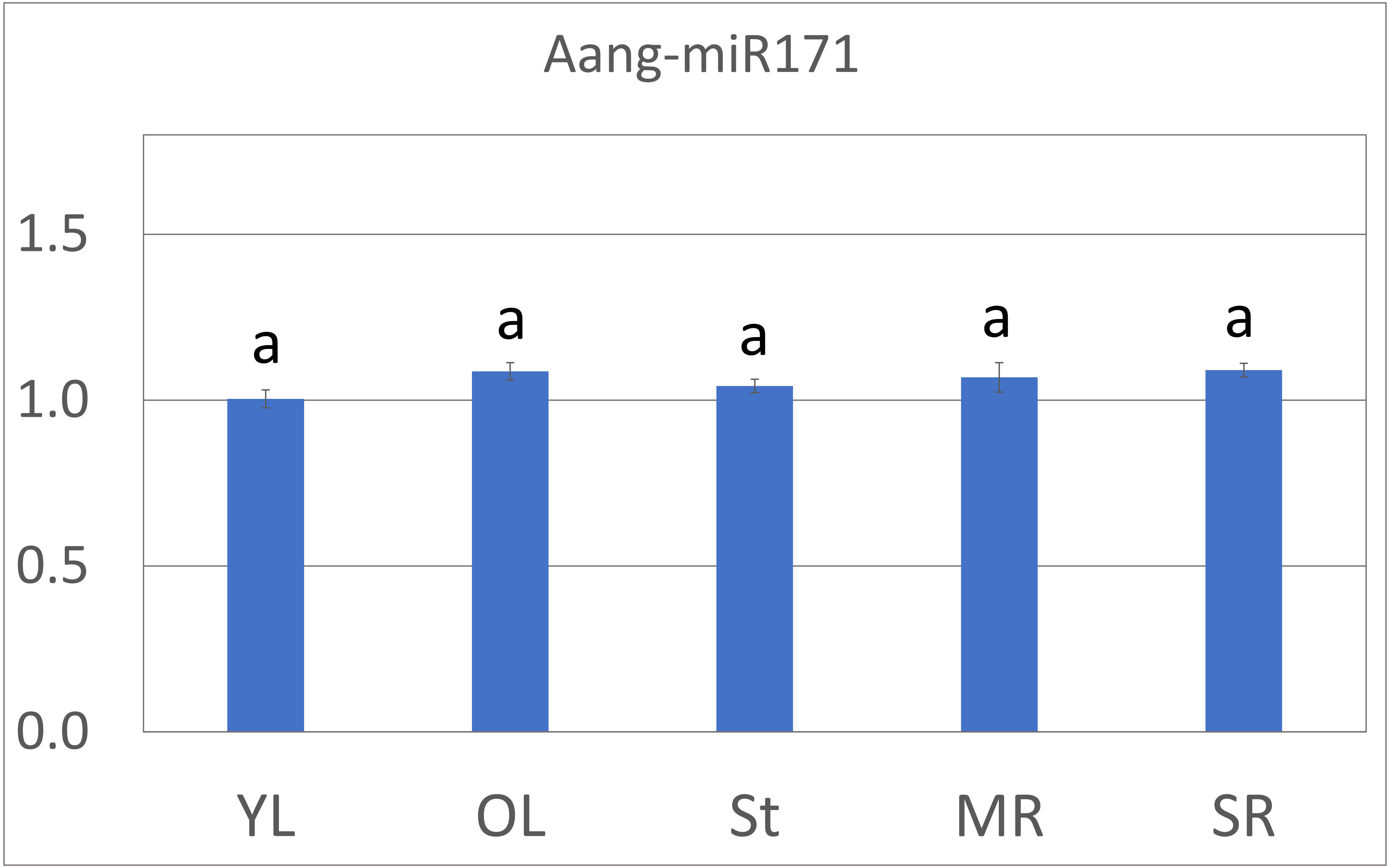
Aang-miR171
1.5
1.0
0.5
0.0
a
a
a
a
a
YL
OL
St
MR
SR

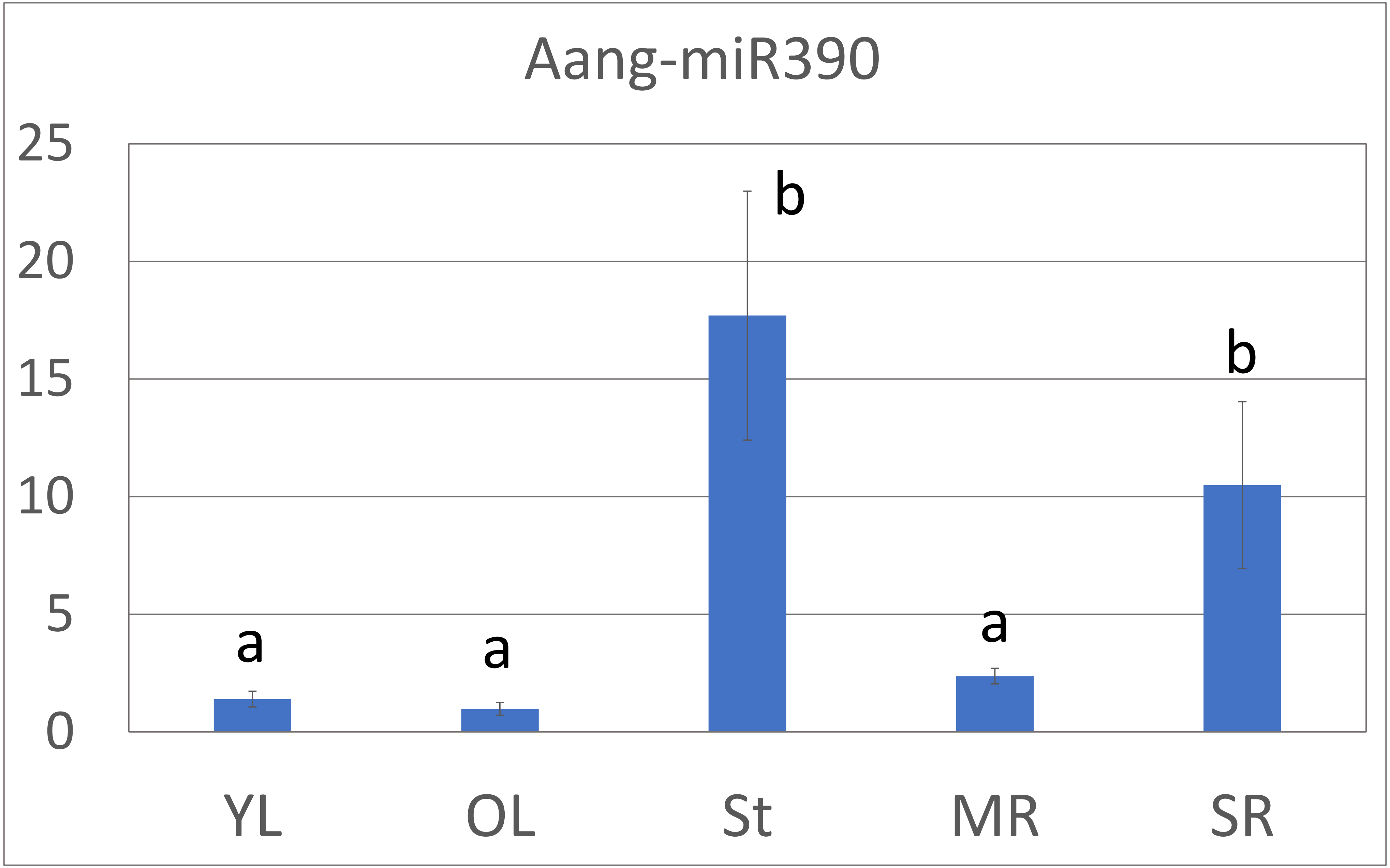
Aang-miR390
25
20
15
10
5
0
a
a
b
a
b
YL
OL
St
MR
SR

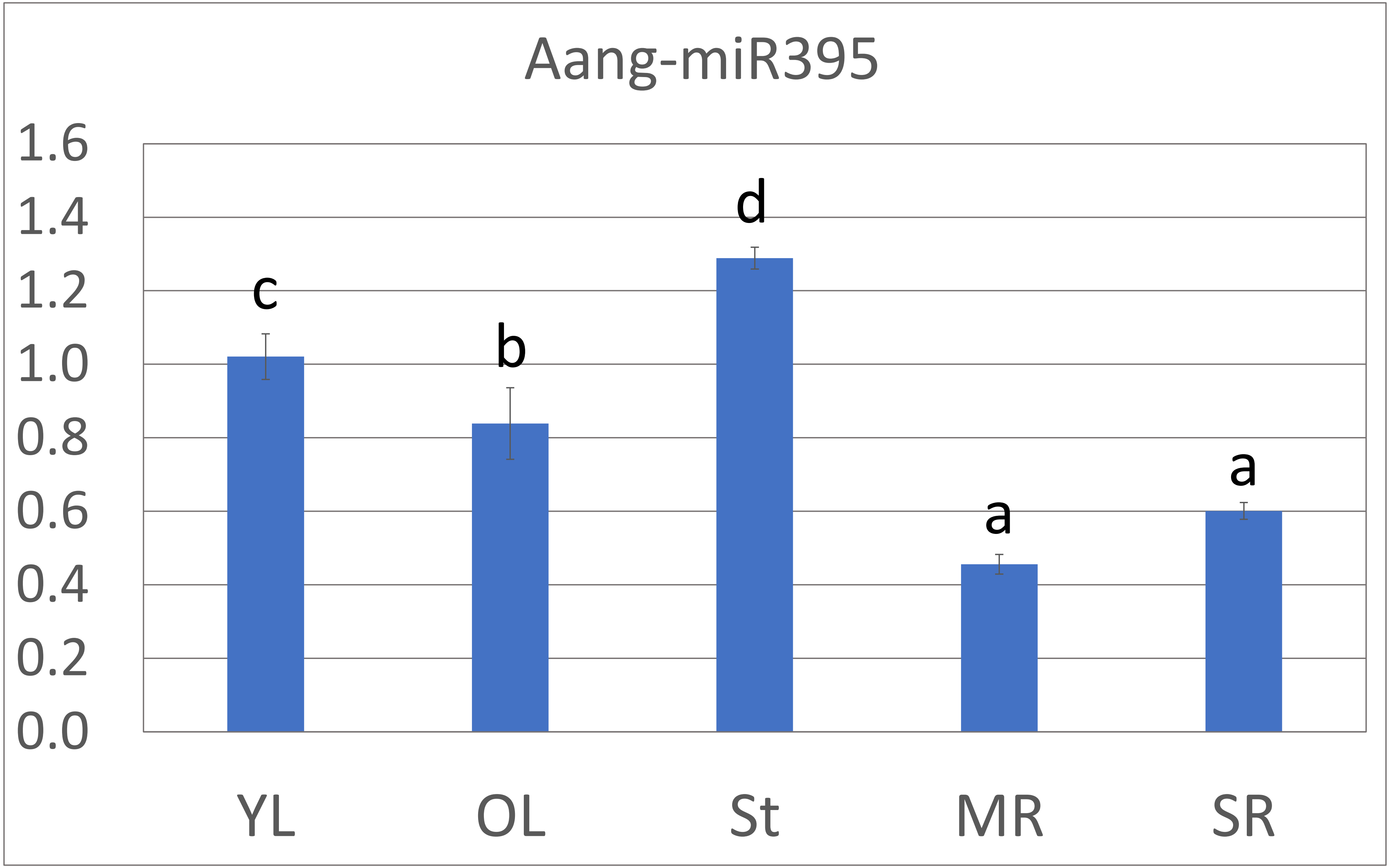
Aang-miR395
1.6
1.4
1.2
1.0
0.8
0.6
0.4
0.2
0.0
c
b
d
a
a
YL
OL
St
MR
SR

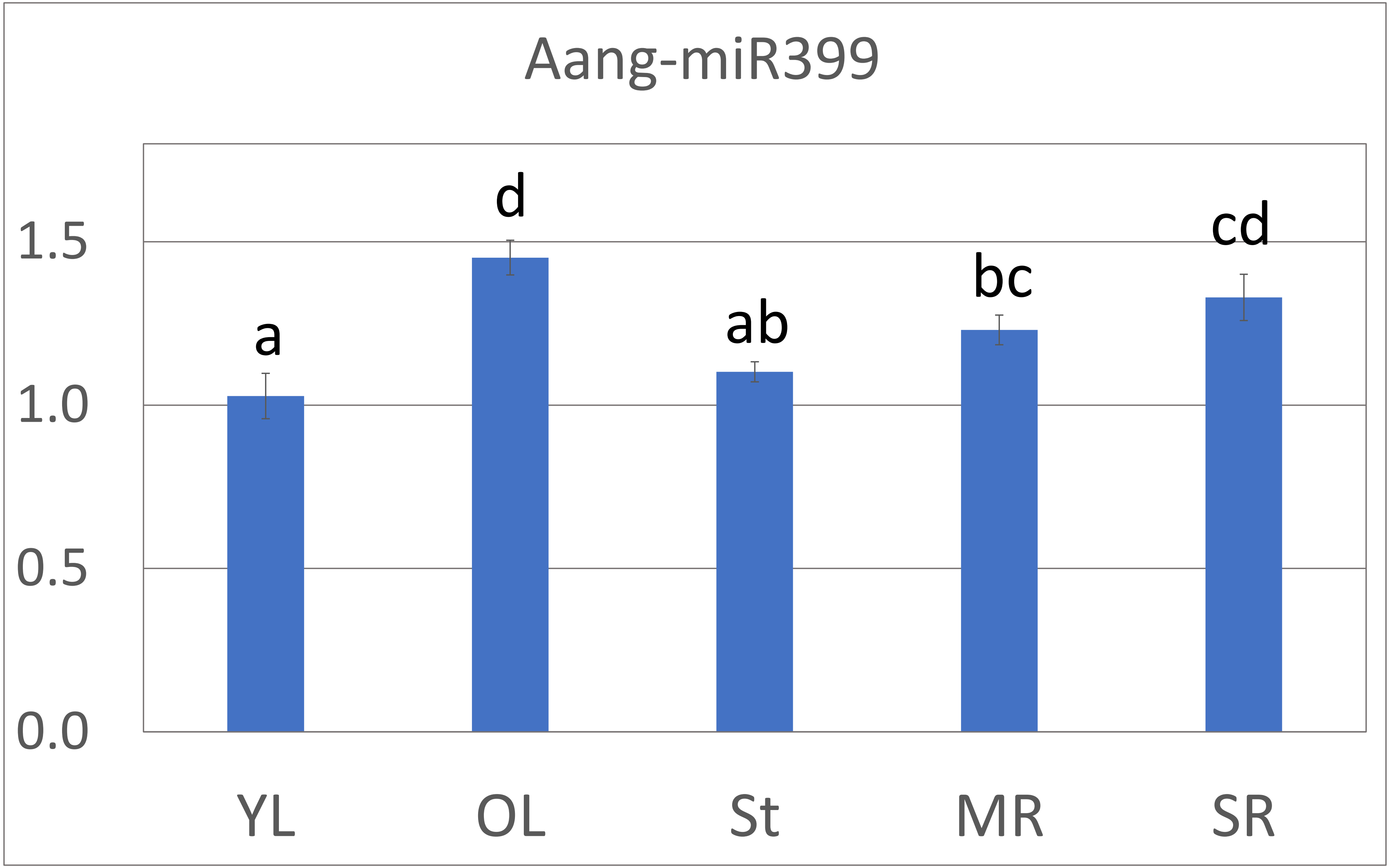
Aang-miR399
1.5
1.0
0.5
0.0
a
d
ab
bc
cd
YL
OL
St
MR
SR

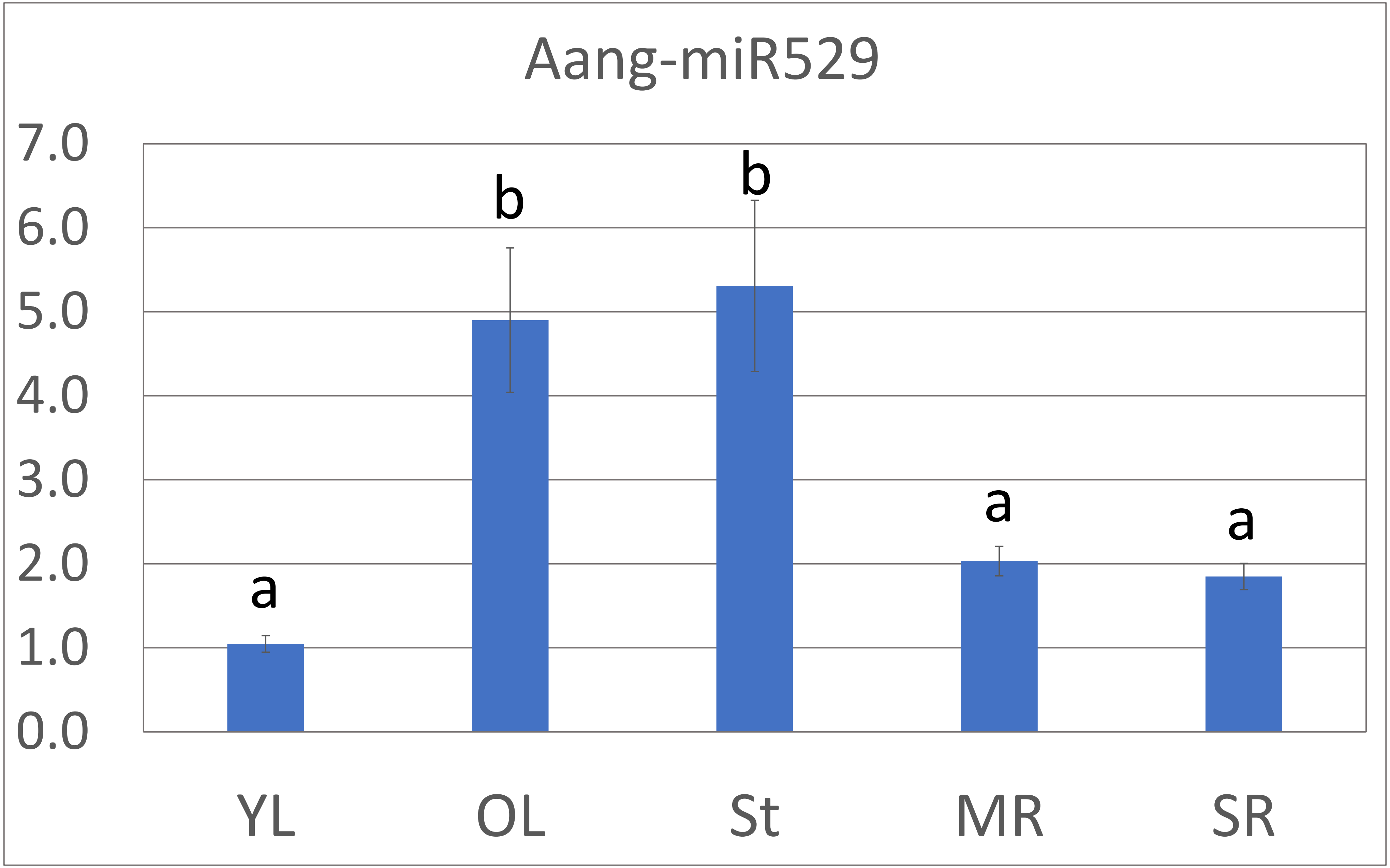
Aang-miR529
7.0
6.0
5.0
4.0
3.0
2.0
1.0
0.0
a
b
b
a
a
YL
OL
St
MR
SR

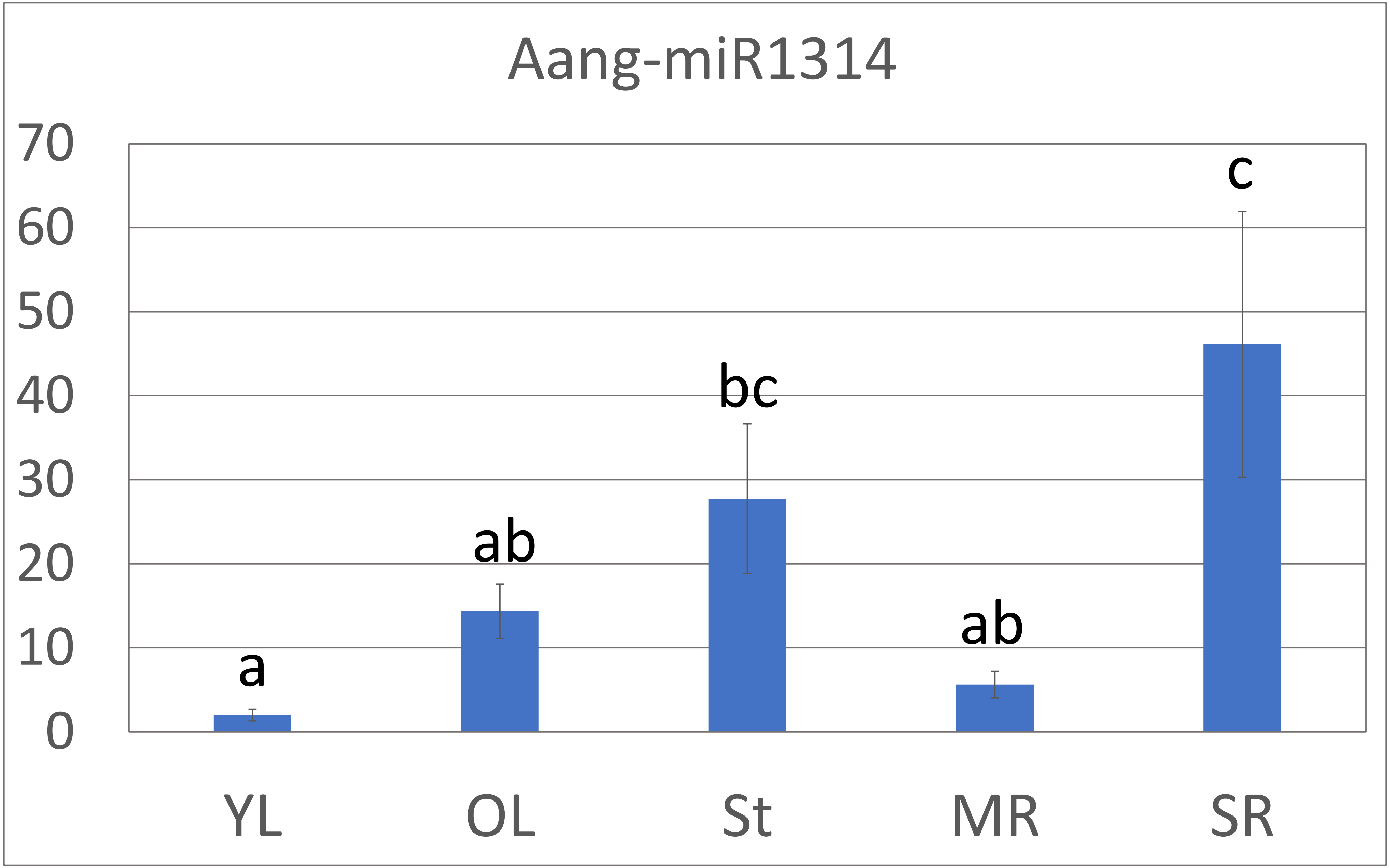

Aang-miR1314
70
60
50
40
30
20
10
0
a
ab
bc
ab
c
YL
OL
St
MR
SR
