# Supplementary material for: Novel and Conserved miRNAs Among Brazilian Pine and Other Gymnosperms
Source: Front Genet. 2019 Mar 22;10:222. doi: 10.3389/fgene.2019.00222 (PMC6448024; doi:10.3389/fgene.2019.00222)
Supplement: Supplementary file 6 [file Data_Sheet_6.PDF]

Data S6. Expression patterns of novel mature miRNAs of *A. angustifolia* in different tissues. The RT-qPCR validation of miRNAs were done in five different tissues (X-axis) : YL (Young Leaf), OL (Old Leaf), St (stem), MR (Main Root), SR (Secondary root). The relative expression values (Y-axis) presented here were the means of four biological replicates ± SD. Letters *a*, *b*, *c or d* indicate statistical differences: $P < 0.05$ (one-way ANOVA followed by Duncan's test).

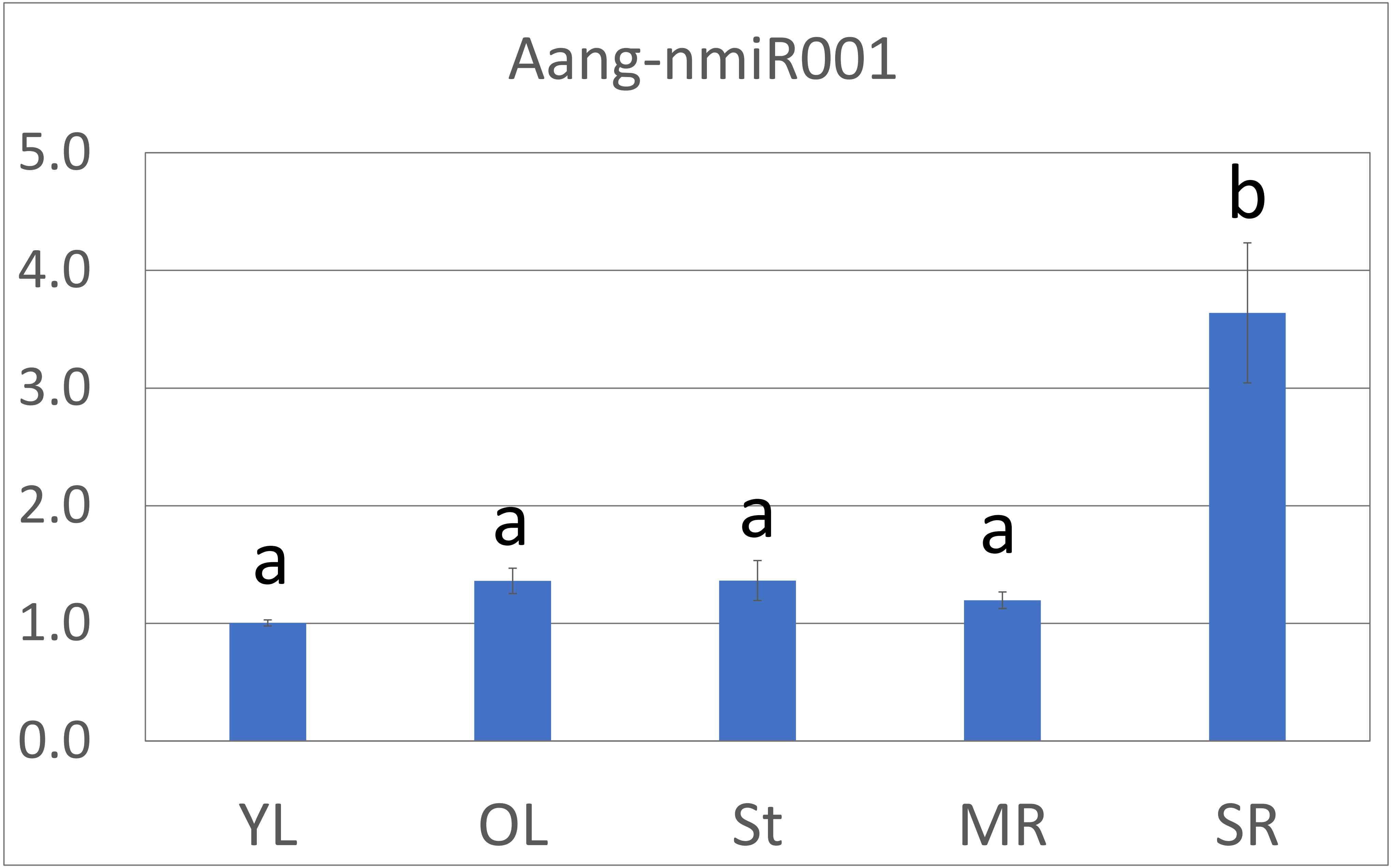

Aang-nmiR001
5.0
4.0
3.0
2.0
1.0
0.0
a
a
a
a
b
YL
OL
St
MR
SR

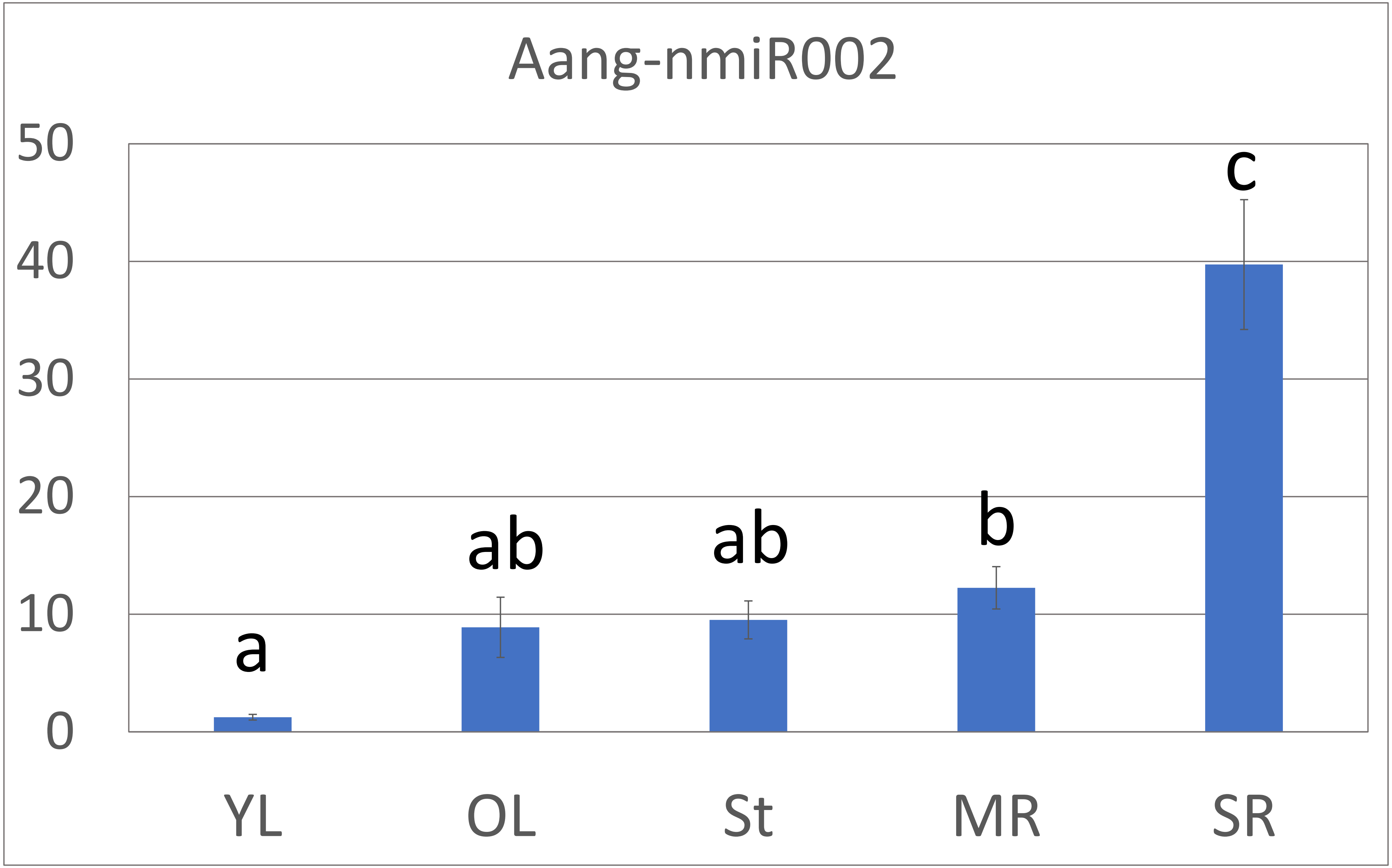
Aang-nmiR002
50
40
30
20
10
0
a
ab
ab
b
c
YL
OL
St
MR
SR

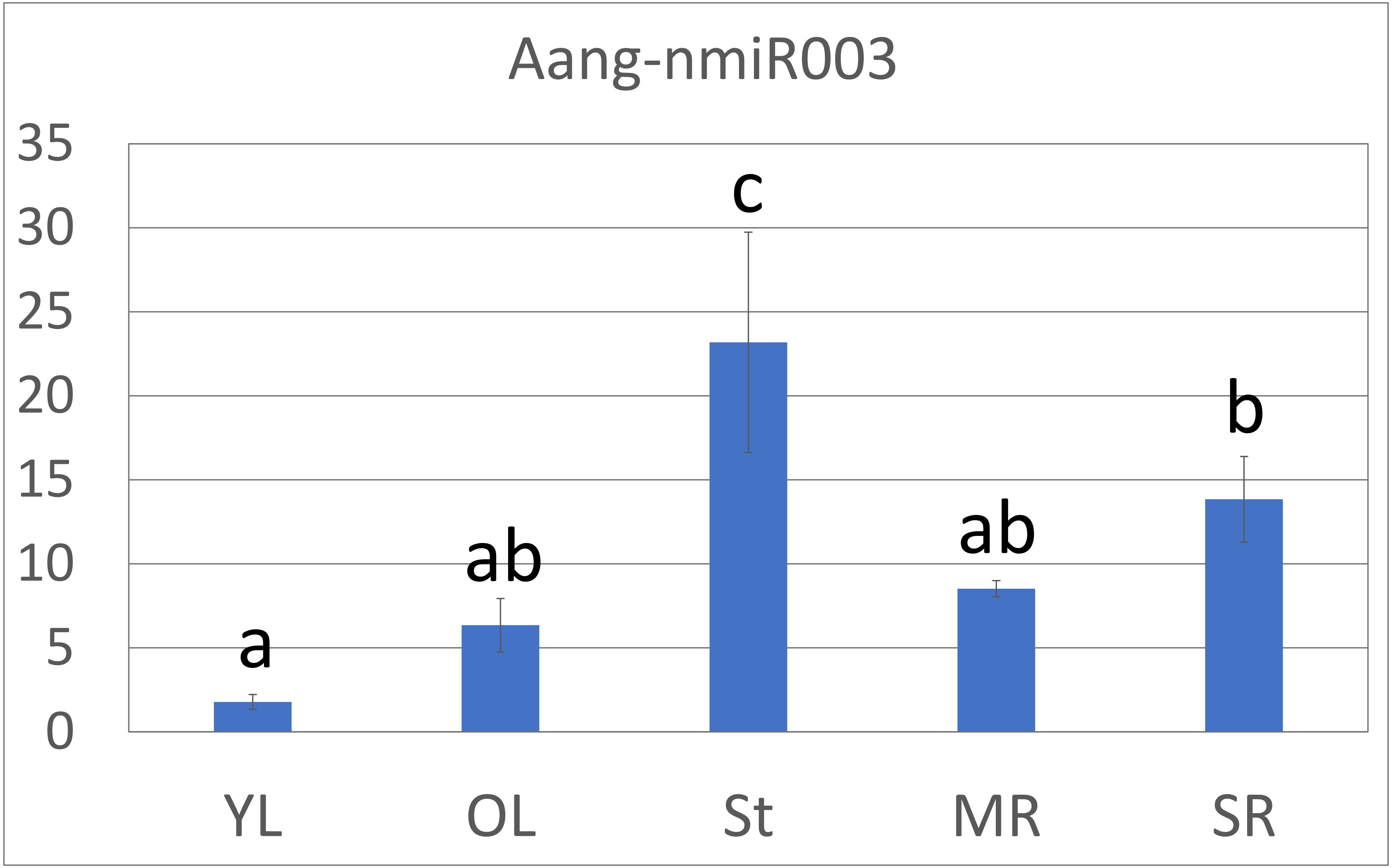
Aang-nmiR003
35
30
25
20
15
10
5
0
a
ab
c
ab
b
YL
OL
St
MR
SR

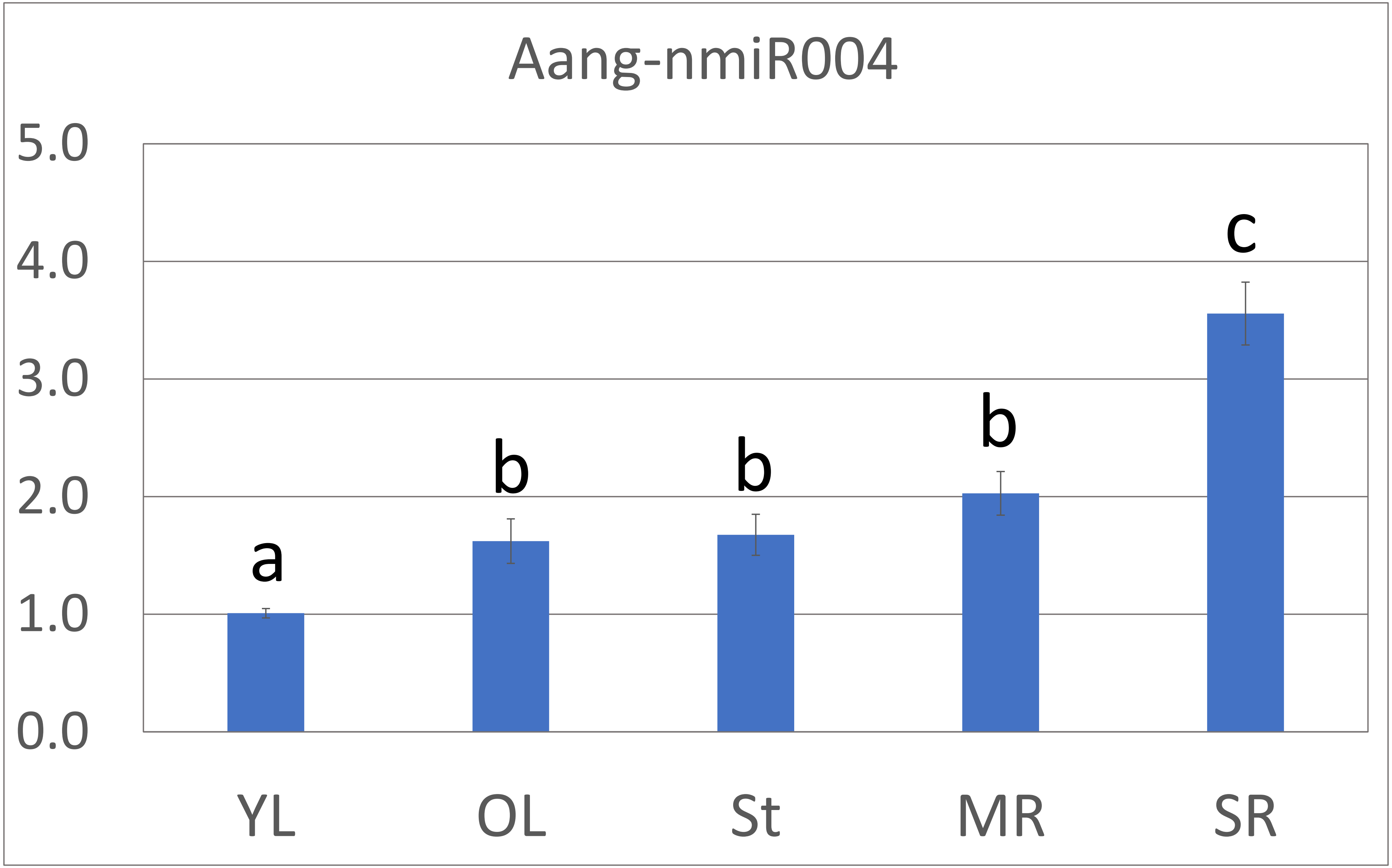
Aang-nmiR004
5.0
4.0
3.0
2.0
1.0
0.0
a
b
b
b
c
YL
OL
St
MR
SR

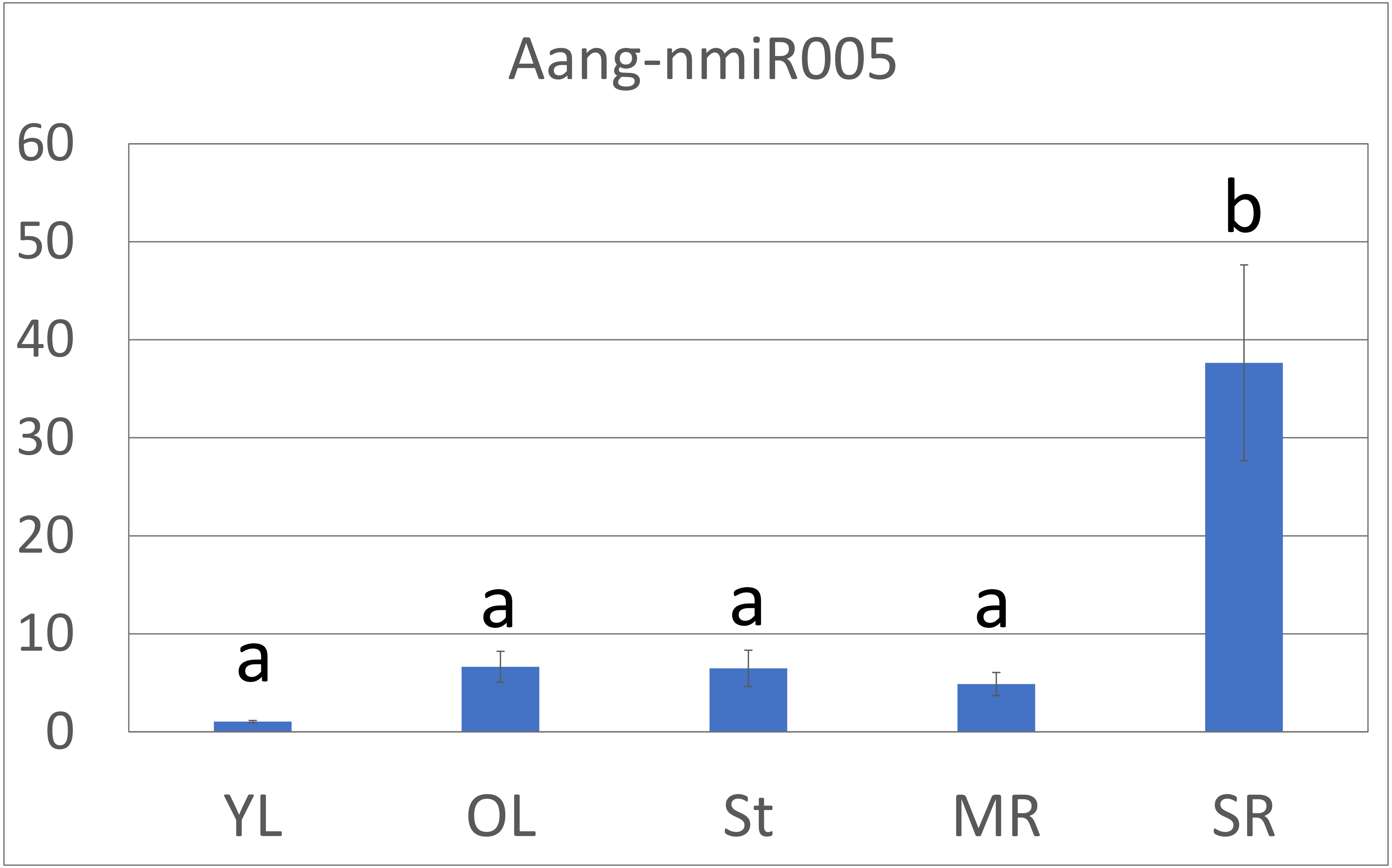
Aang-nmiR005
60
50
40
30
20
10
0
a
a
a
a
b
YL
OL
St
MR
SR

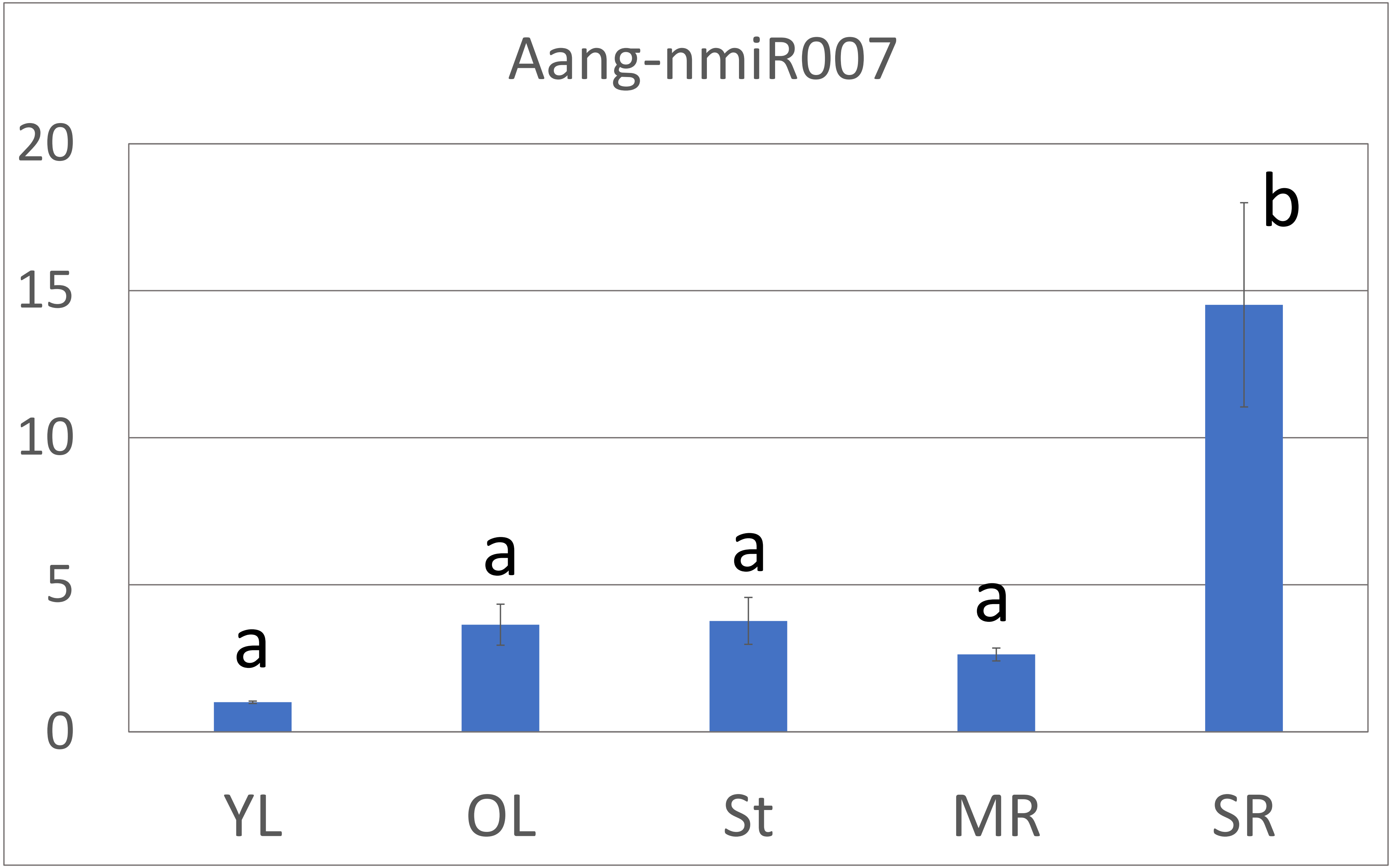
Aang-nmiR007
20
15
10
5
0
a
a
a
a
b
YL
OL
St
MR
SR

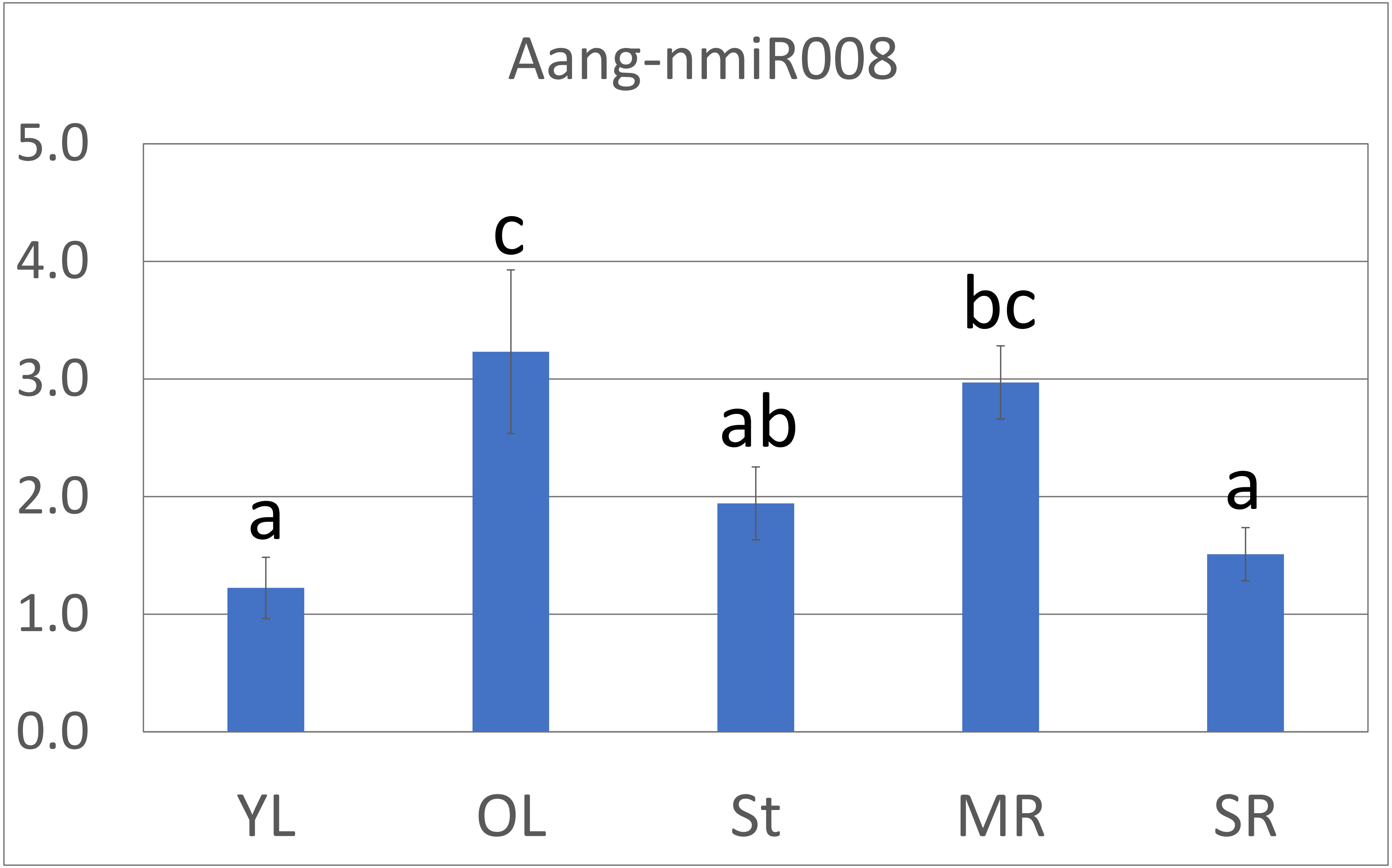

Aang-nmiR008
5.0
4.0
3.0
2.0
1.0
0.0
a
c
ab
bc
a
YL
OL
St
MR
SR

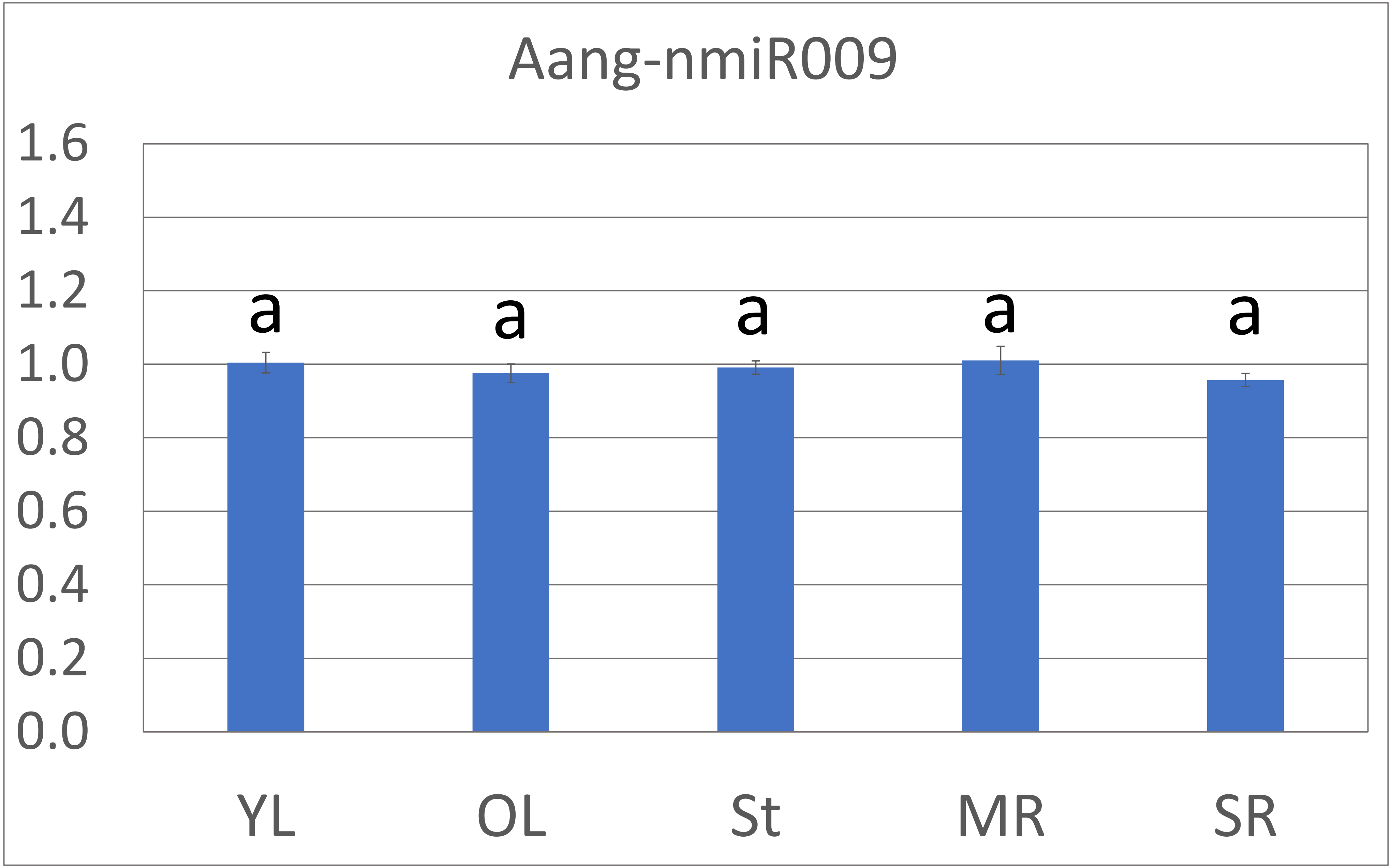

Aang-nmiR009
1.6
1.4
1.2
1.0
0.8
0.6
0.4
0.2
0.0
a
a
a
a
a
YL
OL
St
MR
SR

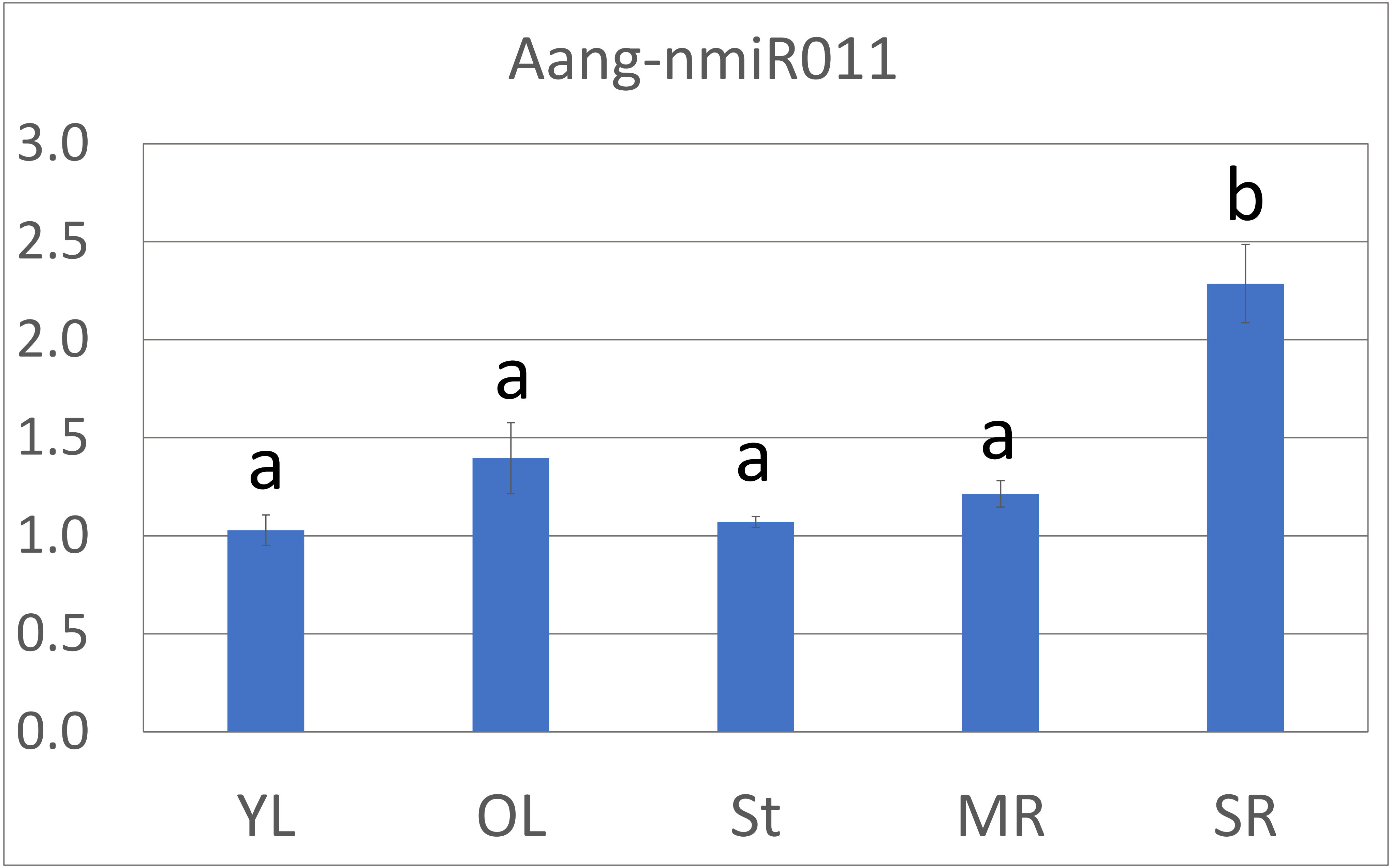
Aang-nmiR011
3.0
2.5
2.0
1.5
1.0
0.5
0.0
a
a
a
a
b
YL
OL
St
MR
SR

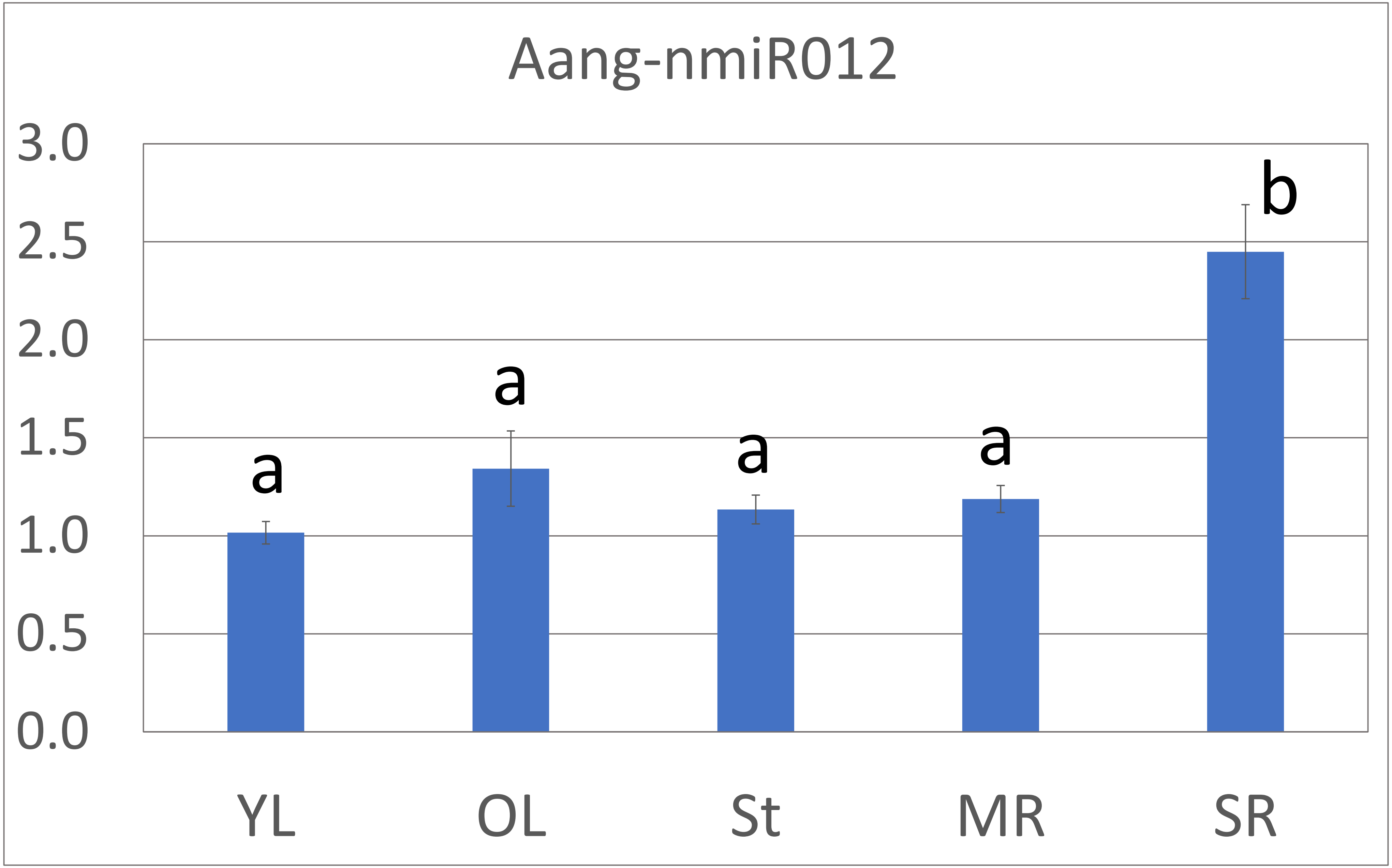
Aang-nmiR012
3.0
2.5
2.0
1.5
1.0
0.5
0.0
a
a
a
a
b
YL
OL
St
MR
SR

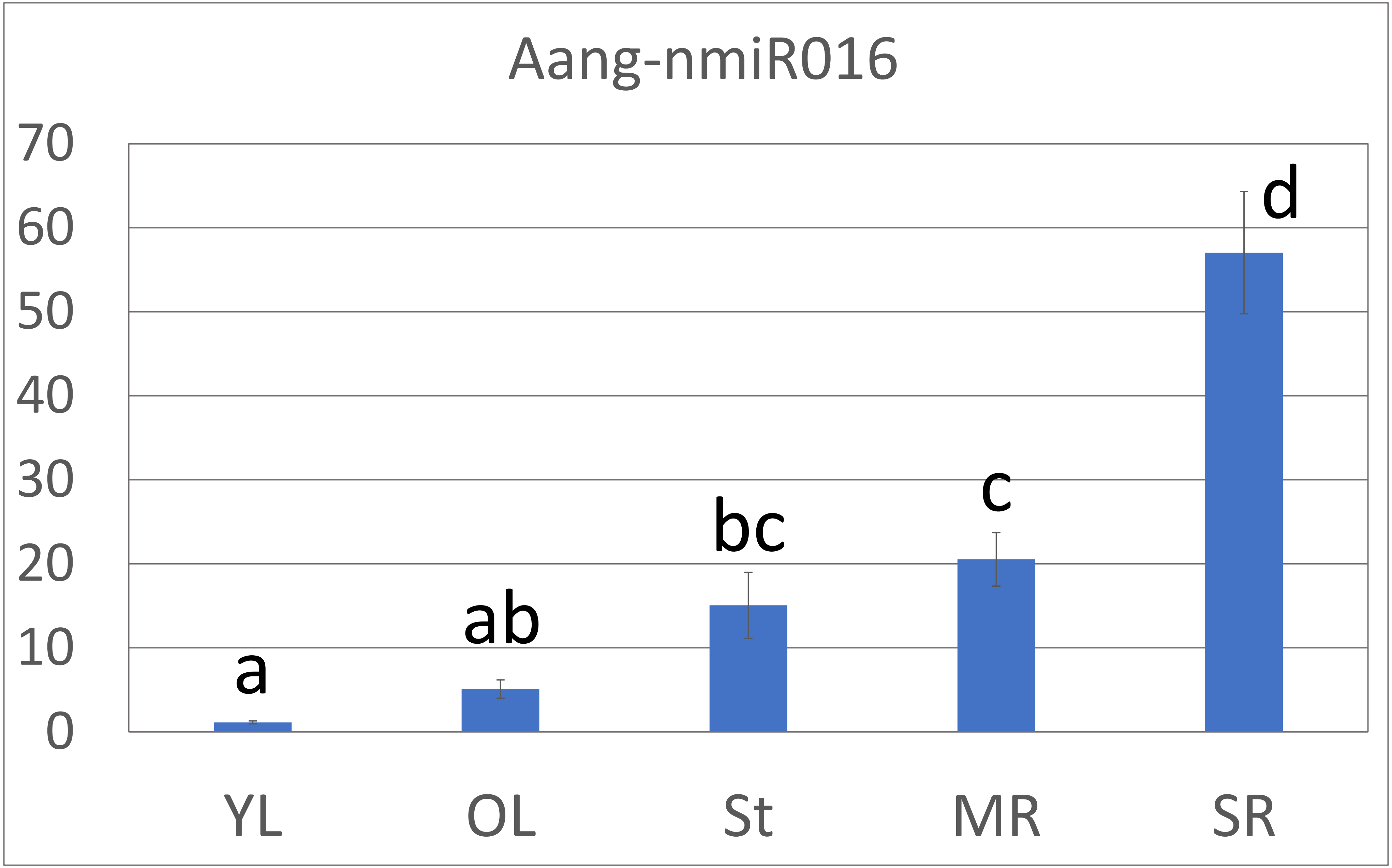

Aang-nmiR016
70
60
50
40
30
20
10
0
a
ab
bc
c
d
YL
OL
St
MR
SR

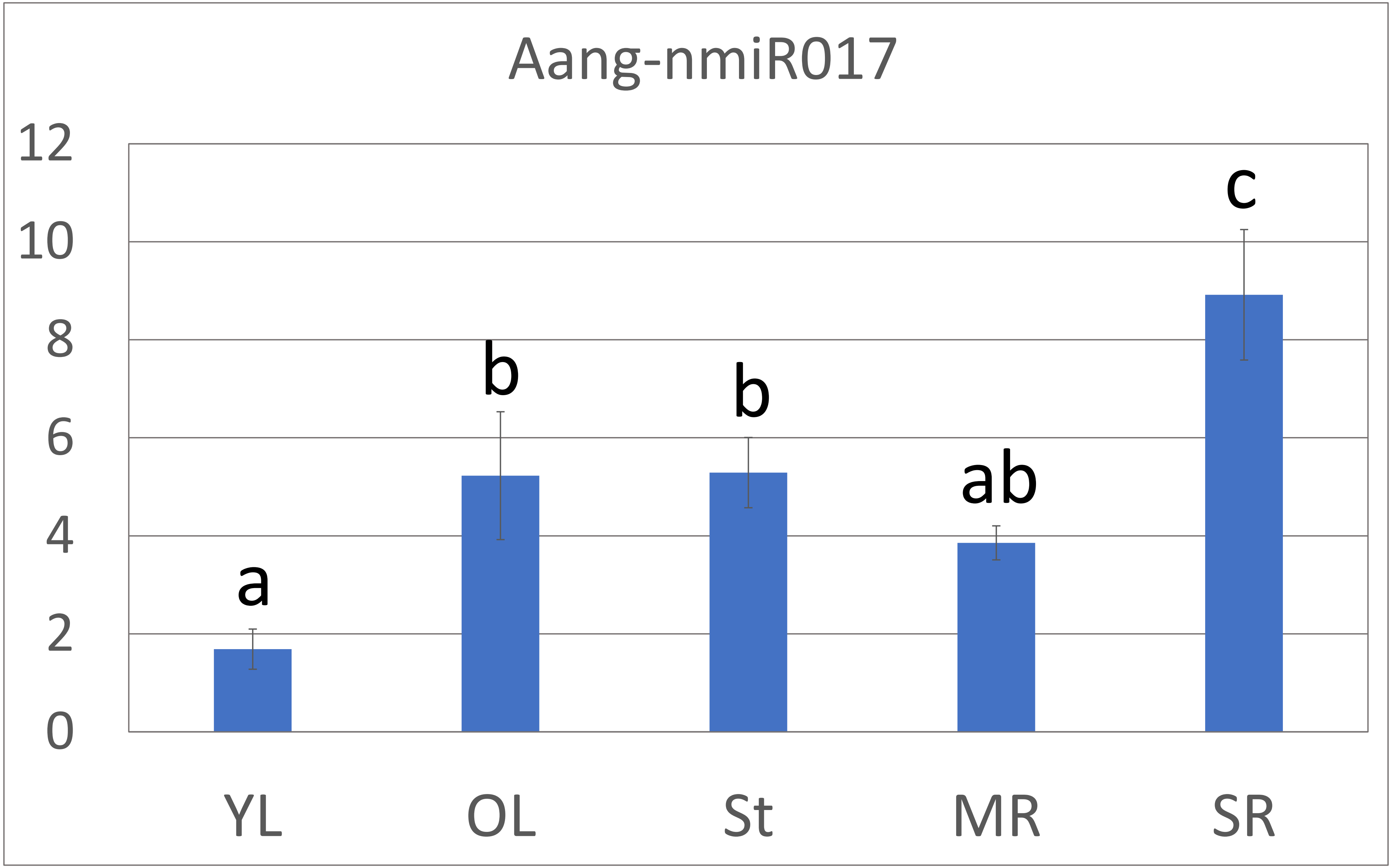
Aang-nmiR017
12
10
8
6
4
2
0
a
b
b
ab
c
YL
OL
St
MR
SR

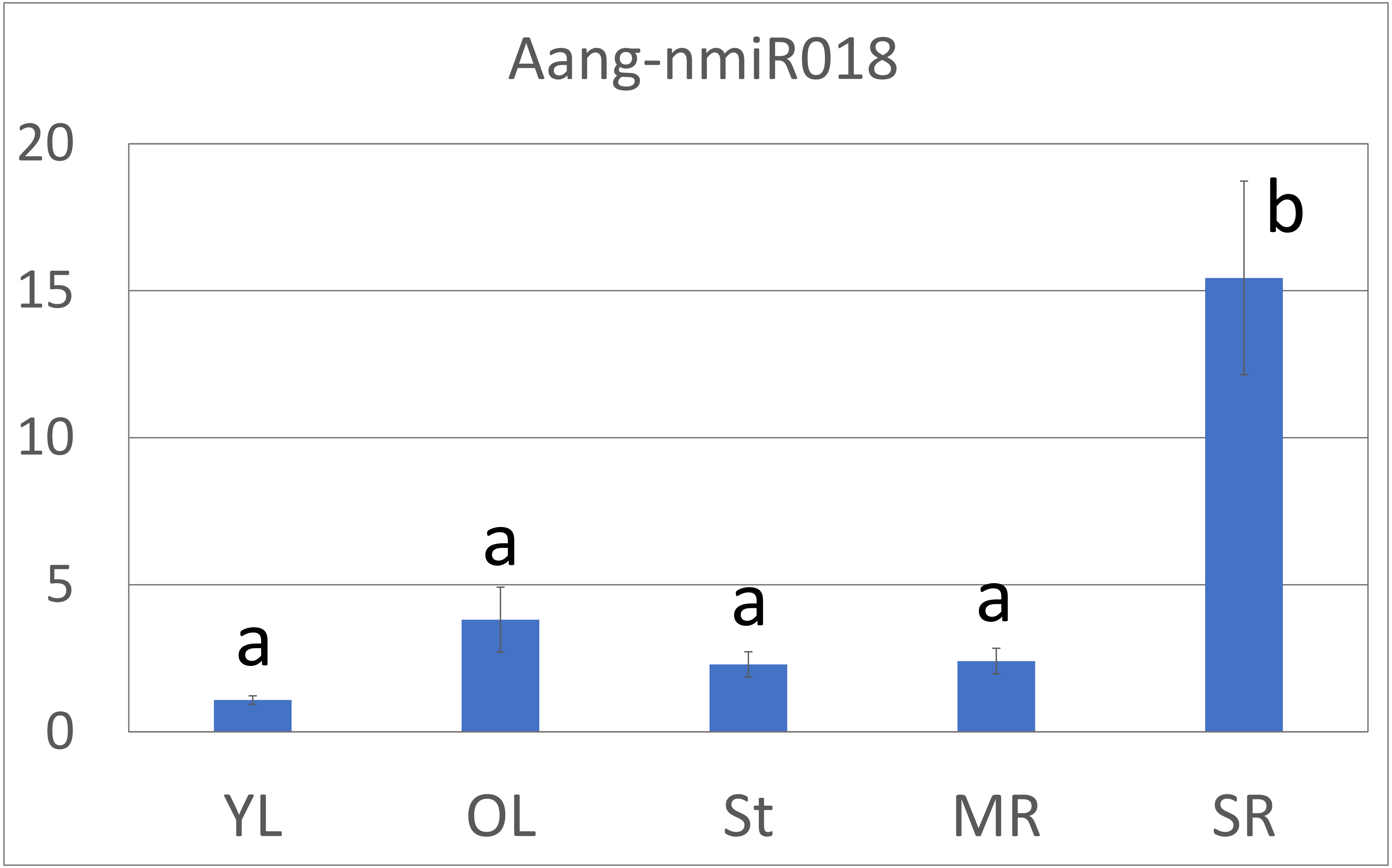
Aang-nmiR018
20
15
10
5
0
a
a
a
a
b
YL
OL
St
MR
SR

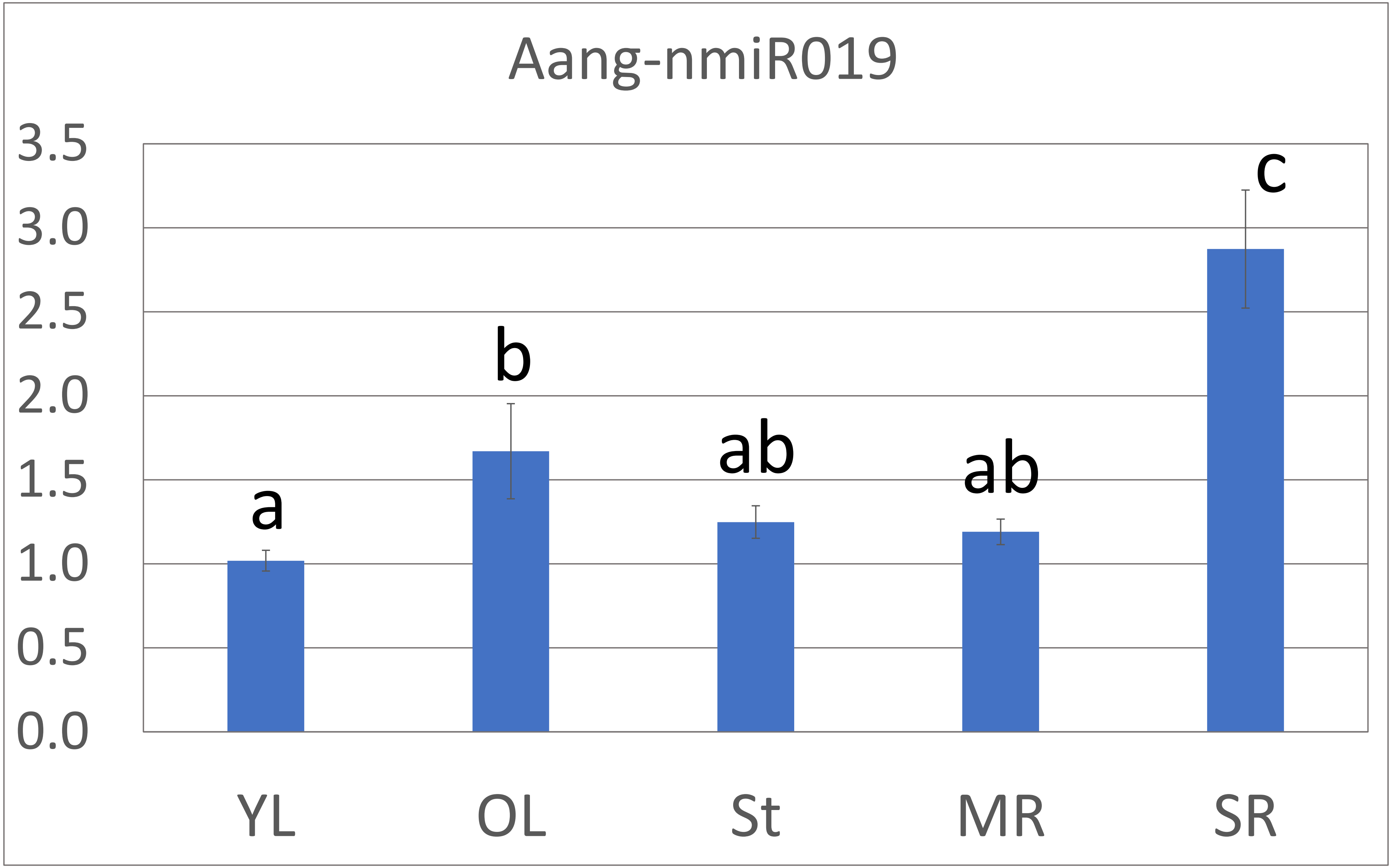
Aang-nmiR019
3.5
3.0
2.5
2.0
1.5
1.0
0.5
0.0
a
b
ab
ab
c
YL
OL
St
MR
SR

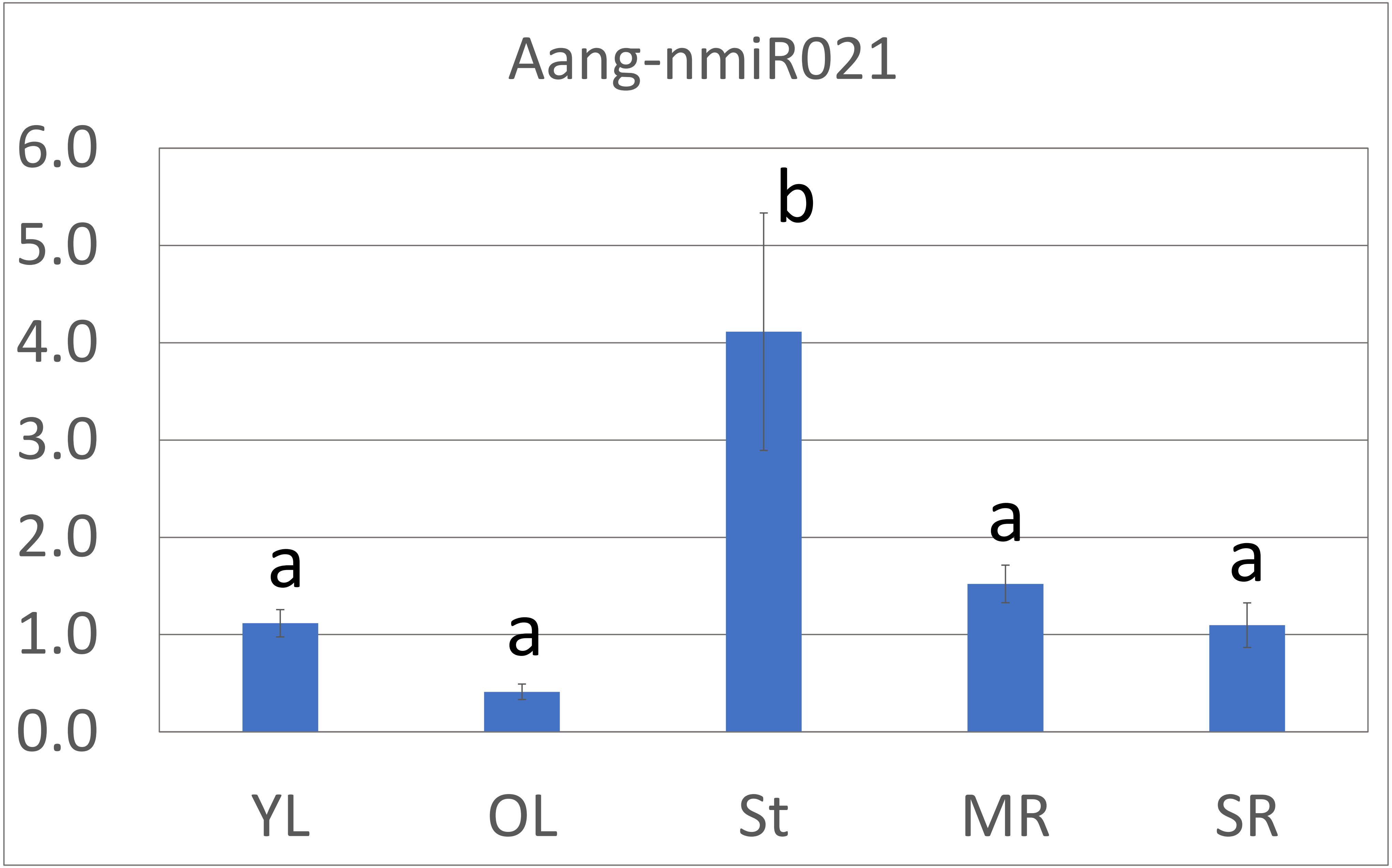
Aang-nmiR021
6.0
5.0
4.0
3.0
2.0
1.0
0.0
a
a
b
a
a
YL
OL
St
MR
SR

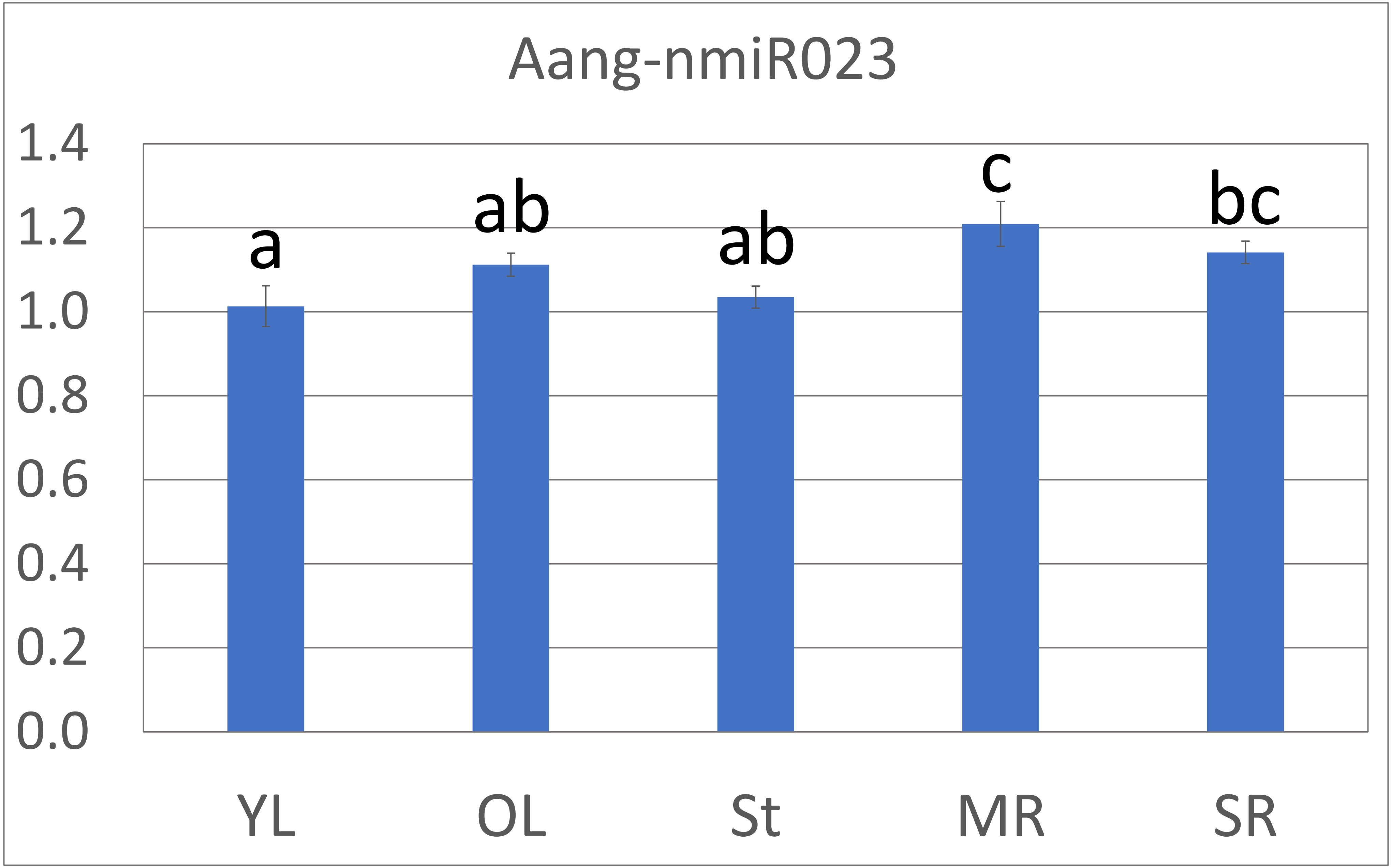

Aang-nmiR023
1.4
1.2
1.0
0.8
0.6
0.4
0.2
0.0
a
ab
ab
c
bc
YL
OL
St
MR
SR

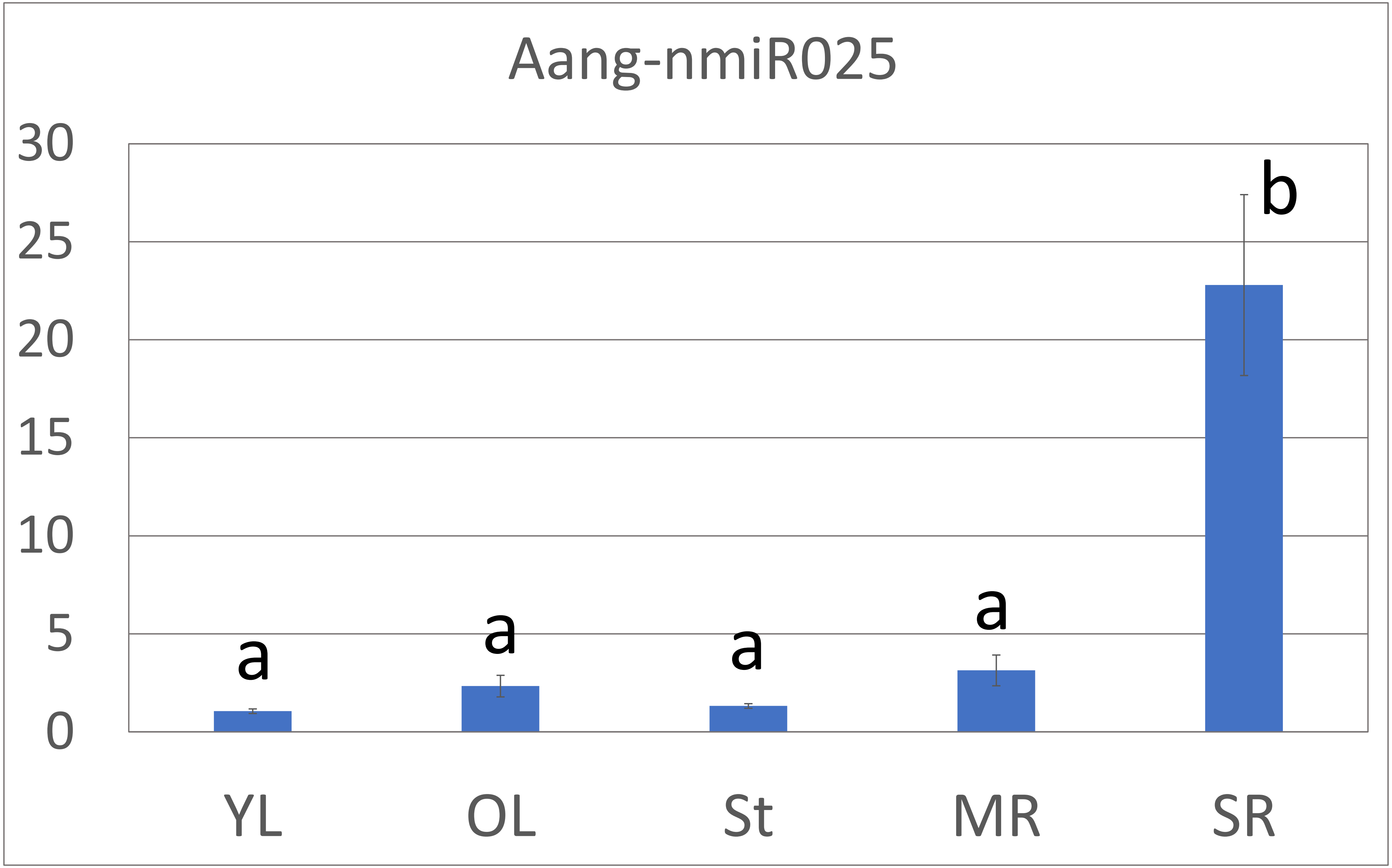
Aang-nmiR025
30
25
20
15
10
5
0
a
a
a
a
b
YL
OL
St
MR
SR

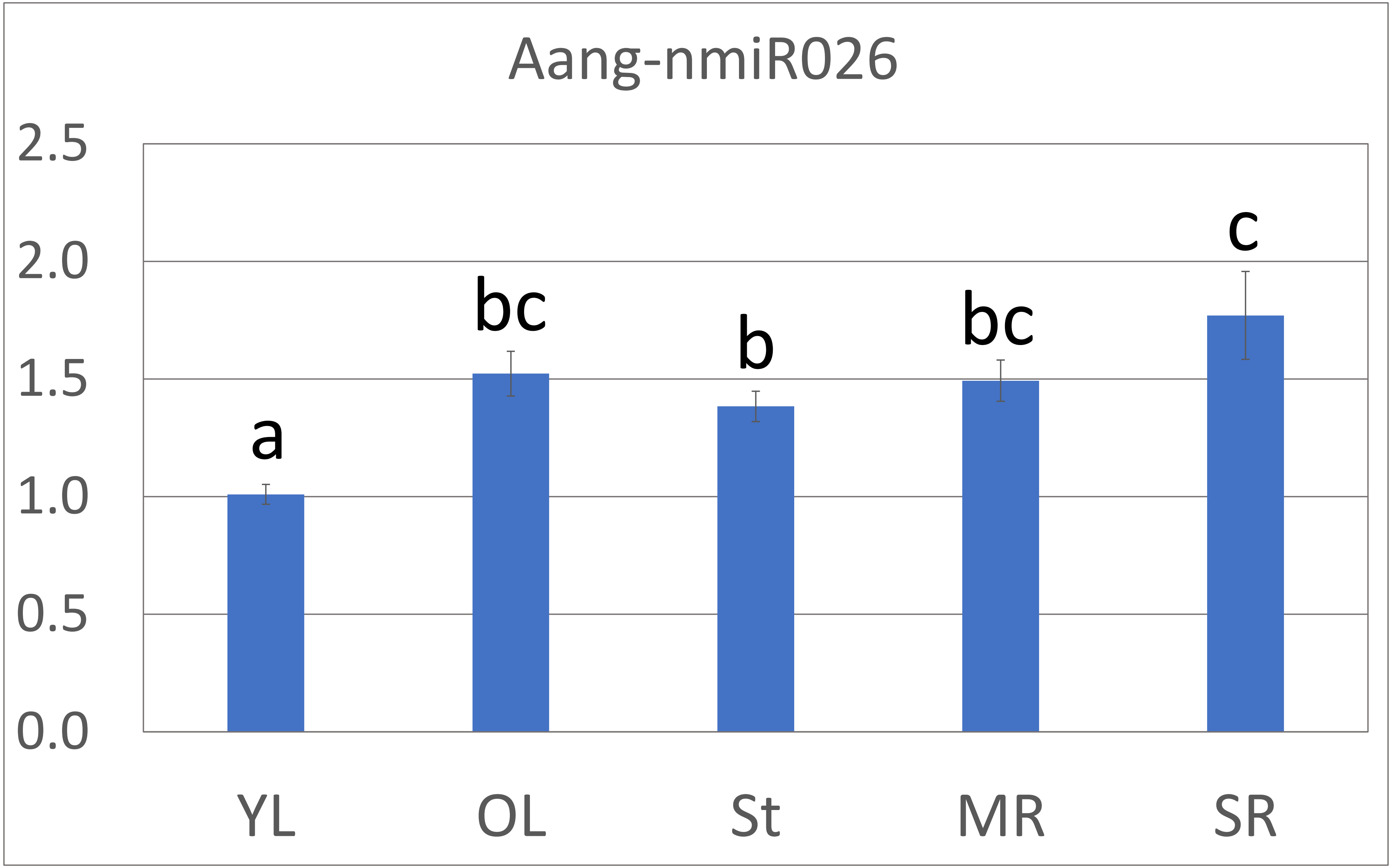

Aang-nmiR026
2.5
2.0
1.5
1.0
0.5
0.0
a
bc
b
bc
c
YL
OL
St
MR
SR

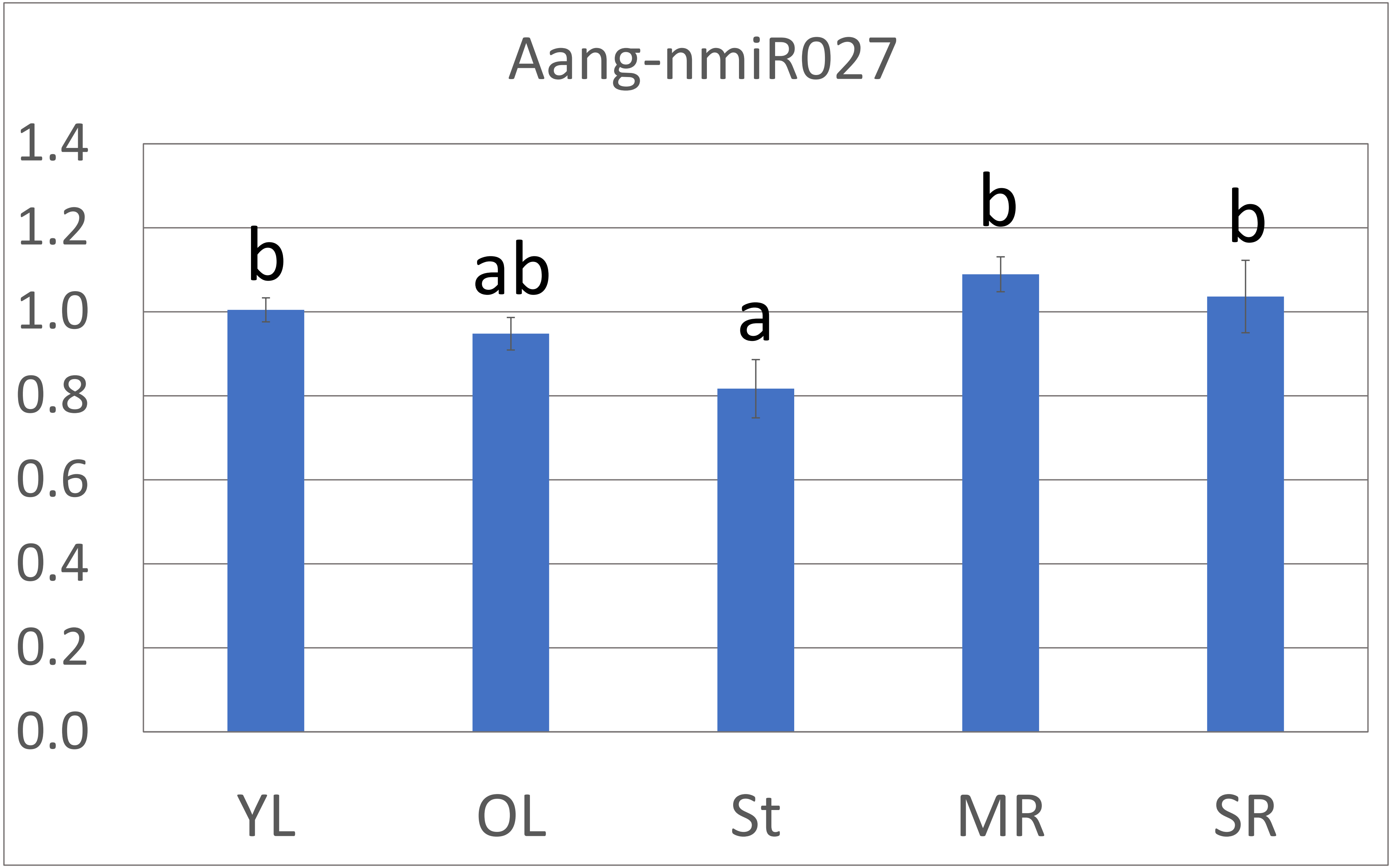

Aang-nmiR027
1.4
1.2
1.0
0.8
0.6
0.4
0.2
0.0
b
ab
a
b
b
YL
OL
St
MR
SR

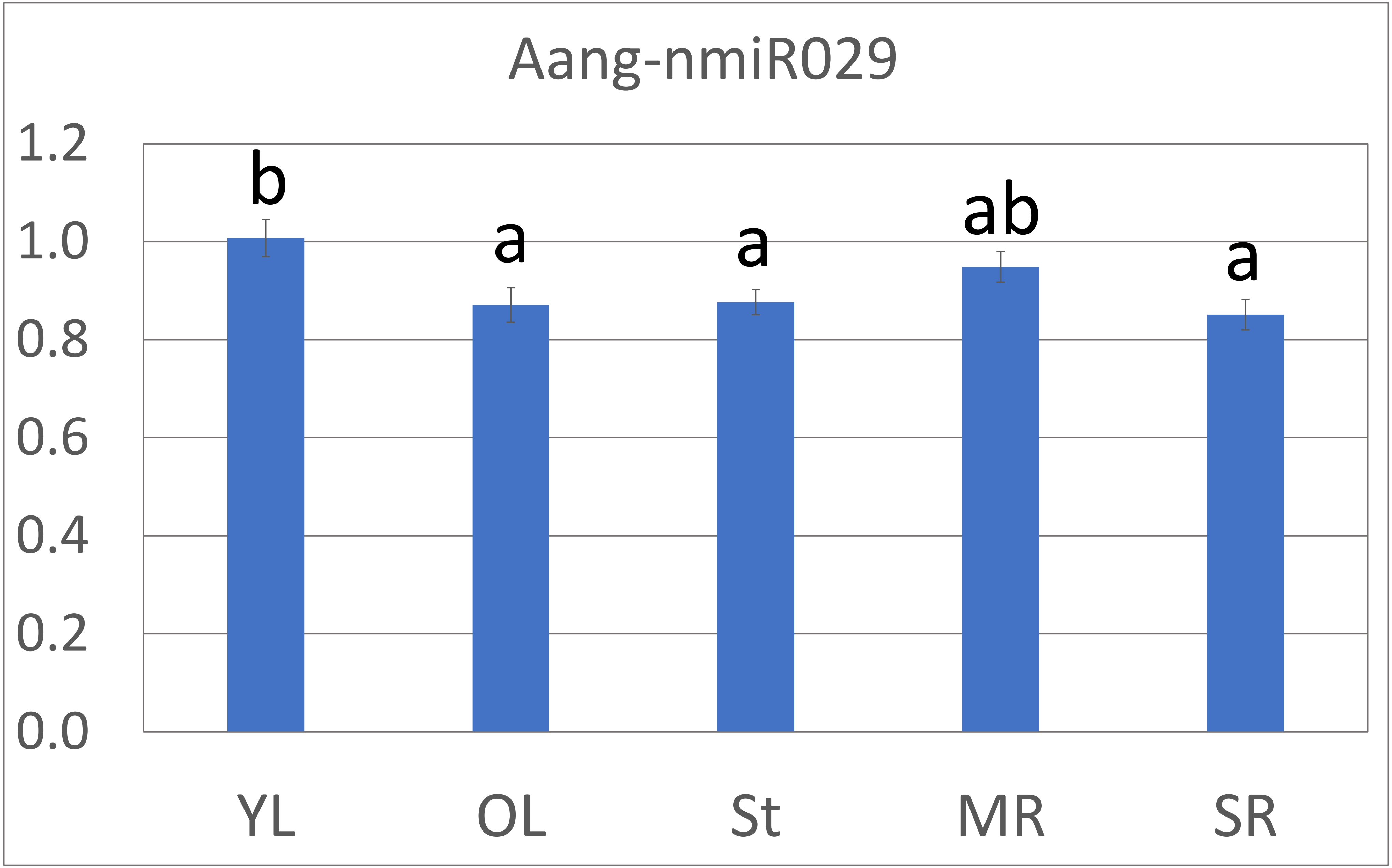

Aang-nmiR029
1.2
1.0
0.8
0.6
0.4
0.2
0.0
b
a
a
ab
a
YL
OL
St
MR
SR

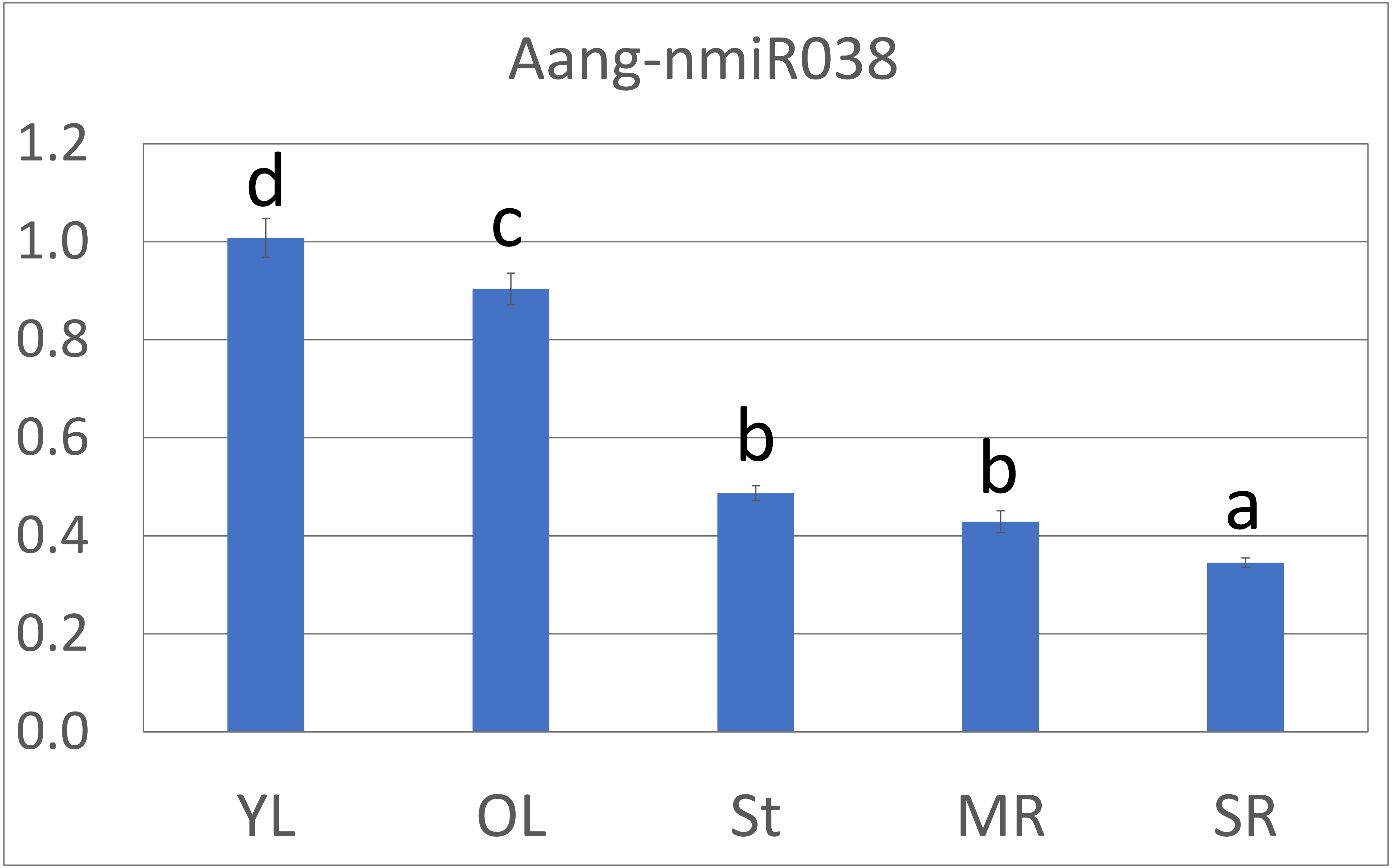

Aang-nmiR038
1.2
1.0
0.8
0.6
0.4
0.2
0.0
d
c
b
b
a
YL
OL
St
MR
SR

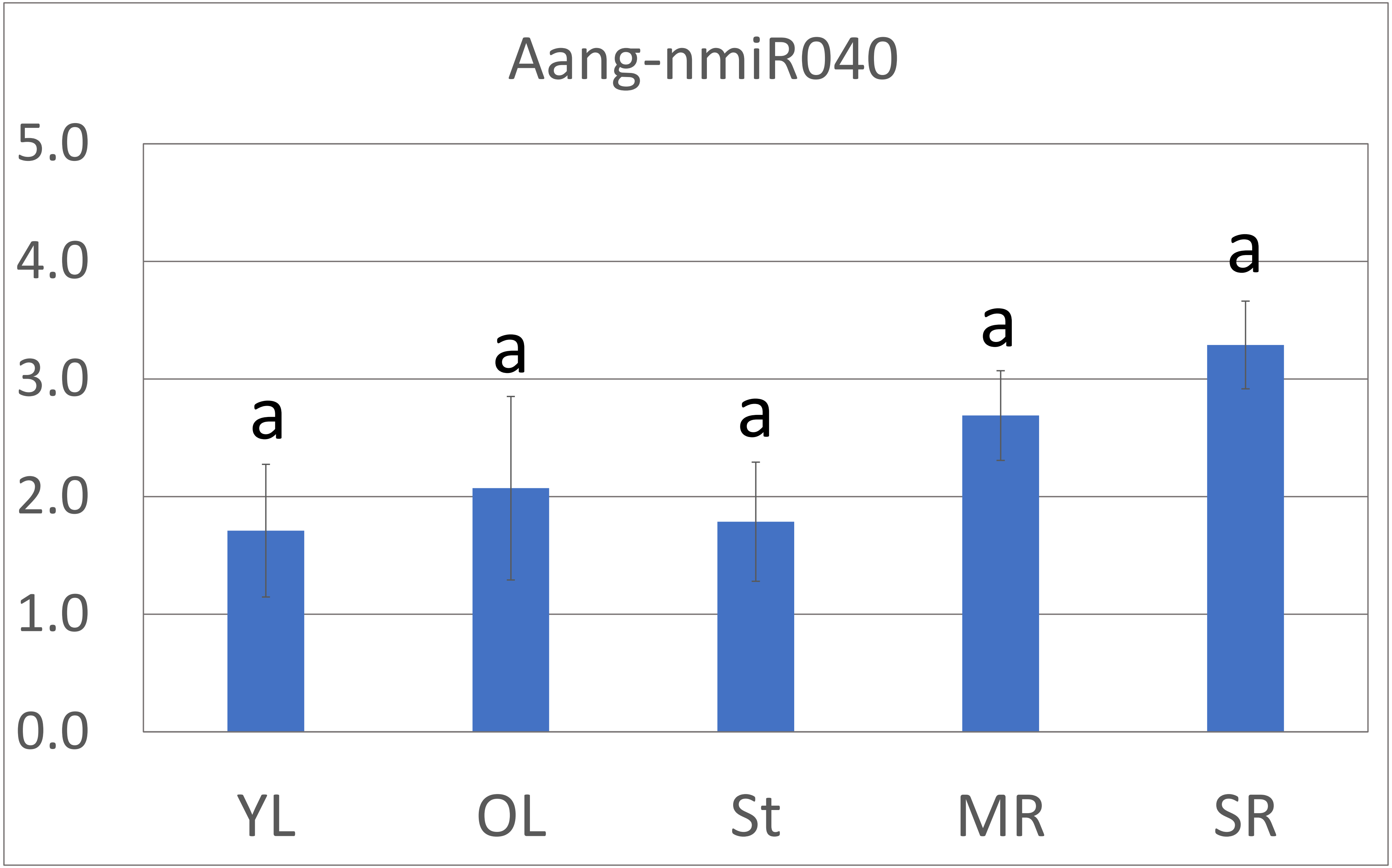

Aang-nmiR040
5.0
4.0
3.0
2.0
1.0
0.0
a
a
a
a
a
YL
OL
St
MR
SR

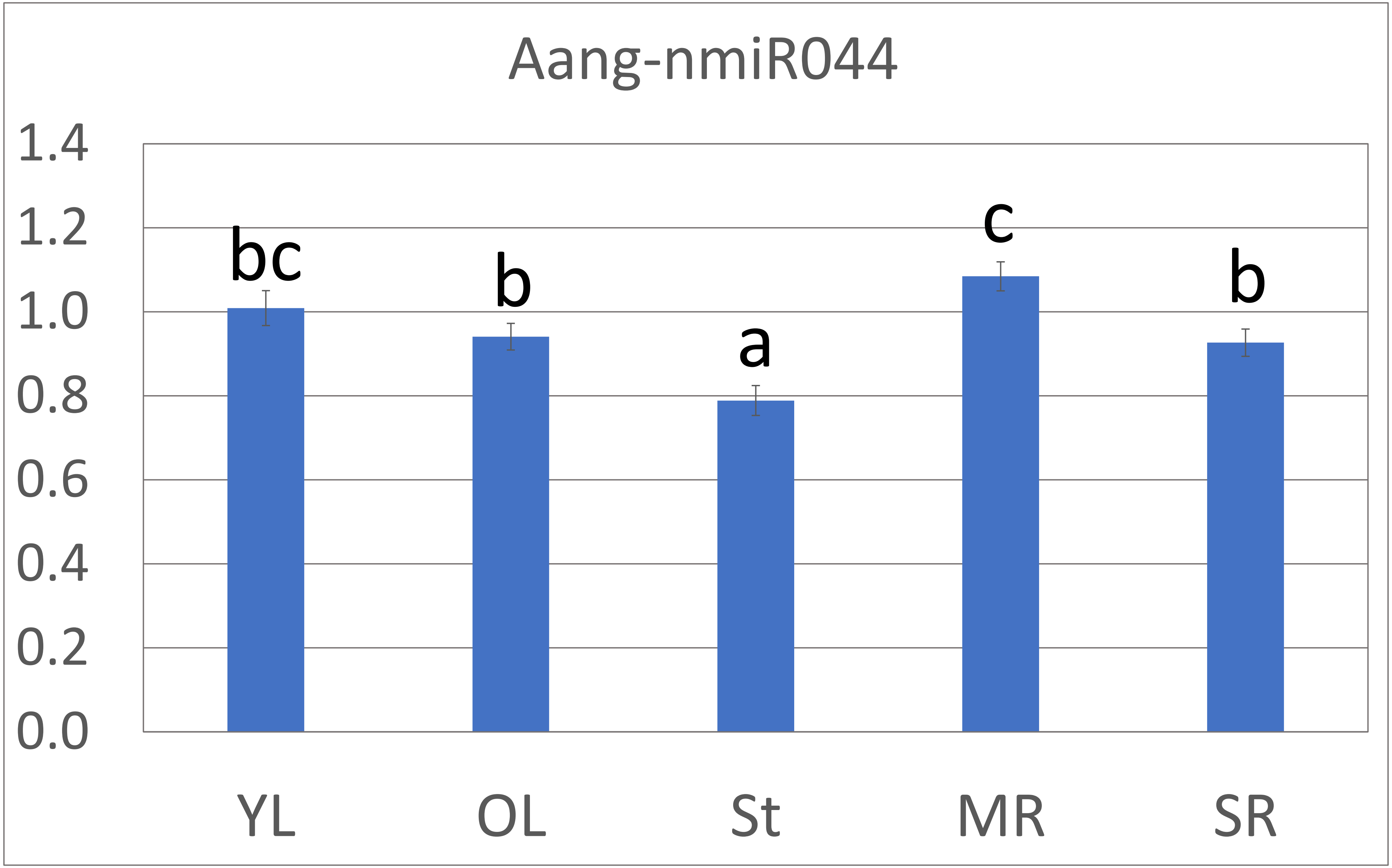

Aang-nmiR044
1.4
1.2
1.0
0.8
0.6
0.4
0.2
0.0
bc
b
a
c
b
YL
OL
St
MR
SR

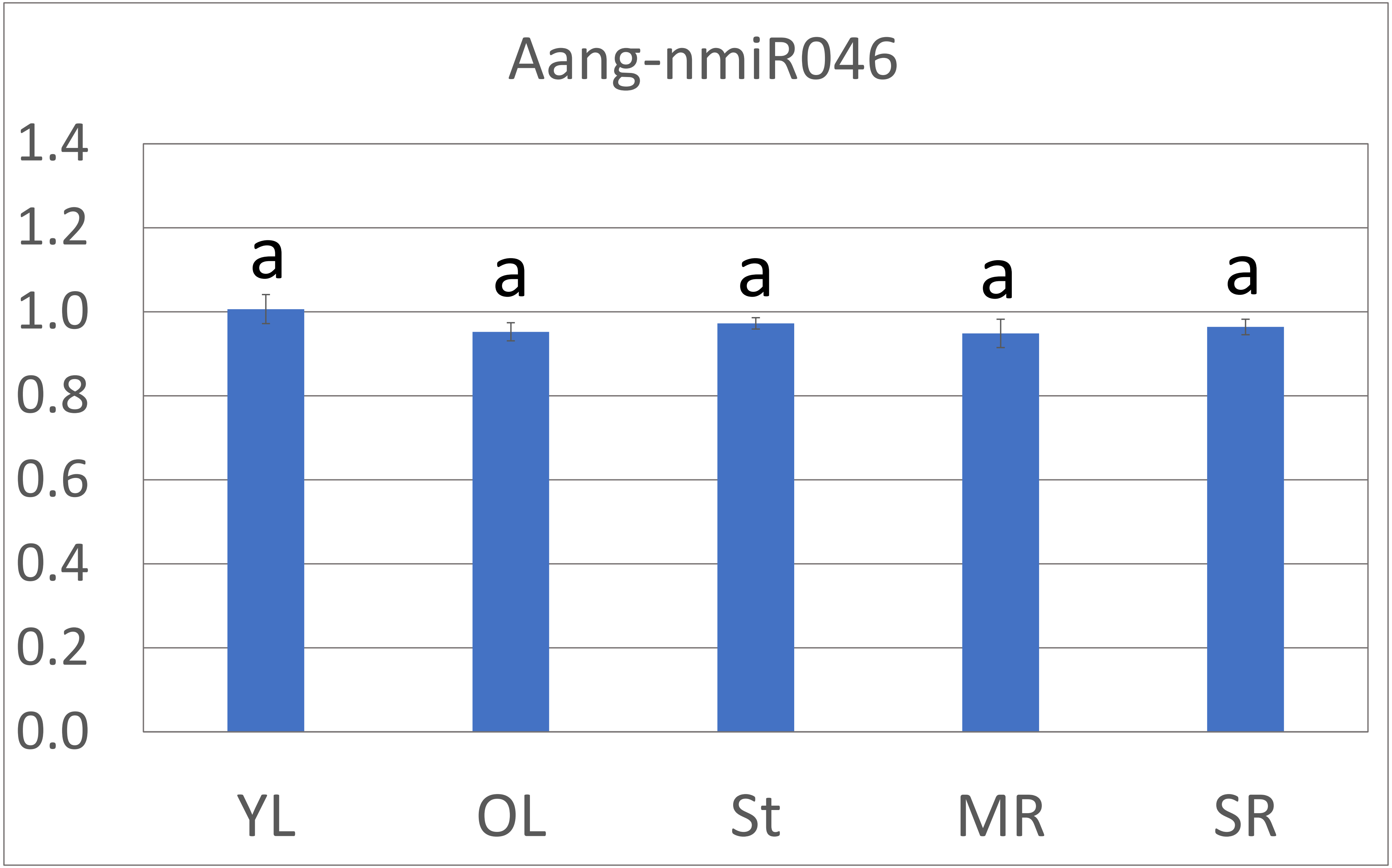
Aang-nmiR046
1.4
1.2
1.0
0.8
0.6
0.4
0.2
0.0
a
a
a
a
a
YL
OL
St
MR
SR

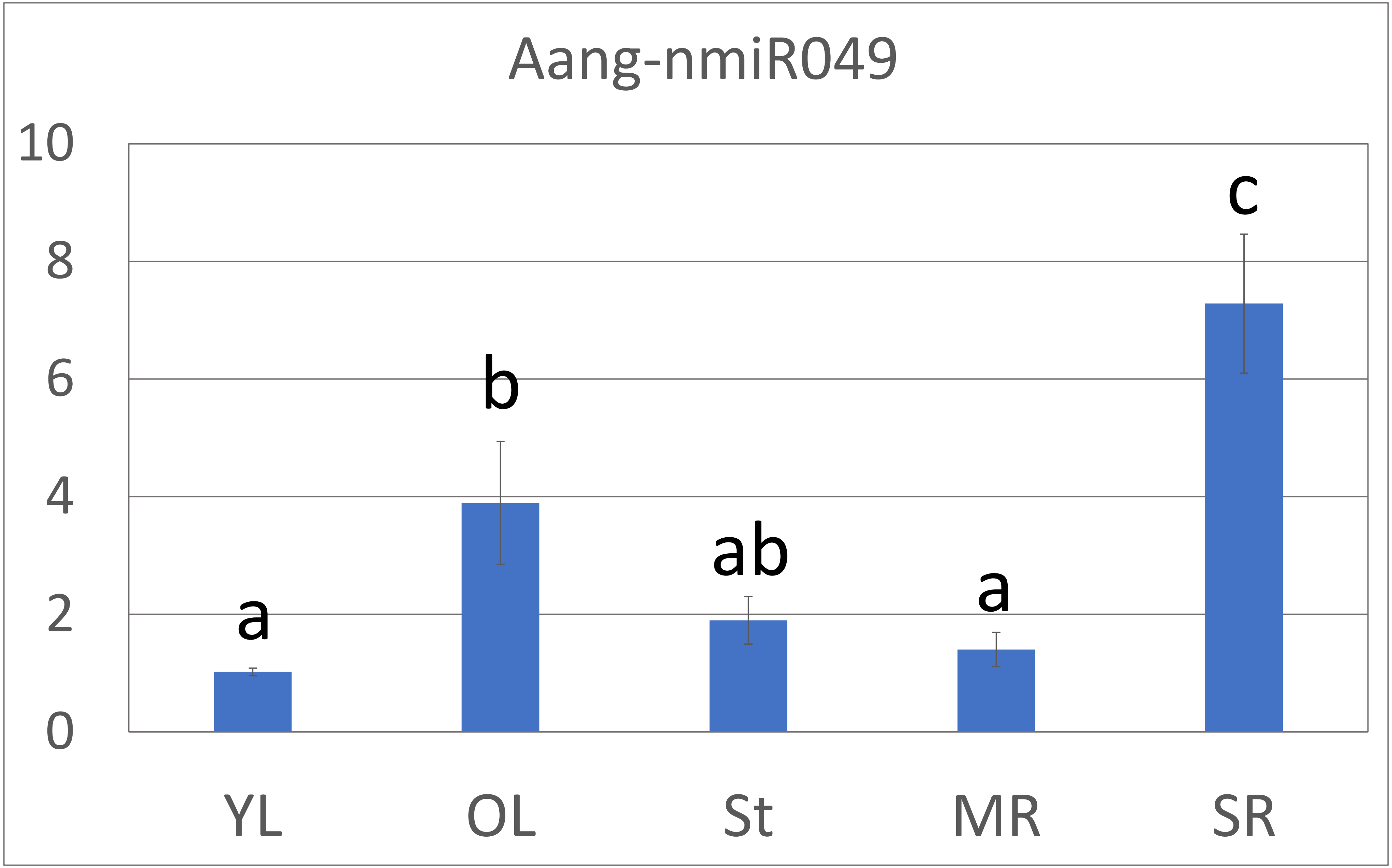
Aang-nmiR049
10
8
6
4
2
0
a
b
ab
a
c
YL
OL
St
MR
SR

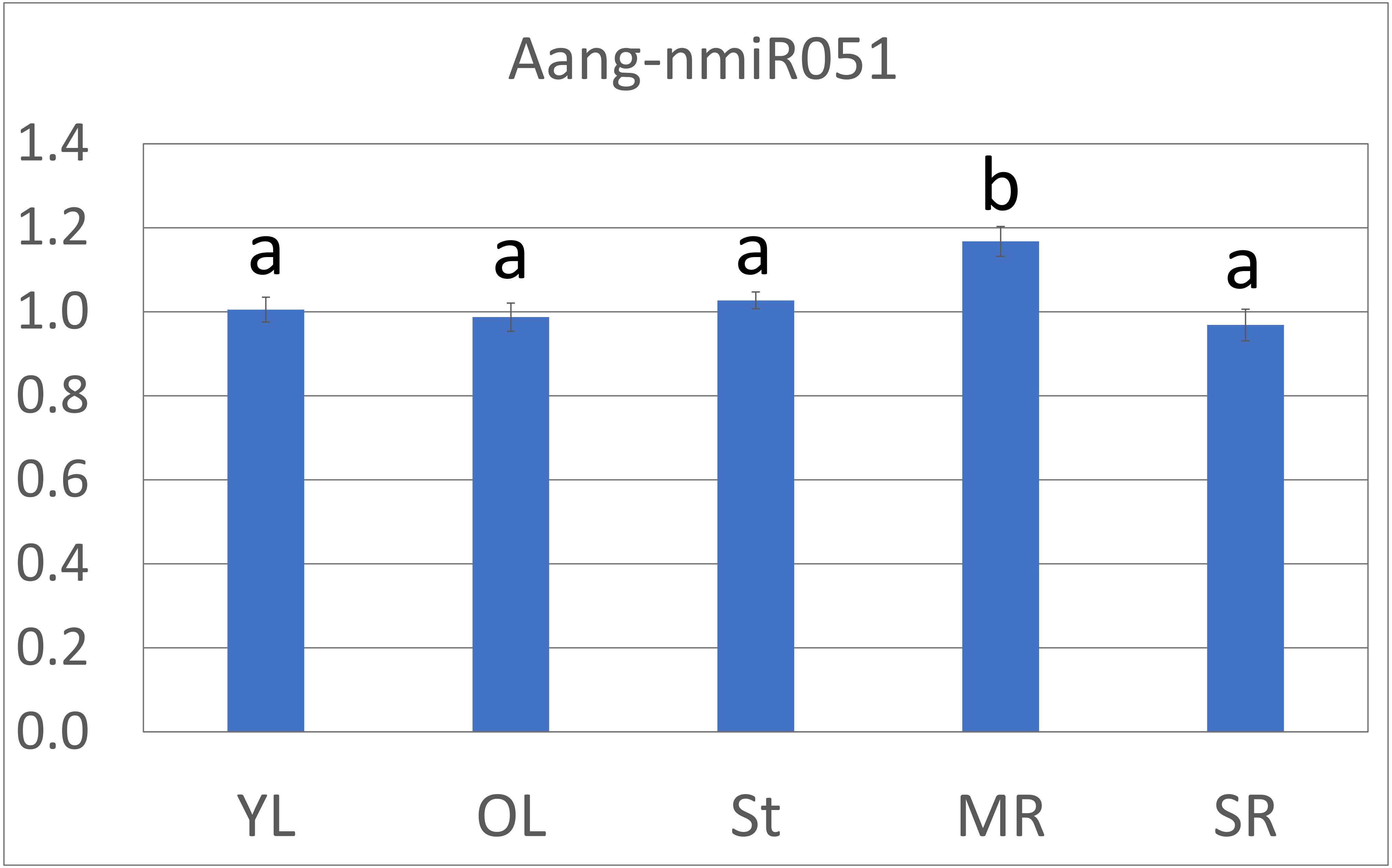
Aang-nmiR051
1.4
1.2
1.0
0.8
0.6
0.4
0.2
0.0
a
a
a
b
a
YL
OL
St
MR
SR

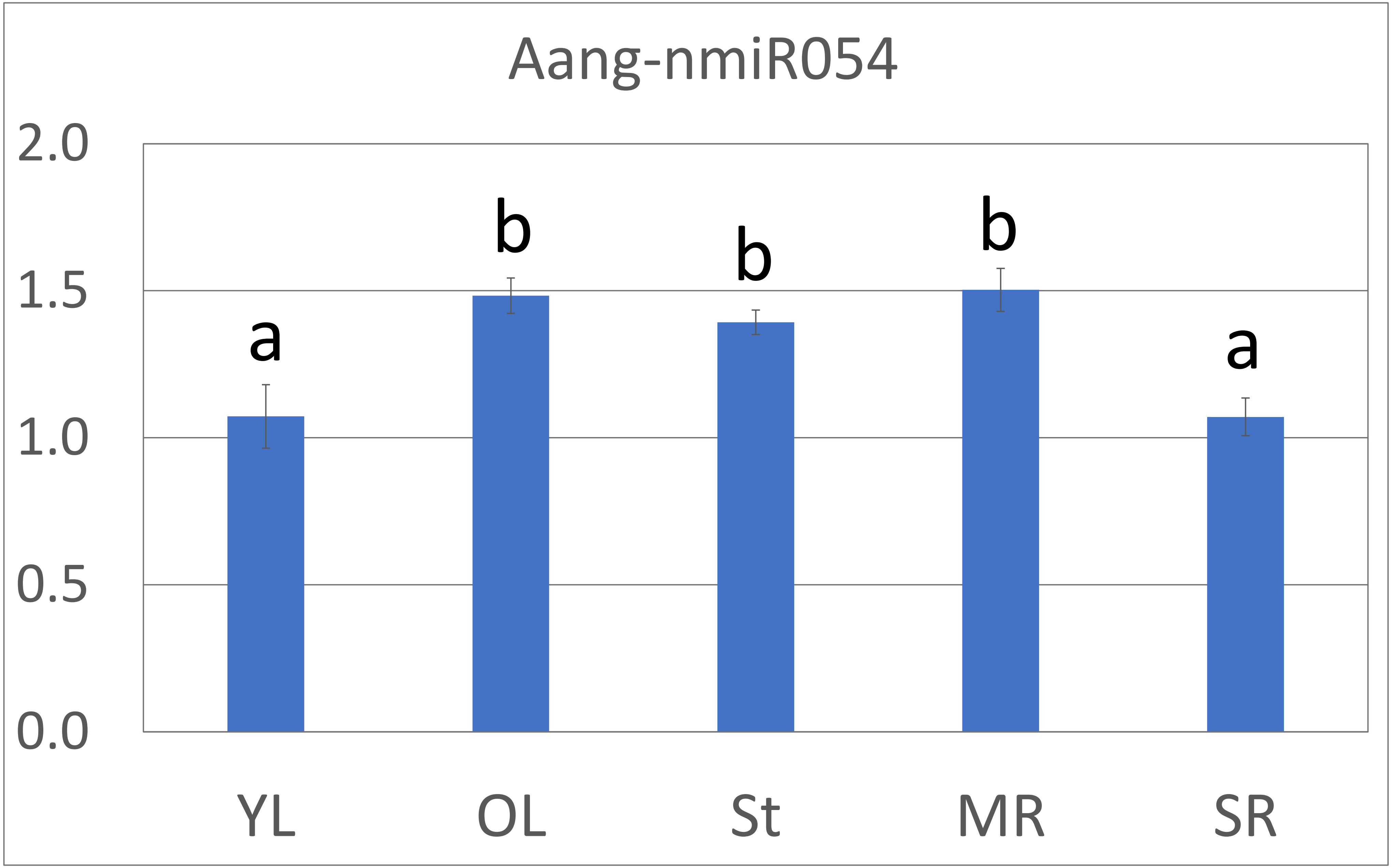
Aang-nmiR054
2.0
1.5
1.0
0.5
0.0
a
b
b
b
a
YL
OL
St
MR
SR

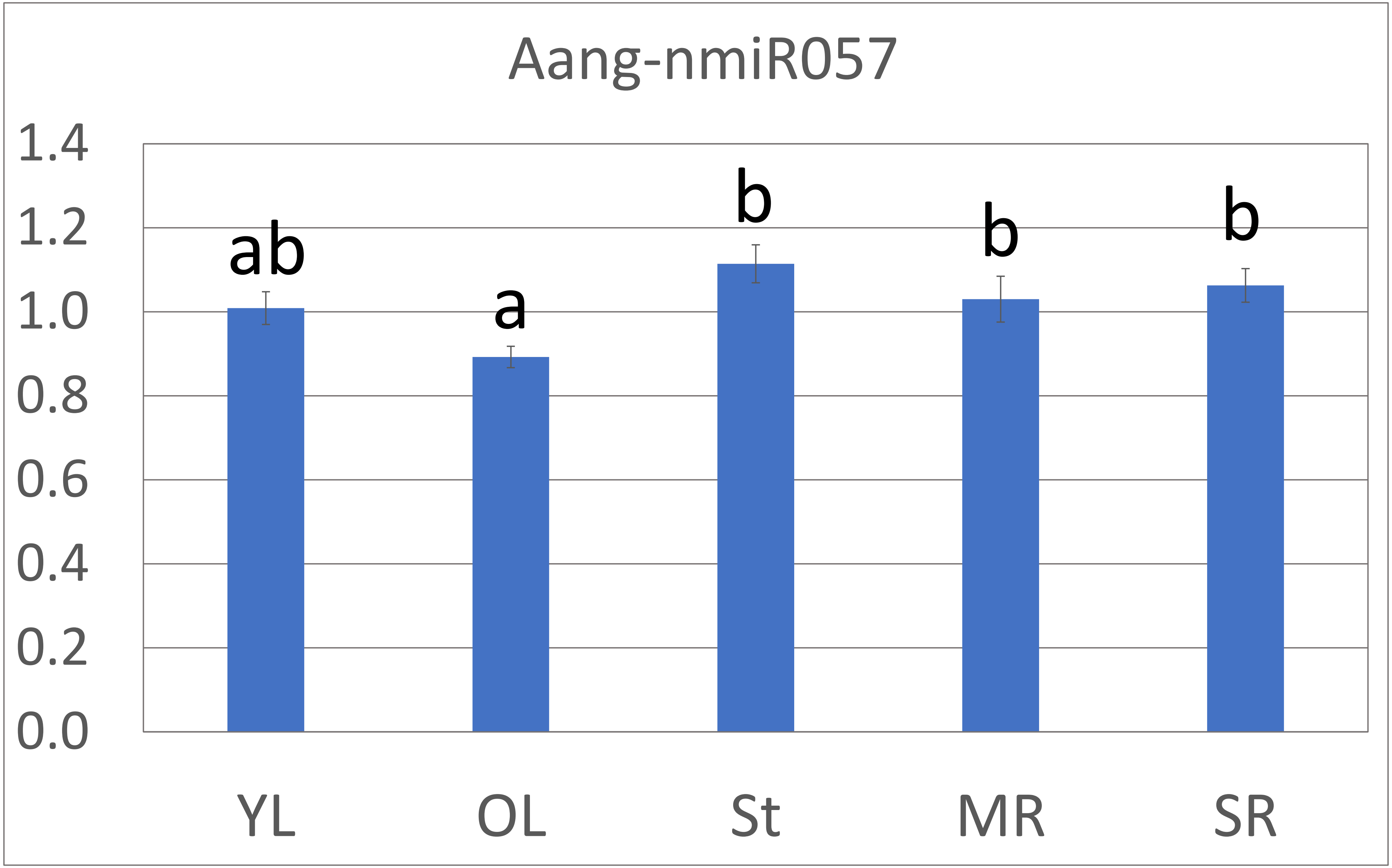
Aang-nmiR057
1.4
1.2
1.0
0.8
0.6
0.4
0.2
0.0
ab
a
b
b
b
YL
OL
St
MR
SR

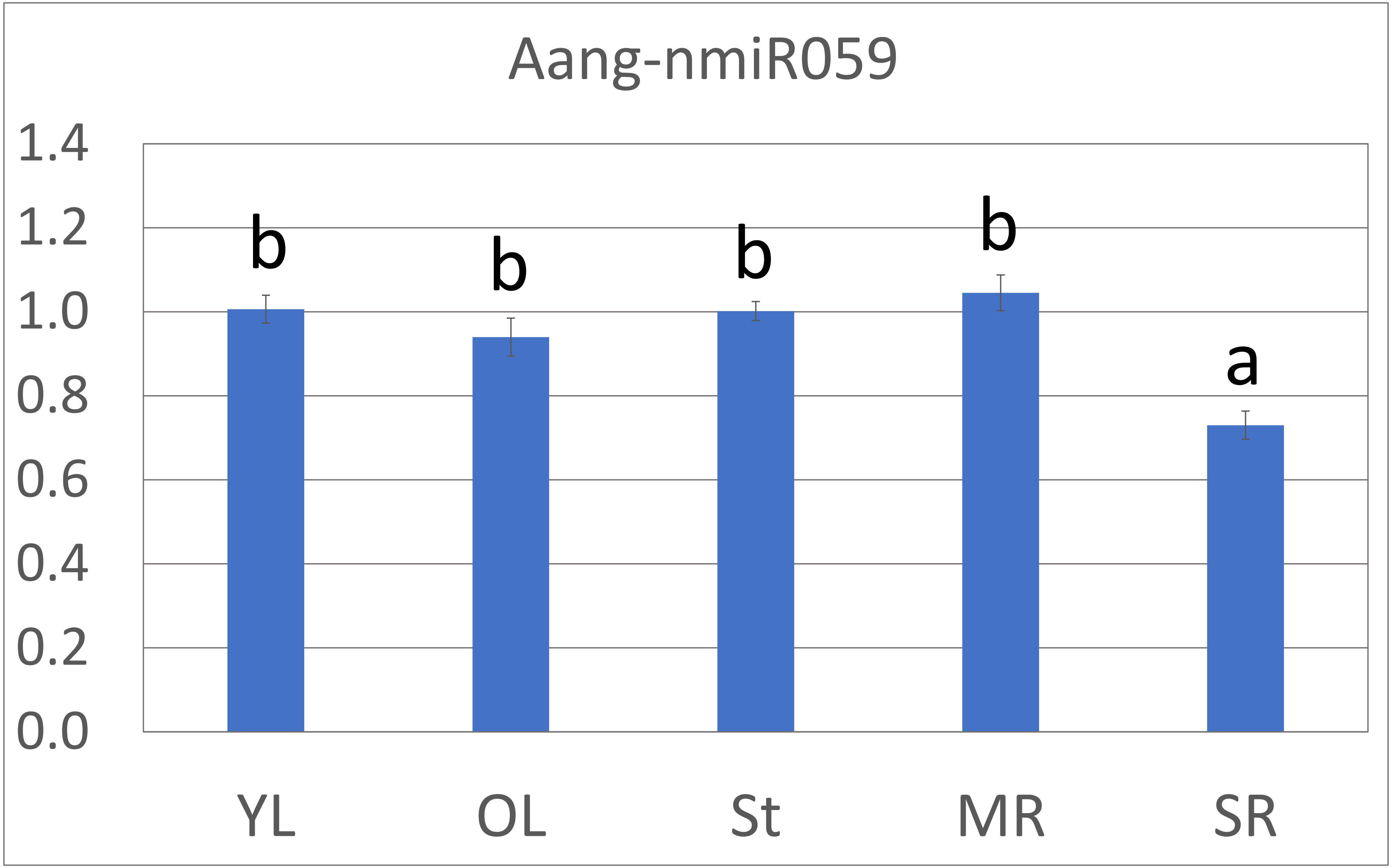
Aang-nmiR059
1.4
1.2
1.0
0.8
0.6
0.4
0.2
0.0
b
b
b
b
a
YL
OL
St
MR
SR

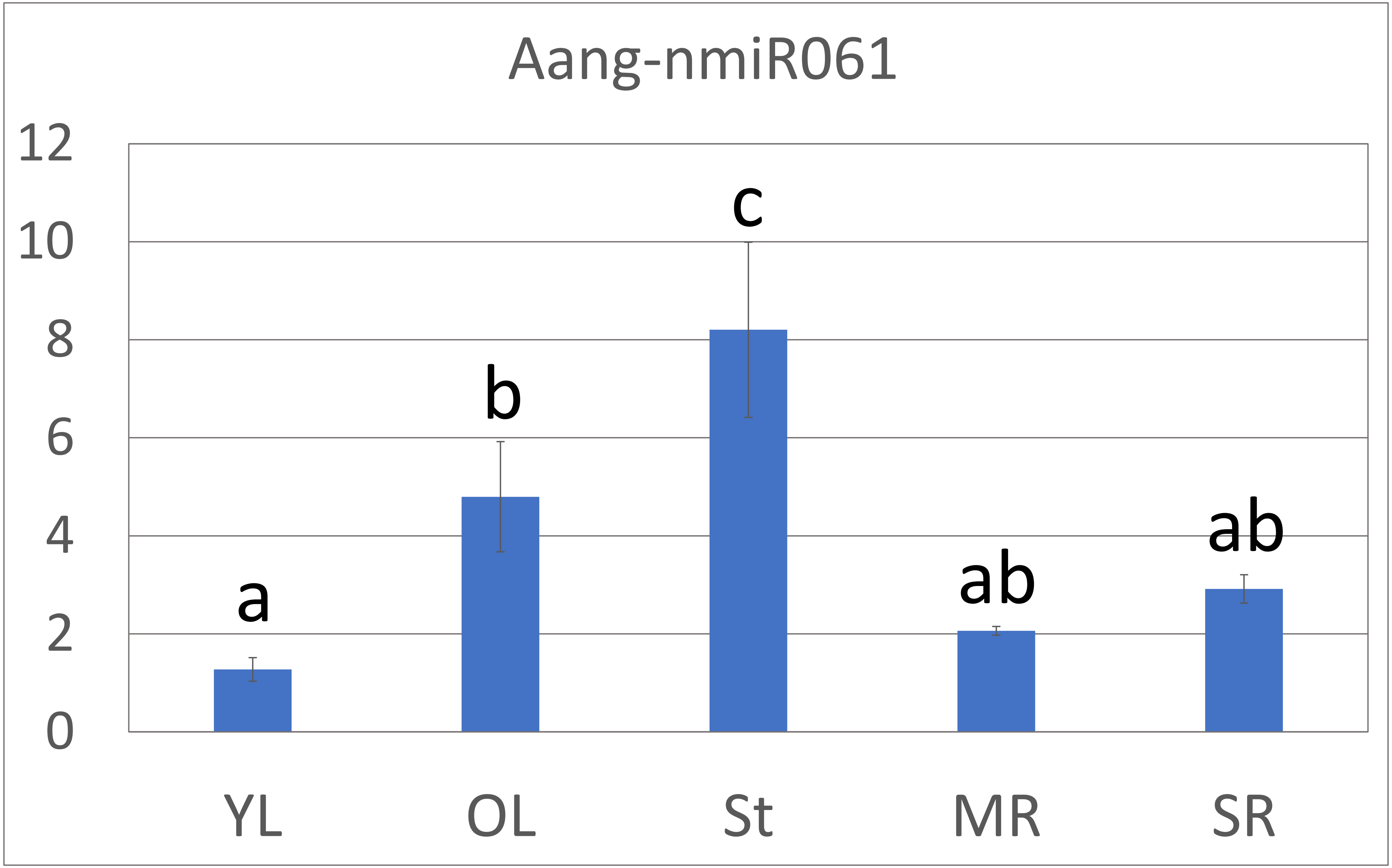
Aang-nmiR061
12
10
8
6
4
2
0
a
b
c
ab
ab
YL
OL
St
MR
SR
