# Supplementary material for: Novel and Conserved miRNAs Among Brazilian Pine and Other Gymnosperms
Source: Front Genet. 2019 Mar 22;10:222. doi: 10.3389/fgene.2019.00222 (PMC6448024; doi:10.3389/fgene.2019.00222)
Supplement: Supplementary file 7 [file Data_Sheet_7.pdf]

Data S7. **Expression patterns of conserved and novel *A. angustifolia* miRNAs in different tissues.** A) A three-month-old plant showing the tissues used for Stem-loop RT-qPCR assays. B) A schematic diagram of the expression patterns of 12 conserved miRNAs in five different tissues in *A. angustifolia*. C) A schematic diagram of the expression patterns of 30 novel miRNAs in five different tissues in *A. angustifolia*. The codes YF, OF, St, MR and SR indicate, respectively, young leaves, old leaves, stem, main root and secondary roots. The means of the samples were analyzed using one-way ANOVA followed by Duncan's multiple range tests. Different letters indicate statistical differences in expression patterns.

A

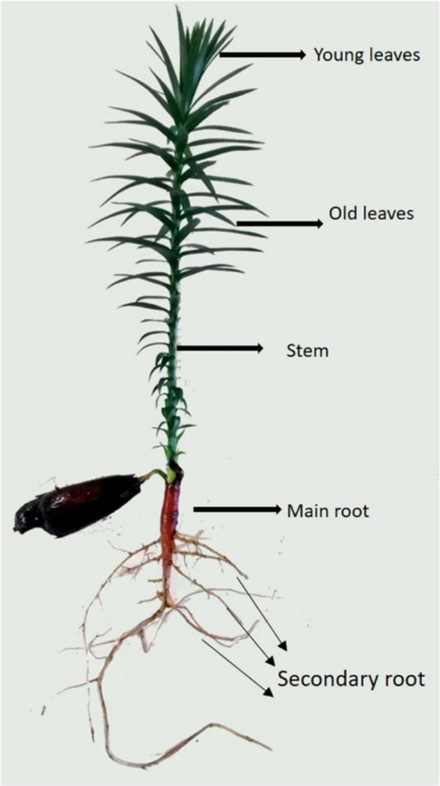

B

	YL	OL	St	MR	SR
miR167	c	ab	a	a	bc
miR399	a	d	ab	bc	cd
miR395	c	b	d	a	a
miR529	a	b	b	a	a
miR166	a	a	b	a	a
miR168	a	a	b	a	a
miR390	a	a	b	a	b
miR169	ab	a	a	b	ab
miR156	a	a	a	a	b
miR159	a	a	a	a	b
miR1314	a	ab	bc	ab	c
miR171	a	a	a	a	a

C

	YL	OL	St	MR	SR
nmiR029	b	a	a	ab	a
nmiR038	d	c	b	b	a
nmiR059	b	b	b	b	a
nmiR027	b	ab	a	b	b
nmiR057	ab	a	b	b	b
nmiR054	a	b	b	b	a
nmiR008	a	c	ab	bc	a
nmiR021	a	a	b	a	a
nmiR003	a	ab	c	ab	b
nmiR061	a	b	c	ab	ab
nmiR051	a	a	a	b	a
nmiR044	bc	b	a	c	b
nmiR023	a	ab	ab	c	bc
nmiR001	a	a	a	a	b
nmiR005	a	a	a	a	b
nmiR007	a	a	a	a	b
nmiR011	a	a	a	a	b
nmiR012	a	a	a	a	b
nmiR018	a	a	a	a	b
nmiR025	a	a	a	a	b
nmiR049	a	b	ab	a	c
nmiR019	a	b	ab	ab	c
nmiR017	a	b	b	ab	c
nmiR004	a	b	b	b	c
nmiR002	a	ab	ab	b	c
nmiR026	a	bc	b	bc	c
nmiR016	a	ab	bc	c	d
nmiR040	a	a	a	a	a
nmiR009	a	a	a	a	a
nmiR046	a	a	a	a	a
